# Rare Copy Number Variations in Adults with Tetralogy of Fallot Implicate Novel Risk Gene Pathways

**DOI:** 10.1371/journal.pgen.1002843

**Published:** 2012-08-09

**Authors:** Candice K. Silversides, Anath C. Lionel, Gregory Costain, Daniele Merico, Ohsuke Migita, Ben Liu, Tracy Yuen, Jessica Rickaby, Bhooma Thiruvahindrapuram, Christian R. Marshall, Stephen W. Scherer, Anne S. Bassett

**Affiliations:** 1 Toronto Congenital Cardiac Centre for Adults, Peter Munk Cardiac Centre, University Health Network, Toronto, Ontario, Canada; 2 Division of Cardiology, Mount Sinai Hospital, Toronto, Ontario, Canada; 3 The Centre for Applied Genomics and Program in Genetics and Genome Biology, The Hospital for Sick Children, Toronto, Ontario, Canada; 4 Department of Molecular Genetics and the McLaughlin Centre, University of Toronto, Ontario, Canada; 5 Clinical Genetics Research Program, Centre for Addiction and Mental Health, Toronto, Ontario, Canada; 6 Department of Psychiatry, University of Toronto, Ontario, Canada; University of Texas Medical School, United States of America

## Abstract

Structural genetic changes, especially copy number variants (CNVs), represent a major source of genetic variation contributing to human disease. Tetralogy of Fallot (TOF) is the most common form of cyanotic congenital heart disease, but to date little is known about the role of CNVs in the etiology of TOF. Using high-resolution genome-wide microarrays and stringent calling methods, we investigated rare CNVs in a prospectively recruited cohort of 433 unrelated adults with TOF and/or pulmonary atresia at a single centre. We excluded those with recognized syndromes, including 22q11.2 deletion syndrome. We identified candidate genes for TOF based on converging evidence between rare CNVs that overlapped the same gene in unrelated individuals and from pathway analyses comparing rare CNVs in TOF cases to those in epidemiologic controls. Even after excluding the 53 (10.7%) subjects with 22q11.2 deletions, we found that adults with TOF had a greater burden of large rare genic CNVs compared to controls (8.82% vs. 4.33%, p = 0.0117). Six loci showed evidence for recurrence in TOF or related congenital heart disease, including typical 1q21.1 duplications in four (1.18%) of 340 Caucasian probands. The rare CNVs implicated novel candidate genes of interest for TOF, including PLXNA2, a gene involved in semaphorin signaling. Independent pathway analyses highlighted developmental processes as potential contributors to the pathogenesis of TOF. These results indicate that individually rare CNVs are collectively significant contributors to the genetic burden of TOF. Further, the data provide new evidence for dosage sensitive genes in PLXNA2-semaphorin signaling and related developmental processes in human cardiovascular development, consistent with previous animal models.

## Author Summary

Congenital heart disease affects nearly 1% of all live births. Tetralogy of Fallot (TOF) is the most common form of cyanotic congenital heart disease. This condition is associated with hemizygous deletions of chromosome 22q11.2 and chromosomal trisomies, but little else is known about the genetic heterogeneity of this complex disease. We used high-resolution microarrays and stringent methods to study structural (copy number) variants in a systematically phenotyped cohort of unrelated adults with TOF. We found that individually rare genic copy number variants (CNVs) were collectively significant contributors to the genetic burden in TOF. Among CNVs that implicated candidate genes of interest were loss CNVs overlapping the PLXNA2 gene that codes for plexin A2. This is the first study to show a role for this semaphorin receptor in human congenital heart disease, consistent with a Plxna2 mouse knockout phenotype. Pathway analyses comparing rare exonic loss CNVs in the TOF sample to controls implicated other novel gene sets suggest new pathogenetic mechanisms.

## Introduction

Tetralogy of Fallot (TOF) is the most common form of cyanotic congenital cardiac disease in humans. With surgical advances and increased longevity, attention has shifted from immediate outcomes to understanding causation. However, for most patients with TOF, the genetic basis for the disease remains unknown. Recently, there has been a focus on unbalanced structural genomic changes, or copy number variants (CNVs), and disease [1]. Copy number variation contributes to the genetic heterogeneity of many complex human diseases [2], [3]. Investigation of CNVs that overlap genes has led to the discovery of novel etiologies and disease pathways, especially for developmental disorders [1], [4]–[6]. Our current understanding of the role of CNVs in the etiology of TOF, however, is limited. Early reports of CNVs in subjects with various types of congenital cardiac conditions, using low resolution methods, suggested that CNVs may be important [7]–[11] but there is just one report of genome-wide CNVs in 111 TOF patients using a high resolution microarray [12]. We used a high resolution genome-wide microarray and proven methods to: a) investigate the burden of rare CNVs in TOF compared to controls, b) identify putative candidate genes associated with rare and recurrent CNVs and c) assess, using a pathway analysis, whether the exonic CNVs found in TOF could identify functional gene sets relevant to cardiac development.

## Results

Of the 495 unrelated adults with TOF recruited, 53 (10.7%; including 49 of European ancestry) had 22q11.2 deletions associated with 22q11.2 deletion syndrome [61], four had chromosomal anomalies detectable on karyotype (two with XXY, one with XXX and one with a 16 Mb 18q22 deletion) and five had previously diagnosed genetic conditions for which clinical genetic testing is in progress (three with Holt-Oram syndrome, one with CHARGE syndrome and one with VACTERL association). The remaining 433 adults [239 (55.2%) male] formed the CNV discovery sample [mean age 32.56 (SD 12.29 years)]; 45 (10.39%) had pulmonary atresia and 57 (13.16%) were in the syndromic group.

Using a strict CNV analysis strategy [5], [6], [13], we detected 63 CNVs on average per genome in TOF cases with a median size of 18,020 (range 397–5,997,249) bp, similar to results for the controls (Tables 1, 2 and 3 in Supporting Information S1). To minimize false positives, we focused on rare CNVs using a conservative definition (<0.1% in population-based controls), and employed identical methods for both cases and the independent Ontario Population Genomics Platform (OPGP) controls used for case-control analyses (see Methods). The main quantitative analyses involved only those subjects of European ancestry. We compared rare CNVs in the 340 TOF cases and 416 OPGP controls of European ancestry. To assess the experimental reproducibility of rare CNVs after *in silico* detection, we tested 68 CNVs across different size ranges using quantitative PCR (qPCR). We observed a high true positive validation rate of 65/68 (95.6%), consistent with our previous studies [5], [6], [13].

### Rare CNV burden in TOF

We first compared the CNV burden of large (>500 kb) rare CNVs in the TOF cases and the OPGP controls. Consistent with our hypothesis, a significantly greater proportion of cases harbored large rare CNVs compared to controls (OR 1.89, 95% CI 1.06–3.35, p = 0.0278) (Table 1). This was most notable for differences in large gains that overlapped exons (OR 2.54, 95% CI 1.17–5.50, p = 0.0148). However, if the 49 individuals with TOF and 22q11.2 deletions of European ancestry had been included, the odds ratio for large rare exonic losses would also have been significantly higher compared to controls (OR 10.87, 95% CI 4.80–24.08, p<0.0001). In contrast, the overall quantitative burden of rare CNVs of any size was similar between the TOF group and OPGP controls; most TOF and OPGP control subjects had one or more rare CNVs (Table 1). When CNV burden for individuals was defined as having two or more rare CNVs, there was no significant difference between cases and controls (data not shown).

**Table 1:** Rare CNV burden in 340 unrelated adults with tetralogy of Fallot and/or pulmonary atresia.

	aRare CNV burden					aRare CNV burden in TOF subgroups				
	OPGP controls (n = 416)		All TOF cases (n = 340)		Analysis	Non-syndromic cases (n = 291)		Syndromic cases (n = 49)		Analysis
	n	(%)	n	(%)	p	n	(%)	n	(%)	p
**Large rare CNVs (>500 kb)**										
Loss or gain	21	(5.05)	31	(9.12)	**0.0278**	24	(8.25)	7	(14.29)	NS
Exonic (loss or gain)	17	(4.09)	30	(8.82)	**0.0073**	23	(7.90)	7	(14.29)	NS
Exonic loss	7	(1.68)	12	(3.53)	NS	5	(1.72)	7	(14.29)	b **0.0004**
Exonic gain	10	(2.40)	20	(5.88)	**0.0148**	18	(6.19)	2	(4.08)	bNS
**All rare CNVs (any size)**										
Loss or gain	372	(89.42)	298	(87.65)	NS	253	(86.94)	45	(91.84)	bNS
Exonic (loss or gain)	249	(59.86)	210	(61.76)	NS	174	(59.79)	36	(73.47)	NS
Exonic loss	139	(33.41)	131	(38.53)	NS	102	(35.05)	29	(59.18)	**0.0013**
Exonic gain	161	(38.70)	133	(39.12)	NS	113	(38.83)	20	(40.82)	NS

a Rare autosomal CNVs>10 kb and <6.5 Mb in size in individuals of European ancestry. Inclusion of three subjects with anomalies >6.5 Mb in a secondary analysis did not change the overall results (data not shown). Note that the above results also do not include 49 subjects of European ancestry with typical 1.5 to 3 Mb 22q11.2 deletions in the TOF group (all syndromic); see text for details on the results if these subjects had been included.

b Fisher's exact test.

### Rare CNV burden in TOF subgroups

For those subjects with TOF, large rare CNVs were enriched in the syndromic subgroup however this difference reached statistical significance only for subjects with large rare exonic losses (OR 9.53, 95% CI 2.89–31.41, p = 0.0004) (Table 1). These results would have been even more significant if individuals with 22q11.2 deletions had been included (data not shown). When individuals with one or more rare exonic loss CNVs of any size were considered, results were still significant but with a smaller OR (OR 2.69, 95% CI 1.35–4.60, p = 0.0013). A further TOF subgroup analysis comparing those with and without pulmonary atresia showed no significant enrichment of individuals with rare exonic loss CNVs in those with pulmonary atresia [48% (16 of 33) vs. 37% (115 of 307), p = 0.2162)].

### Large rare CNVs in TOF

Table 2 shows the 47 large (>500 kb) rare CNVs found in 43 of the 433 adults with TOF in the discovery sample. Most (39/47) were very rare, i.e., not found in any of 2,773 controls (2,357 population controls or 416 OPGP controls) and all but three overlapped genes. Several of these loci showed evidence for recurrence in TOF. The most compelling were 1q21.1 duplications (Figure 2 in Supporting Information S1) (OMIM #612475) identified in four (1.18%) of 340 subjects of European ancestry. None met our syndromic criteria, however detailed examination of the phenotype revealed macrocephaly in two and tall stature in another of these subjects.

**Table 2:** Rare large CNVs (>500 kb) in 43 of 433 unrelated adults with tetralogy of Fallot.

Case	CNV characteristics								
	Locus	Start	Size (bp)	CN	Very rare	Confirmed	Origin	# of genes	Putative candidate gene(s)
1	1p34.3	35,138,921	619,938	Loss	•	•		6	**SFPQ**
2	1q21.1	143,590,972	3,981,720	aGain	•	•	*de novo*	45	b **^,^** c **GJA5**
3	1q21.1	144,643,825	1,630,074	aGain	•	•		14	
4	1q21.1	144,643,825	1,667,590	aGain	•	•		14	
5	1q21.1	144,643,825	1,752,136	aGain	•	•		16	
6	1q41	220,214,502	1,051,264	Loss	•	•		7	**DISP1**
d7	2p23.2-p23.1	29,418,234	1,569,210	Gain	•	•		6	**LBH**
d8	2q11.2	97,982,662	548,388	Gain	•	•		3	
9	2q21.1	131,194,418	826,998	Loss	•	•		13	**ARHGEF4**
10	2q24.1	154,772,039	1,978,861	Gain	•	•	Inherited	2	
11	2q32.1	188,273,007	700,865	Loss	•	•		2	
12	3p26.3	60,002	995,032	Gain	•	•		1	**CHL1**
13	4p15.1	28,835,868	5,997,249	Loss	•	•		1	
14	4q35.2	188,923,641	514,206	Loss	•	•		3	
10	5q14.1-q14.3	80,665,064	5,454,818	Gain	•	•	Inherited	13	**EDIL3,** c **VCAN**
15	5q22.1	109,622,167	611,666	Gain	•	•		2	**SLC25A46**
16	5q23.3-q31.1	130,124,159	811,158	Gain	•	•		4	
17	6p22.3-p22.2	21,587,649	2,098,900	Gain	•	•		4	c **SOX4**
18	6q11.1	61,987,979	991,555	Gain	•	•	Inherited	1	
19	6q14.1	81,166,965	760,841	Gain	•	ND		0	
20	7p11.2-p11.1	57,264,874	645,786	Gain		ND		1	
d21	8p23.2	3,778,626	2,159,631	Gain	•	•		1	
d22	8p23.2-p23.1	6,187,821	1,037,609	Loss	•	•		20	b **ANGPT2**
23	8q11.1-q11.21	47,203,026	898,799	Gain	•	ND		1	
d24	9p13.1-p12	38,951,514	1,658,247	Gain		ND		6	
25	10q11.1-q11.21	41,941,187	752,815	Gain		ND		5	
26	10q26.13	125,510,180	743,304	Gain	•	•		5	
27	11q22.3	103,542,068	1,252,192	Gain		ND		7	**CASP1, CASP4, CASP5, CASP12**

Case	CNV characteristics								
	Locus	CNV start	CNV size	CN	Very rare	Confirmed	Origin	# of genes	Candidate gene(s)
d28	12p11.1	33,432,314	575,409	Loss	•	•		1	
29	12q24.32	125,358,126	793,244	Loss	•	•	Inherited	1	
d30	13q12.3-q13.3	31,086,497	4,726,890	fLoss	•	•	*de novo*	19	b **NBEA, PDS5B, SPG20**
d31	13q14.11	42,009,354	555,346	Gain	•	•		4	**TNFSF11**
32	13q33.1-13q33.2	103,096,790	825,415	Gain	•	ND	Inherited	0	
33	16p11.2	29,487,535	597,774	Gain	•	•		27	**HIRIP3,** c **MAPK3, PPP4C,** c **TBX6**
34	17p13.3	366,915	543,945	Gain		ND		9	**NXN**
35	17p13.2	5,528,898	667,804	Loss	•	•		1	
36	17p11.2	18,296,117	695,535	Gain		ND		15	**FOXO3B**
32	17q12	31,610,407	1,942,495	Gain		ND	*de novo*	25	**HNF1B**
37	18q22.3-18q23	69,207,652	3,406,071	gGain	•	•		13	**CNDP2**
37	18q23	72,614,319	3,501,711	gLoss	•	•		15	c **NFATC1, PARD6G**
38	19p13.3	183,496	805,727	Loss	•	•		34	**CNN2, FSTL3, PTBP1, WDR18**
d39	19p13.3	2,500,048	708,368	Gain	•	•		18	**GNA11, S1PR4**
d40	19q13.41	57,807,872	629,678	Gain	•	•		14	**ZNF347**
41	21q21.1	20,282,405	560,377	Gain	•	•		0	
42	21q21.3	29,607,482	637,303	Loss	•	•	Inherited	3	
41	22q11.21	17,190,110	2,174,700	Gain		•		44	**CRKL, HIRA, TBX1**
43	Xp22.2	13,788,639	615,940	Gain	•	•		2	

Case, subjects from discovery sample (n = 433) with TOF; Locus, cytogenetic location of CNV; CNV start, hg18 (NCBI Build 36.1, March 2006); CNV size, in base pairs; CN, type of copy number aberration; Very rare, not found in 2,773 controls (•), see text for details; Confirmed, by qPCR and/or FISH (•) or not done (ND); Origin, *de novo* or inherited (where known); # of genes, number of known genes overlapped by a CNV as annotated in the Database of Genomic Variants (http://projects.tcag.ca/variation/; September 2011); Candidate gene(s), selected based on reported cardiovascular system involvement; References derived from systematic searches of human (e.g., Online Mendelian Inheritance in Man; http://www.omim.org/) and model organism (e.g., Mouse Genome Informatics; http://www.informatics.jax.org/) databases presented in Table 4 in Supporting Information S1.

a Figure 2 in Supporting Information S1.

b Table 3.

c Neighbor of a top disease gene (GATA4, NKX2-5, TBX5), as identified in the pathway analysis (Table 11 in Supporting Information S1).

d Non-European ancestry.

f Previously reported by our group [21].

g Figure 3 in Supporting Information S1.

There were two other subjects in the non-syndromic subgroup with genomic disorders at loci previously associated with congenital cardiac disease: one proband with a previously undetected 22q11.21 duplication (OMIM #608363) [14] and another with a typical 16p11.2 duplication (OMIM #611913) [15].

Amongst other large rare CNVs of note, one proband with syndromic features had a novel tandem duplication-deletion in the 18q22.3-q23 region that was transmitted to her daughter. Both had TOF, learning difficulties, short stature, obesity, and thyroid disease. This complex CNV overlapped the region involved in the 18q22 deletion syndrome, e.g., the 16 Mb deletion of a subject excluded from our TOF cohort (Figure 3 in Supporting Information S1). There are three candidate genes in the distal end of the 3.5 Mb 18q23 deletion that may have an impact on cardiac development and/or are implicated by a relevant family of genes [16], [17] (Table 2, Figure 3 in Supporting Information S1): NFATC1, PARD6G and SALL3 [18]. Another proband with syndromic features had a 1q41 deletion that may overlap the region of a translocation reported in a patient with TOF [19] and possibly the 1q41 deletion region (OMIM #612530) associated with holoprosencephaly 10.

### Smaller very rare CNVs identifying genes of interest for TOF

The first section of Table 3 shows the smaller (<500 kb) very rare CNVs in the TOF sample that implicate specific candidate genes of interest at loci associated with TOF, including: GJA5 in the 1q21.1 duplication region (Figure 2 in Supporting Information S1) [20], CDH19 at 18q22.1 (Figure 3 in Supporting Information S1), NBEA at 13q13.3 [21] and ANGPT2 at 8p23.1 [22]. Other candidate genes highlighted through overlap with results from other studies include: CECR5 in the cat eye syndrome region [23], RAF1 involved in Noonan syndrome [12] and PPM1K [12].

**Table 3:** Very rare CNVs overlapping 26 putative candidate genes for tetralogy of Fallot.

	Candidate gene^a^	Locus	Case	CNV start	CNV size	CN	Exonic	Confirmed	Known CV involvement	Known structural CV phenotype
Candidate genes for TOF implicated through narrowing of critical regions	**GJA5**	1q21.1	44	145,700,719	10,347	bGain		•	•	•
			Cases 2 to 5: recurrent 1q21.1 duplications (Table 2)							
	**RAF1**	3p25.1	45	12,665,657	17,184	Loss	•	•	•	•
			Probands 756 and 419 from another study^c^							
	**ANGPT2**	8p23.1	46	6,342,621	59,662	Loss	•	•	•	•
			Case 22 (Table 2)							
	**NBEA**	13q13.3	47	34,960,143	20,620	Loss		ND	•	
			Case 30 (Table 2)							
	**CDH19**	18q22.1	48	62,118,826	254,033	dLoss	•	•	•	
			18q22 deletion syndrome (excluded case; see text)							
Candidate genes for TOF implicated by overlapping CNVs	**PLXNA2**	1q32.2	42	206,337,728	61,546	eLoss	•	•	•	•
			49	206,369,712	10,222	eLoss		•		
	**CHRM3**	1q43	50	237,983,869	381,637	Gain	•	ND	•	
			51	238,098,745	10,568	Loss		ND		
	**CCDC148**	2q24.1	52	158,961,179	51,207	Loss		ND		
			53	158,961,179	51,207	Loss		ND		
	**BARD1**	2q35	54	215,266,200	85,667	Loss	•	ND	•	
			55	215,321,117	59,317	Loss	•	ND		
	**PPM1K**	4q22.1	56	89,393,217	55,637	Gain	•	ND	•	•
			Proband 2231 from another study^c^							
	**FSTL4**	5q31.1	57	132,946,021	15,007	Loss		ND	•	
			58	132,953,135	10,498	Loss		ND		
	**GMDS**	6p25.3	56	1,938,284	12,749	Loss		•	•	
			59	1,939,891	11,142	Loss		•		
	**ARHGEF10**	8p23.3	60	1,716,624	156,826	Gain	•	ND	•	
			61	1,736,348	25,900	Loss	•	ND		
	**KCNB2**	8q13.3	62	73,871,523	78,480	Gain		ND	•	
			63	73,885,905	124,789	Gain		ND		
Candidate genes for TOF implicated by overlapping CNVs (continued)	**C12orf66**	12q14.2	20	62,843,704	78,289	Gain	•	ND		
			64	62,843,704	78,289	Gain	•	ND		
	**KIAA1609**	16q24.1	65	83,066,869	16,053	Loss	•	ND		
			66	83,094,514	26,382	Loss	•	ND		
	**CHST8**	19q13.11	67	38,902,591	26,593	Gain		ND	•	
			68	38,902,591	23,819	Gain		ND		
	**CECR5**	22q11.1	69	15,996,510	18,980	Loss	•	ND	•	
			47	16,014,050	84,862	Loss	•	ND		
	**WNK3**	Xp11.22	70	54,325,120	147,676	Gain	•	ND	•	
			32	54,374,737	50,162	Gain	•	ND		
	**IDS**	Xq28	71	148,308,784	153,622	Gain	•	ND	•	
			72	148,377,523	250,837	Gain	•	ND		
Selected candidate genes for TOF implicated by singleton CNVs	**FGF10**	5p12	73	44,314,920	135,646	Loss	•	•	•	•
			Associated with TBX1 and FGF3 (see text)							
	**SNX8**	7p22.2	74	2,310,838	180,068	Loss	•	ND	•	
			Within 7p22 deletion syndrome region (see text)							
	**DNAH11**	7p15.3	75	21,570,799	179,867	Loss	•	ND	•	•
			Associated with primary ciliary dyskinesis (see text)							
	**BBS9**	7p14.3	76	33,293,709	259,054	Loss	•	•	•	
			Associated with Bardet-Biedl syndrome (see text)							
	**SEMA3E**	7q21.11	77	82,968,279	52,328	Loss	•	•	•	•
			Associated with PLXNA2 (see text)							
	**SEMA3D**	7q21.11	78	84,477,068	9,591	Loss		•	•	•
			Associated with PLXNA2 (see text)							

Candidate gene, official HGNC symbol; Locus, cytogenetic location of candidate gene; Case, subjects from discovery sample (n = 433) with TOF; CNV start, hg18 (NCBI Build 36.1, March 2006); CNV size, in base pairs; CN, type of copy number aberration; Exonic, CNV overlaps exon of candidate gene (•); Confirmed, by qPCR (•) or not done (ND); CV involvement, known cardiovascular system involvement (•; not necessarily in human); Structural CV phenotype, known structural cardiovascular system phenotype associated with mutation (•; not necessarily in human); References derived from systematic searches of human (e.g., Online Mendelian Inheritance in Man; www.omim.org/) and model organism (e.g., Mouse Genome Informatics; http://www.informatics.jax.org/) databases are presented in Table 5 in Supporting Information S1.

a Novel and previously proposed candidate genes for TOF identified because of overlap with two or more CNVs in unrelated subjects (at least one Caucasian) with TOF, where the CNVs were not observed in 2,773 controls (see text). We have also shown selected candidate genes overlapped by very rare singleton CNVs in our cohort, including all those that overlapped rare CNVs reported by Greenway et al. [12].

b Figure 2 in Supporting Information S1.

c Greenway et al. [12].

d Figure 3 in Supporting Information S1.

e Figure 1.

**Figure 1:**
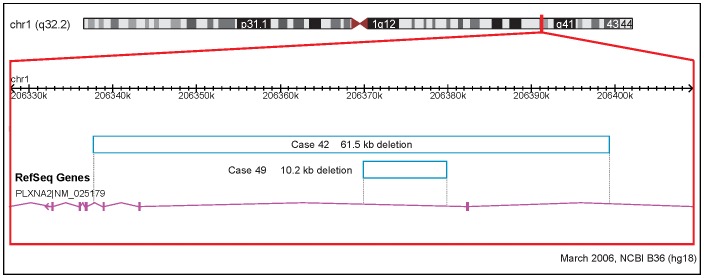
Rare CNVs overlapping novel candidate gene for tetralogy of Fallot: PLXNA2. Solid and open bars represent gains and losses, respectively; genomic parameters from NCBI Build 36.

Table 3 also shows novel very rare (not found in 2,773 controls) CNVs overlapping genes with evidence for cardiovascular involvement. Two unrelated probands had 1q32.2 loss CNVs overlapping the PLXNA2 gene (Figure 1), which were confirmed by qPCR and sequencing across the junction breakpoints. Plexins play an important role in cardiac development, including cardiac neural crest cell migration and outflow tract morphogenesis [24]. We therefore resequenced PLXNA2 exons and splice sites in a subset (n = 192) of the TOF cases of European ancestry. This yielded nine missense variants but no additional nonsense or frame-shift mutations that would lead to haploinsufficiency of the gene (Table 6 in Supporting Information S1). No point mutations were detected in the two individuals with PLXNA2 deletions and *in silico* inspection of the intronic CNV revealed no conclusive evidence of regulatory region disruption. Two other loss CNVs involved adjacent semaphorin genes at 7q21.11 with previous evidence for structural cardiac phenotypes (Table 3). One overlapped three exons of the SEMA3D gene coding for semaphorin 3D and the other overlapped the first intron of the SEMA3E gene, previously associated with CHARGE syndrome [25].

We also identified a group of four subjects with novel small rare CNVs containing genes associated with ciliary dysmotility: DNAH11 (n = 2), BBS9 (n = 1) and SNX8 (n = 1). Primary ciliary dyskinesis has several genetic causes, including mutations in DNAH11, a gene coding for a dynein heavy chain component of the axoneme, the inner cytoskeletal core of cilia (OMIM #6033). Similarly, BBS9 is one of 14 genes known to be responsible for Bardet-Biedl syndrome, a multisystem disorder [26]. Loss of ciliary function results in a multisystem disease and loss of function during embryogenesis can lead to congenital cardiac lesions, typically abnormalities of cardiac situs (heterotaxy), and less commonly TOF [27].

FGF10 was another plausible candidate gene for human congenital cardiac disease [28]–[30] implicated by a very rare exonic loss CNV. FGF10 codes for fibroblast growth factor 10, a protein with dosage sensitive expression in several aspects of early murine cardiovascular development [28], [29]. Notably, the loss CNV involved the entire gene, thus would encompass an evolutionarily conserved cis-regulatory module in intron 1 of the FGF10 gene recently reported to be functional during human cardiac development [30]. The proband with this CNV did not meet criteria for lacrimoauriculodentodigital (LADD) syndrome (OMIM #149730) or autosomal dominant aplasia of lacrimal and salivary glands (ALSG; OMIM #180920), conditions associated with point mutations in FGF10 coding regions that may have different expression from that of an intronic point mutation [30] or a structural variant alone.

### Pathway analysis

**Figure 2:**
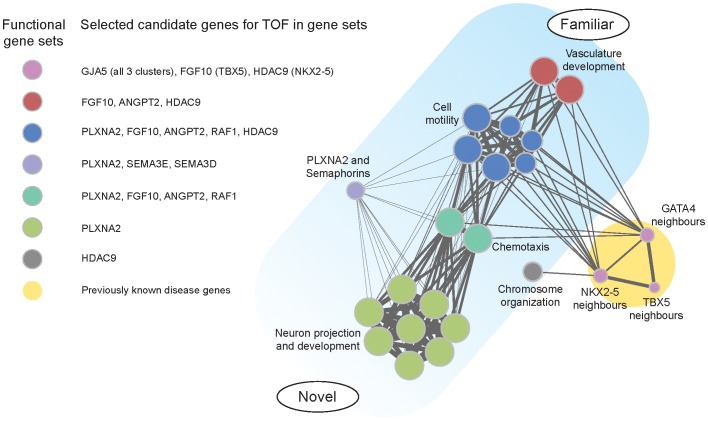
Functional clusters of candidate genes for tetralogy of Fallot. Diagram of results of pathway analyses comparing rare CNVs in cases and controls. Five overlapping functional clusters involved 19 gene-sets; functional neighbors of three known candidate genes identified another cluster (circle size indicates relative number of cases involved).

In pathway analyses testing of case and control subjects with rare CNVs that overlapped 6 or fewer genes, only exonic losses led to significant results for the gene-set test (permutation FDR< = 27.5%, nominal p-value< = 0.05) (Table 7 in Supporting Information S1), in line with previous findings for autism [5]. Nineteen gene-sets passed the significance thresholds (Table 8 in Supporting Information S1) and were selected for visualization (Figure 2). The gene-sets identified belonged to five overlapping functional clusters, representing both those expected and more novel (Figure 2): vasculature development (p = 0.0351), chromosome organization (p = 0.0224), cell motility (p = 0.0224), chemotaxis (p = 0.0440) and neuron projection and development (p = 0.0440). We also selected the three top-scoring previously reported TOF disease genes (GATA4, NKX2-5 and TBX5) and identified as potential disease candidates their high-confidence functional neighbors affected by a rare exonic CNV in two or more cases and none in controls (Table 10 and Table 11 in Supporting Information S1). PLXNA2-semaphorins was the only gene-set found exclusively in our systematic CNV review.

Integrating the results of pathway analysis and systematic CNV review, we identified potentially important convergences (Figure 2). GJA5 was found in all disease gene neighborhoods. ANGPT2 and FGF10 were found in vasculature development, cell motility, chemotaxis and in association with at least one of the disease genes. PLXNA2 was found in cell motility, chemotaxis and neuron projection and development. In contrast, HDAC9 was a novel gene identified by gene-set association (vasculature development and other clusters) and disease gene neighbor analysis (NKX2-5), but not in our systematic CNV review. Figure 4 in Supporting Information S1 presents results of a manual review of further lines of evidence to reconstruct putative regulatory relations between the candidate genes in a potential disease pathway.

## Discussion

Copy number changes appear to be important genetic variants contributing to the etiology of TOF, with rare exonic losses occurring more frequently in patients with TOF than in controls. Many CNVs associated with TOF appear to disrupt gene pathways that control cell migration and vasculature development, both potentially important in cardiac development [31]. Notably, several plausible candidate genes for TOF were implicated in humans for the first time, including the PLXNA2 gene and related pathways. Our findings suggest that individually rare structural genomic changes are important contributors to the collective genetic burden of TOF.

Based on recommendations from the International Standard Cytogenomic Array (ISCA) Consortium, it is now suggested that chromosomal microarrays be used as the first-tier diagnostic test for patients with multiple congenital anomalies and/or unexplained developmental delay [32]. This recommendation is based on the higher diagnostic yield of genetic testing, specifically as it relates to the high sensitivity of detecting submicroscopic deletions and duplications. Many patients seen in adult congenital cardiac clinics, including those with TOF, will meet these criteria [33]. Notably, our data suggest that clinical screening for syndromic features will likely be insufficient to identify patients with large, pathogenic gains. In contrast, large rare losses may more often be associated with complex phenotypes [1]. In the next decade, many more CNVs associated with congenital heart disease will likely be discovered. The results of the current study will contribute to the strategies used to assess the pathogenicity of a CNV for TOF.

After 22q11.2 deletions, the most common large rare CNV in our TOF cohort was the recurrent 1q21.1 duplication. The 1.18% prevalence of the 1q21.1 duplication is consistent with results using a targeted assay in two previous studies of TOF [12], [20] and with early reports of CNVs at the 1q21.1 locus where variable expression included TOF and neuropsychiatric conditions [34]. Congenital cardiac defects that have been reported to be associated with 1q21.1 duplications include TOF [12], [20], ventricular septal defect [35], univentricular heart [35] and unspecified complex congenital cardiac disease [36]. Results of the current study, including pathway results and a very rare CNV overlapping GJA5 in this 1q21.1 CNV region (Figure 2 in Supporting Information S1, Table 3), add to previous studies that implicated GJA5 as a promising candidate gene for TOF [12], [20]. GJA5 codes for connexin40, a gap junction protein in a protein family known to be important in cardiac development and shown to be associated with TOF in mice [37]. Point mutations in GJA5 have also been reported in patients with arrhythmias [38], [39].

This is the first study to report 1q32.2 deletions at the PLXNA2 locus in patients with congenital heart disease. The PLXNA2 gene codes for a transmembrane protein, plexin A2 [40]. Plexin A2 is a receptor for semaphorin C3, which acts as a guidance molecule and is necessary for neural crest influx and endothelial cell function during outflow track septation [41]–[43]. In PLXNA2 knockout mice, congenital cardiac defects, including TOF, have been described [43]. Interactions with other plexins and transcription factors that control neural crest cell migration may also be important in the development of congenital cardiac lesions [44]–[47].

There are multiple semaphorin-plexin pathways. We identified two subjects with loss CNVs involving semaphorin genes: a 7q21.11 deletion overlapping three exons of semaphorin 3D (SEMA3D) and a 7q21.11 deletion intronic to semaphorin 3E (SEMA3E) (Table 3). Semaphorin 3D has been shown to be expressed in the cardiac cushions of chick heart and ventricular trabeculae [48]. Semaphorin 3E is involved in modulating the NOTCH signaling pathway via a VEGF feedback mechanism [49]. Although most commonly due to mutations involving the chromodomain helicase DNA-binding protein-7, CHARGE syndrome can be caused by mutations in the SEMA3E gene [50].

These CNV-related results direct attention to novel genes potentially involved in cardiac development in humans, and extend data from previous animal and human studies. For example, the migration of cardiac neural crest cells into the outflow tract is a process orchestrated, in part, by PLXNA2 signaling [51]. PLXNA2-semaphorin signaling is also implicated in guidance of both blood vessels and nerves [31]. Placed in this context, gene-set clusters labeled “Neuron projection and development” are compelling candidates for importance in cardiac development. Our findings implicating ciliary genes are also consistent with involvement of processes such as migration of cardiac neural crest into the outflow tract and parallel guidance of blood vessels and nerves in development. A further novel finding, from the pathway analysis, indicated HDAC9, a gene previously linked to muscle development and cardiac hypertrophy in mouse and human [52]–[54].

There is a known association between ciliopathies and congenital heart disease. In the current study, we found four cases with rare CNVs overlapping genes responsible for ciliary motility disorders: primary ciliary dyskinesis, Bardet-Biedl syndrome and 7p22 deletion syndrome. Two subjects had CNVs overlapping the DNAH11 gene, one of the genes responsible for primary ciliary dyskinesis. The cardiac lesion in this condition is believed to be due to an abnormality of nodal ciliary motility during development. In addition to abnormalities of cardiac situs (left and right sided heterotaxy), other congenital lesions including TOF have been reported in humans [55], [56] and mice [57]. Another individual with TOF had a very rare loss CNV that overlaps exons 10–21 of BBS9. Although the exact function of the BBS9 gene has not yet been determined, mutations in this gene are known to cause Bardet-Biedl syndrome, another classic ciliopathy that can affect multiple systems and has a highly variable phenotype [26]. Deletion of exons 5–20 in the BBS9 gene was recently reported to have a severe phenotype but this did not include a congenital cardiac defect [58]. The SNX8 gene, coding for sorting nexin 8, lies in the region of overlap of two previously reported 7p22.2 deletions with associated cardiac malformations, including one with TOF [59]. Although the role of SNX8 in development is unknown, sorting nexins have recently been implicated in ciliogenesis [60]. Because of the variable expressivity of the phenotype in ciliopathies, it is possible that patients with congenital cardiac lesions, including TOF, who have these conditions may be undiagnosed [56].

Most of these CNV findings remain in the research realm with functional studies essential to determine the true role of such variants and candidate genes in cardiac maldevelopment. Even the possible role for 1q21.1 duplications in the genetic burden of TOF, detectable by clinical genome-wide and targeted microarrays, requires more data to delineate the associated breadth and penetrance of cardiac and extracardiac expression [34]. There remains a large cohort of adults with TOF of, as yet, unknown etiology. The candidate genes and pathways identified in our study should help to inform subsequent genetic, including sequencing-based, studies of TOF. Most other variants identified will be rarer and of as yet uncertain clinical significance. Nevertheless, these results contribute to our understanding of pathogenesis in TOF - a crucial step towards future clinical applicability of genetic investigations.

### Advantages and limitations

This is the first study of genome-wide CNVs in TOF to use a well-characterized cohort of adult patients, stringent molecular methods, and multiple converging analyses. We have identified novel candidate genes for TOF, in addition to providing replication of previous findings, including some from a smaller genome-wide study of CNVs in TOF [12]. There are, however, limitations to this novel study. The conservative laboratory and CNV analytic methods used, including the restricted focus on rare CNVs at the <0.1% level, may have resulted in missing some rare variants of interest. However, the fact that we used the same approach and adjudication control set to determine rarity meant that our *a priori* decision to minimize false positives, at the expense of such false negatives, would be expected to affect both cases and controls equally. Although our results overlapped certain previously described CNVs, further replication studies will be important to help define the significance and relative prevalence of the novel rare CNVs identified in this study. Large, multicentre studies may be useful, provided that comparable phenotyping and stringent quality control methods, as highlighted in this study, are maintained [2]. Meta-analyses could clarify if the lack of evidence for two or more rare CNVs per subject, as previously found for 22q11.2 deletions [61], is due to insufficient power. Family studies are also needed to delineate inherited or *de novo* status and segregation patterns of CNVs. These data will be essential to determine the true penetrance and variable expression of individual CNVs. Examining CNVs in patients with other forms of conotruncal defects or other forms of congenital heart disease may also be informative, and could reveal a genetically-related spectrum of clinically-distinct cardiac maldevelopment as is increasingly appreciated for, e.g., neuropsychiatric disorders [2]. Other study designs, e.g., using whole genome sequencing, will be needed to fully delineate the genetic architecture of TOF, including detection of relevant sequence-based mutations, such as those in non-coding regulatory regions that may be important for cardiac development [30]. Pathway analyses were restricted to subjects with rare CNVs overlapping 6 or fewer genes, and insufficient numbers precluded separate analyses involving only large rare CNVs. However, pathway results were similar when subjects with multigenic CNVs overlapping >6 genes were included (data not shown). Lastly, proving causality of specific genetic variants is beyond the scope of this study and more evidence, including replication of association in independent cohorts, will be needed to corroborate our putative candidate genes for tetralogy of Fallot. Fortunately, the functional significance of several key candidate genes implicated by our CNV results has already been validated in model organisms such as mice and zebrafish.

### Conclusions

In addition to well known 22q11.2 deletions, other structural genomic changes appear to be important contributors to the genetic heterogeneity of TOF. In particular, these include 1q21.1 duplications and other rare copy number changes that disrupt genes involved in cell migration and vasculature development pathways including PLXNA2-semaphorin signaling and perhaps ciliary motility. Further studies will help to improve our understanding of the complex etiology and pathogenesis of TOF and of congenital heart disease in general.

## Methods

### Ethics statement

The study was approved by institutional research ethics boards at the University Health Network and the Centre for Addiction and Mental Health.

### TOF sample

We prospectively recruited 495 unrelated adults (≥18 years) with TOF including a subset with TOF-pulmonary atresia or pulmonary atresia and ventricular septal defect (collectively termed “TOF” in this study), without autosomal trisomies, from a single clinic (Toronto Congenital Cardiac Centre for Adults). Patients with pulmonary atresia in the setting of more complex cardiac lesions, such as single ventricle lesions or transposition complexes, were not included. We excluded 62 subjects with documented syndromes, including 53 with 22q11.2 deletion syndrome associated with 1.5 to 3.0 Mb 22q11.2 deletions and genome-wide CNV data reported elsewhere [61]. The remaining 433 subjects formed a CNV discovery sample for this study.

TOF diagnosis was confirmed using echocardiogram and/or cardiac catheterization together with other imaging and surgical data reviewed using lifetime medical records [62]. All subjects underwent direct clinical screening for potential syndromic features [63]; available medical records were also reviewed. Subjects were stratified into syndromic and non-syndromic subgroups using criteria previously validated for identifying 22q11.2 deletion syndrome in adults [63]. Individuals with at least two of three features (history of learning difficulties, global dysmorphic facial features, hypernasal voice) were placed in the syndromic subgroup [63]. All phenotyping was done blind to genotype. Further details regarding cardiac and extracardiac phenotypes are provided elsewhere [33]. We were underpowered to perform subgroup analyses involving individuals with specific congenital cardiac outcomes (e.g., heart failure).

### Control sample for formal analyses

To optimize our analyses, we used an independent Canadian control sample from the Ontario Population Genomics Platform (OPGP) genetic epidemiological project that comprised adults of European ancestry [(208 (50.0%) male; mean age 44.96 (SD 12.05) years]. To maximize quality control and minimize artefactual/laboratory-related findings [64], all OPGP control samples were handled and experiments performed by the same laboratory using identical array methods and protocols, including CNV analyses and rarity assignation using separate large control cohorts, as for the TOF cases (see below).

### Genotyping

High quality genomic DNA was genotyped using the high resolution Affymetrix Genome-Wide Human SNP Array 6.0. CNV analysis and adjudication for all TOF case and OPGP control samples were performed at The Centre for Applied Genomics (Toronto, Canada). Arrays meeting Affymetrix-recommended quality control guidelines of contrast QC>0.4 were used for further analysis as outlined below and in Figure 1 in Supporting Information S1.

### Ancestry

To accurately estimate ancestry, in addition to self-reported ethnicities genotypes of the TOF cases from 1,120 genome-wide unlinked SNPs were clustered by the program STRUCTURE [65] together with those from 270 HapMap samples, which were used as references of known ancestry during clustering. Ancestries were assigned with a threshold of coefficient of ancestry >0.9. Of the 433 TOF subjects, there were 340 of European, 61 of Admixed, 27 of East Asian and 5 of African ancestry.

### CNV determination, adjudication, and prioritization

Genome-wide CNVs were determined using a multiple-algorithm approach to maximize sensitivity and specificity of CNV calling, as described previously [13]. Briefly, for each subject we defined “stringent” CNV calls as those detected by at least two of three different CNV calling algorithms: Birdsuite [66], iPattern [67] and Affymetrix Genotyping Console, and spanning 10 kb in length and five or more consecutive array probes. In this dataset, the mean number of calls per sample was 51, 50 and 32 for Birdsuite, iPattern and Genotyping Console, respectively. Overlapping calls at the sample level from Birdsuite and iPattern were merged with the outside probe boundaries. Singleton calls from iPattern or Birdsuite were also included in the stringent CNV set if they overlapped with a Genotyping Console call from the same sample. On average, 59% of CNVs in a sample were stringent. All subsequent analyses focused on the stringent CNVs, which in our experience have very high positive validation rates by independent methods such as quantitative PCR [5], [6], [13]. Merging CNV calls on a sample level across different algorithms has the additional advantage of correcting for the tendency of individual algorithms to segment single CNV events into multiple calls.

Each stringently defined CNV identified in the TOF case and OPGP control samples was then adjudicated for rarity by comparison to those CNVs identified in two large population-based control cohorts comprising 2,357 individuals of European ancestry from Ontario and Germany, which had already been assessed using an identical microarray platform and CNV analysis strategy (i.e., as above) [13]. We adopted a conservative definition of rare CNVs, retaining only those CNVs present in <0.1% of these 2,357 population controls. Further details of the comprehensive adjudication methods, including assessment of segmental duplications and Database of Genomic Variants (http://projects.tcag.ca/variation/) CNVs, may be found elsewhere [5], [13].

CNVs>6.5 Mb in size, likely to be detectable by karyotype and/or potentially indicating artefactual results, were excluded. To ensure consistency of data, for major analyses we used only autosomal CNVs>10 kb in size in individuals of European ancestry (Figure 1 in Supporting Information S1).

Large CNVs were defined as those >500 kb in size. We prioritized smaller CNVs (<500 kb) meeting the following criteria for more detailed examination: a) very rare (i.e., not present in any control sample using a 50% reciprocal overlap criterion) [13] and b) recurrent in unrelated TOF subjects, including those reported in the literature, and/or c) overlapping ‘interesting’ gene(s) possibly involved in TOF. When available, immediate relatives were studied using the same methods as for the proband to determine if a CNV was *de novo* or inherited.

### Experimental validation of CNVs

Confirmatory studies of possible TOF-associated CNVs used Stratagene SYBR Green based quantitative-PCR (qPCR). Each qPCR assay was performed in triplicate, for both the target region and for a control region at the FOXP2 locus on chromosome 7. Where available, molecular cytogenetic or microarray results from clinical laboratories also confirmed CNVs.

### Sequencing and mutation screening

For candidate gene discovery in TOF we prioritized further sequencing characterization to a single gene selected based on our CNV results and previous animal model studies to be the most likely to be involved in cardiac development. We performed mutation screening of PLXNA2 coding sequence (spanning 5,682 nucleotides) using standard PCR-based Sanger sequencing. The PLXNA2 gene contains 31 coding exons (67 to 1,268 bp) that were fully sequenced with 32 amplicons. The program Primer 3 (http://frodo.wi.mit.edu/primer3/) was used to design primers. The amplified products were sequenced with the Big Dye Terminator kit using the ABI 3730XL capillary sequencer (Applied Biosystems) and analyzed for sequence variants using Sequencher (Gene Codes, Ann Arbor, MI, USA). Putative sequence variants of interest were confirmed by sequencing in the reverse direction. SIFT [68] and POLYPHEN [69] were used for in-silico prediction of the effect of missense variants on protein function.

### Statistical methods

Statistical analysis was performed using SAS software (version 9.3, SAS Institute Inc., Cary, NC, USA). The main analyses compared rare CNVs in the 340 TOF cases of European ancestry with those in the 416 OPGP controls and within-group comparisons of syndromic versus non-syndromic TOF subjects. Chi-square or Fisher's exact tests were used to compare categorical variables and Student's t tests for continuous variables, as appropriate. All tests were two-side, with statistical significance defined as p<0.05.

### Pathway analyses

For pathway analyses, we first assessed if pre-defined gene-sets (corresponding to biological functions and pathways) displayed a higher rare CNV load in TOF cases than in OPGP controls. Gene-sets were derived from Gene Ontology annotations (downloaded from NCBI in April 2011 and up-propagated according to ontological relations), pathway databases (KEGG, Reactome, BioCarta, NCI; March 2011) and protein domains (PFAM; March 2011). Only gene-sets with a number of member genes between 25 and 750 were tested: 2,456 total, with 1,939 from GO, 414 from pathways and 103 from PFAM domains. Gene-sets with fewer than 25 genes decrease the statistical power of the analysis, whereas those with more than 750 genes tend to have a very broad biological scope (e.g. GO “regulation of biopolymer catabolism”) and hinder the visualization of results. Subjects with rare CNVs overlapping more than 6 genes were not considered for the gene-set analysis, as these may have a more promiscuous set of gene functions perturbed by the rare variant. For exonic losses this led to the exclusion of 14 TOF cases and 11 OPGP controls.

For each gene-set, we built a contingency matrix with subjects of European ancestry as sampling units. Subjects were categorized as (a) TOF cases or OPGP controls and (b) having at least one gene-set gene harboring a rare CNV or not. On the basis of this contingency table, a one-tailed Fisher's Exact Test was used to test higher prevalence of rare CNVs in TOF probands versus OPGP controls. This test can be regarded as an extension of a single-gene or single-variant association test; however, testing association for groups of genes, unlike single genes or single variants, provides sufficient power to detect significant association even when considering only rare variants [70]. To map CNVs to genes we used a stringent method, and restricted to CNVs overlapping exons. We tested all types of variants as well as losses-only and gains-only; only losses produced significant results (see analysis method below), in line with our previous findings for autism [5].

The Fisher's Exact Test nominal p-value was corrected for multiple tests using a case/control class permutation procedure to estimate an empirical false discovery rate. We favored a permutation strategy over classical Benjamini-Hochberg false discovery rate owing to the highly complex dependency structure among gene-sets and overly conservative nature of this test [5]. Case and control labels were permuted 2,000 times, and for each permutation gene-sets were tested following exactly the same procedure. Real nominal p-values were ranked from lowest (most significant) to highest (least significant) and, for each real p-value, the empirical false discovery rate was computed as the average number of gene-sets with equal or smaller p-value over permutations. Therefore, the empirical false discovery rate can be interpreted as an estimate of the fraction of gene-sets that would be significant under the null hypothesis of no association at the chosen nominal significance level. We selected 27.5% as the empirical false discovery rate significance threshold for final results; we additionally required the nominal p-value to be <0.05.

Previously known TOF disease genes (Table 9 in Supporting Information S1) were scored for association following a similar strategy, but using functional neighbors instead of functional gene-sets. For each known disease gene, we scored TOF case and OPGP control subjects of European ancestry. The score was defined as the highest functional weight between (a) the known disease gene being tested and (b) the CNV-harboring genes in the subject being scored. The functional weight was obtained from STRING, a publicly available resource that predicts the probability of two genes participating in the same pathways based on physical interaction, pathway membership, co-expression and PubMed co-citation. For each TOF disease gene, we tested if functional neighborhood scores were higher in TOF cases compared to OPGP controls by logistic regression analysis. All exonic CNVs (gains and losses) were used; unlike the gene-set association test, restricting to losses did not improve significance. We finally selected the three top-scoring known disease genes (GATA4, NKX2-5, TBX5).

For visualization, we integrated results from gene-set association, disease gene neighborhood analysis and systematic CNV review as a gene-set overlap network using the Cytoscape plugin Enrichment Map [5], [71]. Gene-sets significant after the gene-set association test were restricted to genes with higher prevalence in TOF cases than in OPGP controls [5], whereas functional neighborhood gene-sets included the known TOF disease gene as well as its neighbors that had high interaction confidence according to STRING (score>700, equivalent to interaction probability >70%) and harbored CNVs in 2 or more TOF case subjects but no OPGP control (Table 10 in Supporting Information S1). The combined jaccard-overlap index was used to generate the gene-set network, setting a threshold of 0.2. Clusters of overlapping gene-sets were manually identified and colored.

## Supporting Information

Supporting Information S1 Table 1: List of rare CNVs in 340 TOF and/or pulmonary atresia cases of European ancestry. Table 2: List of rare CNVs in 416 OPGP control individuals. Table 3: Summary of Affymetrix 6.0 microarray CNV data TOF sample (N = 340). Table 4: Rare large CNVs (>500 kb) in 43 of 433 unrelated adults with tetralogy of Fallot. Table 5: Very rare CNVs overlapping 26 candidate genes for tetralogy of Fallot. Table 6: *PLXNA2* sequence variants detected in 192 unrelated TOF cases of European ancestry. Table 7: Gene-set association results for all gene-sets tested, rare CNVs restricted to exonic losses. Table 8: Additional gene-set information for the 19 gene-sets selected for final results. Table 9: Known TOF disease genes used for the disease gene neighborhood analysis. Table 10: Test results on disease gene neighborhoods for all disease genes, using the STRING network. Table 11: Neighbor gene details for the three top disease genes. Figure 1: Overview of study design and CNV analysis workflow. Figure 2: Rare CNVs at chromosome region 1q21.1 in TOF cases. Figure 3: Rare CNVs at chromosome region 18q22.3-q23 in TOF cases. Figure 4: Integrated TOF pathway and candidate gene connectivity. Supplementary References.
(DOC)
