# Supplementary material for: Rare Copy Number Variations in Adults with Tetralogy of Fallot Implicate Novel Risk Gene Pathways
Source: PLoS Genet. 2012 Aug 9;8(8):e1002843. doi: 10.1371/journal.pgen.1002843 (PMC3415418; doi:10.1371/journal.pgen.1002843)
Supplement: Supporting Information S1 — Table 1: List of rare CNVs in 340 TOF and/or pulmonary atresia cases of European ancestry. Table 2: List of rare CNVs in 416 OPGP control individuals. Table 3: Summary of Affymetrix 6.0 microarray CNV data TOF sample (N = 340). Table 4: Rare large CNVs (>500 kb) in 43 of 433 unrelated adults with tetralogy of Fallot. Table 5: Very rare CNVs overlapping 26 candidate genes for tetralogy of Fallot. Table 6: PLXNA2 sequence variants detected in 192 unrelated TOF cases of European ancestry. Table 7: Gene-set association results for all gene-sets tested, rare CNVs restricted to exonic losses. Table 8: Additional gene-set information for the 19 gene-sets selected for final results. Table 9: Known TOF disease genes used for the disease gene neighborhood analysis. Table 10: Test results on disease gene neighborhoods for all disease genes, using the STRING network. Table 11: Neighbor gene details for the three top disease genes. Figure 1: Overview of study design and CNV analysis workflow. Figure 2: Rare CNVs at chromosome region 1q21.1 in TOF cases. Figure 3: Rare CNVs at chromosome region 18q22.3-q23 in TOF cases. Figure 4: Integrated TOF pathway and candidate gene connectivity. Supplementary References. (DOC) [file pgen.1002843.s001.doc]

**SUPPLEMENTARY MATERIAL**

**This file includes the following:**

**Supplemental data**

Supplementary Table 1: List of rare CNVs in 340 TOF and/or pulmonary atresia cases of European ancestry

Supplementary Table 2: List of rare CNVs in 416 OPGP control individuals

Supplementary Table 3: Summary of Affymetrix 6.0 microarray CNV data TOF sample (N=340)

Supplementary Table 4: Rare large CNVs (>500 kb) in 43 of 433 unrelated adults with tetralogy of Fallot

Supplementary Table 5: Very rare CNVs overlapping 26 candidate genes for tetralogy of Fallot

Supplementary Table 6: *PLXNA2* sequence variants detected in 192 unrelated TOF cases of European ancestry

Supplementary Table 7: Gene-set association results for all gene-sets tested, rare CNVs restricted to exonic losses

Supplementary Table 8: Additional gene-set information for the 19 gene-sets selected for final results

Supplementary Table 9: Known TOF disease genes used for the disease gene neighborhood analysis

Supplementary Table 10: Test results on disease gene neighborhoods for all disease genes, using the STRING network

Supplementary Table 11: Neighbor gene details for the three top disease genes

Supplementary Figure 1: Overview of study design and CNV analysis workflow

Supplementary Figure 2: Rare CNVs at chromosome region 1q21.1 in TOF cases

Supplementary Figure 3: Rare CNVs at chromosome region 18q22.3-q23 in TOF cases

Supplementary Figure 4: Integrated TOF pathway and candidate gene connectivity

Supplementary References

**SUPPLEMENTARY TABLES**

**Supplementary Table 1: List of rare CNVs in 340 TOF and/or pulmonary atresia cases of European ancestry**

**This list contains rare CNVs detected in 340 TOF cases of European ancestry that were not detected in 2,357 control individuals, using a 50% reciprocal overlap criteria. All CNVs are stringent (detected by 2 or more algorithms) and span at least 10 kb in length and 5 or more Affymetrix 6.0 array probes. Genes overlapped by these CNVs were used in pathway analyses versus those overlapped by rare CNVs in OPGP controls (Supplementary Table 2).**

| **Sample** | **Sex** | **Chr** | **Start** | **End** | **Size** | **CNV** | **genes** |
| --- | --- | --- | --- | --- | --- | --- | --- |
| 5 | M | 1 | 8,618,355 | 8,634,867 | 16,513 | loss | RERE |
| 115 | F | 1 | 26,248,164 | 26,275,407 | 27,244 | gain | TRIM63 |
| 1 | M | 1 | 35,138,921 | 35,758,858 | 619,938 | loss | KIAA0319L,ZMYM1,ZMYM6,ZMYM4,DLGAP3,SFPQ |
| 1 | M | 1 | 59,461,536 | 59,571,750 | 110,215 | gain | FGGY |
| 294 | F | 1 | 69,014,351 | 69,038,691 | 24,341 | loss | - |
| 293 | F | 1 | 73,954,108 | 74,215,749 | 261,642 | gain | - |
| 49 | M | 1 | 107,630,506 | 107,721,046 | 90,541 | loss | NTNG1 |
| 154 | M | 1 | 119,474,616 | 119,544,802 | 70,187 | loss | WARS2 |
| 2 | F | 1 | 143,590,972 | 147,572,692 | 3,981,720 | gain | ACP6, ANKRD34A, ANKRD35, BCL9, CD160, CHD1L, FLJ39739, FMO5, GJA5, GJA8, GNRHR2, GPR89A, GPR89B, GPR89C, HFE2, ITGA10, LIX1L, LOC100130000, LOC388692, LOC645166, LOC728989, NBPF10, NBPF11, NBPF14, NBPF15, NBPF16, NOTCH2NL, NUDT17, PDE4DIP, PDIA3P, PDZK1, PDZK1P1, PEX11B, PIAS3, POLR3C, POLR3GL, PPIAL4A, PPIAL4D, PPIAL4E, PPIAL4F, PRKAB2, RBM8A, RNF115, RP11-94I2.2, TXNIP |
| 3 | F | 1 | 144,643,825 | 146,273,898 | 1,630,074 | gain | ACP6,PRKAB2,CHD1L,FMO5,GJA8,PDZK1P1,PDIA3P,LOC728989,BCL9,NBPF11,GPR89B,GPR89C,RP11-94I2.2,GJA5 |
| 4 | F | 1 | 144,643,825 | 146,311,414 | 1,667,590 | gain | ACP6,PRKAB2,CHD1L,FMO5,GJA8,PDZK1P1,PDIA3P,LOC728989,BCL9,NBPF11,GPR89B,GPR89C,RP11-94I2.2,GJA5 |
| 5 | M | 1 | 144,643,825 | 146,395,960 | 1,752,136 | gain | ACP6,FLJ39739,PRKAB2,CHD1L,FMO5,GJA8,PDZK1P1,PDIA3P,LOC728989,BCL9,NBPF11,GPR89B,GPR89C,LOC100130000,RP11-94I2.2,GJA5 |
| 339 | M | 1 | 149,237,201 | 149,296,114 | 58,914 | gain | FAM63A,BNIPL,CDC42SE1,PRUNE,C1orf56 |
| 361 | M | 1 | 151,350,591 | 151,372,919 | 22,329 | gain | SPRR2F |
| 272 | F | 1 | 161,671,871 | 161,702,464 | 30,594 | loss | - |
| 317 | F | 1 | 195,202,028 | 195,222,522 | 20,495 | loss | CFHR5 |
| 42 | F | 1 | 206,337,728 | 206,399,273 | 61,546 | loss | PLXNA2 |
| 49 | M | 1 | 206,369,712 | 206,379,933 | 10,222 | loss | PLXNA2 |
| 61 | F | 1 | 211,026,844 | 211,047,750 | 20,907 | loss | NSL1,TATDN3 |
| 6 | F | 1 | 220,214,502 | 221,265,765 | 1,051,264 | loss | TAF1A,HHIPL2,AIDA,FAM177B,MIA3,DISP1,C1orf58 |
| 282 | F | 1 | 237,417,351 | 237,631,305 | 213,955 | gain | - |
| 51 | F | 1 | 238,098,745 | 238,109,312 | 10,568 | loss | CHRM3 |
| 290 | M | 1 | 239,261,298 | 239,281,896 | 20,599 | loss | RGS7 |
| 326 | F | 1 | 246,217,667 | 246,286,012 | 68,346 | loss | OR2L1P,OR2L13,OR2L2 |
| 143 | F | 2 | 2,335,000 | 2,361,350 | 26,351 | loss | - |
| 65 | M | 2 | 12,003,792 | 12,026,633 | 22,842 | loss | - |
| 322 | M | 2 | 19,732,647 | 19,749,388 | 16,742 | loss | - |
| 96 | F | 2 | 21,928,934 | 21,979,545 | 50,612 | gain | - |
| 324 | M | 2 | 23,291,285 | 23,311,333 | 20,049 | gain | - |
| 248 | F | 2 | 33,670,873 | 33,739,443 | 68,571 | gain | FAM98A |
| 308 | M | 2 | 34,671,199 | 34,764,631 | 93,433 | loss | - |
| 80 | M | 2 | 40,096,493 | 40,313,088 | 216,596 | gain | SLC8A1 |
| 199 | F | 2 | 41,145,088 | 41,157,119 | 12,032 | loss | - |
| 102 | M | 2 | 44,167,269 | 44,202,272 | 35,004 | loss | - |
| 244 | M | 2 | 45,165,866 | 45,287,568 | 121,703 | loss | - |
| 319 | F | 2 | 45,181,028 | 45,213,861 | 32,834 | loss | - |
| 187 | F | 2 | 46,522,826 | 46,543,946 | 21,121 | loss | - |
| 263 | M | 2 | 47,399,067 | 47,446,022 | 46,956 | loss | - |
| 208 | M | 2 | 48,603,914 | 48,632,568 | 28,655 | loss | - |
| 6 | F | 2 | 49,735,567 | 49,772,950 | 37,384 | loss | - |
| 105 | F | 2 | 49,792,518 | 49,816,482 | 23,965 | loss | - |
| 143 | F | 2 | 49,824,743 | 49,865,881 | 41,139 | loss | - |
| 86 | M | 2 | 53,232,993 | 53,289,795 | 56,803 | gain | - |
| 73 | F | 2 | 53,618,294 | 53,675,858 | 57,565 | loss | - |
| 274 | M | 2 | 54,339,645 | 54,386,600 | 46,956 | loss | ACYP2 |
| 267 | M | 2 | 56,247,095 | 56,257,578 | 10,484 | loss | - |
| 308 | M | 2 | 62,102,195 | 62,222,015 | 119,821 | gain | COMMD1 |
| 155 | M | 2 | 78,617,311 | 78,667,803 | 50,493 | loss | - |
| 270 | M | 2 | 96,431,165 | 96,465,561 | 34,397 | gain | - |
| 277 | M | 2 | 111,105,101 | 111,172,106 | 67,006 | loss | BUB1 |
| 9 | M | 2 | 131,194,418 | 132,021,415 | 826,998 | loss | FAM123C,FAM168B,TUBA3D,LOC150786,LOC150776,ARHGEF4,FAM128A,GPR148,PLEKHB2,LOC401010,LOC440910,POTEE,CCDC74A |
| 19 | F | 2 | 133,666,418 | 133,680,958 | 14,541 | loss | NCKAP5 |
| 357 | M | 2 | 133,745,635 | 133,832,274 | 86,640 | loss | NCKAP5 |
| 232 | M | 2 | 139,805,532 | 139,841,131 | 35,600 | gain | - |
| 233 | M | 2 | 153,378,432 | 153,505,030 | 126,599 | loss | - |
| 10 | M | 2 | 154,772,039 | 156,750,899 | 1,978,861 | gain | KCNJ3,GALNT13 |
| 294 | F | 2 | 155,711,563 | 155,755,859 | 44,297 | loss | - |
| 52 | M | 2 | 158,961,179 | 159,012,385 | 51,207 | loss | CCDC148 |
| 53 | F | 2 | 158,961,179 | 159,012,385 | 51,207 | loss | CCDC148 |
| 34 | F | 2 | 160,108,711 | 160,123,563 | 14,853 | gain | BAZ2B |
| 286 | M | 2 | 179,642,788 | 179,661,283 | 18,496 | loss | - |
| 147 | M | 2 | 184,690,605 | 184,856,669 | 166,065 | loss | - |
| 65 | M | 2 | 184,758,346 | 184,894,213 | 135,868 | gain | - |
| 201 | F | 2 | 185,466,657 | 185,483,402 | 16,746 | gain | ZNF804A |
| 11 | F | 2 | 188,273,007 | 188,973,871 | 700,865 | loss | GULP1,MIR561 |
| 255 | F | 2 | 189,806,236 | 189,829,210 | 22,975 | gain | - |
| 255 | F | 2 | 201,419,221 | 201,473,879 | 54,659 | loss | CLK1,PPIL3,NIF3L1 |
| 360 | M | 2 | 203,248,567 | 203,367,853 | 119,287 | gain | FAM117B,ICA1L |
| 54 | F | 2 | 215,266,200 | 215,351,866 | 85,667 | loss | BARD1 |
| 185 | F | 2 | 215,400,066 | 215,557,505 | 157,440 | loss | ABCA12 |
| 262 | M | 2 | 216,250,437 | 216,337,619 | 87,183 | gain | - |
| 117 | F | 2 | 221,304,488 | 221,426,532 | 122,045 | loss | - |
| 9 | M | 2 | 226,265,520 | 226,415,445 | 149,926 | loss | - |
| 52 | M | 2 | 228,241,899 | 228,310,862 | 68,964 | gain | SLC19A3 |
| 332 | M | 2 | 230,684,906 | 230,701,523 | 16,618 | loss | - |
| 65 | M | 2 | 232,916,978 | 232,950,147 | 33,170 | gain | - |
| 313 | M | 2 | 236,342,087 | 236,361,146 | 19,060 | loss | AGAP1 |
| 12 | M | 3 | 60,002 | 1,055,033 | 995,032 | gain | CHL1 |
| 19 | F | 3 | 1,146,111 | 1,195,072 | 48,962 | loss | CNTN6 |
| 54 | F | 3 | 5,425,470 | 5,453,372 | 27,903 | loss | - |
| 191 | M | 3 | 7,239,504 | 7,265,044 | 25,541 | gain | GRM7 |
| 289 | M | 3 | 10,620,143 | 10,635,402 | 15,260 | loss | - |
| 45 | F | 3 | 12,665,657 | 12,682,840 | 17,184 | loss | RAF1 |
| 68 | F | 3 | 22,535,176 | 22,552,351 | 17,176 | gain | - |
| 364 | M | 3 | 24,075,728 | 24,130,960 | 55,233 | loss | LOC152024 |
| 134 | F | 3 | 36,924,832 | 36,990,564 | 65,733 | loss | TRANK1 |
| 15 | F | 3 | 48,548,421 | 48,599,487 | 51,067 | gain | UCN2,MIR711,PFKFB4,COL7A1 |
| 336 | F | 3 | 57,506,361 | 57,552,531 | 46,171 | gain | PDE12,ARF4 |
| 285 | F | 3 | 60,496,636 | 60,512,436 | 15,801 | gain | FHIT |
| 294 | F | 3 | 61,034,471 | 61,141,405 | 106,935 | loss | FHIT |
| 134 | F | 3 | 95,941,073 | 96,102,686 | 161,614 | loss | - |
| 253 | M | 3 | 105,276,438 | 105,344,517 | 68,080 | gain | - |
| 252 | F | 3 | 106,782,935 | 106,838,619 | 55,685 | loss | - |
| 197 | F | 3 | 127,703,229 | 127,734,495 | 31,267 | loss | CHST13,UROC1 |
| 307 | M | 3 | 132,482,403 | 132,617,479 | 135,077 | loss | NUDT16P,NUDT16,NEK11 |
| 216 | F | 3 | 134,912,690 | 134,952,715 | 40,026 | loss | TF |
| 216 | F | 3 | 135,065,198 | 135,125,494 | 60,297 | loss | RAB6B |
| 216 | F | 3 | 135,291,948 | 135,313,443 | 21,496 | loss | - |
| 154 | M | 3 | 141,389,874 | 141,425,143 | 35,270 | loss | CLSTN2 |
| 323 | M | 3 | 143,702,232 | 143,939,520 | 237,289 | gain | PLS1,TRPC1,ATR |
| 62 | M | 3 | 158,304,447 | 158,331,331 | 26,885 | gain | - |
| 305 | F | 3 | 160,041,996 | 160,365,625 | 323,630 | gain | IQCJ |
| 324 | M | 3 | 164,473,558 | 164,687,222 | 213,665 | loss | - |
| 353 | M | 3 | 165,337,963 | 165,378,394 | 40,432 | gain | - |
| 324 | M | 3 | 169,802,805 | 169,874,339 | 71,535 | gain | C3orf50 |
| 20 | M | 3 | 174,218,460 | 174,292,092 | 73,633 | gain | SPATA16 |
| 34 | F | 3 | 174,622,952 | 174,646,448 | 23,497 | gain | NLGN1 |
| 155 | M | 3 | 178,888,612 | 178,904,969 | 16,358 | loss | - |
| 346 | F | 3 | 181,629,471 | 181,647,799 | 18,329 | gain | - |
| 45 | F | 3 | 181,775,881 | 181,804,251 | 28,371 | gain | TTC14 |
| 149 | M | 3 | 189,063,555 | 189,089,205 | 25,651 | loss | - |
| 175 | F | 3 | 189,685,244 | 189,748,992 | 63,749 | loss | LPP |
| 295 | F | 3 | 193,459,773 | 193,499,362 | 39,590 | loss | FGF12 |
| 27 | M | 3 | 194,195,280 | 194,222,582 | 27,303 | loss | - |
| 346 | F | 3 | 195,719,679 | 195,750,493 | 30,815 | gain | - |
| 337 | F | 3 | 195,794,530 | 195,841,919 | 47,390 | loss | TMEM44 |
| 84 | M | 3 | 198,672,424 | 198,697,483 | 25,060 | loss | - |
| 59 | M | 4 | 5,116,316 | 5,211,928 | 95,613 | loss | STK32B |
| 320 | M | 4 | 10,716,511 | 10,727,160 | 10,650 | loss | - |
| 295 | F | 4 | 11,904,955 | 11,938,010 | 33,056 | loss | - |
| 68 | F | 4 | 22,670,749 | 22,691,841 | 21,093 | loss | - |
| 110 | F | 4 | 28,348,262 | 28,474,772 | 126,511 | loss | - |
| 13 | F | 4 | 28,835,868 | 34,833,116 | 5,997,249 | loss | PCDH7 |
| 66 | F | 4 | 31,178,246 | 31,673,959 | 495,714 | gain | - |
| 264 | M | 4 | 31,236,234 | 31,246,767 | 10,534 | loss | - |
| 224 | F | 4 | 32,576,732 | 32,612,587 | 35,856 | gain | - |
| 345 | F | 4 | 34,070,871 | 34,121,052 | 50,182 | loss | - |
| 254 | F | 4 | 41,949,808 | 41,991,075 | 41,268 | loss | - |
| 188 | M | 4 | 46,896,575 | 47,171,633 | 275,059 | gain | GABRB1,COMMD8 |
| 337 | F | 4 | 63,653,595 | 63,813,833 | 160,239 | gain | - |
| 1 | M | 4 | 65,817,952 | 65,833,741 | 15,790 | loss | - |
| 102 | M | 4 | 66,473,030 | 66,503,730 | 30,701 | gain | - |
| 297 | F | 4 | 82,036,625 | 82,131,400 | 94,776 | gain | C4orf22 |
| 207 | F | 4 | 83,257,398 | 83,286,189 | 28,792 | gain | - |
| 90 | M | 4 | 84,716,133 | 84,882,453 | 166,321 | gain | AGPAT9 |
| 56 | M | 4 | 89,393,217 | 89,448,853 | 55,637 | gain | PPM1K |
| 129 | F | 4 | 102,554,107 | 102,637,762 | 83,656 | loss | - |
| 107 | M | 4 | 113,928,273 | 113,946,043 | 17,771 | loss | - |
| 222 | M | 4 | 118,207,450 | 118,238,309 | 30,860 | loss | TRAM1L1 |
| 209 | M | 4 | 132,448,474 | 132,468,268 | 19,795 | loss | - |
| 321 | M | 4 | 132,707,474 | 132,718,049 | 10,576 | loss | - |
| 38 | F | 4 | 133,183,878 | 133,320,544 | 136,667 | loss | - |
| 303 | F | 4 | 146,368,192 | 146,439,659 | 71,468 | gain | - |
| 157 | M | 4 | 148,985,752 | 149,019,917 | 34,166 | gain | ARHGAP10 |
| 186 | F | 4 | 157,693,557 | 157,775,935 | 82,379 | gain | - |
| 231 | M | 4 | 161,066,852 | 161,099,792 | 32,941 | loss | - |
| 250 | M | 4 | 162,440,151 | 162,462,824 | 22,674 | gain | - |
| 354 | F | 4 | 166,189,098 | 166,218,042 | 28,945 | loss | TMEM192 |
| 72 | F | 4 | 172,700,218 | 172,729,618 | 29,401 | loss | - |
| 3 | F | 4 | 173,971,165 | 174,009,934 | 38,770 | gain | GALNTL6 |
| 223 | M | 4 | 177,266,774 | 177,388,303 | 121,530 | gain | ASB5,SPATA4,WDR17,MIR1267 |
| 121 | M | 4 | 184,276,327 | 184,456,876 | 180,550 | gain | WWC2 |
| 121 | M | 4 | 186,130,980 | 186,285,321 | 154,342 | gain | HELT |
| 172 | F | 4 | 186,357,903 | 186,412,560 | 54,658 | loss | KIAA1430,SNX25 |
| 139 | F | 4 | 187,549,277 | 187,565,856 | 16,580 | loss | - |
| 14 | F | 4 | 188,923,641 | 189,437,846 | 514,206 | loss | ZFP42,TRIML2,TRIML1 |
| 11 | F | 4 | 189,814,140 | 189,829,654 | 15,515 | loss | - |
| 203 | F | 5 | 68,532 | 214,394 | 145,863 | loss | PLEKHG4B |
| 318 | F | 5 | 604,241 | 663,745 | 59,505 | gain | - |
| 126 | F | 5 | 17,733,842 | 17,743,876 | 10,035 | loss | - |
| 302 | M | 5 | 23,822,081 | 23,887,328 | 65,248 | loss | - |
| 176 | F | 5 | 24,349,044 | 24,417,939 | 68,896 | loss | - |
| 202 | F | 5 | 25,359,277 | 25,373,778 | 14,502 | loss | - |
| 253 | M | 5 | 28,561,853 | 28,614,542 | 52,690 | gain | - |
| 240 | M | 5 | 32,516,076 | 32,629,882 | 113,807 | gain | SUB1 |
| 109 | M | 5 | 40,499,620 | 40,593,642 | 94,023 | gain | - |
| 360 | M | 5 | 41,251,465 | 41,262,522 | 11,058 | loss | C6 |
| 73 | F | 5 | 44,314,920 | 44,450,565 | 135,646 | loss | FGF10 |
| 162 | M | 5 | 53,363,267 | 53,798,742 | 435,476 | gain | ARL15,HSPB3 |
| 364 | M | 5 | 57,178,618 | 57,191,065 | 12,448 | loss | - |
| 20 | M | 5 | 75,231,762 | 75,255,122 | 23,361 | gain | - |
| 288 | M | 5 | 75,647,353 | 75,687,542 | 40,190 | gain | SV2C |
| 123 | M | 5 | 76,655,690 | 76,708,716 | 53,027 | loss | PDE8B |
| 51 | F | 5 | 78,954,433 | 79,339,389 | 384,957 | gain | CMYA5,MTX3,PAPD4 |
| 10 | M | 5 | 80,665,064 | 86,119,881 | 5,454,818 | gain | SCARNA18,RPS23,NBPF22P,EDIL3,VCAN,XRCC4,ATG10,COX7C,TMEM167A,SSBP2,HAPLN1,ACOT12,ATP6AP1L |
| 166 | F | 5 | 92,590,720 | 92,622,201 | 31,482 | gain | - |
| 333 | M | 5 | 97,259,536 | 97,286,945 | 27,410 | loss | - |
| 314 | M | 5 | 101,136,583 | 101,151,437 | 14,855 | loss | - |
| 153 | F | 5 | 101,317,803 | 101,373,332 | 55,530 | loss | - |
| 205 | F | 5 | 101,857,277 | 101,927,153 | 69,877 | gain | SLCO6A1 |
| 200 | F | 5 | 104,145,578 | 104,240,839 | 95,262 | loss | - |
| 293 | F | 5 | 105,848,513 | 105,866,356 | 17,844 | loss | - |
| 256 | F | 5 | 109,507,901 | 109,535,616 | 27,716 | loss | - |
| 15 | F | 5 | 109,622,167 | 110,233,832 | 611,666 | gain | SLC25A46,TMEM232 |
| 192 | M | 5 | 109,737,426 | 109,760,381 | 22,956 | loss | - |
| 321 | M | 5 | 111,599,317 | 111,614,386 | 15,070 | loss | EPB41L4A |
| 84 | M | 5 | 112,984,499 | 113,016,769 | 32,271 | loss | - |
| 251 | F | 5 | 112,984,499 | 113,008,520 | 24,022 | loss | - |
| 104 | M | 5 | 113,220,627 | 113,275,285 | 54,659 | loss | - |
| 275 | M | 5 | 118,311,080 | 118,383,933 | 72,854 | loss | DTWD2 |
| 282 | F | 5 | 120,592,818 | 120,611,963 | 19,146 | loss | - |
| 252 | F | 5 | 120,747,216 | 120,773,806 | 26,591 | loss | - |
| 333 | M | 5 | 127,279,273 | 127,291,342 | 12,070 | gain | - |
| 16 | M | 5 | 130,124,159 | 130,935,316 | 811,158 | gain | CDC42SE2,RAPGEF6,LYRM7,HINT1 |
| 57 | F | 5 | 132,946,021 | 132,961,027 | 15,007 | loss | FSTL4 |
| 184 | F | 5 | 143,889,826 | 143,948,780 | 58,955 | gain | - |
| 180 | F | 5 | 154,189,099 | 154,211,107 | 22,009 | loss | C5orf4 |
| 228 | F | 5 | 161,645,179 | 161,656,839 | 11,661 | gain | - |
| 82 | F | 5 | 165,056,915 | 165,084,786 | 27,872 | loss | - |
| 290 | M | 5 | 171,402,350 | 171,422,574 | 20,225 | loss | STK10 |
| 43 | F | 5 | 180,213,360 | 180,307,129 | 93,770 | gain | BTNL8,ZFP62 |
| 56 | M | 6 | 1,938,284 | 1,951,032 | 12,749 | loss | GMDS |
| 59 | M | 6 | 1,939,891 | 1,951,032 | 11,142 | loss | GMDS |
| 308 | M | 6 | 2,255,036 | 2,287,918 | 32,883 | gain | - |
| 343 | F | 6 | 3,110,117 | 3,168,512 | 58,396 | gain | - |
| 156 | M | 6 | 8,940,232 | 8,964,482 | 24,251 | loss | - |
| 335 | M | 6 | 15,130,560 | 15,207,592 | 77,033 | loss | - |
| 97 | M | 6 | 18,158,780 | 18,172,542 | 13,763 | loss | - |
| 17 | M | 6 | 21,587,649 | 23,686,548 | 2,098,900 | gain | PRL,HDGFL1,FLJ22536,SOX4 |
| 245 | M | 6 | 22,416,212 | 22,462,185 | 45,974 | gain | - |
| 20 | M | 6 | 29,250,455 | 29,319,535 | 69,081 | loss | - |
| 317 | F | 6 | 33,939,650 | 33,993,575 | 53,926 | loss | - |
| 212 | F | 6 | 37,585,240 | 37,636,447 | 51,208 | gain | - |
| 259 | M | 6 | 43,738,893 | 43,838,460 | 99,568 | gain | RSPH9,MRPS18A |
| 68 | F | 6 | 57,416,954 | 57,526,882 | 109,929 | gain | PRIM2 |
| 18 | F | 6 | 61,987,979 | 62,979,533 | 991,555 | gain | KHDRBS2 |
| 243 | M | 6 | 63,475,529 | 63,508,008 | 32,480 | loss | - |
| 153 | F | 6 | 65,659,504 | 65,714,245 | 54,742 | loss | EYS |
| 298 | F | 6 | 66,490,170 | 66,532,321 | 42,152 | gain | - |
| 108 | M | 6 | 68,260,035 | 68,272,746 | 12,712 | gain | - |
| 105 | F | 6 | 76,887,261 | 76,904,717 | 17,457 | gain | - |
| 64 | M | 6 | 78,116,369 | 78,131,662 | 15,294 | loss | - |
| 1 | M | 6 | 78,610,954 | 78,650,416 | 39,463 | gain | - |
| 19 | F | 6 | 81,166,965 | 81,927,805 | 760,841 | gain | - |
| 91 | M | 6 | 89,477,988 | 89,509,074 | 31,087 | loss | RNGTT |
| 130 | F | 6 | 92,817,483 | 92,843,219 | 25,737 | gain | - |
| 244 | M | 6 | 93,682,700 | 93,951,784 | 269,085 | loss | - |
| 191 | M | 6 | 95,446,011 | 95,518,160 | 72,150 | gain | - |
| 2 | F | 6 | 96,084,834 | 96,197,844 | 113,011 | loss | MANEA |
| 218 | F | 6 | 102,515,325 | 102,526,335 | 11,011 | loss | GRIK2 |
| 87 | M | 6 | 111,387,116 | 111,459,392 | 72,277 | gain | RPF2,GTF3C6 |
| 361 | M | 6 | 121,619,149 | 121,645,686 | 26,538 | gain | C6orf170 |
| 245 | M | 6 | 122,004,610 | 122,017,262 | 12,653 | loss | - |
| 325 | M | 6 | 122,004,610 | 122,017,262 | 12,653 | loss | - |
| 331 | F | 6 | 128,124,840 | 128,319,456 | 194,617 | loss | THEMIS |
| 272 | F | 6 | 130,707,082 | 130,721,707 | 14,626 | loss | - |
| 254 | F | 6 | 152,916,343 | 152,970,784 | 54,442 | loss | SYNE1 |
| 231 | M | 6 | 170,172,246 | 170,267,728 | 95,483 | gain | - |
| 130 | F | 7 | 256,525 | 276,088 | 19,564 | gain | - |
| 74 | M | 7 | 2,310,838 | 2,490,905 | 180,068 | loss | EIF3B,SNX8,CHST12 |
| 197 | F | 7 | 4,214,839 | 4,275,511 | 60,673 | gain | SDK1 |
| 15 | F | 7 | 4,321,596 | 4,501,323 | 179,728 | gain | - |
| 183 | F | 7 | 6,517,414 | 6,545,584 | 28,171 | loss | GRID2IP |
| 143 | F | 7 | 8,445,467 | 8,697,869 | 252,403 | gain | NXPH1 |
| 37 | F | 7 | 10,083,089 | 10,119,055 | 35,967 | gain | - |
| 220 | F | 7 | 11,341,231 | 11,678,265 | 337,035 | loss | THSD7A |
| 12 | M | 7 | 14,024,970 | 14,072,405 | 47,436 | loss | - |
| 122 | F | 7 | 17,474,733 | 17,488,211 | 13,479 | gain | - |
| 315 | M | 7 | 17,723,106 | 17,740,782 | 17,677 | loss | - |
| 117 | F | 7 | 18,396,383 | 18,834,971 | 438,589 | loss | HDAC9 |
| 320 | M | 7 | 28,655,263 | 28,677,121 | 21,859 | loss | CREB5 |
| 73 | F | 7 | 29,482,735 | 29,637,243 | 154,509 | gain | CHN2,PRR15 |
| 73 | F | 7 | 29,752,455 | 29,873,443 | 120,989 | gain | WIPF3 |
| 164 | F | 7 | 40,368,636 | 40,410,865 | 42,230 | gain | C7orf10 |
| 175 | F | 7 | 79,395,959 | 79,466,877 | 70,919 | gain | - |
| 257 | F | 7 | 81,842,216 | 81,987,555 | 145,340 | gain | CACNA2D1 |
| 257 | F | 7 | 82,016,914 | 82,138,662 | 121,749 | gain | - |
| 18 | F | 7 | 84,915,683 | 84,942,617 | 26,935 | loss | - |
| 149 | M | 7 | 95,039,113 | 95,107,108 | 67,996 | loss | PDK4 |
| 155 | M | 7 | 107,440,401 | 107,569,826 | 129,426 | loss | LAMB4 |
| 155 | M | 7 | 108,006,004 | 108,066,786 | 60,783 | gain | - |
| 179 | M | 7 | 110,965,699 | 111,121,195 | 155,497 | gain | IMMP2L |
| 226 | F | 7 | 112,391,223 | 112,408,756 | 17,534 | loss | - |
| 36 | F | 7 | 114,366,668 | 114,434,310 | 67,643 | loss | MDFIC |
| 290 | M | 7 | 133,110,627 | 133,300,006 | 189,380 | loss | EXOC4 |
| 18 | F | 7 | 133,435,717 | 133,679,318 | 243,602 | gain | LRGUK,SLC35B4 |
| 62 | M | 7 | 141,562,226 | 141,632,941 | 70,716 | loss | LOC100124692,MOXD2,TRYX3 |
| 96 | F | 7 | 148,290,549 | 148,381,489 | 90,941 | loss | PDIA4 |
| 268 | M | 7 | 155,542,897 | 155,608,286 | 65,390 | gain | - |
| 118 | F | 7 | 156,999,245 | 157,060,389 | 61,145 | gain | PTPRN2,MIR153-2 |
| 250 | M | 8 | 530,949 | 580,379 | 49,431 | loss | - |
| 61 | F | 8 | 1,736,348 | 1,762,247 | 25,900 | loss | ARHGEF10,MIR596 |
| 232 | M | 8 | 4,201,469 | 4,231,318 | 29,850 | loss | CSMD1 |
| 166 | F | 8 | 4,631,280 | 4,643,554 | 12,275 | loss | CSMD1 |
| 69 | M | 8 | 5,863,468 | 5,886,256 | 22,789 | loss | - |
| 46 | M | 8 | 6,342,621 | 6,402,282 | 59,662 | loss | MCPH1,ANGPT2 |
| 362 | M | 8 | 10,487,567 | 10,543,617 | 56,051 | loss | RP1L1 |
| 73 | F | 8 | 18,194,281 | 18,216,291 | 22,011 | loss | - |
| 326 | F | 8 | 24,498,107 | 24,573,431 | 75,325 | gain | - |
| 23 | F | 8 | 47,203,026 | 48,101,824 | 898,799 | gain | BEYLA |
| 48 | F | 8 | 53,759,364 | 53,980,196 | 220,833 | gain | RB1CC1 |
| 344 | M | 8 | 54,486,527 | 54,498,726 | 12,200 | loss | - |
| 107 | M | 8 | 63,490,749 | 63,515,695 | 24,947 | loss | NKAIN3 |
| 240 | M | 8 | 68,188,369 | 68,292,321 | 103,953 | gain | CSPP1,ARFGEF1 |
| 62 | M | 8 | 73,871,523 | 73,950,002 | 78,480 | gain | KCNB2 |
| 63 | M | 8 | 73,885,905 | 74,010,693 | 124,789 | gain | KCNB2 |
| 83 | M | 8 | 79,414,446 | 79,454,136 | 39,691 | loss | - |
| 210 | M | 8 | 80,041,772 | 80,328,846 | 287,075 | loss | - |
| 331 | F | 8 | 85,287,618 | 85,356,902 | 69,285 | loss | RALYL |
| 112 | F | 8 | 88,446,953 | 88,502,268 | 55,316 | loss | CNBD1 |
| 263 | M | 8 | 98,572,588 | 98,635,791 | 63,204 | gain | - |
| 3 | F | 8 | 103,288,416 | 103,387,603 | 99,188 | gain | RRM2B,UBR5 |
| 41 | M | 8 | 107,603,307 | 107,615,665 | 12,359 | gain | OXR1 |
| 364 | M | 8 | 107,932,392 | 107,961,037 | 28,646 | gain | - |
| 275 | M | 8 | 117,640,815 | 117,877,221 | 236,407 | gain | EIF3H,UTP23 |
| 164 | F | 8 | 122,004,525 | 122,077,124 | 72,600 | gain | - |
| 191 | M | 8 | 125,485,483 | 125,538,690 | 53,208 | gain | TRMT12 |
| 88 | F | 8 | 130,452,195 | 130,536,854 | 84,660 | loss | - |
| 25 | F | 8 | 130,753,073 | 130,797,507 | 44,435 | loss | - |
| 164 | F | 8 | 134,908,413 | 134,934,057 | 25,645 | loss | - |
| 15 | F | 8 | 142,627,566 | 142,981,041 | 353,476 | gain | - |
| 129 | F | 9 | 1,267,906 | 1,289,893 | 21,988 | gain | - |
| 168 | M | 9 | 3,050,199 | 3,089,009 | 38,811 | gain | - |
| 241 | M | 9 | 4,745,566 | 4,775,307 | 29,742 | loss | - |
| 14 | F | 9 | 6,318,254 | 6,392,856 | 74,603 | gain | TPD52L3 |
| 166 | F | 9 | 9,671,202 | 9,706,152 | 34,951 | loss | PTPRD |
| 162 | M | 9 | 10,191,885 | 10,237,230 | 45,346 | loss | PTPRD |
| 206 | M | 9 | 10,429,312 | 10,492,650 | 63,339 | loss | PTPRD |
| 72 | F | 9 | 11,771,428 | 11,907,137 | 135,710 | gain | - |
| 186 | F | 9 | 12,571,785 | 12,617,540 | 45,756 | gain | - |
| 199 | F | 9 | 13,222,399 | 13,243,913 | 21,515 | gain | MPDZ |
| 352 | M | 9 | 13,693,903 | 13,826,332 | 132,430 | loss | - |
| 14 | F | 9 | 16,318,329 | 16,329,742 | 11,414 | loss | - |
| 243 | M | 9 | 16,938,400 | 16,953,863 | 15,464 | loss | - |
| 328 | M | 9 | 25,534,793 | 25,713,691 | 178,899 | loss | TUSC1 |
| 147 | M | 9 | 28,387,397 | 28,582,527 | 195,131 | loss | LINGO2 |
| 14 | F | 9 | 36,071,389 | 36,101,003 | 29,615 | loss | RECK |
| 163 | M | 9 | 70,231,698 | 70,250,517 | 18,820 | gain | PGM5 |
| 125 | F | 9 | 73,792,809 | 73,931,235 | 138,427 | loss | C9orf57 |
| 256 | F | 9 | 86,428,967 | 86,464,568 | 35,602 | gain | - |
| 176 | F | 9 | 118,727,651 | 118,737,800 | 10,150 | loss | ASTN2 |
| 313 | M | 9 | 136,861,991 | 137,157,017 | 295,027 | gain | FCN2,FCN1,COL5A1,OLFM1 |
| 324 | M | 9 | 138,229,500 | 138,275,049 | 45,550 | gain | QSOX2,LHX3 |
| 304 | M | 10 | 1,357,514 | 1,368,827 | 11,314 | loss | ADARB2 |
| 27 | M | 10 | 12,540,181 | 12,578,296 | 38,116 | loss | CAMK1D |
| 278 | F | 10 | 19,723,673 | 19,831,233 | 107,561 | gain | - |
| 51 | F | 10 | 21,663,591 | 21,674,579 | 10,989 | loss | - |
| 320 | M | 10 | 30,673,942 | 31,063,750 | 389,809 | gain | LOC729668,MAP3K8,MTPAP,LYZL2 |
| 88 | F | 10 | 32,726,549 | 32,744,471 | 17,923 | loss | - |
| 100 | M | 10 | 54,067,002 | 54,110,702 | 43,701 | gain | - |
| 331 | F | 10 | 56,047,904 | 56,069,130 | 21,227 | loss | PCDH15 |
| 177 | M | 10 | 67,206,570 | 67,333,418 | 126,849 | loss | - |
| 323 | M | 10 | 75,031,516 | 75,099,170 | 67,655 | gain | SYNPO2L,MYOZ1 |
| 48 | F | 10 | 88,477,734 | 88,503,877 | 26,144 | gain | LDB3 |
| 59 | M | 10 | 96,431,290 | 96,623,002 | 191,713 | loss | CYP2C19,CYP2C18 |
| 248 | F | 10 | 116,791,047 | 116,839,724 | 48,678 | loss | - |
| 290 | M | 10 | 124,067,587 | 124,097,474 | 29,888 | loss | BTBD16 |
| 175 | F | 10 | 127,768,493 | 127,792,739 | 24,247 | gain | ADAM12 |
| 181 | M | 10 | 128,766,827 | 128,913,442 | 146,616 | loss | FAM196A,DOCK1 |
| 218 | F | 11 | 5,019,412 | 5,030,062 | 10,651 | loss | OR52J3 |
| 339 | M | 11 | 6,862,381 | 6,926,252 | 63,872 | gain | ZNF215,OR2D2,OR2D3 |
| 123 | M | 11 | 16,472,010 | 16,498,199 | 26,190 | loss | - |
| 85 | F | 11 | 24,657,175 | 24,911,798 | 254,624 | loss | LUZP2 |
| 201 | F | 11 | 25,680,459 | 25,690,870 | 10,412 | loss | - |
| 322 | M | 11 | 31,504,508 | 31,571,895 | 67,388 | loss | ELP4 |
| 324 | M | 11 | 35,534,630 | 35,545,484 | 10,855 | loss | - |
| 330 | M | 11 | 41,991,508 | 42,022,363 | 30,856 | loss | - |
| 18 | F | 11 | 78,852,013 | 78,891,406 | 39,394 | gain | - |
| 210 | M | 11 | 81,950,592 | 81,999,742 | 49,151 | gain | - |
| 332 | M | 11 | 83,927,065 | 84,049,246 | 122,182 | loss | DLG2 |
| 104 | M | 11 | 85,319,279 | 85,359,564 | 40,286 | gain | PICALM |
| 193 | M | 11 | 90,446,944 | 90,477,781 | 30,838 | gain | - |
| 279 | M | 11 | 94,399,142 | 94,422,368 | 23,227 | loss | KDM4DL |
| 309 | M | 11 | 96,105,151 | 96,232,212 | 127,062 | gain | - |
| 264 | M | 11 | 99,498,851 | 99,510,915 | 12,065 | loss | CNTN5 |
| 69 | M | 11 | 104,212,897 | 104,248,128 | 35,232 | loss | - |
| 91 | M | 11 | 111,570,348 | 111,584,200 | 13,853 | loss | BCO2 |
| 126 | F | 11 | 115,004,272 | 115,019,412 | 15,141 | loss | - |
| 104 | M | 11 | 125,399,550 | 125,411,963 | 12,414 | gain | CDON |
| 32 | M | 12 | 9,387,160 | 9,438,707 | 51,548 | gain | - |
| 256 | F | 12 | 30,839,643 | 31,160,565 | 320,923 | gain | TSPAN11,DDX11 |
| 133 | F | 12 | 39,785,500 | 39,866,592 | 81,093 | loss | - |
| 254 | F | 12 | 44,228,538 | 44,431,767 | 203,230 | gain | ARID2,LOC400027 |
| 191 | M | 12 | 46,989,386 | 47,019,351 | 29,966 | loss | H1FNT |
| 20 | M | 12 | 62,843,704 | 62,921,992 | 78,289 | gain | C12orf66 |
| 64 | M | 12 | 62,843,704 | 62,921,992 | 78,289 | gain | C12orf66 |
| 52 | M | 12 | 73,393,644 | 73,435,924 | 42,281 | loss | - |
| 17 | M | 12 | 81,932,739 | 81,943,799 | 11,061 | gain | TMTC2 |
| 151 | F | 12 | 105,788,757 | 105,890,822 | 102,066 | gain | RIC8B,C12orf23 |
| 337 | F | 12 | 121,166,750 | 121,231,275 | 64,526 | gain | LRRC43,MLXIP,IL31 |
| 321 | M | 12 | 121,649,761 | 121,983,699 | 333,939 | gain | CCDC62,DENR,KNTC1,VPS37B,GPR109B,GPR81,ABCB9,GPR109A,HIP1R |
| 29 | F | 12 | 125,358,126 | 126,141,645 | 783,520 | loss | LOC100128554,LOC387895,LOC440117 |
| 200 | F | 12 | 127,439,315 | 127,553,400 | 114,086 | loss | TMEM132C |
| 285 | F | 12 | 128,298,310 | 128,316,477 | 18,168 | loss | TMEM132D |
| 213 | F | 12 | 128,919,047 | 129,361,281 | 442,235 | gain | TMEM132D,FZD10,LOC100190940 |
| 342 | M | 12 | 129,642,826 | 129,696,242 | 53,417 | loss | - |
| 247 | M | 12 | 129,734,810 | 129,758,793 | 23,984 | loss | - |
| 16 | M | 12 | 130,747,265 | 130,783,723 | 36,459 | gain | SFRS8 |
| 45 | F | 13 | 22,320,753 | 22,381,330 | 60,578 | loss | LOC646201 |
| 47 | F | 13 | 34,960,143 | 34,980,762 | 20,620 | loss | NBEA,MIR548F5 |
| 253 | M | 13 | 51,808,690 | 52,028,474 | 219,785 | gain | CKAP2,LOC220115,VPS36,THSD1 |
| 268 | M | 13 | 54,041,427 | 54,374,043 | 332,617 | loss | - |
| 293 | F | 13 | 54,766,214 | 54,873,777 | 107,564 | loss | - |
| 1 | M | 13 | 55,231,739 | 55,281,721 | 49,983 | loss | - |
| 354 | F | 13 | 56,203,414 | 56,272,387 | 68,974 | gain | - |
| 110 | F | 13 | 82,476,609 | 82,496,719 | 20,111 | gain | - |
| 243 | M | 13 | 93,025,804 | 93,202,536 | 176,733 | loss | GPC6 |
| 250 | M | 13 | 93,522,342 | 93,654,654 | 132,313 | loss | GPC6 |
| 318 | F | 13 | 102,773,060 | 103,030,245 | 257,186 | loss | - |
| 349 | F | 13 | 102,826,334 | 102,839,546 | 13,213 | loss | - |
| 32 | M | 13 | 103,096,790 | 103,922,204 | 825,415 | gain | - |
| 342 | M | 13 | 104,223,256 | 104,245,919 | 22,664 | loss | - |
| 36 | F | 13 | 105,503,246 | 105,516,128 | 12,883 | loss | - |
| 217 | M | 13 | 107,161,950 | 107,211,315 | 49,366 | gain | FAM155A |
| 233 | M | 13 | 107,370,833 | 107,564,097 | 193,265 | gain | - |
| 234 | M | 13 | 108,674,639 | 109,026,309 | 351,671 | gain | - |
| 73 | F | 14 | 21,999,173 | 22,035,029 | 35,857 | gain | - |
| 296 | M | 14 | 23,355,915 | 23,377,938 | 22,024 | loss | - |
| 92 | M | 14 | 34,599,292 | 34,793,990 | 194,699 | gain | PPP2R3C,FAM177A1,KIAA0391 |
| 64 | M | 14 | 36,476,535 | 36,592,567 | 116,033 | loss | SLC25A21 |
| 118 | F | 14 | 39,201,887 | 39,347,144 | 145,258 | loss | - |
| 208 | M | 14 | 40,768,616 | 41,158,804 | 390,189 | gain | LRFN5 |
| 19 | F | 14 | 40,940,472 | 40,965,884 | 25,413 | loss | - |
| 18 | F | 14 | 41,552,564 | 41,637,840 | 85,277 | loss | - |
| 100 | M | 14 | 41,768,037 | 42,134,475 | 366,439 | loss | - |
| 9 | M | 14 | 42,406,009 | 42,449,623 | 43,615 | gain | - |
| 273 | M | 14 | 43,044,905 | 43,064,828 | 19,924 | loss | - |
| 226 | F | 14 | 50,143,907 | 50,240,359 | 96,453 | loss | ATL1,SAV1 |
| 2 | F | 14 | 63,839,710 | 63,914,251 | 74,542 | gain | ESR2 |
| 199 | F | 14 | 64,804,949 | 64,859,280 | 54,332 | gain | - |
| 203 | F | 14 | 72,088,444 | 72,257,827 | 169,384 | gain | DPF3,RGS6 |
| 275 | M | 14 | 78,554,233 | 78,640,303 | 86,071 | loss | NRXN3 |
| 136 | F | 14 | 95,329,892 | 95,347,094 | 17,203 | loss | - |
| 297 | F | 14 | 95,331,527 | 95,344,355 | 12,829 | loss | - |
| 134 | F | 15 | 21,790,968 | 21,806,337 | 15,370 | loss | - |
| 212 | F | 15 | 36,983,956 | 37,174,792 | 190,837 | gain | - |
| 198 | F | 15 | 48,677,625 | 48,833,587 | 155,963 | gain | SPPL2A,TRPM7 |
| 280 | M | 15 | 52,118,765 | 52,163,314 | 44,550 | loss | UNC13C |
| 189 | M | 15 | 53,345,483 | 53,492,877 | 147,395 | gain | RAB27A,PIGB,CCPG1,MIR628 |
| 189 | M | 15 | 53,590,225 | 53,731,809 | 141,585 | gain | PYGO1,PRTG |
| 264 | M | 15 | 56,279,346 | 56,298,395 | 19,050 | loss | - |
| 304 | M | 15 | 59,279,170 | 59,349,472 | 70,303 | loss | RORA |
| 136 | F | 15 | 59,731,042 | 59,767,238 | 36,197 | loss | - |
| 304 | M | 15 | 66,064,468 | 66,082,397 | 17,930 | loss | - |
| 14 | F | 15 | 68,554,399 | 68,571,479 | 17,081 | loss | - |
| 62 | M | 15 | 69,022,959 | 69,224,764 | 201,806 | gain | LRRC49,THSD4,CT62 |
| 73 | F | 15 | 93,084,371 | 93,094,516 | 10,146 | loss | - |
| 319 | F | 15 | 94,818,741 | 94,840,851 | 22,111 | gain | - |
| 215 | F | 16 | 1,926,405 | 2,086,460 | 160,056 | gain | GFER,PKD1,NPW,SEPX1,SLC9A3R2,NOXO1,SNORA10,NDUFB10,RPL3L,RPS2,TSC2,SNORA78,SNORA64,MIR1225,ZNF598,RNF151,SNHG9,SYNGR3,NTHL1,TBL3 |
| 159 | F | 16 | 3,524,500 | 3,590,517 | 66,018 | loss | CLUAP1,BTBD12,NLRC3 |
| 187 | F | 16 | 4,062,423 | 4,155,400 | 92,978 | gain | ADCY9 |
| 311 | M | 16 | 4,972,089 | 4,985,460 | 13,372 | loss | SEC14L5 |
| 231 | M | 16 | 5,680,531 | 5,734,330 | 53,800 | loss | - |
| 225 | M | 16 | 6,764,494 | 6,796,588 | 32,095 | gain | A2BP1 |
| 222 | M | 16 | 8,358,150 | 8,474,829 | 116,680 | loss | - |
| 318 | F | 16 | 11,969,127 | 12,015,534 | 46,408 | loss | RUNDC2A,TNFRSF17 |
| 309 | M | 16 | 20,753,084 | 20,774,403 | 21,320 | gain | LOC81691 |
| 11 | F | 16 | 22,558,149 | 22,581,785 | 23,637 | gain | - |
| 235 | F | 16 | 23,368,660 | 23,383,757 | 15,098 | gain | GGA2,COG7 |
| 192 | M | 16 | 27,384,191 | 27,420,058 | 35,868 | loss | GTF3C1 |
| 33 | M | 16 | 29,487,535 | 30,085,308 | 597,774 | gain | DOC2A,ASPHD1,LOC440356,TBX6,LOC100271831,CDIPT,QPRT,YPEL3,SLC7A5P1,PPP4C,MAPK3,SPN,MVP,FAM57B,ALDOA,INO80E,SEZ6L2,TAOK2,KCTD13,MAZ,PRRT2,GDPD3,C16orf92,C16orf53,TMEM219,C16orf54,HIRIP3 |
| 119 | M | 16 | 57,535,186 | 57,600,129 | 64,944 | loss | - |
| 264 | M | 16 | 58,005,768 | 58,240,139 | 234,372 | gain | - |
| 233 | M | 16 | 64,617,243 | 64,628,386 | 11,144 | loss | - |
| 4 | F | 16 | 69,408,394 | 69,763,409 | 355,016 | gain | HYDIN |
| 5 | M | 16 | 69,662,837 | 69,759,990 | 97,154 | gain | HYDIN |
| 338 | M | 16 | 69,976,731 | 70,021,647 | 44,917 | gain | CALB2 |
| 269 | M | 16 | 69,976,731 | 70,012,760 | 36,030 | gain | CALB2 |
| 198 | F | 16 | 70,949,561 | 70,966,457 | 16,897 | loss | - |
| 279 | M | 16 | 71,903,428 | 71,914,449 | 11,022 | loss | - |
| 205 | F | 16 | 73,427,138 | 73,452,560 | 25,423 | loss | - |
| 220 | F | 16 | 73,795,604 | 73,852,090 | 56,487 | loss | CTRB2,CTRB1,BCAR1 |
| 220 | F | 16 | 81,545,373 | 81,604,208 | 58,836 | loss | CDH13 |
| 345 | F | 16 | 81,545,526 | 81,613,645 | 68,120 | loss | CDH13 |
| 253 | M | 16 | 82,902,898 | 82,979,491 | 76,594 | gain | WFDC1,ATP2C2 |
| 65 | M | 16 | 83,066,869 | 83,082,921 | 16,053 | loss | KIAA1609 |
| 66 | F | 16 | 83,094,514 | 83,120,895 | 26,382 | loss | KIAA1609 |
| 234 | M | 16 | 83,104,423 | 83,120,895 | 16,473 | loss | - |
| 316 | F | 16 | 88,190,573 | 88,287,642 | 97,070 | gain | CHMP1A,CPNE7,DPEP1,CDK10,C16orf55 |
| 35 | M | 17 | 5,528,898 | 6,196,701 | 667,804 | loss | WSCD1 |
| 238 | M | 17 | 45,333,985 | 45,485,936 | 151,952 | loss | DLX4,DLX3 |
| 241 | M | 17 | 53,168,547 | 53,221,329 | 52,783 | loss | - |
| 301 | F | 17 | 66,013,671 | 66,078,688 | 65,018 | gain | - |
| 197 | F | 17 | 66,337,220 | 66,365,604 | 28,385 | loss | - |
| 238 | M | 18 | 3,597,746 | 3,628,405 | 30,660 | gain | DLGAP1 |
| 96 | F | 18 | 4,448,600 | 4,484,321 | 35,722 | loss | - |
| 177 | M | 18 | 6,087,226 | 6,104,689 | 17,464 | loss | L3MBTL4 |
| 273 | M | 18 | 10,456,001 | 10,586,012 | 130,012 | gain | NAPG,APCDD1 |
| 279 | M | 18 | 11,303,172 | 11,315,448 | 12,277 | loss | - |
| 49 | M | 18 | 13,160,818 | 13,206,248 | 45,431 | gain | - |
| 218 | F | 18 | 21,728,345 | 21,858,584 | 130,240 | gain | SS18 |
| 136 | F | 18 | 25,645,166 | 25,723,728 | 78,563 | gain | - |
| 357 | M | 18 | 25,645,166 | 25,722,301 | 77,136 | gain | - |
| 20 | M | 18 | 26,327,258 | 26,342,812 | 15,555 | loss | - |
| 317 | F | 18 | 33,831,057 | 33,849,871 | 18,815 | loss | - |
| 5 | M | 18 | 36,443,745 | 36,459,723 | 15,979 | gain | - |
| 293 | F | 18 | 44,049,984 | 44,106,187 | 56,204 | loss | - |
| 179 | M | 18 | 48,058,766 | 48,141,966 | 83,201 | gain | DCC |
| 258 | M | 18 | 57,190,448 | 57,207,556 | 17,109 | loss | - |
| 48 | F | 18 | 62,118,826 | 62,372,858 | 254,033 | loss | CDH19 |
| 192 | M | 18 | 67,524,401 | 67,545,932 | 21,532 | loss | - |
| 260 | F | 18 | 68,188,174 | 68,278,075 | 89,902 | loss | - |
| 160 | M | 18 | 69,096,198 | 69,111,092 | 14,895 | loss | - |
| 37 | F | 18 | 69,207,652 | 72,613,722 | 3,406,071 | gain | C18orf55,TSHZ1,CYB5A,ZADH2,FBXO15,FAM69C,LOC400657,LOC284276,ZNF407,ZNF516,CNDP1,CNDP2,C18orf62 |
| 37 | F | 18 | 72,614,319 | 76,116,029 | 3,501,711 | loss | NFATC1,C18orf22,PARD6G,ZNF236,KCNG2,CTDP1,HSBP1L1,GALR1,ADNP2,ATP9B,SALL3,TXNL4A,MBP,LOC100130522,PQLC1 |
| 38 | F | 19 | 183,496 | 989,222 | 805,727 | loss | ELANE,C2CD4C,PPAP2C,THEG,C19orf6,PRSSL1,C19orf22,C19orf20,C19orf21,LPPR3,MIR3187,CDC34,FSTL3,GRIN3B,ODF3L2,MED16,MADCAM1,PTBP1,BSG,PRTN3,KISS1R,SHC2,MIER2,PALM,ARID3A,CNN2,GZMM,CFD,HCN2,AZU1,POLRMT,RNF126,WDR18,FGF22 |
| 141 | M | 19 | 2,783,567 | 2,914,355 | 130,789 | loss | ZNF556,ZNF554,ZNF555,ZNF77,ZNF57 |
| 36 | F | 19 | 11,822,192 | 11,850,836 | 28,645 | loss | ZNF439 |
| 104 | M | 19 | 15,070,102 | 15,222,826 | 152,725 | gain | ILVBL,EPHX3,BRD4,NOTCH3,SYDE1 |
| 42 | F | 19 | 22,764,976 | 22,802,759 | 37,784 | loss | - |
| 203 | F | 19 | 23,229,046 | 23,294,484 | 65,439 | gain | - |
| 259 | M | 19 | 23,423,866 | 23,505,118 | 81,253 | gain | - |
| 86 | M | 19 | 23,663,378 | 23,740,088 | 76,711 | loss | RPSAP58,ZNF681 |
| 359 | F | 19 | 33,240,400 | 33,356,711 | 116,312 | gain | - |
| 128 | M | 19 | 34,456,548 | 34,517,054 | 60,507 | gain | - |
| 68 | F | 19 | 38,902,591 | 38,926,409 | 23,819 | gain | CHST8 |
| 67 | F | 19 | 38,902,591 | 38,929,183 | 26,593 | gain | CHST8 |
| 306 | F | 19 | 42,377,030 | 42,447,571 | 70,542 | gain | ZNF585B,ZNF383 |
| 120 | M | 19 | 48,882,502 | 48,901,641 | 19,140 | loss | - |
| 141 | M | 19 | 51,560,658 | 51,638,513 | 77,856 | loss | PPP5C,CCDC8 |
| 29 | F | 19 | 53,722,092 | 53,758,471 | 36,380 | loss | SULT2B1 |
| 333 | M | 19 | 62,297,265 | 62,385,750 | 88,486 | loss | DUXA,USP29,ZIM3 |
| 101 | F | 20 | 4,370,540 | 4,812,570 | 442,031 | gain | PRND,PRNT,RASSF2,PRNP,SLC23A2 |
| 15 | F | 20 | 5,895,608 | 5,923,190 | 27,583 | loss | MCM8 |
| 110 | F | 20 | 15,951,406 | 16,027,006 | 75,601 | gain | MACROD2 |
| 165 | F | 20 | 19,730,288 | 19,824,487 | 94,200 | loss | RIN2 |
| 232 | M | 20 | 30,701,745 | 30,726,581 | 24,837 | gain | C20orf203 |
| 302 | M | 20 | 36,149,236 | 36,288,533 | 139,298 | gain | TGM2,RPRD1B,KIAA1755 |
| 321 | M | 20 | 37,725,402 | 37,735,731 | 10,330 | gain | - |
| 210 | M | 20 | 39,196,466 | 39,266,312 | 69,847 | gain | ZHX3,PLCG1 |
| 148 | M | 20 | 47,559,427 | 47,603,521 | 44,095 | loss | PTGIS |
| 46 | M | 21 | 15,357,535 | 15,403,559 | 46,025 | gain | NRIP1 |
| 202 | F | 21 | 16,241,417 | 16,324,225 | 82,809 | gain | - |
| 41 | M | 21 | 18,206,970 | 18,533,457 | 326,488 | gain | - |
| 41 | M | 21 | 20,282,405 | 20,842,781 | 560,377 | gain | - |
| 334 | M | 21 | 20,839,759 | 21,280,034 | 440,276 | loss | C21orf131 |
| 132 | F | 21 | 27,335,028 | 27,624,322 | 289,295 | loss | - |
| 42 | F | 21 | 29,607,482 | 30,244,784 | 637,303 | loss | GRIK1,BACH1,NCRNA00110 |
| 313 | M | 21 | 41,834,843 | 41,865,347 | 30,505 | loss | - |
| 208 | M | 21 | 43,477,781 | 43,488,654 | 10,874 | loss | - |
| 231 | M | 21 | 45,574,311 | 45,711,138 | 136,828 | gain | COL18A1,NCRNA00175 |
| 51 | F | 21 | 46,033,892 | 46,151,676 | 117,785 | gain | PCBP3 |
| 69 | M | 22 | 15,996,510 | 16,015,489 | 18,980 | loss | CECR5 |
| 47 | F | 22 | 16,014,050 | 16,098,911 | 84,862 | loss | CECR1,CECR4,CECR5 |
| 202 | F | 22 | 19,226,383 | 19,314,033 | 87,651 | gain | MED15 |
| 315 | M | 22 | 32,251,753 | 32,264,464 | 12,712 | gain | LARGE |
| 340 | M | 22 | 37,302,679 | 37,315,764 | 13,086 | loss | LOC646851 |
| 6 | F | 22 | 39,648,856 | 40,006,122 | 357,267 | gain | RANGAP1,MIR1281,L3MBTL2,CHADL,RBX1,EP300,XPNPEP3 |
| 203 | F | 22 | 45,624,603 | 45,655,934 | 31,332 | loss | TBC1D22A |
| 87 | M | 22 | 47,653,943 | 47,716,727 | 62,785 | gain | - |
| 218 | F | X | 534,745 | 772,639 | 237,895 | loss | SHOX |
| 23 | F | X | 3,964,972 | 3,985,056 | 20,085 | loss | - |
| 243 | M | X | 10,146,592 | 10,335,804 | 189,213 | gain | CLCN4 |
| 43 | F | X | 13,788,639 | 14,404,578 | 615,940 | gain | GEMIN8,GPM6B |
| 321 | M | X | 17,421,968 | 17,651,407 | 229,440 | gain | NHS |
| 244 | M | X | 30,780,153 | 31,165,421 | 385,269 | gain | TAB3,DMD,FTHL17 |
| 260 | F | X | 32,681,897 | 32,719,084 | 37,188 | gain | DMD |
| 220 | F | X | 37,218,812 | 37,264,580 | 45,769 | gain | - |
| 2 | F | X | 38,626,839 | 38,640,875 | 14,037 | loss | - |
| 319 | F | X | 40,631,448 | 40,648,111 | 16,664 | gain | - |
| 306 | F | X | 41,954,001 | 42,179,525 | 225,525 | gain | - |
| 342 | M | X | 42,746,793 | 43,103,063 | 356,271 | loss | - |
| 264 | M | X | 50,666,932 | 50,879,569 | 212,638 | gain | BMP15 |
| 70 | M | X | 54,325,120 | 54,472,795 | 147,676 | gain | WNK3 |
| 32 | M | X | 54,374,737 | 54,424,898 | 50,162 | gain | WNK3 |
| 169 | F | X | 63,688,844 | 63,824,139 | 135,296 | gain | - |
| 205 | F | X | 82,656,149 | 82,722,076 | 65,928 | gain | - |
| 169 | F | X | 87,303,873 | 87,319,571 | 15,699 | gain | - |
| 83 | M | X | 88,304,464 | 88,342,466 | 38,003 | loss | - |
| 74 | M | X | 93,542,246 | 93,571,032 | 28,787 | gain | - |
| 270 | M | X | 97,648,123 | 97,665,827 | 17,705 | loss | - |
| 348 | M | X | 104,431,315 | 104,507,544 | 76,230 | loss | IL1RAPL2 |
| 363 | M | X | 107,406,181 | 107,447,215 | 41,035 | gain | COL4A6 |
| 91 | M | X | 109,281,155 | 109,449,488 | 168,334 | gain | SNORD96B,TMEM164,AMMECR1 |
| 37 | F | X | 113,157,949 | 113,277,086 | 119,138 | gain | - |
| 319 | F | X | 116,661,071 | 116,672,834 | 11,764 | gain | - |
| 357 | M | X | 117,754,919 | 117,837,240 | 82,322 | gain | IL13RA1 |
| 295 | F | X | 125,025,764 | 125,134,215 | 108,452 | loss | DCAF12L2 |
| 23 | F | X | 125,276,972 | 125,314,710 | 37,739 | gain | - |
| 149 | M | X | 126,190,364 | 126,264,629 | 74,266 | gain | - |
| 207 | F | X | 127,630,603 | 127,644,960 | 14,358 | gain | - |
| 202 | F | X | 141,470,429 | 141,830,275 | 359,847 | gain | - |
| 53 | F | X | 143,324,556 | 143,408,399 | 83,844 | loss | - |
| 363 | M | X | 145,842,979 | 146,062,239 | 219,261 | loss | - |
| 18 | F | X | 147,491,928 | 147,514,998 | 23,071 | loss | AFF2 |
| 71 | M | X | 148,308,784 | 148,462,405 | 153,622 | gain | LOC100131434,IDS,CXorf40A |
| 72 | F | X | 148,377,523 | 148,628,359 | 250,837 | gain | MAGEA11,LOC100131434,IDS,HSFX2,CXorf40A,MAGEA9,HSFX1,MAGEA9B,TMEM185A |

**Supplementary Table 2: List of rare CNVs in 416 OPGP control individuals**

This list contains rare CNVs detected in 416 OPGP control samples of European ancestry that were not detected in 2,357 control individuals, using a 50% reciprocal overlap criteria. All CNVs are stringent (detected by 2 or more algorithms) and span at least 10 kb in length and 5 or more Affymetrix 6.0 array probes. Genes overlapped by these CNVs were used in pathway analyses versus those overlapped by rare CNVs in TOF cases (Supplementary Table 1).

| **Sample** | **Sex** | **Chr** | **Start** | **End** | **Size** | **CNV** | **genes** |
| --- | --- | --- | --- | --- | --- | --- | --- |
| OPGP246 | M | 1 | 3,132,655 | 3,225,901 | 93,247 | gain | PRDM16 |
| OPGP223 | M | 1 | 3,162,562 | 3,230,995 | 68,434 | gain | PRDM16 |
| OPGP248 | M | 1 | 3,273,162 | 3,502,376 | 229,215 | gain | PRDM16,MIR551A,MEGF6,ARHGEF16 |
| SS127_OPGP2 | F | 1 | 10,918,066 | 10,975,411 | 57,346 | loss | C1orf127 |
| OPGP-SS15 | F | 1 | 19,592,679 | 19,608,982 | 16,304 | loss | CAPZB |
| OPGP262 | F | 1 | 26,685,662 | 26,722,882 | 37,221 | loss | - |
| OPGP146 | M | 1 | 33,137,332 | 33,344,564 | 207,233 | gain | AK2,TMEM54,RNF19B,ADC |
| SS122_OPGP2 | F | 1 | 40,738,766 | 40,753,817 | 15,052 | loss | DEM1 |
| OPGP-SS108 | M | 1 | 46,158,906 | 46,278,997 | 120,092 | gain | MAST2,PIK3R3 |
| OPGP-SS12 | F | 1 | 46,243,892 | 46,306,844 | 62,953 | gain | MAST2,PIK3R3 |
| SS81_OPGP2 | F | 1 | 50,062,688 | 50,089,082 | 26,395 | loss | AGBL4 |
| SS85_OPGP2 | M | 1 | 55,268,332 | 55,280,544 | 12,213 | loss | PCSK9 |
| SS58_OPGP2 | F | 1 | 58,121,593 | 58,147,861 | 26,269 | gain | DAB1 |
| OPGP-SS77 | M | 1 | 71,372,770 | 71,425,908 | 53,139 | loss | - |
| OPGP-SS10 | F | 1 | 76,002,427 | 76,017,962 | 15,536 | loss | - |
| OPGP-SS63 | F | 1 | 76,844,658 | 76,875,040 | 30,383 | gain | ST6GALNAC3 |
| OPGP199 | F | 1 | 86,476,458 | 86,681,286 | 204,829 | gain | CLCA2,ODF2L |
| OPGP58 | F | 1 | 86,686,760 | 86,766,066 | 79,307 | gain | CLCA2,CLCA1 |
| OPGP58 | F | 1 | 87,079,658 | 87,247,940 | 168,283 | gain | HS2ST1,SEP15 |
| OPGP133 | M | 1 | 91,962,660 | 91,991,656 | 28,997 | gain | TGFBR3 |
| OPGP235 | F | 1 | 92,787,354 | 92,821,078 | 33,725 | loss | EVI5 |
| OPGP-SS71 | M | 1 | 101,816,320 | 102,613,362 | 797,043 | gain | OLFM3,DNAJA1P5 |
| OPGP80 | F | 1 | 104,901,435 | 104,966,009 | 64,575 | loss | - |
| OPGP-SS47 | M | 1 | 107,863,323 | 107,875,050 | 11,728 | loss | - |
| OPGP231 | F | 1 | 111,645,981 | 111,686,452 | 40,472 | loss | CHIA |
| OPGP130 | F | 1 | 113,105,462 | 113,378,590 | 273,129 | gain | SLC16A1,AKR7A2P1 |
| OPGP-SS68 | F | 1 | 113,615,510 | 113,930,997 | 315,488 | gain | MAGI3 |
| OPGP168 | M | 1 | 145,433,143 | 145,445,807 | 12,665 | gain | - |
| OPGP-SS02 | F | 1 | 152,087,315 | 152,103,245 | 15,931 | loss | GATAD2B |
| OPGP-SS30 | F | 1 | 156,720,980 | 156,780,407 | 59,428 | loss | - |
| OPGP240 | F | 1 | 165,460,615 | 165,471,136 | 10,522 | gain | POU2F1 |
| SS59_OPGP2 | F | 1 | 168,007,475 | 168,018,527 | 11,053 | loss | - |
| OPGP82 | F | 1 | 186,906,390 | 186,964,586 | 58,197 | loss | - |
| OPGP-SS85 | F | 1 | 187,884,325 | 187,928,475 | 44,151 | loss | - |
| OPGP-SS71 | M | 1 | 189,259,675 | 189,313,065 | 53,391 | loss | - |
| OPGP-SS77 | M | 1 | 189,954,806 | 190,006,828 | 52,023 | loss | - |
| OPGP262 | F | 1 | 194,006,743 | 194,258,287 | 251,545 | loss | - |
| SS88_OPGP2 | M | 1 | 206,849,922 | 207,054,121 | 204,200 | gain | - |
| OPGP175 | F | 1 | 210,617,154 | 210,640,252 | 23,099 | loss | TMEM206 |
| OPGP27 | M | 1 | 212,711,886 | 212,760,921 | 49,036 | loss | PTPN14 |
| OPGP-SS83 | M | 1 | 213,519,909 | 213,596,382 | 76,474 | loss | - |
| OPGP185 | F | 1 | 216,790,158 | 217,087,235 | 297,078 | gain | - |
| OPGP201 | F | 1 | 221,699,976 | 221,773,616 | 73,641 | gain | - |
| OPGP30 | F | 1 | 237,168,933 | 237,189,486 | 20,554 | loss | - |
| OPGP96 | M | 1 | 242,020,777 | 242,070,772 | 49,996 | gain | AKT3 |
| OPGP-SS19 | M | 1 | 243,756,388 | 244,239,828 | 483,441 | gain | KIF26B,SMYD3 |
| OPGP-SS118 | M | 1 | 246,218,668 | 246,276,668 | 58,001 | loss | OR2L1P,OR2L13,OR2L2 |
| OPGP-SS51 | M | 2 | 3,836,936 | 3,849,326 | 12,391 | loss | - |
| SS126_OPGP2 | F | 2 | 12,003,792 | 12,026,252 | 22,461 | loss | - |
| OPGP170 | F | 2 | 13,958,075 | 14,652,091 | 694,017 | loss | - |
| OPGP135 | M | 2 | 22,366,001 | 22,459,477 | 93,477 | loss | - |
| OPGP246 | M | 2 | 23,379,382 | 23,412,430 | 33,049 | loss | - |
| SS128_OPGP2 | F | 2 | 35,198,444 | 35,265,044 | 66,601 | loss | - |
| OPGP101 | M | 2 | 40,069,967 | 40,309,879 | 239,913 | gain | SLC8A1 |
| SS60_OPGP2 | F | 2 | 40,998,583 | 41,084,439 | 85,857 | loss | - |
| OPGP-SS19 | M | 2 | 40,998,583 | 41,084,439 | 85,857 | loss | - |
| OPGP-SS01 | M | 2 | 44,596,021 | 44,621,047 | 25,027 | loss | CAMKMT |
| OPGP-SS04 | M | 2 | 45,334,332 | 45,381,926 | 47,595 | loss | UNQ6975 |
| OPGP145 | M | 2 | 51,190,991 | 51,211,206 | 20,216 | loss | - |
| SS41_OPGP2 | M | 2 | 53,537,059 | 53,562,047 | 24,989 | loss | - |
| OPGP-SS02 | F | 2 | 55,893,434 | 55,981,843 | 88,410 | loss | EFEMP1 |
| OPGP-SS84 | F | 2 | 57,446,156 | 57,535,320 | 89,165 | gain | - |
| OPGP-SS33 | F | 2 | 59,157,327 | 59,178,260 | 20,934 | gain | - |
| OPGP-SS51 | M | 2 | 77,084,854 | 77,108,672 | 23,819 | gain | LRRTM4 |
| OPGP160 | M | 2 | 78,599,709 | 78,663,818 | 64,110 | loss | - |
| OPGP-SS65 | F | 2 | 80,217,697 | 80,268,753 | 51,057 | loss | CTNNA2 |
| OPGP194 | M | 2 | 82,866,310 | 83,147,178 | 280,869 | loss | LOC1720 |
| SS64_OPGP2 | F | 2 | 88,907,418 | 88,992,571 | 85,154 | gain | - |
| SS87_OPGP2 | M | 2 | 88,907,418 | 88,992,571 | 85,154 | gain | - |
| SS32_OPGP2 | M | 2 | 107,763,445 | 107,781,596 | 18,152 | loss | - |
| OPGP84 | M | 2 | 129,180,187 | 129,229,752 | 49,566 | gain | - |
| OPGP-SS106 | M | 2 | 131,194,418 | 131,222,786 | 28,369 | gain | GPR148 |
| SS34_OPGP2 | M | 2 | 136,737,724 | 136,759,546 | 21,823 | loss | - |
| OPGP-SS52 | M | 2 | 137,392,303 | 137,434,487 | 42,185 | loss | - |
| OPGP60 | F | 2 | 137,640,231 | 137,939,320 | 299,090 | loss | THSD7B |
| SS39_OPGP2 | M | 2 | 148,728,411 | 148,781,340 | 52,930 | loss | MBD5 |
| OPGP136 | M | 2 | 175,315,568 | 175,338,721 | 23,154 | loss | CHRNA1 |
| OPGP-SS63 | F | 2 | 179,561,710 | 179,605,627 | 43,918 | loss | CCDC141 |
| OPGP238 | F | 2 | 183,223,185 | 183,279,605 | 56,421 | loss | - |
| OPGP102 | M | 2 | 197,249,313 | 197,663,025 | 413,713 | gain | PGAP1,ANKRD44,LOC100130452,GTF3C3,CCDC150,C2orf66 |
| OPGP171 | F | 2 | 199,419,959 | 199,545,890 | 125,932 | gain | - |
| OPGP-SS74 | M | 2 | 205,563,673 | 205,663,716 | 100,044 | loss | PARD3B |
| OPGP84 | M | 2 | 212,798,254 | 212,889,567 | 91,314 | loss | ERBB4 |
| SS88_OPGP2 | M | 2 | 214,308,244 | 214,325,958 | 17,715 | loss | SPAG16 |
| OPGP-SS24 | F | 2 | 231,122,629 | 231,137,464 | 14,836 | gain | - |
| OPGP-SS53 | F | 2 | 235,871,664 | 236,172,371 | 300,708 | gain | AGAP1 |
| OPGP-SS53 | F | 2 | 236,798,401 | 237,062,102 | 263,702 | gain | ASB18,IQCA1 |
| OPGP-SS82 | M | 2 | 237,295,053 | 237,338,319 | 43,267 | loss | - |
| OPGP-SS73 | F | 3 | 1,714,339 | 1,768,951 | 54,613 | loss | - |
| OPGP13 | M | 3 | 1,779,655 | 1,805,430 | 25,776 | loss | - |
| OPGP34 | F | 3 | 1,904,322 | 1,927,460 | 23,139 | loss | - |
| OPGP153 | M | 3 | 5,364,179 | 5,411,289 | 47,111 | loss | - |
| OPGP-SS92 | M | 3 | 5,364,179 | 5,411,289 | 47,111 | loss | - |
| OPGP258 | M | 3 | 5,582,285 | 5,607,263 | 24,979 | loss | - |
| SS71_OPGP2 | M | 3 | 6,546,105 | 6,573,353 | 27,249 | loss | - |
| OPGP110 | F | 3 | 12,543,425 | 12,648,371 | 104,947 | gain | LOC100129480,MKRN2,RAF1,TSEN2 |
| OPGP51 | M | 3 | 15,964,963 | 15,989,921 | 24,959 | loss | - |
| OPGP-SS110 | M | 3 | 22,212,602 | 22,280,792 | 68,191 | gain | - |
| OPGP-SS26 | F | 3 | 23,658,782 | 23,819,436 | 160,655 | gain | - |
| OPGP65 | F | 3 | 26,352,082 | 26,423,930 | 71,849 | loss | - |
| OPGP189 | M | 3 | 34,701,725 | 34,781,687 | 79,963 | loss | - |
| OPGP35 | F | 3 | 34,884,209 | 34,916,077 | 31,869 | loss | - |
| OPGP245 | M | 3 | 41,776,668 | 41,866,580 | 89,913 | loss | ULK4 |
| OPGP98 | M | 3 | 53,722,720 | 53,895,962 | 173,243 | gain | CHDH,SELK,CACNA1D,IL17RB,ACTR8 |
| SS76_OPGP2 | M | 3 | 59,829,199 | 61,005,753 | 1,176,555 | loss | FHIT |
| SS121_OPGP2 | F | 3 | 65,647,659 | 65,693,144 | 45,486 | loss | MAGI1 |
| OPGP-SS63 | F | 3 | 105,512,260 | 105,564,637 | 52,378 | loss | - |
| OPGP178 | F | 3 | 107,896,596 | 107,950,455 | 53,860 | gain | - |
| OPGP269 | F | 3 | 109,497,937 | 109,760,767 | 262,831 | gain | HHLA2,KIAA1524,MYH15 |
| OPGP243 | F | 3 | 109,908,932 | 110,134,824 | 225,893 | loss | RETNLB,TRAT1,GUCA1C |
| OPGP-SS49 | M | 3 | 123,880,407 | 124,235,121 | 354,715 | gain | PARP14,DIRC2,HSPBAP1,SEMA5B,LOC100129550 |
| OPGP153 | M | 3 | 144,956,132 | 145,145,097 | 188,966 | loss | SLC9A9 |
| OPGP262 | F | 3 | 145,665,527 | 145,678,368 | 12,842 | loss | - |
| OPGP33 | F | 3 | 147,125,201 | 147,144,641 | 19,441 | loss | - |
| SS121_OPGP2 | F | 3 | 154,870,103 | 155,031,925 | 161,823 | gain | - |
| OPGP33 | F | 3 | 163,520,556 | 163,606,060 | 85,505 | loss | - |
| OPGP-SS111 | M | 3 | 166,179,336 | 166,273,847 | 94,512 | gain | SI |
| OPGP189 | M | 3 | 176,569,482 | 177,229,640 | 660,159 | loss | NAALADL2 |
| OPGP59 | M | 3 | 183,836,513 | 183,862,220 | 25,708 | loss | - |
| OPGP08 | M | 3 | 191,159,675 | 191,482,446 | 322,772 | loss | LEPREL1 |
| OPGP224 | M | 3 | 193,459,773 | 193,499,362 | 39,590 | loss | FGF12 |
| OPGP-SS48 | M | 3 | 195,719,679 | 195,750,493 | 30,815 | gain | - |
| OPGP117 | F | 3 | 199,157,125 | 199,170,719 | 13,595 | gain | IQCG,RPL35A |
| OPGP268 | F | 4 | 1,042,239 | 1,107,925 | 65,687 | gain | TMED11P,RNF212 |
| OPGP261 | F | 4 | 2,355,110 | 2,366,882 | 11,773 | gain | ZFYVE28 |
| OPGP-SS76 | F | 4 | 2,933,890 | 2,980,407 | 46,518 | gain | GRK4,NOP14 |
| OPGP20 | F | 4 | 9,853,711 | 9,890,922 | 37,212 | loss | - |
| OPGP46 | F | 4 | 19,220,177 | 19,264,426 | 44,250 | loss | - |
| OPGP-SS14 | F | 4 | 21,152,913 | 21,175,276 | 22,364 | loss | KCNIP4 |
| OPGP252 | F | 4 | 28,348,262 | 28,474,772 | 126,511 | loss | MIR4275 |
| OPGP289 | F | 4 | 39,602,122 | 39,791,634 | 189,513 | gain | LOC344967,PDS5A,N4BP2 |
| SS126_OPGP2 | F | 4 | 39,692,394 | 39,737,695 | 45,302 | gain | LOC344967,N4BP2 |
| OPGP06 | M | 4 | 43,325,028 | 43,371,077 | 46,050 | loss | - |
| OPGP44 | F | 4 | 56,819,933 | 56,837,598 | 17,666 | gain | KIAA1211 |
| OPGP147 | M | 4 | 62,227,896 | 62,267,377 | 39,482 | loss | LPHN3 |
| OPGP10 | F | 4 | 73,100,660 | 73,122,182 | 21,523 | loss | NPFFR2 |
| OPGP-SS24 | F | 4 | 79,908,025 | 79,924,975 | 16,951 | gain | BMP2K |
| OPGP84 | M | 4 | 82,700,206 | 82,738,912 | 38,707 | loss | - |
| OPGP51 | M | 4 | 91,887,762 | 91,959,857 | 72,096 | gain | FAM190A |
| OPGP209 | M | 4 | 93,692,083 | 93,709,753 | 17,671 | loss | GRID2 |
| OPGP-SS18 | M | 4 | 95,258,776 | 95,282,631 | 23,856 | gain | - |
| OPGP215 | M | 4 | 102,038,141 | 102,103,608 | 65,468 | loss | - |
| OPGP246 | M | 4 | 102,944,497 | 102,981,734 | 37,238 | gain | BANK1 |
| SS58_OPGP2 | F | 4 | 103,825,185 | 103,864,128 | 38,944 | gain | MANBA |
| OPGP241 | F | 4 | 104,111,158 | 104,147,496 | 36,339 | loss | NHEDC1 |
| OPGP215 | M | 4 | 112,516,864 | 112,529,063 | 12,200 | loss | - |
| OPGP227 | M | 4 | 113,267,887 | 113,324,725 | 56,839 | gain | C4orf32 |
| OPGP01 | F | 4 | 116,420,120 | 116,437,050 | 16,931 | loss | - |
| OPGP243 | F | 4 | 116,422,771 | 116,438,169 | 15,399 | loss | - |
| OPGP119 | F | 4 | 117,070,336 | 117,987,237 | 916,902 | gain | MIR1973 |
| OPGP111 | F | 4 | 117,630,141 | 117,726,424 | 96,284 | gain | - |
| OPGP195 | M | 4 | 127,458,501 | 127,673,045 | 214,545 | loss | - |
| OPGP-SS27 | F | 4 | 131,182,407 | 131,224,159 | 41,753 | loss | - |
| OPGP-SS12 | F | 4 | 135,636,764 | 135,746,833 | 110,070 | loss | - |
| OPGP119 | F | 4 | 136,594,287 | 138,635,305 | 2,041,019 | loss | - |
| OPGP157 | F | 4 | 138,083,646 | 138,098,754 | 15,109 | loss | - |
| OPGP07 | M | 4 | 139,893,157 | 139,927,943 | 34,787 | loss | - |
| SS53_OPGP2 | F | 4 | 143,025,931 | 143,050,531 | 24,601 | gain | - |
| OPGP249 | M | 4 | 148,961,558 | 149,040,682 | 79,125 | loss | ARHGAP10 |
| OPGP47 | F | 4 | 155,304,052 | 155,331,427 | 27,376 | loss | - |
| OPGP151 | M | 4 | 157,098,371 | 157,119,459 | 21,089 | loss | - |
| SS132_OPGP2 | F | 4 | 160,707,810 | 160,718,617 | 10,808 | loss | - |
| OPGP07 | M | 4 | 161,769,134 | 161,895,127 | 125,994 | loss | - |
| OPGP158 | M | 4 | 162,022,141 | 162,050,907 | 28,767 | loss | - |
| SS80_OPGP2 | M | 4 | 163,381,909 | 163,402,576 | 20,668 | loss | - |
| OPGP-SS03 | F | 4 | 166,213,561 | 166,244,840 | 31,280 | loss | TMEM192 |
| OPGP-SS09 | F | 4 | 167,741,303 | 167,828,197 | 86,895 | gain | - |
| OPGP146 | M | 4 | 179,672,854 | 179,697,140 | 24,287 | loss | - |
| OPGP149 | M | 4 | 182,157,291 | 182,182,771 | 25,481 | loss | - |
| SS121_OPGP2 | F | 4 | 182,728,030 | 182,790,037 | 62,008 | loss | - |
| OPGP225 | M | 4 | 185,971,583 | 185,996,924 | 25,342 | loss | ACSL1 |
| OPGP07 | M | 4 | 188,089,824 | 188,686,081 | 596,258 | loss | - |
| OPGP238 | F | 4 | 188,531,974 | 188,763,652 | 231,679 | gain | - |
| OPGP137 | M | 4 | 190,260,773 | 191,261,904 | 1,001,132 | loss | FRG1,FRG2,HSP90AA4P,LOC100288255,DUX2,DUX4L7,DUX4L6,DUX4L5,DUX4L4,DUX4L3,DUX4L2,DUX4,TUBB4Q |
| OPGP248 | M | 5 | 1,055,489 | 1,274,261 | 218,773 | gain | SLC12A7,NKD2,SLC6A19 |
| OPGP116 | F | 5 | 11,798,512 | 11,820,720 | 22,209 | gain | CTNND2 |
| SS132_OPGP2 | F | 5 | 12,251,381 | 12,493,224 | 241,844 | loss | - |
| OPGP120 | F | 5 | 12,833,942 | 13,290,184 | 456,243 | gain | TAG |
| SS70_OPGP2 | M | 5 | 16,243,137 | 16,286,148 | 43,012 | loss | - |
| OPGP165 | M | 5 | 18,608,303 | 18,622,391 | 14,089 | gain | - |
| OPGP61 | F | 5 | 19,053,377 | 19,084,750 | 31,374 | gain | - |
| SS31_OPGP2 | M | 5 | 19,318,840 | 19,348,690 | 29,851 | gain | - |
| OPGP92 | M | 5 | 21,885,181 | 22,025,871 | 140,691 | loss | CDH12 |
| OPGP151 | M | 5 | 26,160,848 | 26,270,673 | 109,826 | gain | - |
| OPGP95 | M | 5 | 29,372,002 | 29,444,560 | 72,559 | loss | - |
| OPGP35 | F | 5 | 35,894,768 | 35,905,874 | 11,107 | loss | IL7R |
| OPGP-SS63 | F | 5 | 43,672,941 | 44,261,062 | 588,122 | gain | NNT |
| OPGP238 | F | 5 | 44,166,372 | 44,261,062 | 94,691 | loss | - |
| OPGP284 | M | 5 | 56,382,095 | 56,399,764 | 17,670 | loss | - |
| OPGP249 | M | 5 | 56,713,368 | 57,357,403 | 644,036 | gain | ACTBL2 |
| OPGP-SS63 | F | 5 | 57,890,289 | 57,931,235 | 40,947 | gain | RAB3C |
| OPGP-SS76 | F | 5 | 61,838,573 | 62,004,154 | 165,582 | gain | LRRC70,IPO11 |
| OPGP84 | M | 5 | 63,974,156 | 64,241,132 | 266,977 | gain | FAM159B,CWC27,SREK1IP1 |
| SS55_OPGP2 | F | 5 | 75,647,353 | 75,682,250 | 34,898 | gain | SV2C |
| SS31_OPGP2 | M | 5 | 82,547,252 | 82,727,044 | 179,793 | gain | XRCC4 |
| OPGP-SS100 | M | 5 | 86,780,522 | 86,821,528 | 41,007 | loss | - |
| SS74_OPGP2 | M | 5 | 102,751,434 | 102,809,432 | 57,999 | gain | - |
| OPGP-SS31 | F | 5 | 104,262,266 | 104,280,607 | 18,342 | gain | - |
| OPGP-SS03 | F | 5 | 104,594,666 | 104,627,852 | 33,187 | loss | - |
| OPGP02 | F | 5 | 107,390,269 | 107,475,020 | 84,752 | loss | FBXL17 |
| OPGP02 | F | 5 | 107,527,862 | 107,543,600 | 15,739 | gain | FBXL17 |
| OPGP67 | F | 5 | 110,250,786 | 110,266,346 | 15,561 | loss | - |
| OPGP-SS62 | F | 5 | 112,756,381 | 112,797,440 | 41,060 | loss | TSSK1B,MCC |
| OPGP189 | M | 5 | 115,661,373 | 115,704,298 | 42,926 | loss | - |
| OPGP104 | M | 5 | 117,768,399 | 118,049,462 | 281,064 | loss | - |
| OPGP36 | M | 5 | 130,013,729 | 131,087,508 | 1,073,780 | gain | CDC42SE2,RAPGEF6,LYRM7,HINT1,FNIP1 |
| OPGP224 | M | 5 | 134,047,374 | 134,143,412 | 96,039 | gain | CAMLG,SEC24A,DDX46 |
| OPGP-SS31 | F | 5 | 143,624,001 | 143,665,623 | 41,623 | gain | KCTD16 |
| SS75_OPGP2 | M | 5 | 149,576,572 | 149,605,730 | 29,159 | gain | CAMK2A |
| OPGP174 | F | 5 | 152,906,598 | 152,964,673 | 58,076 | gain | GRIA1 |
| OPGP104 | M | 5 | 156,334,768 | 156,580,366 | 245,599 | gain | ITK,FAM71B,HAVCR1,HAVCR2,MED7 |
| SS73_OPGP2 | M | 5 | 165,653,184 | 165,666,864 | 13,681 | loss | - |
| OPGP171 | F | 5 | 165,771,420 | 165,782,533 | 11,114 | loss | - |
| OPGP110 | F | 5 | 166,750,150 | 166,766,453 | 16,304 | loss | ODZ2 |
| OPGP-SS69 | M | 5 | 173,488,073 | 173,592,953 | 104,881 | gain | - |
| OPGP276 | F | 5 | 179,381,594 | 179,443,248 | 61,655 | loss | RNF130 |
| SS121_OPGP2 | F | 5 | 179,802,201 | 180,278,690 | 476,490 | gain | BTNL8,OR2Y1,LOC729678,SCGB3A1,ZFP62,MGAT1,FLT4,CNOT6 |
| OPGP-SS49 | M | 6 | 94,661 | 167,304 | 72,644 | loss | - |
| SS83_OPGP2 | M | 6 | 5,315,611 | 5,503,575 | 187,965 | loss | FARS2 |
| OPGP-SS42 | F | 6 | 8,975,313 | 8,987,431 | 12,119 | gain | - |
| OPGP78 | F | 6 | 17,565,591 | 17,788,094 | 222,504 | gain | FAM8A1,NUP153,CAP2 |
| SS132_OPGP2 | F | 6 | 19,005,366 | 19,016,871 | 11,506 | loss | - |
| OPGP-SS39 | F | 6 | 27,229,203 | 27,273,066 | 43,864 | loss | - |
| OPGP126 | F | 6 | 28,502,550 | 28,762,131 | 259,582 | gain | GPX6,GPX5,ZSCAN23 |
| OPGP183 | F | 6 | 29,364,297 | 29,382,592 | 18,296 | loss | OR14J1 |
| OPGP269 | F | 6 | 36,385,529 | 36,410,188 | 24,660 | loss | C6orf222 |
| OPGP-SS53 | F | 6 | 37,599,494 | 37,609,945 | 10,452 | loss | - |
| OPGP107 | F | 6 | 37,941,581 | 38,260,407 | 318,827 | gain | BTBD9,ZFAND3 |
| OPGP-SS52 | M | 6 | 42,010,033 | 42,023,695 | 13,663 | gain | CCND3 |
| SS73_OPGP2 | M | 6 | 46,062,737 | 46,182,615 | 119,879 | gain | CLIC5 |
| OPGP-SS110 | M | 6 | 46,182,615 | 46,266,012 | 83,398 | gain | ENPP4,ENPP5 |
| OPGP-SS108 | M | 6 | 49,641,246 | 49,673,535 | 32,290 | loss | - |
| OPGP209 | M | 6 | 50,305,498 | 50,317,332 | 11,835 | loss | - |
| OPGP54 | M | 6 | 57,210,013 | 57,249,352 | 39,340 | loss | - |
| OPGP205 | M | 6 | 63,659,441 | 63,711,303 | 51,863 | loss | - |
| OPGP77 | F | 6 | 65,659,578 | 65,714,245 | 54,668 | loss | EYS |
| OPGP-SS100 | M | 6 | 65,954,266 | 66,545,669 | 591,404 | gain | EYS |
| OPGP23 | F | 6 | 67,189,492 | 67,395,975 | 206,484 | gain | - |
| OPGP17 | M | 6 | 68,731,624 | 68,793,856 | 62,233 | gain | - |
| SS86_OPGP2 | M | 6 | 76,206,233 | 76,253,570 | 47,338 | loss | FILIP1 |
| OPGP226 | M | 6 | 76,943,707 | 76,957,012 | 13,306 | loss | - |
| OPGP276 | F | 6 | 89,794,375 | 89,826,935 | 32,561 | loss | - |
| OPGP-SS09 | F | 6 | 92,817,483 | 92,839,706 | 22,224 | gain | - |
| OPGP-SS74 | M | 6 | 93,600,268 | 93,650,997 | 50,730 | loss | - |
| SS121_OPGP2 | F | 6 | 95,673,830 | 95,724,798 | 50,969 | loss | - |
| OPGP42 | M | 6 | 103,237,557 | 103,262,085 | 24,529 | loss | - |
| OPGP245 | M | 6 | 110,344,844 | 110,400,167 | 55,324 | loss | - |
| OPGP231 | F | 6 | 116,529,448 | 116,858,777 | 329,330 | gain | COL10A1,TSPYL4,DSE,TSPYL1,NT5DC1 |
| OPGP30 | F | 6 | 118,838,161 | 118,967,398 | 129,238 | gain | C6orf204,BRD7P3 |
| OPGP-SS65 | F | 6 | 118,910,606 | 118,920,621 | 10,016 | loss | C6orf204 |
| OPGP-SS77 | M | 6 | 120,388,063 | 120,490,882 | 102,820 | loss | - |
| OPGP96 | M | 6 | 121,335,988 | 121,555,376 | 219,389 | loss | C6orf170 |
| SS84_OPGP2 | M | 6 | 132,963,213 | 133,093,570 | 130,358 | loss | VNN3,VNN1,TAAR1,TAAR3,TAAR2 |
| OPGP98 | M | 6 | 141,022,503 | 141,104,763 | 82,261 | gain | - |
| OPGP79 | F | 6 | 142,565,022 | 142,614,314 | 49,293 | loss | VTA1 |
| SS56_OPGP2 | F | 6 | 150,221,702 | 150,341,162 | 119,461 | loss | LRP11,ULBP1,RAET1E,RAET1G,ULBP2 |
| OPGP288 | M | 6 | 152,964,606 | 152,997,648 | 33,043 | loss | SYNE1 |
| OPGP172 | F | 7 | 837,774 | 984,755 | 146,982 | loss | ADAP1,COX19,SUN1,GET4 |
| OPGP104 | M | 7 | 2,079,588 | 2,224,642 | 145,055 | loss | MAD1L1 |
| OPGP212 | M | 7 | 3,043,205 | 3,055,596 | 12,392 | gain | CARD11 |
| OPGP279 | F | 7 | 3,689,681 | 3,702,780 | 13,100 | loss | SDK1 |
| SS81_OPGP2 | F | 7 | 4,910,457 | 4,927,869 | 17,413 | loss | MMD2 |
| OPGP-SS05 | M | 7 | 4,910,457 | 4,922,064 | 11,608 | loss | MMD2 |
| OPGP75 | F | 7 | 7,170,546 | 7,245,535 | 74,990 | loss | C1GALT1 |
| OPGP187 | M | 7 | 7,464,930 | 7,567,379 | 102,450 | loss | COL28A1 |
| OPGP260 | M | 7 | 8,322,114 | 8,686,211 | 364,098 | gain | NXPH1 |
| SS74_OPGP2 | M | 7 | 8,925,485 | 9,000,195 | 74,711 | gain | - |
| OPGP269 | F | 7 | 9,471,158 | 9,501,930 | 30,773 | gain | - |
| OPGP-SS52 | M | 7 | 10,368,265 | 10,622,487 | 254,223 | loss | - |
| OPGP202 | F | 7 | 10,534,216 | 10,569,097 | 34,882 | loss | - |
| OPGP225 | M | 7 | 12,234,020 | 12,307,224 | 73,205 | gain | TMEM106B |
| OPGP225 | M | 7 | 16,003,433 | 16,035,549 | 32,117 | loss | - |
| OPGP-SS21 | M | 7 | 16,121,832 | 16,197,707 | 75,876 | loss | ISPD |
| OPGP151 | M | 7 | 17,017,534 | 17,033,970 | 16,437 | loss | - |
| OPGP159 | M | 7 | 17,092,924 | 17,154,865 | 61,942 | loss | - |
| OPGP14 | M | 7 | 20,003,498 | 20,039,131 | 35,634 | loss | - |
| OPGP241 | F | 7 | 20,100,754 | 20,150,035 | 49,282 | loss | MACC1 |
| SS73_OPGP2 | M | 7 | 24,958,926 | 24,984,487 | 25,562 | loss | OSBPL3 |
| OPGP164 | M | 7 | 25,081,173 | 25,108,084 | 26,912 | loss | - |
| OPGP46 | F | 7 | 31,945,813 | 31,984,018 | 38,206 | loss | PDE1C |
| OPGP168 | M | 7 | 33,874,598 | 33,885,395 | 10,798 | loss | - |
| SS128_OPGP2 | F | 7 | 37,363,787 | 37,439,919 | 76,133 | loss | ELMO1 |
| SS76_OPGP2 | M | 7 | 54,277,220 | 54,374,624 | 97,405 | gain | - |
| OPGP-SS113 | M | 7 | 63,771,570 | 63,912,686 | 141,117 | loss | ZNF138,ZNF107 |
| OPGP96 | M | 7 | 69,952,960 | 70,227,885 | 274,926 | gain | - |
| OPGP217 | M | 7 | 71,303,353 | 71,352,194 | 48,842 | loss | CALN1 |
| OPGP185 | F | 7 | 75,212,593 | 75,225,967 | 13,375 | gain | - |
| OPGP194 | M | 7 | 82,644,704 | 82,660,873 | 16,170 | gain | - |
| OPGP228 | M | 7 | 83,003,272 | 83,022,856 | 19,585 | gain | SEMA3E |
| OPGP33 | F | 7 | 85,470,643 | 85,574,947 | 104,305 | loss | - |
| OPGP124 | F | 7 | 96,025,677 | 96,047,043 | 21,367 | gain | - |
| OPGP149 | M | 7 | 98,891,250 | 98,975,124 | 83,875 | gain | ATP5J2,ATP5J2-PTCD1,ZKSCAN5,ZNF789,CPSF4,ZNF394 |
| OPGP09 | F | 7 | 110,262,044 | 110,357,477 | 95,434 | loss | IMMP2L |
| OPGP104 | M | 7 | 111,097,030 | 111,140,597 | 43,568 | loss | - |
| OPGP-SS52 | M | 7 | 117,319,061 | 117,373,836 | 54,776 | gain | - |
| OPGP190 | M | 7 | 135,811,683 | 135,836,026 | 24,344 | loss | - |
| OPGP175 | F | 7 | 143,311,253 | 143,321,679 | 10,427 | loss | - |
| OPGP92 | M | 7 | 152,309,916 | 152,780,053 | 470,138 | gain | - |
| OPGP06 | M | 8 | 173,696 | 307,492 | 133,797 | gain | ZNF596 |
| SS35_OPGP2 | M | 8 | 2,257,735 | 2,318,174 | 60,440 | loss | - |
| SS81_OPGP2 | F | 8 | 2,711,713 | 2,722,424 | 10,712 | loss | - |
| OPGP-SS70 | F | 8 | 4,299,489 | 4,323,910 | 24,422 | loss | CSMD1 |
| SS42_OPGP2 | M | 8 | 4,493,322 | 4,541,100 | 47,779 | loss | CSMD1 |
| OPGP04 | F | 8 | 4,805,813 | 4,816,996 | 11,184 | loss | CSMD1 |
| OPGP-SS43 | F | 8 | 4,822,195 | 4,840,333 | 18,139 | loss | CSMD1 |
| SS64_OPGP2 | F | 8 | 5,481,630 | 5,523,610 | 41,981 | loss | - |
| OPGP23 | F | 8 | 5,659,866 | 5,680,928 | 21,063 | loss | - |
| OPGP162 | M | 8 | 5,766,175 | 6,015,360 | 249,186 | loss | - |
| OPGP126 | F | 8 | 6,222,765 | 6,236,366 | 13,602 | loss | - |
| OPGP261 | F | 8 | 6,639,334 | 7,000,334 | 361,001 | gain | DEFA10P,DEFT1P2,DEFA6,DEFA5,DEFA4,DEFA3,DEFA1,DEFA1B,DEFT1P,XKR5,DEFB1 |
| OPGP244 | M | 8 | 8,375,229 | 8,385,745 | 10,517 | gain | - |
| SS56_OPGP2 | F | 8 | 11,018,996 | 11,056,179 | 37,184 | gain | XKR6 |
| SS131_OPGP2 | F | 8 | 17,709,875 | 17,754,832 | 44,958 | loss | - |
| OPGP-SS111 | M | 8 | 18,707,763 | 18,735,393 | 27,631 | loss | PSD3 |
| OPGP-SS111 | M | 8 | 32,415,229 | 32,440,158 | 24,930 | gain | NRG1 |
| OPGP-SS56 | M | 8 | 48,337,198 | 48,467,466 | 130,269 | gain | KIAA0146 |
| OPGP235 | F | 8 | 50,255,830 | 50,346,706 | 90,877 | gain | - |
| OPGP263 | F | 8 | 53,163,983 | 53,200,562 | 36,580 | loss | ST18 |
| OPGP291 | F | 8 | 55,846,938 | 56,174,269 | 327,332 | loss | - |
| OPGP193 | M | 8 | 67,952,366 | 67,983,383 | 31,018 | loss | C8orf45 |
| OPGP241 | F | 8 | 68,188,369 | 68,324,256 | 135,888 | gain | CSPP1,ARFGEF1 |
| OPGP156 | M | 8 | 88,226,672 | 88,249,601 | 22,930 | loss | CNBD1 |
| OPGP16 | M | 8 | 90,540,918 | 90,857,097 | 316,180 | gain | RIPK2 |
| SS88_OPGP2 | M | 8 | 90,906,777 | 90,923,153 | 16,377 | loss | - |
| OPGP-SS119 | F | 8 | 95,811,805 | 95,848,084 | 36,280 | loss | DPY19L4 |
| OPGP69 | M | 8 | 106,053,559 | 106,172,202 | 118,644 | loss | - |
| OPGP-SS15 | F | 8 | 106,057,535 | 106,115,416 | 57,882 | gain | - |
| OPGP277 | F | 8 | 115,818,013 | 115,979,174 | 161,162 | loss | - |
| OPGP262 | F | 8 | 118,987,651 | 118,998,009 | 10,359 | loss | EXT1 |
| SS64_OPGP2 | F | 8 | 121,483,185 | 121,517,071 | 33,887 | loss | MRPL13 |
| OPGP35 | F | 8 | 126,139,466 | 126,192,164 | 52,699 | loss | NSMCE2,KIAA0196 |
| OPGP07 | M | 8 | 127,720,085 | 127,732,229 | 12,145 | loss | - |
| OPGP272 | F | 8 | 128,699,305 | 128,737,551 | 38,247 | loss | - |
| OPGP248 | M | 8 | 143,368,085 | 143,455,401 | 87,317 | gain | TSNARE1 |
| OPGP33 | F | 9 | 324,524 | 360,439 | 35,916 | loss | DOCK8 |
| OPGP59 | M | 9 | 551,382 | 789,644 | 238,263 | gain | KANK1 |
| OPGP-SS81 | F | 9 | 1,267,906 | 1,288,566 | 20,661 | gain | - |
| OPGP212 | M | 9 | 2,278,588 | 2,342,431 | 63,844 | loss | - |
| OPGP159 | M | 9 | 7,861,163 | 7,922,619 | 61,457 | loss | - |
| OPGP05 | M | 9 | 9,324,204 | 9,577,948 | 253,745 | loss | PTPRD |
| OPGP-SS14 | F | 9 | 9,545,365 | 9,562,879 | 17,515 | gain | PTPRD |
| OPGP-SS14 | F | 9 | 10,244,793 | 10,315,264 | 70,472 | gain | PTPRD |
| SS68_OPGP2 | M | 9 | 11,247,448 | 11,266,796 | 19,349 | loss | - |
| OPGP-SS55 | F | 9 | 12,902,368 | 12,925,849 | 23,482 | loss | - |
| SS56_OPGP2 | F | 9 | 15,844,376 | 15,934,791 | 90,416 | gain | C9orf93 |
| OPGP216 | M | 9 | 15,898,399 | 15,970,320 | 71,922 | loss | C9orf93 |
| OPGP-SS93 | F | 9 | 18,172,087 | 18,238,172 | 66,086 | loss | - |
| OPGP-SS50 | M | 9 | 19,275,457 | 19,320,042 | 44,586 | loss | DENND4C |
| OPGP114 | F | 9 | 20,599,706 | 20,629,298 | 29,593 | gain | MLLT3 |
| OPGP163 | M | 9 | 22,499,340 | 22,555,485 | 56,146 | loss | - |
| OPGP-SS43 | F | 9 | 22,679,215 | 22,742,395 | 63,181 | loss | - |
| SS72_OPGP2 | M | 9 | 27,317,823 | 27,366,422 | 48,600 | gain | MOBKL2B |
| SS61_OPGP2 | F | 9 | 32,621,821 | 32,700,249 | 78,429 | gain | TAF1L |
| OPGP176 | F | 9 | 73,309,592 | 73,481,836 | 172,245 | gain | - |
| OPGP34 | F | 9 | 77,174,742 | 77,230,547 | 55,806 | gain | - |
| OPGP-SS76 | F | 9 | 86,876,079 | 86,901,212 | 25,134 | gain | - |
| OPGP-SS93 | F | 9 | 93,327,123 | 93,377,810 | 50,688 | gain | - |
| OPGP-SS93 | F | 9 | 93,454,057 | 93,567,716 | 113,660 | gain | ROR2 |
| OPGP31 | F | 9 | 93,470,353 | 93,484,097 | 13,745 | gain | - |
| OPGP147 | M | 9 | 96,532,816 | 96,545,429 | 12,614 | loss | C9orf3 |
| OPGP-SS97 | M | 9 | 104,300,323 | 104,350,673 | 50,351 | loss | - |
| OPGP206 | M | 9 | 105,438,547 | 105,465,488 | 26,942 | loss | - |
| OPGP287 | M | 9 | 121,280,124 | 121,301,012 | 20,889 | loss | - |
| OPGP49 | F | 9 | 133,789,187 | 133,819,010 | 29,824 | loss | MED27 |
| OPGP248 | M | 9 | 136,734,887 | 136,764,182 | 29,296 | gain | COL5A1 |
| OPGP246 | M | 9 | 138,501,746 | 138,672,282 | 170,537 | gain | NOTCH1 |
| SS30_OPGP2 | M | 10 | 1,982,646 | 2,085,776 | 103,131 | gain | - |
| SS127_OPGP2 | F | 10 | 2,610,938 | 2,625,988 | 15,051 | loss | - |
| OPGP287 | M | 10 | 7,602,955 | 7,621,471 | 18,517 | loss | - |
| OPGP-SS38 | M | 10 | 7,672,510 | 7,684,255 | 11,746 | loss | ITIH5 |
| OPGP-SS58 | F | 10 | 17,349,910 | 17,530,579 | 180,670 | loss | ST8SIA6,LOC100128098 |
| OPGP179 | M | 10 | 20,902,433 | 21,064,339 | 161,907 | gain | - |
| OPGP66 | F | 10 | 35,200,211 | 35,348,514 | 148,304 | gain | CUL2 |
| OPGP287 | M | 10 | 35,565,000 | 35,647,246 | 82,247 | gain | CCNY |
| OPGP05 | M | 10 | 44,739,339 | 44,868,035 | 128,697 | gain | C10orf10,RASSF4,LOC220980,ZNF22,TMEM72,C10orf25 |
| OPGP237 | F | 10 | 55,882,676 | 55,902,378 | 19,703 | gain | PCDH15 |
| OPGP55 | F | 10 | 56,979,431 | 57,003,485 | 24,055 | gain | - |
| OPGP248 | M | 10 | 58,880,511 | 58,914,629 | 34,119 | loss | - |
| OPGP98 | M | 10 | 75,669,152 | 75,930,726 | 261,575 | loss | ADK |
| OPGP28 | M | 10 | 98,269,043 | 98,460,286 | 191,244 | gain | PIK3AP1,TM9SF3 |
| OPGP225 | M | 10 | 105,148,048 | 105,303,220 | 155,173 | gain | PDCD11,NEURL,CALHM1,CALHM3,CALHM2 |
| OPGP29 | M | 10 | 117,248,910 | 117,305,474 | 56,565 | loss | ATRNL1 |
| OPGP282 | M | 10 | 122,419,759 | 122,459,873 | 40,115 | gain | - |
| OPGP-SS36 | M | 10 | 130,363,010 | 131,616,730 | 1,253,721 | gain | MIR4297,EBF3,MGMT |
| OPGP13 | M | 10 | 130,941,167 | 130,961,684 | 20,518 | gain | - |
| SS130_OPGP2 | M | 10 | 133,999,987 | 134,027,438 | 27,452 | gain | LRRC27 |
| OPGP05 | M | 10 | 134,005,903 | 134,054,275 | 48,373 | gain | LRRC27 |
| OPGP94 | M | 11 | 9,779,172 | 9,845,638 | 66,467 | loss | LOC283104,SBF2 |
| OPGP35 | F | 11 | 11,505,249 | 11,516,232 | 10,984 | gain | GALNTL4 |
| OPGP01 | F | 11 | 21,471,167 | 21,527,034 | 55,868 | loss | NELL1 |
| SS66_OPGP2 | M | 11 | 23,772,032 | 23,809,934 | 37,903 | gain | - |
| OPGP231 | F | 11 | 30,029,067 | 30,059,899 | 30,833 | gain | - |
| OPGP266 | F | 11 | 30,708,854 | 30,720,172 | 11,319 | loss | - |
| OPGP146 | M | 11 | 31,226,693 | 31,282,996 | 56,304 | loss | DCDC1 |
| OPGP36 | M | 11 | 36,351,090 | 36,373,228 | 22,139 | gain | PRR5L |
| OPGP188 | M | 11 | 36,512,834 | 36,529,598 | 16,765 | loss | - |
| OPGP29 | M | 11 | 40,497,095 | 40,547,130 | 50,036 | loss | - |
| OPGP101 | M | 11 | 43,385,497 | 43,408,695 | 23,199 | gain | TTC17 |
| OPGP285 | M | 11 | 55,534,978 | 55,803,476 | 268,499 | loss | OR5J2,OR8H2,OR8H3,OR8I2,OR5T1,OR5T3,OR5T2,OR8J3,OR8K5,OR5AS1 |
| OPGP03 | F | 11 | 55,612,642 | 55,665,287 | 52,646 | gain | OR8J3,OR8H2,OR8H3,OR8I2 |
| OPGP03 | F | 11 | 55,675,275 | 55,817,112 | 141,838 | loss | OR5J2,OR8H1,OR5T1,OR5T3,OR5T2,OR8K5 |
| OPGP-SS51 | M | 11 | 68,480,846 | 68,493,638 | 12,793 | loss | - |
| OPGP-SS116 | M | 11 | 79,787,170 | 79,797,431 | 10,262 | loss | - |
| OPGP255 | F | 11 | 80,740,796 | 80,869,319 | 128,524 | loss | - |
| OPGP72 | F | 11 | 81,544,396 | 81,558,237 | 13,842 | gain | - |
| OPGP-SS52 | M | 11 | 82,267,274 | 82,383,896 | 116,623 | gain | PRCP,C11orf82,RAB30 |
| OPGP268 | F | 11 | 86,990,429 | 87,033,092 | 42,664 | loss | - |
| OPGP-SS72 | F | 11 | 89,457,958 | 89,787,171 | 329,214 | loss | NAALAD2,UBTFL1,CHORDC1 |
| OPGP227 | M | 11 | 89,791,938 | 89,890,660 | 98,723 | loss | - |
| OPGP265 | F | 11 | 91,198,725 | 92,219,783 | 1,021,059 | gain | FAT3 |
| OPGP-SS24 | F | 11 | 101,399,150 | 101,492,312 | 93,163 | gain | C11orf70,YAP1 |
| OPGP-SS111 | M | 11 | 111,549,205 | 111,592,334 | 43,130 | loss | BCO2 |
| SS39_OPGP2 | M | 11 | 117,873,790 | 117,930,629 | 56,840 | gain | TMEM25,IFT46,TTC36,MLL |
| OPGP-SS27 | F | 11 | 134,352,738 | 134,449,982 | 97,245 | loss | - |
| OPGP58 | F | 12 | 30,887 | 104,526 | 73,640 | loss | IQSEC3 |
| OPGP20 | F | 12 | 5,577,633 | 6,102,612 | 524,980 | loss | VWF,ANO2 |
| OPGP-SS15 | F | 12 | 9,344,838 | 9,387,669 | 42,832 | gain | LOC642846 |
| OPGP39 | M | 12 | 30,841,413 | 31,092,984 | 251,572 | gain | TSPAN11 |
| OPGP144 | M | 12 | 32,308,006 | 32,333,520 | 25,515 | gain | BICD1 |
| OPGP147 | M | 12 | 32,448,256 | 32,508,065 | 59,810 | loss | - |
| OPGP-SS111 | M | 12 | 40,300,400 | 40,362,608 | 62,209 | gain | - |
| OPGP127 | F | 12 | 50,799,017 | 50,846,239 | 47,223 | gain | - |
| OPGP22 | F | 12 | 67,739,183 | 67,750,568 | 11,386 | loss | - |
| OPGP163 | M | 12 | 71,730,857 | 71,783,326 | 52,470 | loss | - |
| OPGP-SS13 | F | 12 | 74,329,241 | 74,480,712 | 151,472 | gain | - |
| OPGP202 | F | 12 | 79,156,468 | 79,255,527 | 99,060 | loss | OTOGL |
| OPGP29 | M | 12 | 91,644,061 | 91,848,534 | 204,474 | gain | EEA1,PLEKHG7 |
| OPGP-SS40 | F | 12 | 91,644,061 | 91,845,235 | 201,175 | gain | EEA1,PLEKHG7 |
| OPGP-SS63 | F | 12 | 103,201,466 | 103,224,839 | 23,374 | loss | EID3,TXNRD1 |
| OPGP-SS48 | M | 12 | 125,576,252 | 125,745,397 | 169,146 | gain | - |
| OPGP240 | F | 12 | 127,728,669 | 127,939,053 | 210,385 | gain | SLC15A4,TMEM132C,GLT1D1 |
| OPGP110 | F | 12 | 129,278,320 | 129,772,780 | 494,461 | gain | RIMBP2,PIWIL1 |
| OPGP-SS24 | F | 12 | 130,040,458 | 130,382,165 | 341,708 | gain | LOC116437,GPR133 |
| OPGP33 | F | 12 | 130,044,192 | 130,307,008 | 262,817 | gain | LOC116437,GPR133 |
| OPGP33 | F | 12 | 130,524,700 | 130,674,102 | 149,403 | gain | - |
| OPGP-SS24 | F | 12 | 130,528,580 | 130,674,102 | 145,523 | gain | - |
| OPGP-SS10 | F | 13 | 19,126,655 | 19,225,716 | 99,062 | gain | MPHOSPH8,PSPC1 |
| OPGP106 | F | 13 | 25,189,176 | 25,266,951 | 77,776 | gain | ATP8A2 |
| OPGP16 | M | 13 | 34,433,238 | 34,464,233 | 30,996 | loss | NBEA |
| OPGP-SS85 | F | 13 | 39,533,644 | 39,545,764 | 12,121 | loss | - |
| SS126_OPGP2 | F | 13 | 40,307,515 | 40,374,086 | 66,572 | loss | LOC100616668 |
| OPGP42 | M | 13 | 54,041,427 | 54,374,043 | 332,617 | loss | - |
| OPGP177 | F | 13 | 55,876,886 | 55,906,043 | 29,158 | loss | - |
| OPGP67 | F | 13 | 56,786,597 | 56,943,790 | 157,194 | gain | - |
| OPGP61 | F | 13 | 62,292,555 | 62,322,976 | 30,422 | loss | - |
| OPGP-SS101 | M | 13 | 64,649,291 | 65,321,594 | 672,304 | gain | - |
| OPGP-SS93 | F | 13 | 71,110,420 | 71,128,782 | 18,363 | loss | DACH1 |
| SS87_OPGP2 | M | 13 | 83,479,121 | 83,599,230 | 120,110 | loss | MIR548F1 |
| OPGP-SS13 | F | 13 | 84,707,522 | 84,735,558 | 28,037 | gain | - |
| OPGP53 | F | 13 | 88,056,680 | 88,068,498 | 11,819 | loss | - |
| OPGP217 | M | 13 | 89,780,507 | 89,902,445 | 121,939 | loss | - |
| OPGP76 | F | 13 | 99,921,578 | 100,016,266 | 94,689 | gain | PCCA,A2LD1 |
| OPGP-SS16 | F | 13 | 107,130,075 | 107,283,351 | 153,277 | loss | FAM155A |
| OPGP179 | M | 13 | 113,543,086 | 113,654,849 | 111,764 | gain | FAM70B,GAS6 |
| SS40_OPGP2 | M | 14 | 21,992,078 | 22,048,615 | 56,538 | loss | - |
| OPGP-SS12 | F | 14 | 21,992,078 | 22,025,556 | 33,479 | loss | - |
| SS73_OPGP2 | M | 14 | 26,537,691 | 26,631,461 | 93,771 | loss | - |
| OPGP23 | F | 14 | 27,170,503 | 27,376,515 | 206,013 | gain | - |
| OPGP-SS52 | M | 14 | 30,614,595 | 30,736,869 | 122,275 | gain | AP4S1,HECTD1 |
| OPGP-SS52 | M | 14 | 31,317,416 | 31,373,117 | 55,702 | loss | NUBPL |
| OPGP239 | F | 14 | 34,060,194 | 34,097,259 | 37,066 | loss | EAPP |
| OPGP-SS38 | M | 14 | 38,727,298 | 38,881,982 | 154,685 | gain | CTAGE5,MIA2 |
| OPGP165 | M | 14 | 40,034,310 | 40,114,168 | 79,859 | loss | - |
| OPGP47 | F | 14 | 44,998,959 | 45,027,364 | 28,406 | loss | - |
| SS77_OPGP2 | M | 14 | 45,451,570 | 45,482,761 | 31,192 | gain | - |
| OPGP254 | F | 14 | 46,165,038 | 46,175,925 | 10,888 | loss | - |
| SS55_OPGP2 | F | 14 | 49,033,143 | 49,087,041 | 53,899 | loss | - |
| OPGP27 | M | 14 | 50,085,600 | 50,246,752 | 161,153 | loss | ATL1,SAV1 |
| SS667_OPGP2 | F | 14 | 54,205,175 | 54,227,726 | 22,552 | gain | SAMD4A |
| OPGP-SS04 | M | 14 | 62,587,454 | 62,624,470 | 37,017 | gain | KCNH5 |
| OPGP-SS15 | F | 14 | 66,262,441 | 66,299,550 | 37,110 | loss | GPHN |
| SS126_OPGP2 | F | 14 | 66,605,185 | 66,719,847 | 114,663 | gain | GPHN |
| OPGP97 | F | 14 | 69,969,832 | 70,009,883 | 40,052 | loss | ADAM21,C14orf55 |
| OPGP-SS47 | M | 14 | 70,023,902 | 70,083,000 | 59,099 | gain | ADAM20 |
| OPGP235 | F | 14 | 79,540,616 | 79,556,292 | 15,677 | loss | - |
| SS55_OPGP2 | F | 14 | 86,710,968 | 86,769,895 | 58,928 | loss | - |
| OPGP72 | F | 14 | 99,426,385 | 99,465,902 | 39,518 | gain | EML1 |
| OPGP-SS73 | F | 14 | 106,344,954 | 106,356,482 | 11,529 | gain | - |
| OPGP-SS67 | F | 15 | 21,851,447 | 21,904,021 | 52,575 | loss | - |
| SS33_OPGP2 | M | 15 | 32,374,931 | 32,421,325 | 46,395 | loss | NOP10,SLC12A6 |
| OPGP-SS103 | M | 15 | 36,983,956 | 37,176,174 | 192,219 | gain | - |
| OPGP169 | F | 15 | 53,998,357 | 54,225,066 | 226,710 | gain | RFX7,NEDD4 |
| SS81_OPGP2 | F | 15 | 59,034,061 | 59,050,669 | 16,609 | loss | RORA |
| OPGP286 | M | 15 | 69,022,959 | 69,224,764 | 201,806 | gain | LRRC49,THSD4,CT62 |
| OPGP-SS29 | F | 15 | 75,293,482 | 75,356,376 | 62,895 | loss | C15orf5,PEAK1 |
| OPGP33 | F | 15 | 75,819,762 | 76,127,594 | 307,833 | gain | LOC645752,TBC1D2B,LOC91450 |
| OPGP03 | F | 15 | 79,154,864 | 79,169,669 | 14,806 | loss | - |
| OPGP131 | F | 15 | 79,334,232 | 79,344,892 | 10,661 | loss | IL16 |
| OPGP175 | F | 15 | 82,886,489 | 82,982,104 | 95,616 | gain | SCAND2,LOC100506874,UBE2Q2P1,ZSCAN2 |
| OPGP276 | F | 15 | 83,336,499 | 83,496,203 | 159,705 | loss | PDE8A |
| OPGP-SS58 | F | 15 | 83,688,662 | 83,715,490 | 26,829 | loss | - |
| OPGP168 | M | 15 | 90,565,432 | 90,576,621 | 11,190 | loss | - |
| OPGP-SS116 | M | 15 | 91,740,596 | 91,852,025 | 111,430 | loss | - |
| OPGP143 | M | 15 | 97,167,880 | 97,202,432 | 34,553 | loss | IGF1R |
| SS126_OPGP2 | F | 15 | 97,908,966 | 97,932,653 | 23,688 | loss | MEF2A |
| OPGP266 | F | 15 | 98,510,305 | 98,523,873 | 13,569 | loss | ADAMTS17 |
| OPGP73 | F | 15 | 98,751,652 | 98,906,792 | 155,141 | gain | LASS3 |
| OPGP160 | M | 15 | 99,970,111 | 100,033,288 | 63,178 | loss | TM2D3,TARSL2 |
| OPGP210 | M | 16 | 160,326 | 181,309 | 20,984 | gain | HBA1,HBQ1,HBA2,LUC7L |
| OPGP-SS02 | F | 16 | 4,526,971 | 4,589,721 | 62,751 | loss | C16orf5,LOC342346 |
| OPGP119 | F | 16 | 5,403,914 | 5,419,169 | 15,256 | loss | - |
| OPGP53 | F | 16 | 8,527,689 | 8,545,987 | 18,299 | loss | - |
| OPGP236 | F | 16 | 11,541,247 | 11,558,388 | 17,142 | loss | LITAF |
| OPGP255 | F | 16 | 14,164,051 | 14,189,983 | 25,933 | gain | MKL2 |
| SS76_OPGP2 | M | 16 | 14,446,561 | 14,494,322 | 47,762 | gain | PARN |
| OPGP204 | F | 16 | 46,980,147 | 47,071,325 | 91,179 | gain | - |
| OPGP286 | M | 16 | 54,196,274 | 54,374,206 | 177,933 | gain | CES1P2,SLC6A2,CES1P1 |
| SS125_OPGP2 | F | 16 | 61,976,815 | 61,993,809 | 16,995 | loss | - |
| OPGP-SS03 | F | 16 | 69,977,549 | 70,012,760 | 35,212 | gain | CALB2 |
| OPGP28 | M | 16 | 70,996,201 | 71,100,367 | 104,167 | loss | - |
| OPGP-SS27 | F | 16 | 71,116,930 | 71,131,985 | 15,056 | loss | - |
| OPGP234 | F | 16 | 74,200,364 | 74,212,427 | 12,064 | loss | ADAT1 |
| OPGP121 | F | 16 | 74,524,271 | 74,567,316 | 43,046 | loss | - |
| SS65_OPGP2 | F | 16 | 75,804,807 | 75,879,491 | 74,685 | loss | ADAMTS18 |
| OPGP271 | F | 16 | 79,508,646 | 79,557,426 | 48,781 | loss | - |
| OPGP-SS111 | M | 16 | 80,661,587 | 80,676,273 | 14,687 | loss | HSD17B2 |
| OPGP-SS04 | M | 16 | 81,545,526 | 81,604,208 | 58,683 | loss | CDH13 |
| OPGP156 | M | 16 | 84,802,018 | 84,814,714 | 12,697 | loss | - |
| OPGP06 | M | 16 | 84,837,516 | 84,867,945 | 30,430 | loss | - |
| OPGP-SS110 | M | 16 | 85,030,259 | 85,049,728 | 19,470 | gain | - |
| SS127_OPGP2 | F | 16 | 86,322,658 | 86,340,352 | 17,695 | loss | KLHDC4 |
| OPGP184 | F | 17 | 514,903 | 534,011 | 19,109 | loss | VPS53 |
| OPGP-SS115 | M | 17 | 659,882 | 1,055,525 | 395,644 | loss | ABR,NXN,MIR3183,TIMM22 |
| OPGP114 | F | 17 | 15,810,282 | 16,058,587 | 248,306 | gain | ADORA2B,ZSWIM7,TTC19,NCOR1 |
| OPGP-SS48 | M | 17 | 19,911,470 | 19,926,019 | 14,550 | loss | - |
| OPGP-SS28 | F | 17 | 35,306,091 | 35,439,280 | 133,190 | gain | ORMDL3,GSDMA,GSDMB,MED24,PSMD3,CSF3,LRRC3C,SNORD124 |
| OPGP246 | M | 17 | 45,187,116 | 45,212,702 | 25,587 | loss | FAM117A |
| OPGP41 | M | 17 | 49,286,499 | 49,375,343 | 88,845 | loss | - |
| OPGP20 | F | 17 | 50,229,035 | 50,249,835 | 20,801 | gain | - |
| OPGP149 | M | 17 | 71,898,039 | 71,934,237 | 36,199 | gain | UBE2O |
| OPGP249 | M | 18 | 160,441 | 210,599 | 50,159 | gain | THOC1,USP14 |
| OPGP-SS88 | F | 18 | 311,430 | 332,512 | 21,083 | loss | COLEC12 |
| OPGP116 | F | 18 | 2,974,448 | 2,988,950 | 14,503 | loss | LPIN2 |
| SS37_OPGP2 | M | 18 | 4,404,354 | 4,641,535 | 237,182 | gain | - |
| OPGP247 | M | 18 | 7,213,705 | 7,427,868 | 214,164 | gain | LRRC30 |
| OPGP23 | F | 18 | 7,997,252 | 8,015,176 | 17,925 | loss | PTPRM |
| OPGP67 | F | 18 | 12,510,514 | 12,523,241 | 12,728 | loss | SPIRE1 |
| OPGP165 | M | 18 | 16,783,351 | 16,878,247 | 94,897 | gain | ROCK1 |
| OPGP67 | F | 18 | 21,754,916 | 21,790,936 | 36,021 | loss | - |
| OPGP-SS77 | M | 18 | 23,510,400 | 23,916,848 | 406,449 | gain | CDH2 |
| OPGP187 | M | 18 | 27,991,040 | 28,018,557 | 27,518 | gain | - |
| OPGP-SS20 | M | 18 | 32,312,013 | 32,331,664 | 19,652 | loss | FHOD3 |
| SS75_OPGP2 | M | 18 | 33,521,263 | 33,531,846 | 10,584 | loss | - |
| OPGP148 | M | 18 | 47,146,846 | 47,209,177 | 62,332 | gain | - |
| OPGP114 | F | 18 | 53,047,911 | 53,074,975 | 27,065 | loss | - |
| OPGP132 | F | 18 | 65,832,070 | 66,021,008 | 188,939 | loss | RTTN |
| OPGP22 | F | 18 | 69,281,483 | 69,311,825 | 30,343 | loss | - |
| OPGP204 | F | 18 | 72,565,690 | 72,600,290 | 34,601 | loss | - |
| SS87_OPGP2 | M | 18 | 73,453,193 | 73,510,172 | 56,980 | loss | - |
| OPGP-SS114 | M | 19 | 18,622,276 | 18,653,925 | 31,650 | gain | KLHL26 |
| OPGP161 | M | 19 | 20,133,203 | 20,162,646 | 29,444 | gain | ZNF486 |
| OPGP52 | F | 19 | 23,781,659 | 23,892,043 | 110,385 | gain | RPSAP58,ZNF726 |
| OPGP94 | M | 19 | 33,790,946 | 33,816,349 | 25,404 | loss | - |
| OPGP179 | M | 19 | 34,619,105 | 34,690,833 | 71,729 | gain | - |
| OPGP106 | F | 19 | 47,781,613 | 47,867,134 | 85,522 | loss | CEACAM8 |
| OPGP280 | M | 19 | 55,816,543 | 55,888,141 | 71,599 | gain | LOC342918,SYT3,SHANK1 |
| OPGP-SS33 | F | 19 | 58,556,242 | 58,588,445 | 32,204 | gain | ZNF525 |
| OPGP283 | M | 19 | 59,520,820 | 59,545,647 | 24,828 | loss | LILRA4 |
| OPGP06 | M | 20 | 808,802 | 1,099,029 | 290,228 | gain | PSMF1,ANGPT4,RSPO4 |
| OPGP-SS03 | F | 20 | 5,882,566 | 5,998,478 | 115,913 | gain | CRLS1,LRRN4,MCM8 |
| SS131_OPGP2 | F | 20 | 6,096,050 | 6,112,780 | 16,731 | loss | - |
| OPGP258 | M | 20 | 6,359,203 | 6,369,380 | 10,178 | gain | - |
| OPGP-SS105 | M | 20 | 7,588,629 | 7,618,194 | 29,566 | loss | - |
| OPGP21 | F | 20 | 8,354,002 | 8,364,213 | 10,212 | loss | PLCB1 |
| OPGP163 | M | 20 | 8,583,927 | 8,850,689 | 266,763 | gain | PLCB1 |
| OPGP14 | M | 20 | 23,193,270 | 23,220,469 | 27,200 | gain | - |
| OPGP-SS40 | F | 20 | 25,116,159 | 25,150,508 | 34,350 | loss | ENTPD6 |
| OPGP68 | M | 20 | 29,298,710 | 29,446,231 | 147,522 | gain | DEFB116,DEFB115,DEFB118,DEFB119 |
| OPGP-SS19 | M | 20 | 29,334,284 | 29,359,773 | 25,490 | loss | DEFB116 |
| OPGP-SS116 | M | 20 | 42,279,080 | 42,296,207 | 17,128 | loss | LOC100505783 |
| OPGP35 | F | 20 | 44,909,981 | 44,920,224 | 10,244 | loss | - |
| OPGP101 | M | 20 | 48,265,623 | 48,284,599 | 18,977 | gain | - |
| OPGP-SS55 | F | 21 | 17,738,094 | 18,003,248 | 265,155 | gain | C21orf37,BTG3,CXADR |
| OPGP202 | F | 21 | 20,190,121 | 20,374,791 | 184,671 | loss | - |
| OPGP244 | M | 21 | 21,493,932 | 21,545,328 | 51,397 | loss | NCAM2 |
| SS60_OPGP2 | F | 21 | 22,534,973 | 22,573,005 | 38,033 | loss | - |
| SS82_OPGP2 | M | 21 | 23,169,797 | 23,220,091 | 50,295 | loss | - |
| SS120_OPGP2 | F | 21 | 23,196,562 | 23,232,190 | 35,629 | loss | - |
| SS127_OPGP2 | F | 21 | 34,230,168 | 34,268,175 | 38,008 | loss | - |
| OPGP262 | F | 21 | 37,320,548 | 37,401,155 | 80,608 | gain | PIGP,TTC3 |
| OPGP-SS105 | M | 21 | 43,014,698 | 43,025,706 | 11,009 | gain | PDE9A |
| OPGP-SS07 | M | 22 | 16,783,629 | 16,875,947 | 92,319 | gain | MICAL3 |
| OPGP195 | M | 22 | 18,138,816 | 18,251,778 | 112,963 | loss | GNB1L,TBX1,TXNRD2,C22orf29 |
| OPGP65 | F | 22 | 22,475,809 | 22,613,015 | 137,207 | gain | DERL3,SMARCB1,SLC2A11,MIF |
| OPGP285 | M | 22 | 22,501,305 | 22,601,745 | 100,441 | gain | DERL3,SMARCB1,SLC2A11,MIF |
| OPGP272 | F | 22 | 28,616,197 | 28,678,686 | 62,490 | loss | MTMR3 |
| OPGP-SS82 | M | 22 | 30,259,132 | 30,274,347 | 15,216 | loss | SFI1 |
| OPGP159 | M | 22 | 30,689,003 | 30,794,362 | 105,360 | gain | SLC5A1 |
| OPGP212 | M | 22 | 30,798,490 | 30,856,639 | 58,150 | gain | SLC5A1 |
| OPGP-SS81 | F | 22 | 34,879,640 | 34,900,774 | 21,135 | gain | APOL3 |
| OPGP-SS17 | F | 22 | 35,500,861 | 35,512,627 | 11,767 | loss | IFT27 |
| OPGP08 | M | 22 | 46,835,442 | 46,857,005 | 21,564 | gain | - |
| OPGP199 | F | 22 | 47,529,950 | 47,981,564 | 451,615 | gain | FAM19A5 |
| OPGP-SS46 | M | 22 | 47,653,943 | 47,733,961 | 80,019 | gain | - |
| OPGP26 | M | X | 12,999,157 | 13,522,982 | 523,826 | gain | EGFL6,ATXN3L |
| OPGP58 | F | X | 25,390,656 | 25,412,613 | 21,958 | gain | - |
| SS130_OPGP2 | M | X | 44,319,204 | 44,539,899 | 220,696 | gain | - |
| SS130_OPGP2 | M | X | 44,595,756 | 44,635,864 | 40,109 | gain | KDM6A |
| OPGP144 | M | X | 48,498,134 | 48,577,719 | 79,586 | gain | GLOD5,PCSK1N,HDAC6,ERAS,GATA1 |
| OPGP-SS20 | M | X | 49,710,632 | 49,784,469 | 73,838 | gain | CLCN5 |
| OPGP-SS55 | F | X | 50,670,424 | 50,707,441 | 37,018 | loss | BMP15 |
| OPGP175 | F | X | 56,538,120 | 56,573,034 | 34,915 | gain | - |
| OPGP128 | F | X | 63,670,283 | 63,707,868 | 37,586 | loss | - |
| OPGP44 | F | X | 64,995,259 | 65,872,670 | 877,412 | gain | MIR223,EDA2R,VSIG4, HEPH |
| OPGP-SS38 | M | X | 75,394,779 | 75,509,158 | 114,380 | gain | - |
| OPGP-SS25 | F | X | 81,040,163 | 81,069,627 | 29,465 | loss | - |
| OPGP124 | F | X | 82,012,720 | 82,039,876 | 27,157 | loss | - |
| OPGP61 | F | X | 82,656,149 | 82,722,076 | 65,928 | gain | - |
| SS83_OPGP2 | M | X | 89,138,362 | 89,197,179 | 58,818 | gain | - |
| OPGP28 | M | X | 93,626,701 | 94,309,316 | 682,616 | loss | - |
| OPGP81 | M | X | 101,631,239 | 101,687,961 | 56,723 | gain | TMSB15A |
| OPGP105 | F | X | 101,922,254 | 101,969,255 | 47,002 | loss | - |
| SS126_OPGP2 | F | X | 113,869,101 | 114,000,018 | 130,918 | gain | MIR1911,MIR448,HTR2C |
| OPGP143 | M | X | 114,923,347 | 114,942,982 | 19,636 | gain | - |
| OPGP223 | M | X | 114,923,347 | 114,942,941 | 19,595 | gain | - |
| SS57_OPGP2 | F | X | 116,752,039 | 116,775,440 | 23,402 | gain | - |
| OPGP-SS78 | F | X | 124,851,617 | 124,965,364 | 113,748 | loss | - |
| OPGP01 | F | X | 125,025,764 | 125,134,215 | 108,452 | loss | DCAF12L2 |
| OPGP-SS10 | F | X | 125,766,571 | 125,792,783 | 26,213 | loss | CXorf64 |
| OPGP-SS10 | F | X | 125,972,787 | 126,181,968 | 209,182 | loss | - |
| SS31_OPGP2 | M | X | 126,937,581 | 126,960,956 | 23,376 | gain | - |
| OPGP65 | F | X | 127,143,627 | 127,213,250 | 69,624 | loss | - |
| OPGP-SS63 | F | X | 128,562,880 | 128,583,596 | 20,717 | gain | - |
| OPGP95 | M | X | 138,929,827 | 138,981,888 | 52,062 | gain | - |
| OPGP95 | M | X | 139,128,282 | 139,309,755 | 181,474 | gain | - |
| OPGP-SS68 | F | X | 142,130,638 | 142,230,480 | 99,843 | gain | - |
| OPGP143 | M | X | 146,745,860 | 146,756,080 | 10,221 | loss | - |
| OPGP-SS53 | F | X | 149,555,906 | 149,661,552 | 105,647 | gain | MTM1,MTMR1 |
| OPGP101 | M | X | 149,962,751 | 149,999,642 | 36,892 | gain | - |
| OPGP-SS119 | F | X | 153,702,021 | 153,780,822 | 78,802 | gain | F8,LOC100132963,F8A3,F8A2,H2AFB3,H2AFB1,F8A1,MIR1184-1,MIR1184-2,MIR1184-3 |
| SS667_OPGP2 | F | X | 154,235,666 | 154,835,548 | 599,883 | gain | VAMP7,F8A1,F8A3,F8A2,H2AFB3,H2AFB2,H2AFB1,SPRY3,MIR1184-1,MIR1184-2,MIR1184-3,TMLHE |

**Supplementary Table 3: Summary of Affymetrix 6.0 microarray CNV data TOF sample (N=340)**

|  | **All (n=340) Caucasian probands** | | | | | | |
| --- | --- | --- | --- | --- | --- | --- | --- |
|  | **Non-genic** | | | **Genic** | | | **All** |
|  | **Gain** | **Loss** | **Mixed** | **Gain** | **Loss** | **Mixed** |
| Total number of CNVs for all samples with results | 2562 | 9650 | 54 | 2984 | 5884 | 214 | 21348 |
| % of total number of stringent CNVs | 12.00 | 45.20 | 0.25 | 13.98 | 27.56 | 1.00 | 100 |
| Average number of CNVs per genome^2^ (N=340) | 7.54 | 28.38 | 0.16 | 8.78 | 17.31 | 0.63 | 62.79 |
| Median size in bp (Range) | 23,499 (1,134-1,734,110) | 10,156 (397-16,40,265) | 111,225 (2,280-112,889) | 81,360 (1,366-5,454,818) | 21,735 (937-5,997,249) | 83,941 (4,390-1,852,049) | 18,020 (397-5,997,249) |
| Median size in bp (Range), for rare (0%) CNVs | 50,910 (5,886-825,415) | 22,024 (1,441-366,439) | 0 | 99,188 (6,867-5,454,818) | 52,117 (3,259-5,997,249) | 0 | 45,490.5 (1,441-5,997,249) |
| Number of >100kb CNVs | 354 | 493 | 34 | 1388 | 1285 | 104 | 3658 |
| % of total (entry in first row in each column) | 13.82 | 5.11 | 62.96 | 46.51 | 21.84 | 48.60 | 17.14 |
| Number of rare CNVs | 112 | 199 | 0 | 147 | 164 | 0 | 622 |
| % of total (entry in first row in each column) | 4.37 | 2.06 | 0.00 | 4.93 | 2.79 | 0.00 | 2.91 |
| Average number of rare CNVs per genome^3^ (include subjects with no rare CNV, N=340) | 0.33 | 0.59 | 0.00 | 0.43 | 0.48 | 0.00 | 1.83 |
| Average number of rare CNVs per genome^4^ (only includes subjects with 1 or more rare CNVs, N=274) | 0.41 | 0.73 | 0.00 | 0.54 | 0.60 | 0.00 | 2.27 |
| Number of subjects with rare CNVs in each category, see column heading | 97 | 146 | 0 | 117 | 129 | 0 | 274 |
| % of number of subjects with rare CNVs | 28.53 | 42.94 | 0.00 | 34.41 | 37.94 | 0.00 | 80.59 |

This table accounts for stringent CNVs <6.5Mb; no restriction on chromosome (i.e., includes X chromosome)

^1^ “All” category: Genic and Non-genic, Gain, Loss and Mixed CNVs all together

^2^ Average number of CNVs per genome= total number of CNVs in each column divided by total number of subjects in each category

^3^ Average number of rare CNVs per genome= total number of rare CNVs in each column divided by the total number of subjects (include subjects with no rare CNV)

^4^ Average number of rare CNVs per genome = total number of rare CNVs in each column divided by the total number of subjects (only include subjects with 1 or more rare CNVs)

**Supplementary Table 4: Rare large CNVs (>500 kb) in 43 of 433 unrelated adults with tetralogy of Fallot**

| **Case** | **CNV characteristics** | | | | | | | | | | |
| --- | --- | --- | --- | --- | --- | --- | --- | --- | --- | --- | --- |
|  | **Locus** | **Start** | **Size (bp)** | **CN** | **Very rare** | **Confirmed** | **Origin** | **Seg dup(s)** | **# of genes** | **Candidate gene(s)** | **References** |
| 1 | 1p34.3 | 35,138,921 | 619,938 | Loss | ⚫ | ⚫ |  |  | 6 | **SFPQ** |  |
| 2 | 1q21.1 | 143,590,972 | 3,981,720 | ^a^Gain | ⚫ | ⚫ | *de novo* | ⚫ | 45 | ^b,c^**GJA5** | [2,3,4,5] |
| 3 | 1q21.1 | 144,643,825 | 1,630,074 | ^a^Gain | ⚫ | ⚫ |  | ⚫ | 14 |  |  |
| 4 | 1q21.1 | 144,643,825 | 1,667,590 | ^a^Gain | ⚫ | ⚫ |  | ⚫ | 14 |  |  |
| 5 | 1q21.1 | 144,643,825 | 1,752,136 | ^a^Gain | ⚫ | ⚫ |  | ⚫ | 16 |  |  |
| 6 | 1q41 | 220,214,502 | 1,051,264 | Loss | ⚫ | ⚫ |  |  | 7 | **DISP1** | [6,7,8,9] |
| ^d^7 | 2p23.2-p23.1 | 29,418,234 | 1,569,210 | Gain | ⚫ | ⚫ |  |  | 6 | **LBH** |  |
| ^d^8 | 2q11.2 | 97,982,662 | 548,388 | Gain | ⚫ | ⚫ |  |  | 3 |  |  |
| 9 | 2q21.1 | 131,194,418 | 826,998 | Loss | ⚫ | ⚫ |  | ⚫ | 13 | **ARHGEF4** |  |
| 10 | 2q24.1 | 154,772,039 | 1,978,861 | Gain | ⚫ | ⚫ | Inherited |  | 2 |  |  |
| 11 | 2q32.1 | 188,273,007 | 700,865 | Loss | ⚫ | ⚫ |  |  | 2 |  |  |
| 12 | 3p26.3 | 60,002 | 995,032 | Gain | ⚫ | ⚫ |  | ⚫ | 1 | **CHL1** |  |
| 13 | 4p15.1 | 28,835,868 | 5,997,249 | Loss | ⚫ | ⚫ |  |  | 1 |  |  |
| 14 | 4q35.2 | 188,923,641 | 514,206 | Loss | ⚫ | ⚫ |  |  | 3 |  |  |
| 10 | 5q14.1-q14.3 | 80,665,064 | 5,454,818 | Gain | ⚫ | ⚫ | Inherited |  | 13 | **EDIL3,** ^c^**VCAN** | [14,15,16,17,18,19,20,21,22,23,24] |
| 15 | 5q22.1 | 109,622,167 | 611,666 | Gain | ⚫ | ⚫ |  |  | 2 | **SLC25A46** |  |
| 16 | 5q23.3-q31.1 | 130,124,159 | 811,158 | Gain | ⚫ | ⚫ |  |  | 4 |  |  |
| 17 | 6p22.3-p22.2 | 21,587,649 | 2,098,900 | Gain | ⚫ | ⚫ |  |  | 4 | ^c^**SOX4** | [17,26,27,28] |
| 18 | 6q11.1 | 61,987,979 | 991,555 | Gain | ⚫ | ⚫ | Inherited |  | 1 |  |  |
| 19 | 6q14.1 | 81,166,965 | 760,841 | Gain | ⚫ | ND |  |  | 0 |  |  |
| 20 | 7p11.2-p11.1 | 57,264,874 | 645,786 | Gain |  | ND |  | ⚫ | 1 |  |  |
| ^d^21 | 8p23.2 | 3,778,626 | 2,159,631 | Gain | ⚫ | ⚫ |  |  | 1 |  |  |
| ^d^22 | 8p23.2-p23.1 | 6,187,821 | 1,037,609 | Loss | ⚫ | ⚫ |  | ⚫ | 20 | ^b^**ANGPT2** | [29,30,31] |
| 23 | 8q11.1-q11.21 | 47,203,026 | 898,799 | Gain | ⚫ | ND |  |  | 1 |  |  |
| ^d^24 | 9p13.1-p12 | 38,951,514 | 1,658,247 | Gain |  | ND |  | ⚫ | 6 |  |  |
| 25 | 10q11.1-q11.21 | 41,941,187 | 752,815 | Gain |  | ND |  | ⚫ | 5 |  |  |
| 26 | 10q26.13 | 125,510,180 | 743,304 | Gain | ⚫ | ⚫ |  |  | 5 |  |  |
| 27 | 11q22.3 | 103,542,068 | 1,252,192 | Gain |  | ND |  |  | 7 | **CASP1, CASP4, CASP5, CASP12** | [32,33,34] |
| ^d^28 | 12p11.1 | 33,432,314 | 575,409 | Loss | ⚫ | ⚫ |  | ⚫ | 1 |  |  |
| **Case** | **CNV characteristics** | | | | | | | | | | |
|  | **Locus** | **CNV start** | **CNV size** | **CN** | **Very rare** | **Confirmed** | **Origin** | **Seg dup(s)** | **# of genes** | **Candidate gene(s)** | **References** |
| 29 | 12q24.32 | 125,358,126 | 793,244 | Loss | ⚫ | ⚫ | Inherited |  | 1 |  |  |
| ^d^30 | 13q12.3-q13.3 | 31,086,497 | 4,726,890 | ^f^Loss | ⚫ | ⚫ | *de novo* |  | 19 | ^b^**NBEA, PDS5B, SPG20** | [35,36,37,38,39,40] |
| ^d^31 | 13q14.11 | 42,009,354 | 555,346 | Gain | ⚫ | ⚫ |  |  | 4 | **TNFSF11** |  |
| 32 | 13q33.1-13q33.2 | 103,096,790 | 825,415 | Gain | ⚫ | ND | Inherited |  | 0 |  |  |
| 33 | 16p11.2 | 29,487,535 | 597,774 | Gain | ⚫ | ⚫ |  |  | 27 | **HIRIP3,** ^c^**MAPK3, PPP4C,** ^c^**TBX6** | [42,43,44,45,46,47,48,49,50] |
| 34 | 17p13.3 | 366,915 | 543,945 | Gain |  | ND |  |  | 9 | **NXN** |  |
| 35 | 17p13.2 | 5,528,898 | 667,804 | Loss | ⚫ | ⚫ |  |  | 1 |  |  |
| 36 | 17p11.2 | 18,296,117 | 695,535 | Gain |  | ND |  |  | 15 | **FOXO3B** |  |
| 32 | 17q12 | 31,610,407 | 1,942,495 | Gain |  | ND | *de novo* |  | 25 | **HNF1B** |  |
| 37 | 18q22.3-18q23 | 69,207,652 | 3,406,071 | ^g^Gain | ⚫ | ⚫ |  |  | 13 | **CNDP2** |  |
| 37 | 18q23 | 72,614,319 | 3,501,711 | ^g^Loss | ⚫ | ⚫ |  |  | 15 | ^c^**NFATC1, PARD6G** | [41,55,56,57,58,59,60,61,62] |
| 38 | 19p13.3 | 183,496 | 805,727 | Loss | ⚫ | ⚫ |  |  | 34 | **CNN2, FSTL3, PTBP1, WDR18** | [63,64,65,66,67,68,69,70] |
| ^d^39 | 19p13.3 | 2,500,048 | 708,368 | Gain | ⚫ | ⚫ |  |  | 18 | **GNA11, S1PR4** | [71,72,73] |
| ^d^40 | 19q13.41 | 57,807,872 | 629,678 | Gain | ⚫ | ⚫ |  | ⚫ | 14 | **ZNF347** |  |
| 41 | 21q21.1 | 20,282,405 | 560,377 | Gain | ⚫ | ⚫ |  |  | 0 |  |  |
| 42 | 21q21.3 | 29,607,482 | 637,303 | Loss | ⚫ | ⚫ | Inherited |  | 3 |  |  |
| 41 | 22q11.21 | 17,190,110 | 2,174,700 | Gain |  | ⚫ |  | ⚫ | 44 | **CRKL, HIRA, TBX1** | [75,76,77,78,79,80,81] |
| 43 | Xp22.2 | 13,788,639 | 615,940 | Gain | ⚫ | ⚫ |  |  | 2 |  |  |

Case, subjects from discovery sample (n=433) with TOF; Locus, cytogenetic location of CNV; CNV start, hg18 (NCBI Build 36.1, March 2006); CNV size, in base pairs; CN, type of copy number aberration; Very rare, not found in 2,773 controls (⚫), see text for details; Confirmed, by qPCR and/or FISH (⚫) or not done (ND); Origin, *de novo* or inherited (where known); Seg dup(s), known flanking segmental duplication(s) (from the UCSC Genome Browser hg18 version) that cover at least 20% of the CNV length (⚫); # of genes, number of known genes overlapped by CNV as annotated in the Database of Genomic Variants (<http://projects.tcag.ca/variation/>; September 2011); Candidate gene(s), selected based on reported cardiovascular system involvement; References, derived from systematic searches of human (e.g., Online Mendelian Inheritance in Man; <http://www.omim.org/>) and model organism (e.g., Mouse Genome Informatics; <http://www.informatics.jax.org/>) databases.

^a^Supplementary Figure 2

^b^Table 3

^c^Neighbor of a top disease gene (GATA4, NKX2-5, TBX5), as identified in the pathway analysis (Supplementary Table 11)

^d^Non-European ancestry

^f^Previously reported by our group

^g^Supplementary Figure 3

**Supplementary Table 5: Very rare CNVs overlapping 26 candidate genes for tetralogy of Fallot**

|  | **Candidate gene**^a^ | **Locus** | **Case** | **CNV start** | **CNV size** | | **CN** | | **Exonic** | **Confirmed** | **Known CV involvement** | **Known structural CV phenotype** | **References** |
| --- | --- | --- | --- | --- | --- | --- | --- | --- | --- | --- | --- | --- | --- |
| Candidate genes for TOF implicated through narrowing of critical regions | **GJA5** | 1q21.1 | 44 | 145,700,719 | 10,347 | | ^b^Gain | |  | ⚫ | ⚫ | ⚫ | [2,3,4,5] |
|  |  |  | Cases 2 to 5: recurrent 1q21.1 duplications (Table 2) | | | | | | | |  |  |  |
|  | **RAF1** | 3p25.1 | 45 | 12,665,657 | 17,184 | | Loss | | ⚫ | ⚫ | ⚫ | ⚫ | [2,82,83,84,85] |
|  |  |  | Probands 756 and 419 from another study^c^ | | | | | | | |  |  |  |
|  | **ANGPT2** | 8p23.1 | 46 | 6,342,621 | 59,662 | | Loss | | ⚫ | ⚫ | ⚫ | ⚫ | [29,30,31] |
|  |  |  | Case 22 (Table 2) | | | | | | | |  |  |  |
|  | **NBEA** | 13q13.3 | 47 | 34,960,143 | 20,620 | | Loss | |  | ND | ⚫ |  |  |
|  |  |  | Case 30 (Table 2) | | | | | | | |  |  |  |
|  | **CDH19** | 18q22.1 | 48 | 62,118,826 | 254,033 | | ^d^Loss | | ⚫ | ⚫ | ⚫ |  | [86,87,88,89] |
|  |  |  | 18q22 deletion syndrome (excluded case; see text) | | | | | | | |  |  |  |
| Candidate genes for TOF implicated by overlapping CNVs | **PLXNA2** | 1q32.2 | 42 | 206,337,728 | 61,546 | | ^e^Loss | | ⚫ | ⚫ | ⚫ | ⚫ | [90,91,92] |
|  |  |  | 49 | 206,369,712 | 10,222 | | ^e^Loss | |  | ⚫ |  |  |  |
|  | **CHRM3** | 1q43 | 50 | 237,983,869 | 381,637 | | Gain | | ⚫ | ND | ⚫ |  | [93,94,95,96] |
|  |  |  | 51 | 238,098,745 | 10,568 | | Loss | |  | ND |  |  |  |
|  | **CCDC148** | 2q24.1 | 52 | 158,961,179 | 51,207 | | Loss | |  | ND |  |  |  |
|  |  |  | 53 | 158,961,179 | 51,207 | | Loss | |  | ND |  |  |  |
|  | **BARD1** | 2q35 | 54 | 215,266,200 | 85,667 | | Loss | | ⚫ | ND | ⚫ |  |  |
|  |  |  | 55 | 215,321,117 | 59,317 | | Loss | | ⚫ | ND |  |  |  |
|  | **PPM1K** | 4q22.1 | 56 | 89,393,217 | 55,637 | | Gain | | ⚫ | ND | ⚫ | ⚫ |  |
|  |  |  | Proband 2231 from another study^c^ | | | | | | | |  |  |  |
|  | **FSTL4** | 5q31.1 | 57 | 132,946,021 | | 15,007 | | Loss |  | ND | ⚫ |  |  |
|  |  |  | 58 | 132,953,135 | | 10,498 | | Loss |  | ND |  |  |  |
|  | **GMDS** | 6p25.3 | 56 | 1,938,284 | | 12,749 | | Loss |  | ⚫ | ⚫ |  |  |
|  |  |  | 59 | 1,939,891 | | 11,142 | | Loss |  | ⚫ |  |  |  |
|  | **ARHGEF10** | 8p23.3 | 60 | 1,716,624 | | 156,826 | | Gain | ⚫ | ND | ⚫ |  |  |
|  |  |  | 61 | 1,736,348 | | 25,900 | | Loss | ⚫ | ND |  |  |  |
|  | **Candidate gene**^a^ | **Locus** | **Case** | **CNV start** | | **CNV size** | | **CN** | **Exonic** | **Confirmed** | **CV involvement** | **Structural CV phenotype** | **References** |
| Candidate genes for TOF implicated by overlapping CNVs (continued) | **KCNB2** | 8q13.3 | 62 | 73,871,523 | | 78,480 | | Gain |  | ND | ⚫ |  |  |
|  |  |  | 63 | 73,885,905 | | 124,789 | | Gain |  | ND |  |  |  |
|  | **C12orf66** | 12q14.2 | 20 | 62,843,704 | | 78,289 | | Gain | ⚫ | ND |  |  |  |
|  |  |  | 64 | 62,843,704 | | 78,289 | | Gain | ⚫ | ND |  |  |  |
|  | **KIAA1609** | 16q24.1 | 65 | 83,066,869 | | 16,053 | | Loss | ⚫ | ND |  |  |  |
|  |  |  | 66 | 83,094,514 | | 26,382 | | Loss | ⚫ | ND |  |  |  |
|  | **CHST8** | 19q13.11 | 67 | 38,902,591 | | 26,593 | | Gain |  | ND | ⚫ |  |  |
|  |  |  | 68 | 38,902,591 | | 23,819 | | Gain |  | ND |  |  |  |
|  | **CECR5** | 22q11.1 | 69 | 15,996,510 | | 18,980 | | Loss | ⚫ | ND | ⚫ |  |  |
|  |  |  | 47 | 16,014,050 | | 84,862 | | Loss | ⚫ | ND |  |  |  |
|  | **WNK3** | Xp11.22 | 70 | 54,325,120 | | 147,676 | | Gain | ⚫ | ND | ⚫ |  |  |
|  |  |  | 32 | 54,374,737 | | 50,162 | | Gain | ⚫ | ND |  |  |  |
|  | **IDS** | Xq28 | 71 | 148,308,784 | | 153,622 | | Gain | ⚫ | ND | ⚫ |  |  |
|  |  |  | 72 | 148,377,523 | | 250,837 | | Gain | ⚫ | ND |  |  |  |
| Selected candidate genes for TOF implicated by singleton CNVs | **FGF10** | 5p12 | 73 | 44,314,920 | | 135,646 | | Loss | ⚫ | ⚫ | ⚫ | ⚫ | [111,112,113] |
|  |  |  | Associated with TBX1 and FGF3 (see text) | | | | | | | |  |  |  |
|  | **SNX8** | 7p22.2 | 74 | 2,310,838 | 180,068 | | Loss | | ⚫ | ND | ⚫ |  |  |
|  |  |  | Within 7p22 deletion syndrome region (see text) | | | | | | | |  |  |  |
|  | **DNAH11** | 7p15.3 | 75 | 21,570,799 | 179,867 | | Loss | | ⚫ | ND | ⚫ | ⚫ | [116,117,118,119,120] |
|  |  |  | Associated with primary ciliary dyskinesis (see text) | | | | | | | |  |  |  |
|  | **BBS9** | 7p14.3 | 76 | 33,293,709 | 259,054 | | Loss | | ⚫ | ⚫ | ⚫ |  | [121,122,123] |
|  |  |  | Associated with Bardet-Biedl syndrome (see text) | | | | | | | |  |  |  |
|  | **SEMA3E** | 7q21.11 | 77 | 82,968,279 | 52,328 | | Loss | | ⚫ | ⚫ | ⚫ | ⚫ | [124,125,126,127] |
|  |  |  | Associated with PLXNA2 (see text) | | | | | | | |  |  |  |
|  | **SEMA3D** | 7q21.11 | 78 | 84,477,068 | 9,591 | | Loss | |  | ⚫ | ⚫ | ⚫ |  |
|  |  |  | Associated with PLXNA2 (see text) | | | | | | | |  |  |  |

Candidate gene, official HGNC symbol; Locus, cytogenetic location of candidate gene; Case, subjects from discovery sample (n=433) with TOF; CNV start, hg18 (NCBI Build 36.1, March 2006); CNV size, in base pairs; CN, type of copy number aberration; Exonic, CNV overlaps exon of candidate gene (⚫); Confirmed, by qPCR (⚫) or not done (ND); CV involvement, known cardiovascular system involvement (⚫; not necessarily in human); Structural CV phenotype, known structural cardiovascular system phenotype associated with mutation (⚫; not necessarily in human); References, derived from systematic searches of human (e.g., Online Mendelian Inheritance in Man; [www.omim.org/](http://www.omim.org/)) and model organism (e.g., Mouse Genome Informatics; <http://www.informatics.jax.org/>) databases.

^a^Novel and previously proposed candidate genes for TOF identified because of overlap with two or more CNVs in unrelated subjects (at least one Caucasian) with TOF, where the CNVs were not observed in 2,773 controls (see text). We have also shown selected candidate genes overlapped by very rare singleton CNVs in our cohort, including all those that overlapped rare CNVs reported by Greenway et al.

^b^Supplementary Figure 2

^c^Greenway et al.

^d^Supplementary Figure 3

^e^Figure 1

**Supplementary table 6: *PLXNA2* sequence variants detected in 192 unrelated TOF and/or pulmonary atresia cases of European ancestry.**

| **Position (hg18)** | **Type^1^** | **cDNA residue^2^** | **#AA^3^** | #**AB^3^** | #**BB^3^** | **Cases MAF^4^** | **dbSNP id** | **dbSNP MAF^4^** | **NHLBI MAF^5^** | **Exon** | **Amino acid change** | **Protein domain** | **SIFT** | **POLYPHEN** |
| --- | --- | --- | --- | --- | --- | --- | --- | --- | --- | --- | --- | --- | --- | --- |
| chr1:206,457,877 | mis | 14(G>A) | 81 | 88 | 23 | 0.349 | rs2782948 | 0.224 – 0.512 | 0.311 | 1 | R5Q |  | damaging | benign |
| chr1:206,457,721 | mis | 170(A>G) | 172 | 20 | 0 | 0.052 | rs11119014 | 0.022 – 0.182 | 0.067 | 1 | Q57R | SEMA domain | tolerated | benign |
| chr1:206,457,708 | syn | 183(C>T) | 134 | 52 | 6 | 0.167 | rs12120681 | 0.003 – 0.186 | 0.165 | 1 |  | SEMA domain |  |  |
| chr1:206,457,650 | mis | 241(G>T) | 191 | 1 | 0 | 0.003 | rs79601528 | 0.003 | 0.005 | 1 | V81L | SEMA domain | tolerated | benign |
| chr1:206,457,092 | mis | 799(G>A) | 111 | 71 | 10 | 0.237 | rs3748735 | 0.051 – 0.229 | 0.198 | 1 | A267T | SEMA domain | tolerated | benign |
| chr1:206,456,785 | mis | 1106(A>G) | 139 | 48 | 5 | 0.151 | rs4844658 | 0 – 0.233 | 0.190 | 1 | E369G | SEMA domain | damaging | benign |
| chr1:206,456,778 | syn | 1113(G>A) | 136 | 50 | 6 | 0.161 | rs4844657 | 0.022 – 0.236 | 0.190 | 1 |  | SEMA domain |  |  |
| chr1:206,456,709 | syn | 1182(C>G) | 79 | 88 | 25 | 0.359 | rs1664227 | 0.310 – 0.500 | 0.323 | 1 |  | SEMA domain |  |  |
| chr1:206,338,964 | int | IVS4-27(c>t) | 191 | 1 | 0 | 0.003 | not found |  | 0.002 |  |  |  |  |  |
| chr1:206,343,231 | int | IVS5-16(g>t) | 112 | 41 | 39 | 0.310 | not found |  | 0 |  |  |  |  |  |
| chr1:206,322,500 | int | IVS9-24(a>g) | 19 | 78 | 95 | 0.698 | rs6540453 | 0.267 – 0.527 | 0.305 |  |  |  |  |  |
| chr1:206,319,400 | mis | 2414(C>G) | 182 | 10 | 0 | 0.026 | rs17011882 | 0 – 0.089 | 0.017 | 11 | A805G | Plexin-repeat | tolerated | benign |
| chr1:206,319,304 | mis | 2510(A>G) | 187 | 5 | 0 | 0.013 | rs41309627 | 0.005 – 0.008 | 0.007 | 11 | H837R | Plexin-repeat | tolerated | benign |
| chr1:206,292,321 | syn | 2967(G>A) | 191 | 1 | 0 | 0.003 | not found |  | 0 | 14 |  | IPT plexin repeat |  |  |
| chr1:206,292,297 | syn | 2991(C>T) | 173 | 19 | 0 | 0.049 | rs41283120 | 0.030 – 0.033 | 0.043 | 14 |  | IPT plexin repeat |  |  |
| chr1:206,291,238 | syn | 3147(G>A) | 191 | 1 | 0 | 0.003 | not found |  | 0.000 | 15 |  | IPT plexin repeat |  |  |
| chr1:206,278,847 | mis | 4606(G>A) | 184 | 8 | 0 | 0.021 | rs34457681 | 0.016 – 0.050 | 0.029 | 24 | V1536M | Cytoplasmic | damaging | benign |
| chr1:206,274,462 | syn | 4863(C>T) | 33 | 93 | 66 | 0.586 | rs3736963 | 0.247 – 0.668 | 0.425 | 26 |  | Cytoplasmic |  |  |
| chr1:206,273,371 | syn | 4921(C>T) | 191 | 1 | 0 | 0.003 | not found |  | 0 | 27 |  | Cytoplasmic |  |  |
| chr1:206,273,291 | mis | 5051(C>G) **^6^** | 191 | 1 | 0 | 0.003 | not found |  | 0 | 27 | T1684S | Cytoplasmic | damaging | benign |
| chr1:206,271,548 | int | IVS28+10(c>t) | 191 | 1 | 0 | 0.003 | not found |  | 0.013 |  |  |  |  |  |
| chr1:206,271,542 | int | IVS28+16(g>t) | 188 | 4 | 0 | 0.010 | rs138753762 | NA | 0.311 |  |  |  |  |  |

**^1^** Type of sequence variation: mis: missense change; syn: synonymous change and int: intronic variant. **^2^** This column indicates the nucleotide change from the reference sequence and the cDNA position of the change. Exonic and intronic changes are in upper and lower case respectively. **^3^** These columns indicate the number of TOF cases with genotypes that are homozygous for the reference allele (AA), heterozygous (AB) and homozygous for the variant minor allele (BB). **^4^** These columns indicate the minor allele frequency (MAF) in the TOF cases and in control samples from the NCBI dbSNP database build 134. For the latter, MAF range was calculated using data from studies comprising at least 10 individuals. **^5^** This column indicates the minor allele frequency (MAF) in whole exome sequence data from > 5,000 individuals from the NIH National Heart, Lung and Blood Institute (NHLBI) Exome Sequencing project (http://evs.gs.washington.edu/EVS/). **^6^** This change is in the Plexin cytoplasmic RasGAP Domain; Threonine at this position is highly conserved across vertebrates.

**Supplementary Table 7: Gene-set association results for all gene-sets tested, rare CNVs restricted to exonic losses**

Table fields: GsID: gene-set ID; GsName: gene-set name; GsSize: number of genes in the gene-set; TOF_N: number of case individuals harbouring a CNV on a gene within this gene-set; Ctrl_N: number of OPGP controls individuals harbouring a CNV on a gene within this gene-set; TOF_%: fraction of case individuals harbouring a CNV on a gene within this gene-set; Ctrl_%: fraction of OPGP controls individuals harbouring a CNV on a gene within this gene-set; FET_pv: gene-set association p-value (Fisher's Exact Test); FET_fdr: phenotype permutation-based FDR q-value (FDR: false discovery rate)

| GsID | GsName | GsSize | TOF_N | Ctrl_N | TOF_% | Ctrl_% | FET_pv | FET_fdr |
| --- | --- | --- | --- | --- | --- | --- | --- | --- |
| GO:0030334 | regulation of cell migration | 230 | 5 | 0 | 2.512563 | 0 | 0.022432 | 0.64075 |
| GO:0051270 | regulation of cellular component movement | 258 | 5 | 0 | 2.512563 | 0 | 0.022432 | 0.64075 |
| GO:0051276 | chromosome organization | 609 | 5 | 0 | 2.512563 | 0 | 0.022432 | 0.64075 |
| GO:2000145 | regulation of cell motility | 233 | 5 | 0 | 2.512563 | 0 | 0.022432 | 0.64075 |
| GO:0016477 | cell migration | 554 | 7 | 1 | 3.517588 | 0.446429 | 0.022981 | 0.443357 |
| GO:0048870 | cell motility | 590 | 7 | 1 | 3.517588 | 0.446429 | 0.022981 | 0.443357 |
| GO:0051674 | localization of cell | 590 | 7 | 1 | 3.517588 | 0.446429 | 0.022981 | 0.443357 |
| GO:0001568 | blood vessel development | 330 | 8 | 2 | 4.020101 | 0.892857 | 0.035105 | 0.383611 |
| GO:0001944 | vasculature development | 349 | 8 | 2 | 4.020101 | 0.892857 | 0.035105 | 0.383611 |
| GO:0000904 | cell morphogenesis involved in differentiation | 548 | 6 | 1 | 3.015075 | 0.446429 | 0.043967 | 0.268105 |
| GO:0006935 | chemotaxis | 520 | 6 | 1 | 3.015075 | 0.446429 | 0.043967 | 0.268105 |
| GO:0007409 | axonogenesis | 444 | 6 | 1 | 3.015075 | 0.446429 | 0.043967 | 0.268105 |
| GO:0031175 | neuron projection development | 545 | 6 | 1 | 3.015075 | 0.446429 | 0.043967 | 0.268105 |
| GO:0032990 | cell part morphogenesis | 529 | 6 | 1 | 3.015075 | 0.446429 | 0.043967 | 0.268105 |
| GO:0042330 | taxis | 520 | 6 | 1 | 3.015075 | 0.446429 | 0.043967 | 0.268105 |
| GO:0048666 | neuron development | 615 | 6 | 1 | 3.015075 | 0.446429 | 0.043967 | 0.268105 |
| GO:0048667 | cell morphogenesis involved in neuron differentiation | 478 | 6 | 1 | 3.015075 | 0.446429 | 0.043967 | 0.268105 |
| GO:0048812 | neuron projection morphogenesis | 480 | 6 | 1 | 3.015075 | 0.446429 | 0.043967 | 0.268105 |
| GO:0048858 | cell projection morphogenesis | 525 | 6 | 1 | 3.015075 | 0.446429 | 0.043967 | 0.268105 |
| GO:0006325 | chromatin organization | 464 | 4 | 0 | 2.01005 | 0 | 0.048201 | 0.290278 |
| GO:0007411 | axon guidance | 327 | 4 | 0 | 2.01005 | 0 | 0.048201 | 0.290278 |
| GO:0007417 | central nervous system development | 489 | 4 | 0 | 2.01005 | 0 | 0.048201 | 0.290278 |
| GO:0008134 | transcription factor binding | 297 | 4 | 0 | 2.01005 | 0 | 0.048201 | 0.290278 |
| GO:0008146 | sulfotransferase activity | 51 | 4 | 0 | 2.01005 | 0 | 0.048201 | 0.290278 |
| GO:0010551 | regulation of gene-specific transcription from RNA polymerase II promoter | 335 | 4 | 0 | 2.01005 | 0 | 0.048201 | 0.290278 |
| GO:0016782 | transferase activity, transferring sulfur-containing groups | 59 | 4 | 0 | 2.01005 | 0 | 0.048201 | 0.290278 |
| GO:0019220 | regulation of phosphate metabolic process | 626 | 4 | 0 | 2.01005 | 0 | 0.048201 | 0.290278 |
| GO:0030335 | positive regulation of cell migration | 136 | 4 | 0 | 2.01005 | 0 | 0.048201 | 0.290278 |
| GO:0032569 | gene-specific transcription from RNA polymerase II promoter | 337 | 4 | 0 | 2.01005 | 0 | 0.048201 | 0.290278 |
| GO:0042325 | regulation of phosphorylation | 605 | 4 | 0 | 2.01005 | 0 | 0.048201 | 0.290278 |
| GO:0051174 | regulation of phosphorus metabolic process | 626 | 4 | 0 | 2.01005 | 0 | 0.048201 | 0.290278 |
| GO:0051272 | positive regulation of cellular component movement | 146 | 4 | 0 | 2.01005 | 0 | 0.048201 | 0.290278 |
| GO:2000147 | positive regulation of cell motility | 136 | 4 | 0 | 2.01005 | 0 | 0.048201 | 0.290278 |
| KEGG:04810 | KEGG: Regulation of actin cytoskeleton | 216 | 4 | 0 | 2.01005 | 0 | 0.048201 | 0.290278 |
| KEGG:05200 | KEGG: Pathways in cancer | 328 | 4 | 0 | 2.01005 | 0 | 0.048201 | 0.290278 |
| REACT:1062 | REACT: Axon guidance | 265 | 4 | 0 | 2.01005 | 0 | 0.048201 | 0.290278 |
| GO:0006928 | cellular component movement | 685 | 7 | 2 | 3.517588 | 0.892857 | 0.06202 | 0.287974 |
| GO:0030030 | cell projection organization | 685 | 7 | 2 | 3.517588 | 0.892857 | 0.06202 | 0.287974 |
| GO:0072358 | cardiovascular system development | 516 | 8 | 3 | 4.020101 | 1.339286 | 0.076813 | 0.286088 |
| GO:0072359 | circulatory system development | 516 | 8 | 3 | 4.020101 | 1.339286 | 0.076813 | 0.286088 |
| GO:0007243 | intracellular protein kinase cascade | 640 | 5 | 1 | 2.512563 | 0.446429 | 0.082539 | 0.268694 |
| GO:0007268 | synaptic transmission | 475 | 5 | 1 | 2.512563 | 0.446429 | 0.082539 | 0.268694 |
| GO:0009719 | response to endogenous stimulus | 638 | 5 | 1 | 2.512563 | 0.446429 | 0.082539 | 0.268694 |
| GO:0009725 | response to hormone stimulus | 584 | 5 | 1 | 2.512563 | 0.446429 | 0.082539 | 0.268694 |
| GO:0023014 | signal transduction via phosphorylation event | 640 | 5 | 1 | 2.512563 | 0.446429 | 0.082539 | 0.268694 |
| GO:0032868 | response to insulin stimulus | 232 | 5 | 1 | 2.512563 | 0.446429 | 0.082539 | 0.268694 |
| GO:0032869 | cellular response to insulin stimulus | 187 | 5 | 1 | 2.512563 | 0.446429 | 0.082539 | 0.268694 |
| GO:0032870 | cellular response to hormone stimulus | 308 | 5 | 1 | 2.512563 | 0.446429 | 0.082539 | 0.268694 |
| GO:0040012 | regulation of locomotion | 261 | 5 | 1 | 2.512563 | 0.446429 | 0.082539 | 0.268694 |
| GO:0043434 | response to peptide hormone stimulus | 334 | 5 | 1 | 2.512563 | 0.446429 | 0.082539 | 0.268694 |
| GO:0043565 | sequence-specific DNA binding | 661 | 5 | 1 | 2.512563 | 0.446429 | 0.082539 | 0.268694 |
| GO:0071375 | cellular response to peptide hormone stimulus | 234 | 5 | 1 | 2.512563 | 0.446429 | 0.082539 | 0.268694 |
| GO:0071495 | cellular response to endogenous stimulus | 327 | 5 | 1 | 2.512563 | 0.446429 | 0.082539 | 0.268694 |
| KEGG:04510 | KEGG: Focal adhesion | 201 | 5 | 1 | 2.512563 | 0.446429 | 0.082539 | 0.268694 |
| GO:0000165 | MAPKKK cascade | 346 | 3 | 0 | 1.507538 | 0 | 0.103288 | 0.388686 |
| GO:0001932 | regulation of protein phosphorylation | 568 | 3 | 0 | 1.507538 | 0 | 0.103288 | 0.388686 |
| GO:0006790 | sulfur compound metabolic process | 138 | 3 | 0 | 1.507538 | 0 | 0.103288 | 0.388686 |
| GO:0006805 | xenobiotic metabolic process | 137 | 3 | 0 | 1.507538 | 0 | 0.103288 | 0.388686 |
| GO:0008286 | insulin receptor signaling pathway | 151 | 3 | 0 | 1.507538 | 0 | 0.103288 | 0.388686 |
| GO:0009410 | response to xenobiotic stimulus | 139 | 3 | 0 | 1.507538 | 0 | 0.103288 | 0.388686 |
| GO:0010594 | regulation of endothelial cell migration | 46 | 3 | 0 | 1.507538 | 0 | 0.103288 | 0.388686 |
| GO:0016568 | chromatin modification | 365 | 3 | 0 | 1.507538 | 0 | 0.103288 | 0.388686 |
| GO:0016853 | isomerase activity | 131 | 3 | 0 | 1.507538 | 0 | 0.103288 | 0.388686 |
| GO:0022603 | regulation of anatomical structure morphogenesis | 292 | 3 | 0 | 1.507538 | 0 | 0.103288 | 0.388686 |
| GO:0030029 | actin filament-based process | 329 | 3 | 0 | 1.507538 | 0 | 0.103288 | 0.388686 |
| GO:0030036 | actin cytoskeleton organization | 291 | 3 | 0 | 1.507538 | 0 | 0.103288 | 0.388686 |
| GO:0031399 | regulation of protein modification process | 716 | 3 | 0 | 1.507538 | 0 | 0.103288 | 0.388686 |
| GO:0033674 | positive regulation of kinase activity | 299 | 3 | 0 | 1.507538 | 0 | 0.103288 | 0.388686 |
| GO:0043085 | positive regulation of catalytic activity | 647 | 3 | 0 | 1.507538 | 0 | 0.103288 | 0.388686 |
| GO:0043534 | blood vessel endothelial cell migration | 35 | 3 | 0 | 1.507538 | 0 | 0.103288 | 0.388686 |
| GO:0043542 | endothelial cell migration | 68 | 3 | 0 | 1.507538 | 0 | 0.103288 | 0.388686 |
| GO:0043549 | regulation of kinase activity | 452 | 3 | 0 | 1.507538 | 0 | 0.103288 | 0.388686 |
| GO:0044057 | regulation of system process | 319 | 3 | 0 | 1.507538 | 0 | 0.103288 | 0.388686 |
| GO:0044463 | cell projection part | 401 | 3 | 0 | 1.507538 | 0 | 0.103288 | 0.388686 |
| GO:0045859 | regulation of protein kinase activity | 435 | 3 | 0 | 1.507538 | 0 | 0.103288 | 0.388686 |
| GO:0045860 | positive regulation of protein kinase activity | 289 | 3 | 0 | 1.507538 | 0 | 0.103288 | 0.388686 |
| GO:0048193 | Golgi vesicle transport | 137 | 3 | 0 | 1.507538 | 0 | 0.103288 | 0.388686 |
| GO:0051338 | regulation of transferase activity | 467 | 3 | 0 | 1.507538 | 0 | 0.103288 | 0.388686 |
| GO:0051347 | positive regulation of transferase activity | 308 | 3 | 0 | 1.507538 | 0 | 0.103288 | 0.388686 |
| GO:0071466 | cellular response to xenobiotic stimulus | 137 | 3 | 0 | 1.507538 | 0 | 0.103288 | 0.388686 |
| NCI:119 | Signaling events mediated by Hepatocyte Growth Factor Receptor (c-Met) | 76 | 3 | 0 | 1.507538 | 0 | 0.103288 | 0.388686 |
| NCI:68 | Signaling events mediated by focal adhesion kinase | 58 | 3 | 0 | 1.507538 | 0 | 0.103288 | 0.388686 |
| PF00046 | PFAM: Homeobox domain | 262 | 3 | 0 | 1.507538 | 0 | 0.103288 | 0.388686 |
| PF00622 | PFAM: SPRY domain | 106 | 3 | 0 | 1.507538 | 0 | 0.103288 | 0.388686 |
| PF00643 | PFAM: B-box zinc finger | 86 | 3 | 0 | 1.507538 | 0 | 0.103288 | 0.388686 |
| REACT:583 | REACT: Biological oxidations | 130 | 3 | 0 | 1.507538 | 0 | 0.103288 | 0.388686 |
| GO:0000902 | cell morphogenesis | 626 | 6 | 2 | 3.015075 | 0.892857 | 0.106924 | 0.385398 |
| GO:0004674 | protein serine/threonine kinase activity | 429 | 6 | 2 | 3.015075 | 0.892857 | 0.106924 | 0.385398 |
| GO:0032989 | cellular component morphogenesis | 659 | 6 | 2 | 3.015075 | 0.892857 | 0.106924 | 0.385398 |
| GO:0042060 | wound healing | 574 | 6 | 2 | 3.015075 | 0.892857 | 0.106924 | 0.385398 |
| GO:0048514 | blood vessel morphogenesis | 284 | 6 | 2 | 3.015075 | 0.892857 | 0.106924 | 0.385398 |
| GO:0050878 | regulation of body fluid levels | 554 | 6 | 2 | 3.015075 | 0.892857 | 0.106924 | 0.385398 |
| PF00069 | PFAM: Protein kinase domain | 379 | 6 | 2 | 3.015075 | 0.892857 | 0.106924 | 0.385398 |
| GO:0004672 | protein kinase activity | 595 | 7 | 3 | 3.517588 | 1.339286 | 0.124821 | 0.391537 |
| GO:0005506 | iron ion binding | 217 | 4 | 1 | 2.01005 | 0.446429 | 0.151275 | 0.445605 |
| GO:0006886 | intracellular protein transport | 456 | 4 | 1 | 2.01005 | 0.446429 | 0.151275 | 0.445605 |
| GO:0007050 | cell cycle arrest | 327 | 4 | 1 | 2.01005 | 0.446429 | 0.151275 | 0.445605 |
| GO:0017111 | nucleoside-triphosphatase activity | 744 | 4 | 1 | 2.01005 | 0.446429 | 0.151275 | 0.445605 |
| GO:0032583 | regulation of gene-specific transcription | 394 | 4 | 1 | 2.01005 | 0.446429 | 0.151275 | 0.445605 |
| GO:0032787 | monocarboxylic acid metabolic process | 347 | 4 | 1 | 2.01005 | 0.446429 | 0.151275 | 0.445605 |
| GO:0040017 | positive regulation of locomotion | 144 | 4 | 1 | 2.01005 | 0.446429 | 0.151275 | 0.445605 |
| GO:0045786 | negative regulation of cell cycle | 383 | 4 | 1 | 2.01005 | 0.446429 | 0.151275 | 0.445605 |
| GO:0045893 | positive regulation of transcription, DNA-dependent | 572 | 4 | 1 | 2.01005 | 0.446429 | 0.151275 | 0.445605 |
| GO:0051254 | positive regulation of RNA metabolic process | 580 | 4 | 1 | 2.01005 | 0.446429 | 0.151275 | 0.445605 |
| GO:0051726 | regulation of cell cycle | 580 | 4 | 1 | 2.01005 | 0.446429 | 0.151275 | 0.445605 |
| GO:0007596 | blood coagulation | 481 | 5 | 2 | 2.512563 | 0.892857 | 0.178968 | 0.455341 |
| GO:0007599 | hemostasis | 484 | 5 | 2 | 2.512563 | 0.892857 | 0.178968 | 0.455341 |
| GO:0018193 | peptidyl-amino acid modification | 428 | 5 | 2 | 2.512563 | 0.892857 | 0.178968 | 0.455341 |
| GO:0019226 | transmission of nerve impulse | 532 | 5 | 2 | 2.512563 | 0.892857 | 0.178968 | 0.455341 |
| GO:0032940 | secretion by cell | 539 | 5 | 2 | 2.512563 | 0.892857 | 0.178968 | 0.455341 |
| GO:0035637 | multicellular organismal signaling | 532 | 5 | 2 | 2.512563 | 0.892857 | 0.178968 | 0.455341 |
| GO:0044432 | endoplasmic reticulum part | 734 | 5 | 2 | 2.512563 | 0.892857 | 0.178968 | 0.455341 |
| GO:0046903 | secretion | 650 | 5 | 2 | 2.512563 | 0.892857 | 0.178968 | 0.455341 |
| GO:0048646 | anatomical structure formation involved in morphogenesis | 529 | 5 | 2 | 2.512563 | 0.892857 | 0.178968 | 0.455341 |
| GO:0050817 | coagulation | 484 | 5 | 2 | 2.512563 | 0.892857 | 0.178968 | 0.455341 |
| REACT:244 | REACT: Hemostasis | 466 | 5 | 2 | 2.512563 | 0.892857 | 0.178968 | 0.455341 |
| GO:0071310 | cellular response to organic substance | 622 | 6 | 3 | 3.015075 | 1.339286 | 0.196733 | 0.463346 |
| GO:0001775 | cell activation | 620 | 7 | 4 | 3.517588 | 1.785714 | 0.208834 | 0.458463 |
| GO:0019752 | carboxylic acid metabolic process | 746 | 7 | 4 | 3.517588 | 1.785714 | 0.208834 | 0.458463 |
| GO:0019899 | enzyme binding | 715 | 7 | 4 | 3.517588 | 1.785714 | 0.208834 | 0.458463 |
| GO:0043436 | oxoacid metabolic process | 746 | 7 | 4 | 3.517588 | 1.785714 | 0.208834 | 0.458463 |
| BIOC:178 | BIOC: PYK2 Pathway | 30 | 2 | 0 | 1.005025 | 0 | 0.220732 | 0.548053 |
| BIOC:31 | BIOC: INTEGRIN Pathway | 34 | 2 | 0 | 1.005025 | 0 | 0.220732 | 0.548053 |
| BIOC:76 | BIOC: MET Pathway | 37 | 2 | 0 | 1.005025 | 0 | 0.220732 | 0.548053 |
| GO:0000118 | histone deacetylase complex | 42 | 2 | 0 | 1.005025 | 0 | 0.220732 | 0.548053 |
| GO:0000187 | activation of MAPK activity | 106 | 2 | 0 | 1.005025 | 0 | 0.220732 | 0.548053 |
| GO:0000228 | nuclear chromosome | 210 | 2 | 0 | 1.005025 | 0 | 0.220732 | 0.548053 |
| GO:0000271 | polysaccharide biosynthetic process | 67 | 2 | 0 | 1.005025 | 0 | 0.220732 | 0.548053 |
| GO:0001570 | vasculogenesis | 44 | 2 | 0 | 1.005025 | 0 | 0.220732 | 0.548053 |
| GO:0003002 | regionalization | 239 | 2 | 0 | 1.005025 | 0 | 0.220732 | 0.548053 |
| GO:0003714 | transcription corepressor activity | 149 | 2 | 0 | 1.005025 | 0 | 0.220732 | 0.548053 |
| GO:0004497 | monooxygenase activity | 91 | 2 | 0 | 1.005025 | 0 | 0.220732 | 0.548053 |
| GO:0005539 | glycosaminoglycan binding | 152 | 2 | 0 | 1.005025 | 0 | 0.220732 | 0.548053 |
| GO:0005769 | early endosome | 146 | 2 | 0 | 1.005025 | 0 | 0.220732 | 0.548053 |
| GO:0005788 | endoplasmic reticulum lumen | 102 | 2 | 0 | 1.005025 | 0 | 0.220732 | 0.548053 |
| GO:0005912 | adherens junction | 154 | 2 | 0 | 1.005025 | 0 | 0.220732 | 0.548053 |
| GO:0005924 | cell-substrate adherens junction | 107 | 2 | 0 | 1.005025 | 0 | 0.220732 | 0.548053 |
| GO:0005925 | focal adhesion | 102 | 2 | 0 | 1.005025 | 0 | 0.220732 | 0.548053 |
| GO:0006023 | aminoglycan biosynthetic process | 26 | 2 | 0 | 1.005025 | 0 | 0.220732 | 0.548053 |
| GO:0006029 | proteoglycan metabolic process | 46 | 2 | 0 | 1.005025 | 0 | 0.220732 | 0.548053 |
| GO:0006397 | mRNA processing | 376 | 2 | 0 | 1.005025 | 0 | 0.220732 | 0.548053 |
| GO:0006476 | protein deacetylation | 31 | 2 | 0 | 1.005025 | 0 | 0.220732 | 0.548053 |
| GO:0006631 | fatty acid metabolic process | 257 | 2 | 0 | 1.005025 | 0 | 0.220732 | 0.548053 |
| GO:0006633 | fatty acid biosynthetic process | 116 | 2 | 0 | 1.005025 | 0 | 0.220732 | 0.548053 |
| GO:0006869 | lipid transport | 177 | 2 | 0 | 1.005025 | 0 | 0.220732 | 0.548053 |
| GO:0006913 | nucleocytoplasmic transport | 260 | 2 | 0 | 1.005025 | 0 | 0.220732 | 0.548053 |
| GO:0007059 | chromosome segregation | 114 | 2 | 0 | 1.005025 | 0 | 0.220732 | 0.548053 |
| GO:0007219 | Notch signaling pathway | 69 | 2 | 0 | 1.005025 | 0 | 0.220732 | 0.548053 |
| GO:0007229 | integrin-mediated signaling pathway | 57 | 2 | 0 | 1.005025 | 0 | 0.220732 | 0.548053 |
| GO:0007420 | brain development | 349 | 2 | 0 | 1.005025 | 0 | 0.220732 | 0.548053 |
| GO:0008201 | heparin binding | 116 | 2 | 0 | 1.005025 | 0 | 0.220732 | 0.548053 |
| GO:0008285 | negative regulation of cell proliferation | 395 | 2 | 0 | 1.005025 | 0 | 0.220732 | 0.548053 |
| GO:0010553 | negative regulation of gene-specific transcription from RNA polymerase II promoter | 149 | 2 | 0 | 1.005025 | 0 | 0.220732 | 0.548053 |
| GO:0010595 | positive regulation of endothelial cell migration | 28 | 2 | 0 | 1.005025 | 0 | 0.220732 | 0.548053 |
| GO:0010876 | lipid localization | 198 | 2 | 0 | 1.005025 | 0 | 0.220732 | 0.548053 |
| GO:0016053 | organic acid biosynthetic process | 236 | 2 | 0 | 1.005025 | 0 | 0.220732 | 0.548053 |
| GO:0016071 | mRNA metabolic process | 506 | 2 | 0 | 1.005025 | 0 | 0.220732 | 0.548053 |
| GO:0016202 | regulation of striated muscle tissue development | 53 | 2 | 0 | 1.005025 | 0 | 0.220732 | 0.548053 |
| GO:0016566 | specific transcriptional repressor activity | 95 | 2 | 0 | 1.005025 | 0 | 0.220732 | 0.548053 |
| GO:0016569 | covalent chromatin modification | 200 | 2 | 0 | 1.005025 | 0 | 0.220732 | 0.548053 |
| GO:0016570 | histone modification | 197 | 2 | 0 | 1.005025 | 0 | 0.220732 | 0.548053 |
| GO:0016585 | chromatin remodeling complex | 94 | 2 | 0 | 1.005025 | 0 | 0.220732 | 0.548053 |
| GO:0016860 | intramolecular oxidoreductase activity | 39 | 2 | 0 | 1.005025 | 0 | 0.220732 | 0.548053 |
| GO:0016887 | ATPase activity | 350 | 2 | 0 | 1.005025 | 0 | 0.220732 | 0.548053 |
| GO:0018205 | peptidyl-lysine modification | 96 | 2 | 0 | 1.005025 | 0 | 0.220732 | 0.548053 |
| GO:0020037 | heme binding | 130 | 2 | 0 | 1.005025 | 0 | 0.220732 | 0.548053 |
| GO:0030055 | cell-substrate junction | 111 | 2 | 0 | 1.005025 | 0 | 0.220732 | 0.548053 |
| GO:0030111 | regulation of Wnt receptor signaling pathway | 127 | 2 | 0 | 1.005025 | 0 | 0.220732 | 0.548053 |
| GO:0030166 | proteoglycan biosynthetic process | 30 | 2 | 0 | 1.005025 | 0 | 0.220732 | 0.548053 |
| GO:0030183 | B cell differentiation | 54 | 2 | 0 | 1.005025 | 0 | 0.220732 | 0.548053 |
| GO:0030198 | extracellular matrix organization | 98 | 2 | 0 | 1.005025 | 0 | 0.220732 | 0.548053 |
| GO:0030203 | glycosaminoglycan metabolic process | 57 | 2 | 0 | 1.005025 | 0 | 0.220732 | 0.548053 |
| GO:0030425 | dendrite | 197 | 2 | 0 | 1.005025 | 0 | 0.220732 | 0.548053 |
| GO:0031644 | regulation of neurological system process | 142 | 2 | 0 | 1.005025 | 0 | 0.220732 | 0.548053 |
| GO:0032386 | regulation of intracellular transport | 115 | 2 | 0 | 1.005025 | 0 | 0.220732 | 0.548053 |
| GO:0032387 | negative regulation of intracellular transport | 36 | 2 | 0 | 1.005025 | 0 | 0.220732 | 0.548053 |
| GO:0033157 | regulation of intracellular protein transport | 96 | 2 | 0 | 1.005025 | 0 | 0.220732 | 0.548053 |
| GO:0034708 | methyltransferase complex | 58 | 2 | 0 | 1.005025 | 0 | 0.220732 | 0.548053 |
| GO:0035097 | histone methyltransferase complex | 58 | 2 | 0 | 1.005025 | 0 | 0.220732 | 0.548053 |
| GO:0035107 | appendage morphogenesis | 104 | 2 | 0 | 1.005025 | 0 | 0.220732 | 0.548053 |
| GO:0035108 | limb morphogenesis | 104 | 2 | 0 | 1.005025 | 0 | 0.220732 | 0.548053 |
| GO:0035601 | protein deacylation | 32 | 2 | 0 | 1.005025 | 0 | 0.220732 | 0.548053 |
| GO:0042826 | histone deacetylase binding | 45 | 2 | 0 | 1.005025 | 0 | 0.220732 | 0.548053 |
| GO:0043405 | regulation of MAP kinase activity | 189 | 2 | 0 | 1.005025 | 0 | 0.220732 | 0.548053 |
| GO:0043406 | positive regulation of MAP kinase activity | 136 | 2 | 0 | 1.005025 | 0 | 0.220732 | 0.548053 |
| GO:0043408 | regulation of MAPKKK cascade | 183 | 2 | 0 | 1.005025 | 0 | 0.220732 | 0.548053 |
| GO:0044272 | sulfur compound biosynthetic process | 56 | 2 | 0 | 1.005025 | 0 | 0.220732 | 0.548053 |
| GO:0044454 | nuclear chromosome part | 169 | 2 | 0 | 1.005025 | 0 | 0.220732 | 0.548053 |
| GO:0045765 | regulation of angiogenesis | 94 | 2 | 0 | 1.005025 | 0 | 0.220732 | 0.548053 |
| GO:0045766 | positive regulation of angiogenesis | 48 | 2 | 0 | 1.005025 | 0 | 0.220732 | 0.548053 |
| GO:0045944 | positive regulation of transcription from RNA polymerase II promoter | 439 | 2 | 0 | 1.005025 | 0 | 0.220732 | 0.548053 |
| GO:0046394 | carboxylic acid biosynthetic process | 236 | 2 | 0 | 1.005025 | 0 | 0.220732 | 0.548053 |
| GO:0046546 | development of primary male sexual characteristics | 84 | 2 | 0 | 1.005025 | 0 | 0.220732 | 0.548053 |
| GO:0046661 | male sex differentiation | 90 | 2 | 0 | 1.005025 | 0 | 0.220732 | 0.548053 |
| GO:0046822 | regulation of nucleocytoplasmic transport | 99 | 2 | 0 | 1.005025 | 0 | 0.220732 | 0.548053 |
| GO:0046823 | negative regulation of nucleocytoplasmic transport | 32 | 2 | 0 | 1.005025 | 0 | 0.220732 | 0.548053 |
| GO:0046906 | tetrapyrrole binding | 138 | 2 | 0 | 1.005025 | 0 | 0.220732 | 0.548053 |
| GO:0048610 | cellular process involved in reproduction | 353 | 2 | 0 | 1.005025 | 0 | 0.220732 | 0.548053 |
| GO:0048634 | regulation of muscle organ development | 55 | 2 | 0 | 1.005025 | 0 | 0.220732 | 0.548053 |
| GO:0048641 | regulation of skeletal muscle tissue development | 30 | 2 | 0 | 1.005025 | 0 | 0.220732 | 0.548053 |
| GO:0048736 | appendage development | 109 | 2 | 0 | 1.005025 | 0 | 0.220732 | 0.548053 |
| GO:0050804 | regulation of synaptic transmission | 117 | 2 | 0 | 1.005025 | 0 | 0.220732 | 0.548053 |
| GO:0051147 | regulation of muscle cell differentiation | 64 | 2 | 0 | 1.005025 | 0 | 0.220732 | 0.548053 |
| GO:0051153 | regulation of striated muscle cell differentiation | 31 | 2 | 0 | 1.005025 | 0 | 0.220732 | 0.548053 |
| GO:0051169 | nuclear transport | 262 | 2 | 0 | 1.005025 | 0 | 0.220732 | 0.548053 |
| GO:0051224 | negative regulation of protein transport | 57 | 2 | 0 | 1.005025 | 0 | 0.220732 | 0.548053 |
| GO:0051247 | positive regulation of protein metabolic process | 365 | 2 | 0 | 1.005025 | 0 | 0.220732 | 0.548053 |
| GO:0051969 | regulation of transmission of nerve impulse | 129 | 2 | 0 | 1.005025 | 0 | 0.220732 | 0.548053 |
| GO:0060173 | limb development | 109 | 2 | 0 | 1.005025 | 0 | 0.220732 | 0.548053 |
| GO:0060284 | regulation of cell development | 296 | 2 | 0 | 1.005025 | 0 | 0.220732 | 0.548053 |
| GO:0070161 | anchoring junction | 172 | 2 | 0 | 1.005025 | 0 | 0.220732 | 0.548053 |
| GO:0071702 | organic substance transport | 488 | 2 | 0 | 1.005025 | 0 | 0.220732 | 0.548053 |
| GO:0071900 | regulation of protein serine/threonine kinase activity | 264 | 2 | 0 | 1.005025 | 0 | 0.220732 | 0.548053 |
| GO:0071902 | positive regulation of protein serine/threonine kinase activity | 161 | 2 | 0 | 1.005025 | 0 | 0.220732 | 0.548053 |
| GO:0080135 | regulation of cellular response to stress | 154 | 2 | 0 | 1.005025 | 0 | 0.220732 | 0.548053 |
| GO:0090317 | negative regulation of intracellular protein transport | 34 | 2 | 0 | 1.005025 | 0 | 0.220732 | 0.548053 |
| GO:2000241 | regulation of reproductive process | 107 | 2 | 0 | 1.005025 | 0 | 0.220732 | 0.548053 |
| KEGG:00380 | KEGG: Tryptophan metabolism | 40 | 2 | 0 | 1.005025 | 0 | 0.220732 | 0.548053 |
| KEGG:00590 | KEGG: Arachidonic acid metabolism | 58 | 2 | 0 | 1.005025 | 0 | 0.220732 | 0.548053 |
| KEGG:04010 | KEGG: MAPK signaling pathway | 267 | 2 | 0 | 1.005025 | 0 | 0.220732 | 0.548053 |
| KEGG:04062 | KEGG: Chemokine signaling pathway | 189 | 2 | 0 | 1.005025 | 0 | 0.220732 | 0.548053 |
| KEGG:04360 | KEGG: Axon guidance | 129 | 2 | 0 | 1.005025 | 0 | 0.220732 | 0.548053 |
| KEGG:05146 | KEGG: Amoebiasis | 105 | 2 | 0 | 1.005025 | 0 | 0.220732 | 0.548053 |
| KEGG:05218 | KEGG: Melanoma | 71 | 2 | 0 | 1.005025 | 0 | 0.220732 | 0.548053 |
| NCI:127 | Netrin-mediated signaling events | 30 | 2 | 0 | 1.005025 | 0 | 0.220732 | 0.548053 |
| NCI:143 | PDGFR-beta signaling pathway | 126 | 2 | 0 | 1.005025 | 0 | 0.220732 | 0.548053 |
| NCI:196 | Urokinase-type plasminogen activator (uPA) and uPAR-mediated signaling | 45 | 2 | 0 | 1.005025 | 0 | 0.220732 | 0.548053 |
| NCI:38 | CDC42 signaling events | 70 | 2 | 0 | 1.005025 | 0 | 0.220732 | 0.548053 |
| NCI:50 | Stabilization and expansion of the E-cadherin adherens junction | 40 | 2 | 0 | 1.005025 | 0 | 0.220732 | 0.548053 |
| NCI:51 | Endothelins | 63 | 2 | 0 | 1.005025 | 0 | 0.220732 | 0.548053 |
| NCI:80 | Signaling events mediated by HDAC Class I | 66 | 2 | 0 | 1.005025 | 0 | 0.220732 | 0.548053 |
| NCI:81 | Signaling events mediated by HDAC Class II | 34 | 2 | 0 | 1.005025 | 0 | 0.220732 | 0.548053 |
| NCI:94 | IGF1 pathway | 28 | 2 | 0 | 1.005025 | 0 | 0.220732 | 0.548053 |
| PF00067 | PFAM: Cytochrome P450 | 64 | 2 | 0 | 1.005025 | 0 | 0.220732 | 0.548053 |
| PF00685 | PFAM: Sulfotransferase domain | 38 | 2 | 0 | 1.005025 | 0 | 0.220732 | 0.548053 |
| PF00787 | PFAM: PX domain | 51 | 2 | 0 | 1.005025 | 0 | 0.220732 | 0.548053 |
| PF07648 | PFAM: Kazal-type serine protease inhibitor domain | 35 | 2 | 0 | 1.005025 | 0 | 0.220732 | 0.548053 |
| REACT:146 | REACT: Cytochrome P450 - arranged by substrate type | 49 | 2 | 0 | 1.005025 | 0 | 0.220732 | 0.548053 |
| REACT:200 | REACT: IRS-mediated signalling | 80 | 2 | 0 | 1.005025 | 0 | 0.220732 | 0.548053 |
| REACT:28 | REACT: Signaling by Insulin receptor | 108 | 2 | 0 | 1.005025 | 0 | 0.220732 | 0.548053 |
| REACT:304 | REACT: Insulin receptor signalling cascade | 85 | 2 | 0 | 1.005025 | 0 | 0.220732 | 0.548053 |
| REACT:592 | REACT: Phase 1 - Functionalization of compounds | 68 | 2 | 0 | 1.005025 | 0 | 0.220732 | 0.548053 |
| REACT:667 | REACT: Signaling by PDGF | 64 | 2 | 0 | 1.005025 | 0 | 0.220732 | 0.548053 |
| REACT:797 | REACT: IRS-related events | 80 | 2 | 0 | 1.005025 | 0 | 0.220732 | 0.548053 |
| REACT:920 | REACT: Down-stream signal transduction | 36 | 2 | 0 | 1.005025 | 0 | 0.220732 | 0.548053 |
| GO:0000279 | M phase | 458 | 3 | 1 | 1.507538 | 0.446429 | 0.268549 | 0.583745 |
| GO:0005088 | Ras guanyl-nucleotide exchange factor activity | 91 | 3 | 1 | 1.507538 | 0.446429 | 0.268549 | 0.583745 |
| GO:0005667 | transcription factor complex | 248 | 3 | 1 | 1.507538 | 0.446429 | 0.268549 | 0.583745 |
| GO:0005694 | chromosome | 534 | 3 | 1 | 1.507538 | 0.446429 | 0.268549 | 0.583745 |
| GO:0006281 | DNA repair | 339 | 3 | 1 | 1.507538 | 0.446429 | 0.268549 | 0.583745 |
| GO:0006605 | protein targeting | 284 | 3 | 1 | 1.507538 | 0.446429 | 0.268549 | 0.583745 |
| GO:0009055 | electron carrier activity | 177 | 3 | 1 | 1.507538 | 0.446429 | 0.268549 | 0.583745 |
| GO:0009790 | embryo development | 656 | 3 | 1 | 1.507538 | 0.446429 | 0.268549 | 0.583745 |
| GO:0010564 | regulation of cell cycle process | 329 | 3 | 1 | 1.507538 | 0.446429 | 0.268549 | 0.583745 |
| GO:0010817 | regulation of hormone levels | 332 | 3 | 1 | 1.507538 | 0.446429 | 0.268549 | 0.583745 |
| GO:0016323 | basolateral plasma membrane | 237 | 3 | 1 | 1.507538 | 0.446429 | 0.268549 | 0.583745 |
| GO:0016564 | transcription repressor activity | 303 | 3 | 1 | 1.507538 | 0.446429 | 0.268549 | 0.583745 |
| GO:0019901 | protein kinase binding | 202 | 3 | 1 | 1.507538 | 0.446429 | 0.268549 | 0.583745 |
| GO:0032880 | regulation of protein localization | 203 | 3 | 1 | 1.507538 | 0.446429 | 0.268549 | 0.583745 |
| GO:0044283 | small molecule biosynthetic process | 632 | 3 | 1 | 1.507538 | 0.446429 | 0.268549 | 0.583745 |
| GO:0044427 | chromosomal part | 448 | 3 | 1 | 1.507538 | 0.446429 | 0.268549 | 0.583745 |
| GO:0051051 | negative regulation of transport | 182 | 3 | 1 | 1.507538 | 0.446429 | 0.268549 | 0.583745 |
| GO:0051223 | regulation of protein transport | 165 | 3 | 1 | 1.507538 | 0.446429 | 0.268549 | 0.583745 |
| GO:0060341 | regulation of cellular localization | 398 | 3 | 1 | 1.507538 | 0.446429 | 0.268549 | 0.583745 |
| GO:0070201 | regulation of establishment of protein localization | 179 | 3 | 1 | 1.507538 | 0.446429 | 0.268549 | 0.583745 |
| GO:0071156 | regulation of cell cycle arrest | 222 | 3 | 1 | 1.507538 | 0.446429 | 0.268549 | 0.583745 |
| PF00096 | PFAM: Zinc finger, C2H2 type | 700 | 3 | 1 | 1.507538 | 0.446429 | 0.268549 | 0.583745 |
| PF00097 | PFAM: Zinc finger, C3HC4 type (RING finger) | 199 | 3 | 1 | 1.507538 | 0.446429 | 0.268549 | 0.583745 |
| PF01352 | PFAM: KRAB box | 415 | 3 | 1 | 1.507538 | 0.446429 | 0.268549 | 0.583745 |
| REACT:432 | REACT: Synaptic Transmission | 197 | 3 | 1 | 1.507538 | 0.446429 | 0.268549 | 0.583745 |
| REACT:506 | REACT: Transmission across Chemical Synapses | 192 | 3 | 1 | 1.507538 | 0.446429 | 0.268549 | 0.583745 |
| REACT:684 | REACT: Neuroransmitter Receptor Binding And Downstream Transmission In The Postsynaptic Cell | 136 | 3 | 1 | 1.507538 | 0.446429 | 0.268549 | 0.583745 |
| REACT:894 | REACT: Formation of Platelet plug | 262 | 3 | 1 | 1.507538 | 0.446429 | 0.268549 | 0.583745 |
| GO:0001525 | angiogenesis | 240 | 4 | 2 | 2.01005 | 0.892857 | 0.288749 | 0.595046 |
| GO:0005789 | endoplasmic reticulum membrane | 650 | 4 | 2 | 2.01005 | 0.892857 | 0.288749 | 0.595046 |
| GO:0006974 | response to DNA damage stimulus | 497 | 4 | 2 | 2.01005 | 0.892857 | 0.288749 | 0.595046 |
| GO:0019900 | kinase binding | 238 | 4 | 2 | 2.01005 | 0.892857 | 0.288749 | 0.595046 |
| GO:0022403 | cell cycle phase | 728 | 4 | 2 | 2.01005 | 0.892857 | 0.288749 | 0.595046 |
| GO:0044451 | nucleoplasm part | 701 | 4 | 2 | 2.01005 | 0.892857 | 0.288749 | 0.595046 |
| GO:0045934 | negative regulation of nucleobase, nucleoside, nucleotide and nucleic acid metabolic process | 733 | 4 | 2 | 2.01005 | 0.892857 | 0.288749 | 0.595046 |
| GO:0046983 | protein dimerization activity | 625 | 4 | 2 | 2.01005 | 0.892857 | 0.288749 | 0.595046 |
| GO:0051172 | negative regulation of nitrogen compound metabolic process | 740 | 4 | 2 | 2.01005 | 0.892857 | 0.288749 | 0.595046 |
| REACT:666 | REACT: Gene Expression | 429 | 4 | 2 | 2.01005 | 0.892857 | 0.288749 | 0.595046 |
| GO:0030054 | cell junction | 590 | 5 | 3 | 2.512563 | 1.339286 | 0.299041 | 0.601798 |
| GO:0034613 | cellular protein localization | 567 | 5 | 3 | 2.512563 | 1.339286 | 0.299041 | 0.601798 |
| GO:0045321 | leukocyte activation | 399 | 5 | 3 | 2.512563 | 1.339286 | 0.299041 | 0.601798 |
| GO:0046649 | lymphocyte activation | 343 | 5 | 3 | 2.512563 | 1.339286 | 0.299041 | 0.601798 |
| GO:0051049 | regulation of transport | 663 | 5 | 3 | 2.512563 | 1.339286 | 0.299041 | 0.601798 |
| GO:0070727 | cellular macromolecule localization | 569 | 5 | 3 | 2.512563 | 1.339286 | 0.299041 | 0.601798 |
| GO:0016773 | phosphotransferase activity, alcohol group as acceptor | 710 | 7 | 5 | 3.517588 | 2.232143 | 0.30753 | 0.608412 |
| GO:0006163 | purine nucleotide metabolic process | 644 | 6 | 5 | 3.015075 | 2.232143 | 0.419521 | 0.60767 |
| GO:0016491 | oxidoreductase activity | 705 | 6 | 5 | 3.015075 | 2.232143 | 0.419521 | 0.60767 |
| GO:0044282 | small molecule catabolic process | 688 | 6 | 5 | 3.015075 | 2.232143 | 0.419521 | 0.60767 |
| GO:0046700 | heterocycle catabolic process | 457 | 6 | 5 | 3.015075 | 2.232143 | 0.419521 | 0.60767 |
| GO:0005768 | endosome | 440 | 5 | 4 | 2.512563 | 1.785714 | 0.426926 | 0.613942 |
| GO:0000139 | Golgi membrane | 478 | 4 | 3 | 2.01005 | 1.339286 | 0.435123 | 0.631198 |
| GO:0002520 | immune system development | 377 | 4 | 3 | 2.01005 | 1.339286 | 0.435123 | 0.631198 |
| GO:0007167 | enzyme linked receptor protein signaling pathway | 737 | 4 | 3 | 2.01005 | 1.339286 | 0.435123 | 0.631198 |
| GO:0007169 | transmembrane receptor protein tyrosine kinase signaling pathway | 551 | 4 | 3 | 2.01005 | 1.339286 | 0.435123 | 0.631198 |
| GO:0042175 | nuclear membrane-endoplasmic reticulum network | 665 | 4 | 3 | 2.01005 | 1.339286 | 0.435123 | 0.631198 |
| GO:0044255 | cellular lipid metabolic process | 664 | 4 | 3 | 2.01005 | 1.339286 | 0.435123 | 0.631198 |
| GO:0044431 | Golgi apparatus part | 578 | 4 | 3 | 2.01005 | 1.339286 | 0.435123 | 0.631198 |
| GO:0044456 | synapse part | 297 | 4 | 3 | 2.01005 | 1.339286 | 0.435123 | 0.631198 |
| GO:0045202 | synapse | 398 | 4 | 3 | 2.01005 | 1.339286 | 0.435123 | 0.631198 |
| GO:0048534 | hemopoietic or lymphoid organ development | 350 | 4 | 3 | 2.01005 | 1.339286 | 0.435123 | 0.631198 |
| GO:0000278 | mitotic cell cycle | 659 | 3 | 2 | 1.507538 | 0.892857 | 0.444459 | 0.644416 |
| GO:0002253 | activation of immune response | 250 | 3 | 2 | 1.507538 | 0.892857 | 0.444459 | 0.644416 |
| GO:0002521 | leukocyte differentiation | 208 | 3 | 2 | 1.507538 | 0.892857 | 0.444459 | 0.644416 |
| GO:0003006 | developmental process involved in reproduction | 329 | 3 | 2 | 1.507538 | 0.892857 | 0.444459 | 0.644416 |
| GO:0004984 | olfactory receptor activity | 419 | 3 | 2 | 1.507538 | 0.892857 | 0.444459 | 0.644416 |
| GO:0005759 | mitochondrial matrix | 263 | 3 | 2 | 1.507538 | 0.892857 | 0.444459 | 0.644416 |
| GO:0005975 | carbohydrate metabolic process | 630 | 3 | 2 | 1.507538 | 0.892857 | 0.444459 | 0.644416 |
| GO:0006897 | endocytosis | 281 | 3 | 2 | 1.507538 | 0.892857 | 0.444459 | 0.644416 |
| GO:0007606 | sensory perception of chemical stimulus | 481 | 3 | 2 | 1.507538 | 0.892857 | 0.444459 | 0.644416 |
| GO:0007608 | sensory perception of smell | 434 | 3 | 2 | 1.507538 | 0.892857 | 0.444459 | 0.644416 |
| GO:0010324 | membrane invagination | 281 | 3 | 2 | 1.507538 | 0.892857 | 0.444459 | 0.644416 |
| GO:0010629 | negative regulation of gene expression | 728 | 3 | 2 | 1.507538 | 0.892857 | 0.444459 | 0.644416 |
| GO:0016481 | negative regulation of transcription | 668 | 3 | 2 | 1.507538 | 0.892857 | 0.444459 | 0.644416 |
| GO:0016788 | hydrolase activity, acting on ester bonds | 710 | 3 | 2 | 1.507538 | 0.892857 | 0.444459 | 0.644416 |
| GO:0016810 | hydrolase activity, acting on carbon-nitrogen (but not peptide) bonds | 117 | 3 | 2 | 1.507538 | 0.892857 | 0.444459 | 0.644416 |
| GO:0019904 | protein domain specific binding | 433 | 3 | 2 | 1.507538 | 0.892857 | 0.444459 | 0.644416 |
| GO:0030097 | hemopoiesis | 326 | 3 | 2 | 1.507538 | 0.892857 | 0.444459 | 0.644416 |
| GO:0030098 | lymphocyte differentiation | 144 | 3 | 2 | 1.507538 | 0.892857 | 0.444459 | 0.644416 |
| GO:0031225 | anchored to membrane | 133 | 3 | 2 | 1.507538 | 0.892857 | 0.444459 | 0.644416 |
| GO:0042692 | muscle cell differentiation | 160 | 3 | 2 | 1.507538 | 0.892857 | 0.444459 | 0.644416 |
| GO:0042803 | protein homodimerization activity | 422 | 3 | 2 | 1.507538 | 0.892857 | 0.444459 | 0.644416 |
| GO:0043005 | neuron projection | 399 | 3 | 2 | 1.507538 | 0.892857 | 0.444459 | 0.644416 |
| GO:0050778 | positive regulation of immune response | 304 | 3 | 2 | 1.507538 | 0.892857 | 0.444459 | 0.644416 |
| GO:0051094 | positive regulation of developmental process | 415 | 3 | 2 | 1.507538 | 0.892857 | 0.444459 | 0.644416 |
| GO:0051253 | negative regulation of RNA metabolic process | 500 | 3 | 2 | 1.507538 | 0.892857 | 0.444459 | 0.644416 |
| GO:0061061 | muscle structure development | 308 | 3 | 2 | 1.507538 | 0.892857 | 0.444459 | 0.644416 |
| GO:2000113 | negative regulation of cellular macromolecule biosynthetic process | 747 | 3 | 2 | 1.507538 | 0.892857 | 0.444459 | 0.644416 |
| REACT:1035 | REACT: Olfactory Signaling Pathway | 378 | 3 | 2 | 1.507538 | 0.892857 | 0.444459 | 0.644416 |
| REACT:352 | REACT: Transmembrane transport of small molecules | 401 | 3 | 2 | 1.507538 | 0.892857 | 0.444459 | 0.644416 |
| GO:0000075 | cell cycle checkpoint | 213 | 2 | 1 | 1.005025 | 0.446429 | 0.45562 | 0.646366 |
| GO:0000087 | M phase of mitotic cell cycle | 332 | 2 | 1 | 1.005025 | 0.446429 | 0.45562 | 0.646366 |
| GO:0000122 | negative regulation of transcription from RNA polymerase II promoter | 368 | 2 | 1 | 1.005025 | 0.446429 | 0.45562 | 0.646366 |
| GO:0000236 | mitotic prometaphase | 86 | 2 | 1 | 1.005025 | 0.446429 | 0.45562 | 0.646366 |
| GO:0000280 | nuclear division | 321 | 2 | 1 | 1.005025 | 0.446429 | 0.45562 | 0.646366 |
| GO:0000775 | chromosome, centromeric region | 145 | 2 | 1 | 1.005025 | 0.446429 | 0.45562 | 0.646366 |
| GO:0000776 | kinetochore | 90 | 2 | 1 | 1.005025 | 0.446429 | 0.45562 | 0.646366 |
| GO:0000777 | condensed chromosome kinetochore | 74 | 2 | 1 | 1.005025 | 0.446429 | 0.45562 | 0.646366 |
| GO:0000779 | condensed chromosome, centromeric region | 79 | 2 | 1 | 1.005025 | 0.446429 | 0.45562 | 0.646366 |
| GO:0000793 | condensed chromosome | 140 | 2 | 1 | 1.005025 | 0.446429 | 0.45562 | 0.646366 |
| GO:0000988 | protein binding transcription factor activity | 376 | 2 | 1 | 1.005025 | 0.446429 | 0.45562 | 0.646366 |
| GO:0000989 | transcription factor binding transcription factor activity | 376 | 2 | 1 | 1.005025 | 0.446429 | 0.45562 | 0.646366 |
| GO:0001871 | pattern binding | 166 | 2 | 1 | 1.005025 | 0.446429 | 0.45562 | 0.646366 |
| GO:0002429 | immune response-activating cell surface receptor signaling pathway | 118 | 2 | 1 | 1.005025 | 0.446429 | 0.45562 | 0.646366 |
| GO:0002768 | immune response-regulating cell surface receptor signaling pathway | 122 | 2 | 1 | 1.005025 | 0.446429 | 0.45562 | 0.646366 |
| GO:0003712 | transcription cofactor activity | 374 | 2 | 1 | 1.005025 | 0.446429 | 0.45562 | 0.646366 |
| GO:0003924 | GTPase activity | 217 | 2 | 1 | 1.005025 | 0.446429 | 0.45562 | 0.646366 |
| GO:0005080 | protein kinase C binding | 34 | 2 | 1 | 1.005025 | 0.446429 | 0.45562 | 0.646366 |
| GO:0005089 | Rho guanyl-nucleotide exchange factor activity | 73 | 2 | 1 | 1.005025 | 0.446429 | 0.45562 | 0.646366 |
| GO:0005230 | extracellular ligand-gated ion channel activity | 73 | 2 | 1 | 1.005025 | 0.446429 | 0.45562 | 0.646366 |
| GO:0005604 | basement membrane | 72 | 2 | 1 | 1.005025 | 0.446429 | 0.45562 | 0.646366 |
| GO:0005792 | microsome | 243 | 2 | 1 | 1.005025 | 0.446429 | 0.45562 | 0.646366 |
| GO:0005976 | polysaccharide metabolic process | 134 | 2 | 1 | 1.005025 | 0.446429 | 0.45562 | 0.646366 |
| GO:0006022 | aminoglycan metabolic process | 67 | 2 | 1 | 1.005025 | 0.446429 | 0.45562 | 0.646366 |
| GO:0006720 | isoprenoid metabolic process | 64 | 2 | 1 | 1.005025 | 0.446429 | 0.45562 | 0.646366 |
| GO:0006721 | terpenoid metabolic process | 43 | 2 | 1 | 1.005025 | 0.446429 | 0.45562 | 0.646366 |
| GO:0006887 | exocytosis | 224 | 2 | 1 | 1.005025 | 0.446429 | 0.45562 | 0.646366 |
| GO:0006909 | phagocytosis | 67 | 2 | 1 | 1.005025 | 0.446429 | 0.45562 | 0.646366 |
| GO:0007067 | mitosis | 321 | 2 | 1 | 1.005025 | 0.446429 | 0.45562 | 0.646366 |
| GO:0007156 | homophilic cell adhesion | 139 | 2 | 1 | 1.005025 | 0.446429 | 0.45562 | 0.646366 |
| GO:0007173 | epidermal growth factor receptor signaling pathway | 96 | 2 | 1 | 1.005025 | 0.446429 | 0.45562 | 0.646366 |
| GO:0007266 | Rho protein signal transduction | 147 | 2 | 1 | 1.005025 | 0.446429 | 0.45562 | 0.646366 |
| GO:0007346 | regulation of mitotic cell cycle | 254 | 2 | 1 | 1.005025 | 0.446429 | 0.45562 | 0.646366 |
| GO:0007389 | pattern specification process | 336 | 2 | 1 | 1.005025 | 0.446429 | 0.45562 | 0.646366 |
| GO:0007507 | heart development | 240 | 2 | 1 | 1.005025 | 0.446429 | 0.45562 | 0.646366 |
| GO:0007517 | muscle organ development | 229 | 2 | 1 | 1.005025 | 0.446429 | 0.45562 | 0.646366 |
| GO:0007519 | skeletal muscle tissue development | 91 | 2 | 1 | 1.005025 | 0.446429 | 0.45562 | 0.646366 |
| GO:0007586 | digestion | 106 | 2 | 1 | 1.005025 | 0.446429 | 0.45562 | 0.646366 |
| GO:0009100 | glycoprotein metabolic process | 255 | 2 | 1 | 1.005025 | 0.446429 | 0.45562 | 0.646366 |
| GO:0009101 | glycoprotein biosynthetic process | 210 | 2 | 1 | 1.005025 | 0.446429 | 0.45562 | 0.646366 |
| GO:0010627 | regulation of intracellular protein kinase cascade | 365 | 2 | 1 | 1.005025 | 0.446429 | 0.45562 | 0.646366 |
| GO:0010948 | negative regulation of cell cycle process | 69 | 2 | 1 | 1.005025 | 0.446429 | 0.45562 | 0.646366 |
| GO:0014706 | striated muscle tissue development | 163 | 2 | 1 | 1.005025 | 0.446429 | 0.45562 | 0.646366 |
| GO:0015276 | ligand-gated ion channel activity | 126 | 2 | 1 | 1.005025 | 0.446429 | 0.45562 | 0.646366 |
| GO:0016023 | cytoplasmic membrane-bounded vesicle | 698 | 2 | 1 | 1.005025 | 0.446429 | 0.45562 | 0.646366 |
| GO:0016051 | carbohydrate biosynthetic process | 187 | 2 | 1 | 1.005025 | 0.446429 | 0.45562 | 0.646366 |
| GO:0016055 | Wnt receptor signaling pathway | 216 | 2 | 1 | 1.005025 | 0.446429 | 0.45562 | 0.646366 |
| GO:0016811 | hydrolase activity, acting on carbon-nitrogen (but not peptide) bonds, in linear amides | 60 | 2 | 1 | 1.005025 | 0.446429 | 0.45562 | 0.646366 |
| GO:0017124 | SH3 domain binding | 111 | 2 | 1 | 1.005025 | 0.446429 | 0.45562 | 0.646366 |
| GO:0018108 | peptidyl-tyrosine phosphorylation | 117 | 2 | 1 | 1.005025 | 0.446429 | 0.45562 | 0.646366 |
| GO:0018212 | peptidyl-tyrosine modification | 119 | 2 | 1 | 1.005025 | 0.446429 | 0.45562 | 0.646366 |
| GO:0019748 | secondary metabolic process | 69 | 2 | 1 | 1.005025 | 0.446429 | 0.45562 | 0.646366 |
| GO:0022834 | ligand-gated channel activity | 126 | 2 | 1 | 1.005025 | 0.446429 | 0.45562 | 0.646366 |
| GO:0030163 | protein catabolic process | 402 | 2 | 1 | 1.005025 | 0.446429 | 0.45562 | 0.646366 |
| GO:0030168 | platelet activation | 236 | 2 | 1 | 1.005025 | 0.446429 | 0.45562 | 0.646366 |
| GO:0030247 | polysaccharide binding | 166 | 2 | 1 | 1.005025 | 0.446429 | 0.45562 | 0.646366 |
| GO:0031410 | cytoplasmic vesicle | 741 | 2 | 1 | 1.005025 | 0.446429 | 0.45562 | 0.646366 |
| GO:0031988 | membrane-bounded vesicle | 711 | 2 | 1 | 1.005025 | 0.446429 | 0.45562 | 0.646366 |
| GO:0032101 | regulation of response to external stimulus | 219 | 2 | 1 | 1.005025 | 0.446429 | 0.45562 | 0.646366 |
| GO:0032582 | negative regulation of gene-specific transcription | 179 | 2 | 1 | 1.005025 | 0.446429 | 0.45562 | 0.646366 |
| GO:0035023 | regulation of Rho protein signal transduction | 111 | 2 | 1 | 1.005025 | 0.446429 | 0.45562 | 0.646366 |
| GO:0042113 | B cell activation | 125 | 2 | 1 | 1.005025 | 0.446429 | 0.45562 | 0.646366 |
| GO:0042445 | hormone metabolic process | 160 | 2 | 1 | 1.005025 | 0.446429 | 0.45562 | 0.646366 |
| GO:0042598 | vesicular fraction | 251 | 2 | 1 | 1.005025 | 0.446429 | 0.45562 | 0.646366 |
| GO:0043062 | extracellular structure organization | 170 | 2 | 1 | 1.005025 | 0.446429 | 0.45562 | 0.646366 |
| GO:0043066 | negative regulation of apoptosis | 429 | 2 | 1 | 1.005025 | 0.446429 | 0.45562 | 0.646366 |
| GO:0043069 | negative regulation of programmed cell death | 434 | 2 | 1 | 1.005025 | 0.446429 | 0.45562 | 0.646366 |
| GO:0044092 | negative regulation of molecular function | 448 | 2 | 1 | 1.005025 | 0.446429 | 0.45562 | 0.646366 |
| GO:0044419 | interspecies interaction between organisms | 354 | 2 | 1 | 1.005025 | 0.446429 | 0.45562 | 0.646366 |
| GO:0044420 | extracellular matrix part | 115 | 2 | 1 | 1.005025 | 0.446429 | 0.45562 | 0.646366 |
| GO:0048011 | nerve growth factor receptor signaling pathway | 219 | 2 | 1 | 1.005025 | 0.446429 | 0.45562 | 0.646366 |
| GO:0048285 | organelle fission | 333 | 2 | 1 | 1.005025 | 0.446429 | 0.45562 | 0.646366 |
| GO:0048741 | skeletal muscle fiber development | 46 | 2 | 1 | 1.005025 | 0.446429 | 0.45562 | 0.646366 |
| GO:0048747 | muscle fiber development | 53 | 2 | 1 | 1.005025 | 0.446429 | 0.45562 | 0.646366 |
| GO:0050795 | regulation of behavior | 80 | 2 | 1 | 1.005025 | 0.446429 | 0.45562 | 0.646366 |
| GO:0050851 | antigen receptor-mediated signaling pathway | 109 | 2 | 1 | 1.005025 | 0.446429 | 0.45562 | 0.646366 |
| GO:0050852 | T cell receptor signaling pathway | 89 | 2 | 1 | 1.005025 | 0.446429 | 0.45562 | 0.646366 |
| GO:0050918 | positive chemotaxis | 33 | 2 | 1 | 1.005025 | 0.446429 | 0.45562 | 0.646366 |
| GO:0050920 | regulation of chemotaxis | 59 | 2 | 1 | 1.005025 | 0.446429 | 0.45562 | 0.646366 |
| GO:0051046 | regulation of secretion | 300 | 2 | 1 | 1.005025 | 0.446429 | 0.45562 | 0.646366 |
| GO:0051146 | striated muscle cell differentiation | 104 | 2 | 1 | 1.005025 | 0.446429 | 0.45562 | 0.646366 |
| GO:0055001 | muscle cell development | 77 | 2 | 1 | 1.005025 | 0.446429 | 0.45562 | 0.646366 |
| GO:0055002 | striated muscle cell development | 72 | 2 | 1 | 1.005025 | 0.446429 | 0.45562 | 0.646366 |
| GO:0060537 | muscle tissue development | 173 | 2 | 1 | 1.005025 | 0.446429 | 0.45562 | 0.646366 |
| GO:0060538 | skeletal muscle organ development | 95 | 2 | 1 | 1.005025 | 0.446429 | 0.45562 | 0.646366 |
| GO:0060548 | negative regulation of cell death | 446 | 2 | 1 | 1.005025 | 0.446429 | 0.45562 | 0.646366 |
| KEGG:04512 | KEGG: ECM-receptor interaction | 84 | 2 | 1 | 1.005025 | 0.446429 | 0.45562 | 0.646366 |
| KEGG:04914 | KEGG: Progesterone-mediated oocyte maturation | 87 | 2 | 1 | 1.005025 | 0.446429 | 0.45562 | 0.646366 |
| KEGG:05100 | KEGG: Bacterial invasion of epithelial cells | 73 | 2 | 1 | 1.005025 | 0.446429 | 0.45562 | 0.646366 |
| KEGG:05222 | KEGG: Small cell lung cancer | 84 | 2 | 1 | 1.005025 | 0.446429 | 0.45562 | 0.646366 |
| PF00018 | PFAM: SH3 domain | 163 | 2 | 1 | 1.005025 | 0.446429 | 0.45562 | 0.646366 |
| PF00028 | PFAM: Cadherin domain | 117 | 2 | 1 | 1.005025 | 0.446429 | 0.45562 | 0.646366 |
| PF00053 | PFAM: Laminin EGF-like (Domains III and V) | 34 | 2 | 1 | 1.005025 | 0.446429 | 0.45562 | 0.646366 |
| PF00621 | PFAM: RhoGEF domain | 71 | 2 | 1 | 1.005025 | 0.446429 | 0.45562 | 0.646366 |
| PF07679 | PFAM: Immunoglobulin I-set domain | 201 | 2 | 1 | 1.005025 | 0.446429 | 0.45562 | 0.646366 |
| REACT:1048 | REACT: tRNA Aminoacylation | 42 | 2 | 1 | 1.005025 | 0.446429 | 0.45562 | 0.646366 |
| REACT:179 | REACT: DNA Replication | 200 | 2 | 1 | 1.005025 | 0.446429 | 0.45562 | 0.646366 |
| REACT:489 | REACT: M Phase | 96 | 2 | 1 | 1.005025 | 0.446429 | 0.45562 | 0.646366 |
| REACT:742 | REACT: Platelet Activation | 244 | 2 | 1 | 1.005025 | 0.446429 | 0.45562 | 0.646366 |
| REACT:762 | REACT: Mitotic Prometaphase | 92 | 2 | 1 | 1.005025 | 0.446429 | 0.45562 | 0.646366 |
| REACT:787 | REACT: Mitotic M-M/G1 phases | 178 | 2 | 1 | 1.005025 | 0.446429 | 0.45562 | 0.646366 |
| BIOC:131 | BIOC: BCR Pathway | 34 | 1 | 0 | 0.502513 | 0 | 0.470449 | 0.692903 |
| BIOC:134 | BIOC: IL2RB Pathway | 35 | 1 | 0 | 0.502513 | 0 | 0.470449 | 0.692903 |
| BIOC:157 | BIOC: FMLP Pathway | 39 | 1 | 0 | 0.502513 | 0 | 0.470449 | 0.692903 |
| BIOC:185 | BIOC: BIOPEPTIDES Pathway | 37 | 1 | 0 | 0.502513 | 0 | 0.470449 | 0.692903 |
| BIOC:189 | BIOC: EGF Pathway | 26 | 1 | 0 | 0.502513 | 0 | 0.470449 | 0.692903 |
| BIOC:193 | BIOC: PDGF Pathway | 26 | 1 | 0 | 0.502513 | 0 | 0.470449 | 0.692903 |
| BIOC:203 | BIOC: MAPK Pathway | 89 | 1 | 0 | 0.502513 | 0 | 0.470449 | 0.692903 |
| BIOC:215 | BIOC: GPCR Pathway | 34 | 1 | 0 | 0.502513 | 0 | 0.470449 | 0.692903 |
| BIOC:225 | BIOC: KERATINOCYTE Pathway | 45 | 1 | 0 | 0.502513 | 0 | 0.470449 | 0.692903 |
| BIOC:30 | BIOC: ERK Pathway | 29 | 1 | 0 | 0.502513 | 0 | 0.470449 | 0.692903 |
| BIOC:39 | BIOC: FCER1 Pathway | 37 | 1 | 0 | 0.502513 | 0 | 0.470449 | 0.692903 |
| BIOC:45 | BIOC: NFAT Pathway | 52 | 1 | 0 | 0.502513 | 0 | 0.470449 | 0.692903 |
| BIOC:47 | BIOC: GH Pathway | 25 | 1 | 0 | 0.502513 | 0 | 0.470449 | 0.692903 |
| BIOC:5 | BIOC: TCR Pathway | 42 | 1 | 0 | 0.502513 | 0 | 0.470449 | 0.692903 |
| BIOC:73 | BIOC: AT1R Pathway | 34 | 1 | 0 | 0.502513 | 0 | 0.470449 | 0.692903 |
| GO:0000041 | transition metal ion transport | 102 | 1 | 0 | 0.502513 | 0 | 0.470449 | 0.692903 |
| GO:0000077 | DNA damage checkpoint | 110 | 1 | 0 | 0.502513 | 0 | 0.470449 | 0.692903 |
| GO:0000079 | regulation of cyclin-dependent protein kinase activity | 61 | 1 | 0 | 0.502513 | 0 | 0.470449 | 0.692903 |
| GO:0000082 | G1/S transition of mitotic cell cycle | 172 | 1 | 0 | 0.502513 | 0 | 0.470449 | 0.692903 |
| GO:0000151 | ubiquitin ligase complex | 139 | 1 | 0 | 0.502513 | 0 | 0.470449 | 0.692903 |
| GO:0000152 | nuclear ubiquitin ligase complex | 29 | 1 | 0 | 0.502513 | 0 | 0.470449 | 0.692903 |
| GO:0000186 | activation of MAPKK activity | 45 | 1 | 0 | 0.502513 | 0 | 0.470449 | 0.692903 |
| GO:0000785 | chromatin | 233 | 1 | 0 | 0.502513 | 0 | 0.470449 | 0.692903 |
| GO:0000790 | nuclear chromatin | 79 | 1 | 0 | 0.502513 | 0 | 0.470449 | 0.692903 |
| GO:0000794 | condensed nuclear chromosome | 56 | 1 | 0 | 0.502513 | 0 | 0.470449 | 0.692903 |
| GO:0001558 | regulation of cell growth | 228 | 1 | 0 | 0.502513 | 0 | 0.470449 | 0.692903 |
| GO:0001654 | eye development | 166 | 1 | 0 | 0.502513 | 0 | 0.470449 | 0.692903 |
| GO:0001656 | metanephros development | 44 | 1 | 0 | 0.502513 | 0 | 0.470449 | 0.692903 |
| GO:0001664 | G-protein-coupled receptor binding | 166 | 1 | 0 | 0.502513 | 0 | 0.470449 | 0.692903 |
| GO:0001667 | ameboidal cell migration | 54 | 1 | 0 | 0.502513 | 0 | 0.470449 | 0.692903 |
| GO:0001726 | ruffle | 84 | 1 | 0 | 0.502513 | 0 | 0.470449 | 0.692903 |
| GO:0001756 | somitogenesis | 45 | 1 | 0 | 0.502513 | 0 | 0.470449 | 0.692903 |
| GO:0001763 | morphogenesis of a branching structure | 138 | 1 | 0 | 0.502513 | 0 | 0.470449 | 0.692903 |
| GO:0001894 | tissue homeostasis | 70 | 1 | 0 | 0.502513 | 0 | 0.470449 | 0.692903 |
| GO:0001934 | positive regulation of protein phosphorylation | 152 | 1 | 0 | 0.502513 | 0 | 0.470449 | 0.692903 |
| GO:0001942 | hair follicle development | 55 | 1 | 0 | 0.502513 | 0 | 0.470449 | 0.692903 |
| GO:0002009 | morphogenesis of an epithelium | 238 | 1 | 0 | 0.502513 | 0 | 0.470449 | 0.692903 |
| GO:0002237 | response to molecule of bacterial origin | 149 | 1 | 0 | 0.502513 | 0 | 0.470449 | 0.692903 |
| GO:0002790 | peptide secretion | 148 | 1 | 0 | 0.502513 | 0 | 0.470449 | 0.692903 |
| GO:0002791 | regulation of peptide secretion | 120 | 1 | 0 | 0.502513 | 0 | 0.470449 | 0.692903 |
| GO:0003001 | generation of a signal involved in cell-cell signaling | 268 | 1 | 0 | 0.502513 | 0 | 0.470449 | 0.692903 |
| GO:0003156 | regulation of organ formation | 26 | 1 | 0 | 0.502513 | 0 | 0.470449 | 0.692903 |
| GO:0003678 | DNA helicase activity | 43 | 1 | 0 | 0.502513 | 0 | 0.470449 | 0.692903 |
| GO:0003707 | steroid hormone receptor activity | 51 | 1 | 0 | 0.502513 | 0 | 0.470449 | 0.692903 |
| GO:0003743 | translation initiation factor activity | 52 | 1 | 0 | 0.502513 | 0 | 0.470449 | 0.692903 |
| GO:0003755 | peptidyl-prolyl cis-trans isomerase activity | 35 | 1 | 0 | 0.502513 | 0 | 0.470449 | 0.692903 |
| GO:0004003 | ATP-dependent DNA helicase activity | 33 | 1 | 0 | 0.502513 | 0 | 0.470449 | 0.692903 |
| GO:0004221 | ubiquitin thiolesterase activity | 73 | 1 | 0 | 0.502513 | 0 | 0.470449 | 0.692903 |
| GO:0004386 | helicase activity | 143 | 1 | 0 | 0.502513 | 0 | 0.470449 | 0.692903 |
| GO:0004402 | histone acetyltransferase activity | 39 | 1 | 0 | 0.502513 | 0 | 0.470449 | 0.692903 |
| GO:0004468 | lysine N-acetyltransferase activity | 39 | 1 | 0 | 0.502513 | 0 | 0.470449 | 0.692903 |
| GO:0004518 | nuclease activity | 161 | 1 | 0 | 0.502513 | 0 | 0.470449 | 0.692903 |
| GO:0004519 | endonuclease activity | 98 | 1 | 0 | 0.502513 | 0 | 0.470449 | 0.692903 |
| GO:0004520 | endodeoxyribonuclease activity | 26 | 1 | 0 | 0.502513 | 0 | 0.470449 | 0.692903 |
| GO:0004536 | deoxyribonuclease activity | 37 | 1 | 0 | 0.502513 | 0 | 0.470449 | 0.692903 |
| GO:0004715 | non-membrane spanning protein tyrosine kinase activity | 40 | 1 | 0 | 0.502513 | 0 | 0.470449 | 0.692903 |
| GO:0004879 | ligand-dependent nuclear receptor activity | 54 | 1 | 0 | 0.502513 | 0 | 0.470449 | 0.692903 |
| GO:0005057 | receptor signaling protein activity | 100 | 1 | 0 | 0.502513 | 0 | 0.470449 | 0.692903 |
| GO:0005201 | extracellular matrix structural constituent | 81 | 1 | 0 | 0.502513 | 0 | 0.470449 | 0.692903 |
| GO:0005581 | collagen | 36 | 1 | 0 | 0.502513 | 0 | 0.470449 | 0.692903 |
| GO:0005681 | spliceosomal complex | 137 | 1 | 0 | 0.502513 | 0 | 0.470449 | 0.692903 |
| GO:0005741 | mitochondrial outer membrane | 100 | 1 | 0 | 0.502513 | 0 | 0.470449 | 0.692903 |
| GO:0005874 | microtubule | 296 | 1 | 0 | 0.502513 | 0 | 0.470449 | 0.692903 |
| GO:0005905 | coated pit | 52 | 1 | 0 | 0.502513 | 0 | 0.470449 | 0.692903 |
| GO:0005930 | axoneme | 58 | 1 | 0 | 0.502513 | 0 | 0.470449 | 0.692903 |
| GO:0005938 | cell cortex | 136 | 1 | 0 | 0.502513 | 0 | 0.470449 | 0.692903 |
| GO:0005996 | monosaccharide metabolic process | 251 | 1 | 0 | 0.502513 | 0 | 0.470449 | 0.692903 |
| GO:0006006 | glucose metabolic process | 178 | 1 | 0 | 0.502513 | 0 | 0.470449 | 0.692903 |
| GO:0006084 | acetyl-CoA metabolic process | 43 | 1 | 0 | 0.502513 | 0 | 0.470449 | 0.692903 |
| GO:0006090 | pyruvate metabolic process | 26 | 1 | 0 | 0.502513 | 0 | 0.470449 | 0.692903 |
| GO:0006275 | regulation of DNA replication | 80 | 1 | 0 | 0.502513 | 0 | 0.470449 | 0.692903 |
| GO:0006282 | regulation of DNA repair | 29 | 1 | 0 | 0.502513 | 0 | 0.470449 | 0.692903 |
| GO:0006323 | DNA packaging | 136 | 1 | 0 | 0.502513 | 0 | 0.470449 | 0.692903 |
| GO:0006383 | transcription from RNA polymerase III promoter | 45 | 1 | 0 | 0.502513 | 0 | 0.470449 | 0.692903 |
| GO:0006413 | translational initiation | 69 | 1 | 0 | 0.502513 | 0 | 0.470449 | 0.692903 |
| GO:0006417 | regulation of translation | 168 | 1 | 0 | 0.502513 | 0 | 0.470449 | 0.692903 |
| GO:0006446 | regulation of translational initiation | 46 | 1 | 0 | 0.502513 | 0 | 0.470449 | 0.692903 |
| GO:0006511 | ubiquitin-dependent protein catabolic process | 317 | 1 | 0 | 0.502513 | 0 | 0.470449 | 0.692903 |
| GO:0006606 | protein import into nucleus | 150 | 1 | 0 | 0.502513 | 0 | 0.470449 | 0.692903 |
| GO:0006611 | protein export from nucleus | 26 | 1 | 0 | 0.502513 | 0 | 0.470449 | 0.692903 |
| GO:0006612 | protein targeting to membrane | 38 | 1 | 0 | 0.502513 | 0 | 0.470449 | 0.692903 |
| GO:0006636 | unsaturated fatty acid biosynthetic process | 42 | 1 | 0 | 0.502513 | 0 | 0.470449 | 0.692903 |
| GO:0006662 | glycerol ether metabolic process | 105 | 1 | 0 | 0.502513 | 0 | 0.470449 | 0.692903 |
| GO:0006690 | icosanoid metabolic process | 53 | 1 | 0 | 0.502513 | 0 | 0.470449 | 0.692903 |
| GO:0006692 | prostanoid metabolic process | 29 | 1 | 0 | 0.502513 | 0 | 0.470449 | 0.692903 |
| GO:0006826 | iron ion transport | 58 | 1 | 0 | 0.502513 | 0 | 0.470449 | 0.692903 |
| GO:0006875 | cellular metal ion homeostasis | 280 | 1 | 0 | 0.502513 | 0 | 0.470449 | 0.692903 |
| GO:0006879 | cellular iron ion homeostasis | 65 | 1 | 0 | 0.502513 | 0 | 0.470449 | 0.692903 |
| GO:0006956 | complement activation | 60 | 1 | 0 | 0.502513 | 0 | 0.470449 | 0.692903 |
| GO:0006959 | humoral immune response | 106 | 1 | 0 | 0.502513 | 0 | 0.470449 | 0.692903 |
| GO:0007015 | actin filament organization | 147 | 1 | 0 | 0.502513 | 0 | 0.470449 | 0.692903 |
| GO:0007126 | meiosis | 122 | 1 | 0 | 0.502513 | 0 | 0.470449 | 0.692903 |
| GO:0007163 | establishment or maintenance of cell polarity | 62 | 1 | 0 | 0.502513 | 0 | 0.470449 | 0.692903 |
| GO:0007178 | transmembrane receptor protein serine/threonine kinase signaling pathway | 181 | 1 | 0 | 0.502513 | 0 | 0.470449 | 0.692903 |
| GO:0007215 | glutamate signaling pathway | 25 | 1 | 0 | 0.502513 | 0 | 0.470449 | 0.692903 |
| GO:0007224 | smoothened signaling pathway | 49 | 1 | 0 | 0.502513 | 0 | 0.470449 | 0.692903 |
| GO:0007254 | JNK cascade | 130 | 1 | 0 | 0.502513 | 0 | 0.470449 | 0.692903 |
| GO:0007257 | activation of JUN kinase activity | 28 | 1 | 0 | 0.502513 | 0 | 0.470449 | 0.692903 |
| GO:0007270 | nerve-nerve synaptic transmission | 55 | 1 | 0 | 0.502513 | 0 | 0.470449 | 0.692903 |
| GO:0007281 | germ cell development | 100 | 1 | 0 | 0.502513 | 0 | 0.470449 | 0.692903 |
| GO:0007286 | spermatid development | 58 | 1 | 0 | 0.502513 | 0 | 0.470449 | 0.692903 |
| GO:0007423 | sensory organ development | 276 | 1 | 0 | 0.502513 | 0 | 0.470449 | 0.692903 |
| GO:0007431 | salivary gland development | 32 | 1 | 0 | 0.502513 | 0 | 0.470449 | 0.692903 |
| GO:0007435 | salivary gland morphogenesis | 29 | 1 | 0 | 0.502513 | 0 | 0.470449 | 0.692903 |
| GO:0007589 | body fluid secretion | 67 | 1 | 0 | 0.502513 | 0 | 0.470449 | 0.692903 |
| GO:0008026 | ATP-dependent helicase activity | 109 | 1 | 0 | 0.502513 | 0 | 0.470449 | 0.692903 |
| GO:0008066 | glutamate receptor activity | 29 | 1 | 0 | 0.502513 | 0 | 0.470449 | 0.692903 |
| GO:0008080 | N-acetyltransferase activity | 67 | 1 | 0 | 0.502513 | 0 | 0.470449 | 0.692903 |
| GO:0008094 | DNA-dependent ATPase activity | 70 | 1 | 0 | 0.502513 | 0 | 0.470449 | 0.692903 |
| GO:0008135 | translation factor activity, nucleic acid binding | 85 | 1 | 0 | 0.502513 | 0 | 0.470449 | 0.692903 |
| GO:0008234 | cysteine-type peptidase activity | 143 | 1 | 0 | 0.502513 | 0 | 0.470449 | 0.692903 |
| GO:0008380 | RNA splicing | 300 | 1 | 0 | 0.502513 | 0 | 0.470449 | 0.692903 |
| GO:0008415 | acyltransferase activity | 210 | 1 | 0 | 0.502513 | 0 | 0.470449 | 0.692903 |
| GO:0008543 | fibroblast growth factor receptor signaling pathway | 70 | 1 | 0 | 0.502513 | 0 | 0.470449 | 0.692903 |
| GO:0008544 | epidermis development | 217 | 1 | 0 | 0.502513 | 0 | 0.470449 | 0.692903 |
| GO:0008584 | male gonad development | 70 | 1 | 0 | 0.502513 | 0 | 0.470449 | 0.692903 |
| GO:0008589 | regulation of smoothened signaling pathway | 31 | 1 | 0 | 0.502513 | 0 | 0.470449 | 0.692903 |
| GO:0009063 | cellular amino acid catabolic process | 88 | 1 | 0 | 0.502513 | 0 | 0.470449 | 0.692903 |
| GO:0009108 | coenzyme biosynthetic process | 93 | 1 | 0 | 0.502513 | 0 | 0.470449 | 0.692903 |
| GO:0009152 | purine ribonucleotide biosynthetic process | 100 | 1 | 0 | 0.502513 | 0 | 0.470449 | 0.692903 |
| GO:0009310 | amine catabolic process | 96 | 1 | 0 | 0.502513 | 0 | 0.470449 | 0.692903 |
| GO:0009798 | axis specification | 57 | 1 | 0 | 0.502513 | 0 | 0.470449 | 0.692903 |
| GO:0009880 | embryonic pattern specification | 44 | 1 | 0 | 0.502513 | 0 | 0.470449 | 0.692903 |
| GO:0009896 | positive regulation of catabolic process | 83 | 1 | 0 | 0.502513 | 0 | 0.470449 | 0.692903 |
| GO:0009914 | hormone transport | 184 | 1 | 0 | 0.502513 | 0 | 0.470449 | 0.692903 |
| GO:0009952 | anterior/posterior pattern formation | 160 | 1 | 0 | 0.502513 | 0 | 0.470449 | 0.692903 |
| GO:0009954 | proximal/distal pattern formation | 28 | 1 | 0 | 0.502513 | 0 | 0.470449 | 0.692903 |
| GO:0010001 | glial cell differentiation | 84 | 1 | 0 | 0.502513 | 0 | 0.470449 | 0.692903 |
| GO:0010466 | negative regulation of peptidase activity | 61 | 1 | 0 | 0.502513 | 0 | 0.470449 | 0.692903 |
| GO:0010552 | positive regulation of gene-specific transcription from RNA polymerase II promoter | 148 | 1 | 0 | 0.502513 | 0 | 0.470449 | 0.692903 |
| GO:0010562 | positive regulation of phosphorus metabolic process | 171 | 1 | 0 | 0.502513 | 0 | 0.470449 | 0.692903 |
| GO:0010565 | regulation of cellular ketone metabolic process | 135 | 1 | 0 | 0.502513 | 0 | 0.470449 | 0.692903 |
| GO:0010608 | posttranscriptional regulation of gene expression | 276 | 1 | 0 | 0.502513 | 0 | 0.470449 | 0.692903 |
| GO:0010648 | negative regulation of cell communication | 412 | 1 | 0 | 0.502513 | 0 | 0.470449 | 0.692903 |
| GO:0010951 | negative regulation of endopeptidase activity | 32 | 1 | 0 | 0.502513 | 0 | 0.470449 | 0.692903 |
| GO:0014070 | response to organic cyclic compound | 178 | 1 | 0 | 0.502513 | 0 | 0.470449 | 0.692903 |
| GO:0015682 | ferric iron transport | 30 | 1 | 0 | 0.502513 | 0 | 0.470449 | 0.692903 |
| GO:0015833 | peptide transport | 159 | 1 | 0 | 0.502513 | 0 | 0.470449 | 0.692903 |
| GO:0016032 | viral reproduction | 415 | 1 | 0 | 0.502513 | 0 | 0.470449 | 0.692903 |
| GO:0016054 | organic acid catabolic process | 152 | 1 | 0 | 0.502513 | 0 | 0.470449 | 0.692903 |
| GO:0016197 | endosome transport | 88 | 1 | 0 | 0.502513 | 0 | 0.470449 | 0.692903 |
| GO:0016324 | apical plasma membrane | 174 | 1 | 0 | 0.502513 | 0 | 0.470449 | 0.692903 |
| GO:0016331 | morphogenesis of embryonic epithelium | 87 | 1 | 0 | 0.502513 | 0 | 0.470449 | 0.692903 |
| GO:0016407 | acetyltransferase activity | 81 | 1 | 0 | 0.502513 | 0 | 0.470449 | 0.692903 |
| GO:0016410 | N-acyltransferase activity | 82 | 1 | 0 | 0.502513 | 0 | 0.470449 | 0.692903 |
| GO:0016563 | transcription activator activity | 319 | 1 | 0 | 0.502513 | 0 | 0.470449 | 0.692903 |
| GO:0016591 | DNA-directed RNA polymerase II, holoenzyme | 77 | 1 | 0 | 0.502513 | 0 | 0.470449 | 0.692903 |
| GO:0016709 | oxidoreductase activity, acting on paired donors, with incorporation or reduction of molecular oxygen, NADH or NADPH as one donor, and incorporation of one atom of oxygen | 27 | 1 | 0 | 0.502513 | 0 | 0.470449 | 0.692903 |
| GO:0016712 | oxidoreductase activity, acting on paired donors, with incorporation or reduction of molecular oxygen, reduced flavin or flavoprotein as one donor, and incorporation of one atom of oxygen | 26 | 1 | 0 | 0.502513 | 0 | 0.470449 | 0.692903 |
| GO:0016746 | transferase activity, transferring acyl groups | 220 | 1 | 0 | 0.502513 | 0 | 0.470449 | 0.692903 |
| GO:0016747 | transferase activity, transferring acyl groups other than amino-acyl groups | 211 | 1 | 0 | 0.502513 | 0 | 0.470449 | 0.692903 |
| GO:0016790 | thiolester hydrolase activity | 98 | 1 | 0 | 0.502513 | 0 | 0.470449 | 0.692903 |
| GO:0016829 | lyase activity | 162 | 1 | 0 | 0.502513 | 0 | 0.470449 | 0.692903 |
| GO:0016835 | carbon-oxygen lyase activity | 56 | 1 | 0 | 0.502513 | 0 | 0.470449 | 0.692903 |
| GO:0016836 | hydro-lyase activity | 41 | 1 | 0 | 0.502513 | 0 | 0.470449 | 0.692903 |
| GO:0016859 | cis-trans isomerase activity | 37 | 1 | 0 | 0.502513 | 0 | 0.470449 | 0.692903 |
| GO:0016893 | endonuclease activity, active with either ribo- or deoxyribonucleic acids and producing 5'-phosphomonoesters | 37 | 1 | 0 | 0.502513 | 0 | 0.470449 | 0.692903 |
| GO:0017144 | drug metabolic process | 25 | 1 | 0 | 0.502513 | 0 | 0.470449 | 0.692903 |
| GO:0018105 | peptidyl-serine phosphorylation | 67 | 1 | 0 | 0.502513 | 0 | 0.470449 | 0.692903 |
| GO:0018107 | peptidyl-threonine phosphorylation | 28 | 1 | 0 | 0.502513 | 0 | 0.470449 | 0.692903 |
| GO:0018209 | peptidyl-serine modification | 76 | 1 | 0 | 0.502513 | 0 | 0.470449 | 0.692903 |
| GO:0018210 | peptidyl-threonine modification | 31 | 1 | 0 | 0.502513 | 0 | 0.470449 | 0.692903 |
| GO:0018904 | organic ether metabolic process | 106 | 1 | 0 | 0.502513 | 0 | 0.470449 | 0.692903 |
| GO:0019058 | viral infectious cycle | 203 | 1 | 0 | 0.502513 | 0 | 0.470449 | 0.692903 |
| GO:0019080 | viral genome expression | 140 | 1 | 0 | 0.502513 | 0 | 0.470449 | 0.692903 |
| GO:0019083 | viral transcription | 140 | 1 | 0 | 0.502513 | 0 | 0.470449 | 0.692903 |
| GO:0019207 | kinase regulator activity | 105 | 1 | 0 | 0.502513 | 0 | 0.470449 | 0.692903 |
| GO:0019318 | hexose metabolic process | 220 | 1 | 0 | 0.502513 | 0 | 0.470449 | 0.692903 |
| GO:0019717 | synaptosome | 102 | 1 | 0 | 0.502513 | 0 | 0.470449 | 0.692903 |
| GO:0019825 | oxygen binding | 45 | 1 | 0 | 0.502513 | 0 | 0.470449 | 0.692903 |
| GO:0019827 | stem cell maintenance | 31 | 1 | 0 | 0.502513 | 0 | 0.470449 | 0.692903 |
| GO:0019887 | protein kinase regulator activity | 92 | 1 | 0 | 0.502513 | 0 | 0.470449 | 0.692903 |
| GO:0019902 | phosphatase binding | 55 | 1 | 0 | 0.502513 | 0 | 0.470449 | 0.692903 |
| GO:0019903 | protein phosphatase binding | 49 | 1 | 0 | 0.502513 | 0 | 0.470449 | 0.692903 |
| GO:0019941 | modification-dependent protein catabolic process | 323 | 1 | 0 | 0.502513 | 0 | 0.470449 | 0.692903 |
| GO:0021536 | diencephalon development | 40 | 1 | 0 | 0.502513 | 0 | 0.470449 | 0.692903 |
| GO:0021700 | developmental maturation | 102 | 1 | 0 | 0.502513 | 0 | 0.470449 | 0.692903 |
| GO:0021915 | neural tube development | 87 | 1 | 0 | 0.502513 | 0 | 0.470449 | 0.692903 |
| GO:0021983 | pituitary gland development | 27 | 1 | 0 | 0.502513 | 0 | 0.470449 | 0.692903 |
| GO:0022404 | molting cycle process | 55 | 1 | 0 | 0.502513 | 0 | 0.470449 | 0.692903 |
| GO:0022405 | hair cycle process | 55 | 1 | 0 | 0.502513 | 0 | 0.470449 | 0.692903 |
| GO:0022406 | membrane docking | 31 | 1 | 0 | 0.502513 | 0 | 0.470449 | 0.692903 |
| GO:0022415 | viral reproductive process | 224 | 1 | 0 | 0.502513 | 0 | 0.470449 | 0.692903 |
| GO:0022600 | digestive system process | 47 | 1 | 0 | 0.502513 | 0 | 0.470449 | 0.692903 |
| GO:0022612 | gland morphogenesis | 84 | 1 | 0 | 0.502513 | 0 | 0.470449 | 0.692903 |
| GO:0023061 | signal release | 268 | 1 | 0 | 0.502513 | 0 | 0.470449 | 0.692903 |
| GO:0030003 | cellular cation homeostasis | 311 | 1 | 0 | 0.502513 | 0 | 0.470449 | 0.692903 |
| GO:0030010 | establishment of cell polarity | 30 | 1 | 0 | 0.502513 | 0 | 0.470449 | 0.692903 |
| GO:0030072 | peptide hormone secretion | 144 | 1 | 0 | 0.502513 | 0 | 0.470449 | 0.692903 |
| GO:0030073 | insulin secretion | 132 | 1 | 0 | 0.502513 | 0 | 0.470449 | 0.692903 |
| GO:0030139 | endocytic vesicle | 80 | 1 | 0 | 0.502513 | 0 | 0.470449 | 0.692903 |
| GO:0030165 | PDZ domain binding | 70 | 1 | 0 | 0.502513 | 0 | 0.470449 | 0.692903 |
| GO:0030177 | positive regulation of Wnt receptor signaling pathway | 42 | 1 | 0 | 0.502513 | 0 | 0.470449 | 0.692903 |
| GO:0030261 | chromosome condensation | 26 | 1 | 0 | 0.502513 | 0 | 0.470449 | 0.692903 |
| GO:0030323 | respiratory tube development | 104 | 1 | 0 | 0.502513 | 0 | 0.470449 | 0.692903 |
| GO:0030324 | lung development | 101 | 1 | 0 | 0.502513 | 0 | 0.470449 | 0.692903 |
| GO:0030336 | negative regulation of cell migration | 76 | 1 | 0 | 0.502513 | 0 | 0.470449 | 0.692903 |
| GO:0030426 | growth cone | 68 | 1 | 0 | 0.502513 | 0 | 0.470449 | 0.692903 |
| GO:0030427 | site of polarized growth | 70 | 1 | 0 | 0.502513 | 0 | 0.470449 | 0.692903 |
| GO:0030850 | prostate gland development | 42 | 1 | 0 | 0.502513 | 0 | 0.470449 | 0.692903 |
| GO:0030855 | epithelial cell differentiation | 206 | 1 | 0 | 0.502513 | 0 | 0.470449 | 0.692903 |
| GO:0030879 | mammary gland development | 93 | 1 | 0 | 0.502513 | 0 | 0.470449 | 0.692903 |
| GO:0030900 | forebrain development | 172 | 1 | 0 | 0.502513 | 0 | 0.470449 | 0.692903 |
| GO:0030902 | hindbrain development | 72 | 1 | 0 | 0.502513 | 0 | 0.470449 | 0.692903 |
| GO:0031016 | pancreas development | 143 | 1 | 0 | 0.502513 | 0 | 0.470449 | 0.692903 |
| GO:0031098 | stress-activated protein kinase signaling cascade | 168 | 1 | 0 | 0.502513 | 0 | 0.470449 | 0.692903 |
| GO:0031099 | regeneration | 92 | 1 | 0 | 0.502513 | 0 | 0.470449 | 0.692903 |
| GO:0031123 | RNA 3'-end processing | 81 | 1 | 0 | 0.502513 | 0 | 0.470449 | 0.692903 |
| GO:0031124 | mRNA 3'-end processing | 69 | 1 | 0 | 0.502513 | 0 | 0.470449 | 0.692903 |
| GO:0031128 | developmental induction | 31 | 1 | 0 | 0.502513 | 0 | 0.470449 | 0.692903 |
| GO:0031252 | cell leading edge | 179 | 1 | 0 | 0.502513 | 0 | 0.470449 | 0.692903 |
| GO:0031401 | positive regulation of protein modification process | 279 | 1 | 0 | 0.502513 | 0 | 0.470449 | 0.692903 |
| GO:0031513 | nonmotile primary cilium | 40 | 1 | 0 | 0.502513 | 0 | 0.470449 | 0.692903 |
| GO:0031570 | DNA integrity checkpoint | 117 | 1 | 0 | 0.502513 | 0 | 0.470449 | 0.692903 |
| GO:0031625 | ubiquitin protein ligase binding | 79 | 1 | 0 | 0.502513 | 0 | 0.470449 | 0.692903 |
| GO:0031645 | negative regulation of neurological system process | 31 | 1 | 0 | 0.502513 | 0 | 0.470449 | 0.692903 |
| GO:0031901 | early endosome membrane | 53 | 1 | 0 | 0.502513 | 0 | 0.470449 | 0.692903 |
| GO:0032088 | negative regulation of NF-kappaB transcription factor activity | 38 | 1 | 0 | 0.502513 | 0 | 0.470449 | 0.692903 |
| GO:0032102 | negative regulation of response to external stimulus | 74 | 1 | 0 | 0.502513 | 0 | 0.470449 | 0.692903 |
| GO:0032147 | activation of protein kinase activity | 149 | 1 | 0 | 0.502513 | 0 | 0.470449 | 0.692903 |
| GO:0032270 | positive regulation of cellular protein metabolic process | 348 | 1 | 0 | 0.502513 | 0 | 0.470449 | 0.692903 |
| GO:0032355 | response to estradiol stimulus | 82 | 1 | 0 | 0.502513 | 0 | 0.470449 | 0.692903 |
| GO:0032496 | response to lipopolysaccharide | 136 | 1 | 0 | 0.502513 | 0 | 0.470449 | 0.692903 |
| GO:0032947 | protein complex scaffold | 30 | 1 | 0 | 0.502513 | 0 | 0.470449 | 0.692903 |
| GO:0033044 | regulation of chromosome organization | 51 | 1 | 0 | 0.502513 | 0 | 0.470449 | 0.692903 |
| GO:0033559 | unsaturated fatty acid metabolic process | 56 | 1 | 0 | 0.502513 | 0 | 0.470449 | 0.692903 |
| GO:0033572 | transferrin transport | 30 | 1 | 0 | 0.502513 | 0 | 0.470449 | 0.692903 |
| GO:0033865 | nucleoside bisphosphate metabolic process | 25 | 1 | 0 | 0.502513 | 0 | 0.470449 | 0.692903 |
| GO:0034220 | ion transmembrane transport | 235 | 1 | 0 | 0.502513 | 0 | 0.470449 | 0.692903 |
| GO:0034339 | regulation of transcription from RNA polymerase II promoter by nuclear hormone receptor | 58 | 1 | 0 | 0.502513 | 0 | 0.470449 | 0.692903 |
| GO:0034762 | regulation of transmembrane transport | 145 | 1 | 0 | 0.502513 | 0 | 0.470449 | 0.692903 |
| GO:0034763 | negative regulation of transmembrane transport | 32 | 1 | 0 | 0.502513 | 0 | 0.470449 | 0.692903 |
| GO:0035085 | cilium axoneme | 46 | 1 | 0 | 0.502513 | 0 | 0.470449 | 0.692903 |
| GO:0035148 | tube formation | 71 | 1 | 0 | 0.502513 | 0 | 0.470449 | 0.692903 |
| GO:0035239 | tube morphogenesis | 189 | 1 | 0 | 0.502513 | 0 | 0.470449 | 0.692903 |
| GO:0035249 | synaptic transmission, glutamatergic | 28 | 1 | 0 | 0.502513 | 0 | 0.470449 | 0.692903 |
| GO:0035265 | organ growth | 45 | 1 | 0 | 0.502513 | 0 | 0.470449 | 0.692903 |
| GO:0035272 | exocrine system development | 42 | 1 | 0 | 0.502513 | 0 | 0.470449 | 0.692903 |
| GO:0035282 | segmentation | 67 | 1 | 0 | 0.502513 | 0 | 0.470449 | 0.692903 |
| GO:0035295 | tube development | 281 | 1 | 0 | 0.502513 | 0 | 0.470449 | 0.692903 |
| GO:0040008 | regulation of growth | 389 | 1 | 0 | 0.502513 | 0 | 0.470449 | 0.692903 |
| GO:0040013 | negative regulation of locomotion | 90 | 1 | 0 | 0.502513 | 0 | 0.470449 | 0.692903 |
| GO:0042063 | gliogenesis | 98 | 1 | 0 | 0.502513 | 0 | 0.470449 | 0.692903 |
| GO:0042147 | retrograde transport, endosome to Golgi | 25 | 1 | 0 | 0.502513 | 0 | 0.470449 | 0.692903 |
| GO:0042176 | regulation of protein catabolic process | 97 | 1 | 0 | 0.502513 | 0 | 0.470449 | 0.692903 |
| GO:0042246 | tissue regeneration | 35 | 1 | 0 | 0.502513 | 0 | 0.470449 | 0.692903 |
| GO:0042303 | molting cycle | 57 | 1 | 0 | 0.502513 | 0 | 0.470449 | 0.692903 |
| GO:0042306 | regulation of protein import into nucleus | 83 | 1 | 0 | 0.502513 | 0 | 0.470449 | 0.692903 |
| GO:0042308 | negative regulation of protein import into nucleus | 29 | 1 | 0 | 0.502513 | 0 | 0.470449 | 0.692903 |
| GO:0042327 | positive regulation of phosphorylation | 168 | 1 | 0 | 0.502513 | 0 | 0.470449 | 0.692903 |
| GO:0042446 | hormone biosynthetic process | 82 | 1 | 0 | 0.502513 | 0 | 0.470449 | 0.692903 |
| GO:0042470 | melanosome | 92 | 1 | 0 | 0.502513 | 0 | 0.470449 | 0.692903 |
| GO:0042471 | ear morphogenesis | 82 | 1 | 0 | 0.502513 | 0 | 0.470449 | 0.692903 |
| GO:0042472 | inner ear morphogenesis | 69 | 1 | 0 | 0.502513 | 0 | 0.470449 | 0.692903 |
| GO:0042475 | odontogenesis of dentine-containing tooth | 44 | 1 | 0 | 0.502513 | 0 | 0.470449 | 0.692903 |
| GO:0042476 | odontogenesis | 77 | 1 | 0 | 0.502513 | 0 | 0.470449 | 0.692903 |
| GO:0042493 | response to drug | 288 | 1 | 0 | 0.502513 | 0 | 0.470449 | 0.692903 |
| GO:0042623 | ATPase activity, coupled | 280 | 1 | 0 | 0.502513 | 0 | 0.470449 | 0.692903 |
| GO:0042633 | hair cycle | 57 | 1 | 0 | 0.502513 | 0 | 0.470449 | 0.692903 |
| GO:0043010 | camera-type eye development | 129 | 1 | 0 | 0.502513 | 0 | 0.470449 | 0.692903 |
| GO:0043025 | neuronal cell body | 180 | 1 | 0 | 0.502513 | 0 | 0.470449 | 0.692903 |
| GO:0043197 | dendritic spine | 50 | 1 | 0 | 0.502513 | 0 | 0.470449 | 0.692903 |
| GO:0043392 | negative regulation of DNA binding | 85 | 1 | 0 | 0.502513 | 0 | 0.470449 | 0.692903 |
| GO:0043410 | positive regulation of MAPKKK cascade | 97 | 1 | 0 | 0.502513 | 0 | 0.470449 | 0.692903 |
| GO:0043433 | negative regulation of transcription factor activity | 75 | 1 | 0 | 0.502513 | 0 | 0.470449 | 0.692903 |
| GO:0043506 | regulation of JUN kinase activity | 54 | 1 | 0 | 0.502513 | 0 | 0.470449 | 0.692903 |
| GO:0043507 | positive regulation of JUN kinase activity | 44 | 1 | 0 | 0.502513 | 0 | 0.470449 | 0.692903 |
| GO:0043583 | ear development | 117 | 1 | 0 | 0.502513 | 0 | 0.470449 | 0.692903 |
| GO:0043627 | response to estrogen stimulus | 134 | 1 | 0 | 0.502513 | 0 | 0.470449 | 0.692903 |
| GO:0043632 | modification-dependent macromolecule catabolic process | 323 | 1 | 0 | 0.502513 | 0 | 0.470449 | 0.692903 |
| GO:0044297 | cell body | 185 | 1 | 0 | 0.502513 | 0 | 0.470449 | 0.692903 |
| GO:0044309 | neuron spine | 53 | 1 | 0 | 0.502513 | 0 | 0.470449 | 0.692903 |
| GO:0044441 | cilium part | 70 | 1 | 0 | 0.502513 | 0 | 0.470449 | 0.692903 |
| GO:0044448 | cell cortex part | 65 | 1 | 0 | 0.502513 | 0 | 0.470449 | 0.692903 |
| GO:0045165 | cell fate commitment | 154 | 1 | 0 | 0.502513 | 0 | 0.470449 | 0.692903 |
| GO:0045168 | cell-cell signaling involved in cell fate commitment | 31 | 1 | 0 | 0.502513 | 0 | 0.470449 | 0.692903 |
| GO:0045177 | apical part of cell | 219 | 1 | 0 | 0.502513 | 0 | 0.470449 | 0.692903 |
| GO:0045178 | basal part of cell | 26 | 1 | 0 | 0.502513 | 0 | 0.470449 | 0.692903 |
| GO:0045444 | fat cell differentiation | 91 | 1 | 0 | 0.502513 | 0 | 0.470449 | 0.692903 |
| GO:0045732 | positive regulation of protein catabolic process | 54 | 1 | 0 | 0.502513 | 0 | 0.470449 | 0.692903 |
| GO:0045740 | positive regulation of DNA replication | 33 | 1 | 0 | 0.502513 | 0 | 0.470449 | 0.692903 |
| GO:0045787 | positive regulation of cell cycle | 74 | 1 | 0 | 0.502513 | 0 | 0.470449 | 0.692903 |
| GO:0045937 | positive regulation of phosphate metabolic process | 171 | 1 | 0 | 0.502513 | 0 | 0.470449 | 0.692903 |
| GO:0045995 | regulation of embryonic development | 42 | 1 | 0 | 0.502513 | 0 | 0.470449 | 0.692903 |
| GO:0046328 | regulation of JNK cascade | 92 | 1 | 0 | 0.502513 | 0 | 0.470449 | 0.692903 |
| GO:0046395 | carboxylic acid catabolic process | 152 | 1 | 0 | 0.502513 | 0 | 0.470449 | 0.692903 |
| GO:0046456 | icosanoid biosynthetic process | 39 | 1 | 0 | 0.502513 | 0 | 0.470449 | 0.692903 |
| GO:0046777 | protein autophosphorylation | 89 | 1 | 0 | 0.502513 | 0 | 0.470449 | 0.692903 |
| GO:0046782 | regulation of viral transcription | 56 | 1 | 0 | 0.502513 | 0 | 0.470449 | 0.692903 |
| GO:0046879 | hormone secretion | 176 | 1 | 0 | 0.502513 | 0 | 0.470449 | 0.692903 |
| GO:0046883 | regulation of hormone secretion | 145 | 1 | 0 | 0.502513 | 0 | 0.470449 | 0.692903 |
| GO:0046888 | negative regulation of hormone secretion | 38 | 1 | 0 | 0.502513 | 0 | 0.470449 | 0.692903 |
| GO:0046982 | protein heterodimerization activity | 228 | 1 | 0 | 0.502513 | 0 | 0.470449 | 0.692903 |
| GO:0048010 | vascular endothelial growth factor receptor signaling pathway | 32 | 1 | 0 | 0.502513 | 0 | 0.470449 | 0.692903 |
| GO:0048144 | fibroblast proliferation | 42 | 1 | 0 | 0.502513 | 0 | 0.470449 | 0.692903 |
| GO:0048145 | regulation of fibroblast proliferation | 41 | 1 | 0 | 0.502513 | 0 | 0.470449 | 0.692903 |
| GO:0048146 | positive regulation of fibroblast proliferation | 32 | 1 | 0 | 0.502513 | 0 | 0.470449 | 0.692903 |
| GO:0048167 | regulation of synaptic plasticity | 49 | 1 | 0 | 0.502513 | 0 | 0.470449 | 0.692903 |
| GO:0048278 | vesicle docking | 26 | 1 | 0 | 0.502513 | 0 | 0.470449 | 0.692903 |
| GO:0048286 | lung alveolus development | 27 | 1 | 0 | 0.502513 | 0 | 0.470449 | 0.692903 |
| GO:0048515 | spermatid differentiation | 61 | 1 | 0 | 0.502513 | 0 | 0.470449 | 0.692903 |
| GO:0048524 | positive regulation of viral reproduction | 62 | 1 | 0 | 0.502513 | 0 | 0.470449 | 0.692903 |
| GO:0048545 | response to steroid hormone stimulus | 264 | 1 | 0 | 0.502513 | 0 | 0.470449 | 0.692903 |
| GO:0048546 | digestive tract morphogenesis | 37 | 1 | 0 | 0.502513 | 0 | 0.470449 | 0.692903 |
| GO:0048565 | digestive tract development | 68 | 1 | 0 | 0.502513 | 0 | 0.470449 | 0.692903 |
| GO:0048645 | organ formation | 39 | 1 | 0 | 0.502513 | 0 | 0.470449 | 0.692903 |
| GO:0048709 | oligodendrocyte differentiation | 33 | 1 | 0 | 0.502513 | 0 | 0.470449 | 0.692903 |
| GO:0048729 | tissue morphogenesis | 303 | 1 | 0 | 0.502513 | 0 | 0.470449 | 0.692903 |
| GO:0048730 | epidermis morphogenesis | 27 | 1 | 0 | 0.502513 | 0 | 0.470449 | 0.692903 |
| GO:0048754 | branching morphogenesis of a tube | 103 | 1 | 0 | 0.502513 | 0 | 0.470449 | 0.692903 |
| GO:0048762 | mesenchymal cell differentiation | 90 | 1 | 0 | 0.502513 | 0 | 0.470449 | 0.692903 |
| GO:0048770 | pigment granule | 92 | 1 | 0 | 0.502513 | 0 | 0.470449 | 0.692903 |
| GO:0048771 | tissue remodeling | 79 | 1 | 0 | 0.502513 | 0 | 0.470449 | 0.692903 |
| GO:0048806 | genitalia development | 29 | 1 | 0 | 0.502513 | 0 | 0.470449 | 0.692903 |
| GO:0048839 | inner ear development | 98 | 1 | 0 | 0.502513 | 0 | 0.470449 | 0.692903 |
| GO:0048863 | stem cell differentiation | 47 | 1 | 0 | 0.502513 | 0 | 0.470449 | 0.692903 |
| GO:0048864 | stem cell development | 34 | 1 | 0 | 0.502513 | 0 | 0.470449 | 0.692903 |
| GO:0048871 | multicellular organismal homeostasis | 103 | 1 | 0 | 0.502513 | 0 | 0.470449 | 0.692903 |
| GO:0050434 | positive regulation of viral transcription | 50 | 1 | 0 | 0.502513 | 0 | 0.470449 | 0.692903 |
| GO:0050673 | epithelial cell proliferation | 161 | 1 | 0 | 0.502513 | 0 | 0.470449 | 0.692903 |
| GO:0050678 | regulation of epithelial cell proliferation | 139 | 1 | 0 | 0.502513 | 0 | 0.470449 | 0.692903 |
| GO:0050679 | positive regulation of epithelial cell proliferation | 79 | 1 | 0 | 0.502513 | 0 | 0.470449 | 0.692903 |
| GO:0050730 | regulation of peptidyl-tyrosine phosphorylation | 86 | 1 | 0 | 0.502513 | 0 | 0.470449 | 0.692903 |
| GO:0050731 | positive regulation of peptidyl-tyrosine phosphorylation | 66 | 1 | 0 | 0.502513 | 0 | 0.470449 | 0.692903 |
| GO:0050792 | regulation of viral reproduction | 73 | 1 | 0 | 0.502513 | 0 | 0.470449 | 0.692903 |
| GO:0050796 | regulation of insulin secretion | 112 | 1 | 0 | 0.502513 | 0 | 0.470449 | 0.692903 |
| GO:0050805 | negative regulation of synaptic transmission | 25 | 1 | 0 | 0.502513 | 0 | 0.470449 | 0.692903 |
| GO:0051048 | negative regulation of secretion | 80 | 1 | 0 | 0.502513 | 0 | 0.470449 | 0.692903 |
| GO:0051054 | positive regulation of DNA metabolic process | 66 | 1 | 0 | 0.502513 | 0 | 0.470449 | 0.692903 |
| GO:0051090 | regulation of transcription factor activity | 232 | 1 | 0 | 0.502513 | 0 | 0.470449 | 0.692903 |
| GO:0051098 | regulation of binding | 305 | 1 | 0 | 0.502513 | 0 | 0.470449 | 0.692903 |
| GO:0051100 | negative regulation of binding | 105 | 1 | 0 | 0.502513 | 0 | 0.470449 | 0.692903 |
| GO:0051101 | regulation of DNA binding | 253 | 1 | 0 | 0.502513 | 0 | 0.470449 | 0.692903 |
| GO:0051168 | nuclear export | 95 | 1 | 0 | 0.502513 | 0 | 0.470449 | 0.692903 |
| GO:0051170 | nuclear import | 153 | 1 | 0 | 0.502513 | 0 | 0.470449 | 0.692903 |
| GO:0051188 | cofactor biosynthetic process | 127 | 1 | 0 | 0.502513 | 0 | 0.470449 | 0.692903 |
| GO:0051271 | negative regulation of cellular component movement | 82 | 1 | 0 | 0.502513 | 0 | 0.470449 | 0.692903 |
| GO:0051321 | meiotic cell cycle | 124 | 1 | 0 | 0.502513 | 0 | 0.470449 | 0.692903 |
| GO:0051327 | M phase of meiotic cell cycle | 122 | 1 | 0 | 0.502513 | 0 | 0.470449 | 0.692903 |
| GO:0051345 | positive regulation of hydrolase activity | 270 | 1 | 0 | 0.502513 | 0 | 0.470449 | 0.692903 |
| GO:0051346 | negative regulation of hydrolase activity | 97 | 1 | 0 | 0.502513 | 0 | 0.470449 | 0.692903 |
| GO:0051603 | proteolysis involved in cellular protein catabolic process | 333 | 1 | 0 | 0.502513 | 0 | 0.470449 | 0.692903 |
| GO:0051789 | response to protein stimulus | 126 | 1 | 0 | 0.502513 | 0 | 0.470449 | 0.692903 |
| GO:0051970 | negative regulation of transmission of nerve impulse | 29 | 1 | 0 | 0.502513 | 0 | 0.470449 | 0.692903 |
| GO:0052547 | regulation of peptidase activity | 154 | 1 | 0 | 0.502513 | 0 | 0.470449 | 0.692903 |
| GO:0052548 | regulation of endopeptidase activity | 147 | 1 | 0 | 0.502513 | 0 | 0.470449 | 0.692903 |
| GO:0055065 | metal ion homeostasis | 289 | 1 | 0 | 0.502513 | 0 | 0.470449 | 0.692903 |
| GO:0055072 | iron ion homeostasis | 69 | 1 | 0 | 0.502513 | 0 | 0.470449 | 0.692903 |
| GO:0055123 | digestive system development | 78 | 1 | 0 | 0.502513 | 0 | 0.470449 | 0.692903 |
| GO:0060070 | canonical Wnt receptor signaling pathway | 124 | 1 | 0 | 0.502513 | 0 | 0.470449 | 0.692903 |
| GO:0060425 | lung morphogenesis | 27 | 1 | 0 | 0.502513 | 0 | 0.470449 | 0.692903 |
| GO:0060429 | epithelium development | 398 | 1 | 0 | 0.502513 | 0 | 0.470449 | 0.692903 |
| GO:0060443 | mammary gland morphogenesis | 36 | 1 | 0 | 0.502513 | 0 | 0.470449 | 0.692903 |
| GO:0060485 | mesenchyme development | 100 | 1 | 0 | 0.502513 | 0 | 0.470449 | 0.692903 |
| GO:0060512 | prostate gland morphogenesis | 28 | 1 | 0 | 0.502513 | 0 | 0.470449 | 0.692903 |
| GO:0060541 | respiratory system development | 119 | 1 | 0 | 0.502513 | 0 | 0.470449 | 0.692903 |
| GO:0060560 | developmental growth involved in morphogenesis | 83 | 1 | 0 | 0.502513 | 0 | 0.470449 | 0.692903 |
| GO:0060562 | epithelial tube morphogenesis | 156 | 1 | 0 | 0.502513 | 0 | 0.470449 | 0.692903 |
| GO:0060606 | tube closure | 45 | 1 | 0 | 0.502513 | 0 | 0.470449 | 0.692903 |
| GO:0060688 | regulation of morphogenesis of a branching structure | 31 | 1 | 0 | 0.502513 | 0 | 0.470449 | 0.692903 |
| GO:0060740 | prostate gland epithelium morphogenesis | 27 | 1 | 0 | 0.502513 | 0 | 0.470449 | 0.692903 |
| GO:0060828 | regulation of canonical Wnt receptor signaling pathway | 85 | 1 | 0 | 0.502513 | 0 | 0.470449 | 0.692903 |
| GO:0061053 | somite development | 48 | 1 | 0 | 0.502513 | 0 | 0.470449 | 0.692903 |
| GO:0061138 | morphogenesis of a branching epithelium | 117 | 1 | 0 | 0.502513 | 0 | 0.470449 | 0.692903 |
| GO:0061180 | mammary gland epithelium development | 43 | 1 | 0 | 0.502513 | 0 | 0.470449 | 0.692903 |
| GO:0070035 | purine NTP-dependent helicase activity | 109 | 1 | 0 | 0.502513 | 0 | 0.470449 | 0.692903 |
| GO:0070302 | regulation of stress-activated protein kinase signaling cascade | 100 | 1 | 0 | 0.502513 | 0 | 0.470449 | 0.692903 |
| GO:0070371 | ERK1 and ERK2 cascade | 54 | 1 | 0 | 0.502513 | 0 | 0.470449 | 0.692903 |
| GO:0070372 | regulation of ERK1 and ERK2 cascade | 47 | 1 | 0 | 0.502513 | 0 | 0.470449 | 0.692903 |
| GO:0070374 | positive regulation of ERK1 and ERK2 cascade | 39 | 1 | 0 | 0.502513 | 0 | 0.470449 | 0.692903 |
| GO:0071103 | DNA conformation change | 157 | 1 | 0 | 0.502513 | 0 | 0.470449 | 0.692903 |
| GO:0072372 | primary cilium | 44 | 1 | 0 | 0.502513 | 0 | 0.470449 | 0.692903 |
| GO:0090046 | regulation of transcription regulator activity | 236 | 1 | 0 | 0.502513 | 0 | 0.470449 | 0.692903 |
| GO:0090048 | negative regulation of transcription regulator activity | 75 | 1 | 0 | 0.502513 | 0 | 0.470449 | 0.692903 |
| GO:0090087 | regulation of peptide transport | 120 | 1 | 0 | 0.502513 | 0 | 0.470449 | 0.692903 |
| GO:0090092 | regulation of transmembrane receptor protein serine/threonine kinase signaling pathway | 116 | 1 | 0 | 0.502513 | 0 | 0.470449 | 0.692903 |
| GO:0090263 | positive regulation of canonical Wnt receptor signaling pathway | 31 | 1 | 0 | 0.502513 | 0 | 0.470449 | 0.692903 |
| GO:0090276 | regulation of peptide hormone secretion | 118 | 1 | 0 | 0.502513 | 0 | 0.470449 | 0.692903 |
| GO:2000027 | regulation of organ morphogenesis | 81 | 1 | 0 | 0.502513 | 0 | 0.470449 | 0.692903 |
| GO:2000146 | negative regulation of cell motility | 76 | 1 | 0 | 0.502513 | 0 | 0.470449 | 0.692903 |
| GO:2000243 | positive regulation of reproductive process | 67 | 1 | 0 | 0.502513 | 0 | 0.470449 | 0.692903 |
| KEGG:00340 | KEGG: Histidine metabolism | 29 | 1 | 0 | 0.502513 | 0 | 0.470449 | 0.692903 |
| KEGG:00591 | KEGG: Linoleic acid metabolism | 29 | 1 | 0 | 0.502513 | 0 | 0.470449 | 0.692903 |
| KEGG:00620 | KEGG: Pyruvate metabolism | 40 | 1 | 0 | 0.502513 | 0 | 0.470449 | 0.692903 |
| KEGG:00830 | KEGG: Retinol metabolism | 65 | 1 | 0 | 0.502513 | 0 | 0.470449 | 0.692903 |
| KEGG:00980 | KEGG: Metabolism of xenobiotics by cytochrome P450 | 71 | 1 | 0 | 0.502513 | 0 | 0.470449 | 0.692903 |
| KEGG:00982 | KEGG: Drug metabolism - cytochrome P450 | 73 | 1 | 0 | 0.502513 | 0 | 0.470449 | 0.692903 |
| KEGG:02010 | KEGG: ABC transporters | 44 | 1 | 0 | 0.502513 | 0 | 0.470449 | 0.692903 |
| KEGG:04012 | KEGG: ErbB signaling pathway | 87 | 1 | 0 | 0.502513 | 0 | 0.470449 | 0.692903 |
| KEGG:04114 | KEGG: Oocyte meiosis | 114 | 1 | 0 | 0.502513 | 0 | 0.470449 | 0.692903 |
| KEGG:04141 | KEGG: Protein processing in endoplasmic reticulum | 167 | 1 | 0 | 0.502513 | 0 | 0.470449 | 0.692903 |
| KEGG:04270 | KEGG: Vascular smooth muscle contraction | 116 | 1 | 0 | 0.502513 | 0 | 0.470449 | 0.692903 |
| KEGG:04370 | KEGG: VEGF signaling pathway | 76 | 1 | 0 | 0.502513 | 0 | 0.470449 | 0.692903 |
| KEGG:04530 | KEGG: Tight junction | 134 | 1 | 0 | 0.502513 | 0 | 0.470449 | 0.692903 |
| KEGG:04540 | KEGG: Gap junction | 90 | 1 | 0 | 0.502513 | 0 | 0.470449 | 0.692903 |
| KEGG:04650 | KEGG: Natural killer cell mediated cytotoxicity | 137 | 1 | 0 | 0.502513 | 0 | 0.470449 | 0.692903 |
| KEGG:04660 | KEGG: T cell receptor signaling pathway | 108 | 1 | 0 | 0.502513 | 0 | 0.470449 | 0.692903 |
| KEGG:04662 | KEGG: B cell receptor signaling pathway | 75 | 1 | 0 | 0.502513 | 0 | 0.470449 | 0.692903 |
| KEGG:04664 | KEGG: Fc epsilon RI signaling pathway | 79 | 1 | 0 | 0.502513 | 0 | 0.470449 | 0.692903 |
| KEGG:04666 | KEGG: Fc gamma R-mediated phagocytosis | 95 | 1 | 0 | 0.502513 | 0 | 0.470449 | 0.692903 |
| KEGG:04670 | KEGG: Leukocyte transendothelial migration | 118 | 1 | 0 | 0.502513 | 0 | 0.470449 | 0.692903 |
| KEGG:04672 | KEGG: Intestinal immune network for IgA production | 49 | 1 | 0 | 0.502513 | 0 | 0.470449 | 0.692903 |
| KEGG:04720 | KEGG: Long-term potentiation | 70 | 1 | 0 | 0.502513 | 0 | 0.470449 | 0.692903 |
| KEGG:04722 | KEGG: Neurotrophin signaling pathway | 126 | 1 | 0 | 0.502513 | 0 | 0.470449 | 0.692903 |
| KEGG:04730 | KEGG: Long-term depression | 70 | 1 | 0 | 0.502513 | 0 | 0.470449 | 0.692903 |
| KEGG:04910 | KEGG: Insulin signaling pathway | 137 | 1 | 0 | 0.502513 | 0 | 0.470449 | 0.692903 |
| KEGG:04912 | KEGG: GnRH signaling pathway | 101 | 1 | 0 | 0.502513 | 0 | 0.470449 | 0.692903 |
| KEGG:04916 | KEGG: Melanogenesis | 102 | 1 | 0 | 0.502513 | 0 | 0.470449 | 0.692903 |
| KEGG:05110 | KEGG: Vibrio cholerae infection | 56 | 1 | 0 | 0.502513 | 0 | 0.470449 | 0.692903 |
| KEGG:05131 | KEGG: Shigellosis | 64 | 1 | 0 | 0.502513 | 0 | 0.470449 | 0.692903 |
| KEGG:05210 | KEGG: Colorectal cancer | 62 | 1 | 0 | 0.502513 | 0 | 0.470449 | 0.692903 |
| KEGG:05211 | KEGG: Renal cell carcinoma | 70 | 1 | 0 | 0.502513 | 0 | 0.470449 | 0.692903 |
| KEGG:05212 | KEGG: Pancreatic cancer | 70 | 1 | 0 | 0.502513 | 0 | 0.470449 | 0.692903 |
| KEGG:05213 | KEGG: Endometrial cancer | 52 | 1 | 0 | 0.502513 | 0 | 0.470449 | 0.692903 |
| KEGG:05214 | KEGG: Glioma | 65 | 1 | 0 | 0.502513 | 0 | 0.470449 | 0.692903 |
| KEGG:05215 | KEGG: Prostate cancer | 89 | 1 | 0 | 0.502513 | 0 | 0.470449 | 0.692903 |
| KEGG:05219 | KEGG: Bladder cancer | 42 | 1 | 0 | 0.502513 | 0 | 0.470449 | 0.692903 |
| KEGG:05220 | KEGG: Chronic myeloid leukemia | 73 | 1 | 0 | 0.502513 | 0 | 0.470449 | 0.692903 |
| KEGG:05221 | KEGG: Acute myeloid leukemia | 60 | 1 | 0 | 0.502513 | 0 | 0.470449 | 0.692903 |
| NCI:11 | Angiopoietin receptor Tie2-mediated signaling | 48 | 1 | 0 | 0.502513 | 0 | 0.470449 | 0.692903 |
| NCI:111 | Insulin Pathway | 43 | 1 | 0 | 0.502513 | 0 | 0.470449 | 0.692903 |
| NCI:112 | Signaling events mediated by Stem cell factor receptor (c-Kit) | 52 | 1 | 0 | 0.502513 | 0 | 0.470449 | 0.692903 |
| NCI:117 | LPA receptor mediated events | 66 | 1 | 0 | 0.502513 | 0 | 0.470449 | 0.692903 |
| NCI:118 | Trk receptor signaling mediated by the MAPK pathway | 34 | 1 | 0 | 0.502513 | 0 | 0.470449 | 0.692903 |
| NCI:120 | mTOR signaling pathway | 70 | 1 | 0 | 0.502513 | 0 | 0.470449 | 0.692903 |
| NCI:133 | Notch signaling pathway | 60 | 1 | 0 | 0.502513 | 0 | 0.470449 | 0.692903 |
| NCI:14 | Nongenotropic Androgen signaling | 30 | 1 | 0 | 0.502513 | 0 | 0.470449 | 0.692903 |
| NCI:144 | Class I PI3K signaling events mediated by Akt | 34 | 1 | 0 | 0.502513 | 0 | 0.470449 | 0.692903 |
| NCI:149 | PLK1 signaling events | 44 | 1 | 0 | 0.502513 | 0 | 0.470449 | 0.692903 |
| NCI:154 | Signaling events mediated by PTP1B | 51 | 1 | 0 | 0.502513 | 0 | 0.470449 | 0.692903 |
| NCI:155 | RAC1 signaling pathway | 54 | 1 | 0 | 0.502513 | 0 | 0.470449 | 0.692903 |
| NCI:159 | Regulation of retinoblastoma protein | 64 | 1 | 0 | 0.502513 | 0 | 0.470449 | 0.692903 |
| NCI:162 | Signaling events regulated by Ret tyrosine kinase | 38 | 1 | 0 | 0.502513 | 0 | 0.470449 | 0.692903 |
| NCI:19 | Arf6 trafficking events | 51 | 1 | 0 | 0.502513 | 0 | 0.470449 | 0.692903 |
| NCI:194 | Neurotrophic factor-mediated Trk receptor signaling | 62 | 1 | 0 | 0.502513 | 0 | 0.470449 | 0.692903 |
| NCI:26 | Integrins in angiogenesis | 47 | 1 | 0 | 0.502513 | 0 | 0.470449 | 0.692903 |
| NCI:27 | Osteopontin-mediated events | 32 | 1 | 0 | 0.502513 | 0 | 0.470449 | 0.692903 |
| NCI:28 | BARD1 signaling events | 29 | 1 | 0 | 0.502513 | 0 | 0.470449 | 0.692903 |
| NCI:29 | BCR signaling pathway | 66 | 1 | 0 | 0.502513 | 0 | 0.470449 | 0.692903 |
| NCI:36 | Downstream signaling in naive CD8+ T cells | 68 | 1 | 0 | 0.502513 | 0 | 0.470449 | 0.692903 |
| NCI:40 | Ceramide signaling pathway | 44 | 1 | 0 | 0.502513 | 0 | 0.470449 | 0.692903 |
| NCI:44 | CXCR3-mediated signaling events | 43 | 1 | 0 | 0.502513 | 0 | 0.470449 | 0.692903 |
| NCI:45 | CXCR4-mediated signaling events | 98 | 1 | 0 | 0.502513 | 0 | 0.470449 | 0.692903 |
| NCI:5 | Posttranslational regulation of adherens junction stability and dissassembly | 49 | 1 | 0 | 0.502513 | 0 | 0.470449 | 0.692903 |
| NCI:54 | EPHB forward signaling | 37 | 1 | 0 | 0.502513 | 0 | 0.470449 | 0.692903 |
| NCI:60 | Plasma membrane estrogen receptor signaling | 39 | 1 | 0 | 0.502513 | 0 | 0.470449 | 0.692903 |
| NCI:62 | ErbB1 downstream signaling | 108 | 1 | 0 | 0.502513 | 0 | 0.470449 | 0.692903 |
| NCI:63 | Internalization of ErbB1 | 39 | 1 | 0 | 0.502513 | 0 | 0.470449 | 0.692903 |
| NCI:70 | Fc-epsilon receptor I signaling in mast cells | 58 | 1 | 0 | 0.502513 | 0 | 0.470449 | 0.692903 |
| NCI:79 | GMCSF-mediated signaling events | 36 | 1 | 0 | 0.502513 | 0 | 0.470449 | 0.692903 |
| NCI:86 | HIF-1-alpha transcription factor network | 65 | 1 | 0 | 0.502513 | 0 | 0.470449 | 0.692903 |
| NCI:90 | FOXA1 transcription factor network | 43 | 1 | 0 | 0.502513 | 0 | 0.470449 | 0.692903 |
| NCI:98 | IL2-mediated signaling events | 55 | 1 | 0 | 0.502513 | 0 | 0.470449 | 0.692903 |
| PF00005 | PFAM: ABC transporter | 49 | 1 | 0 | 0.502513 | 0 | 0.470449 | 0.692903 |
| PF00023 | PFAM: Ankyrin repeat | 272 | 1 | 0 | 0.502513 | 0 | 0.470449 | 0.692903 |
| PF00027 | PFAM: Cyclic nucleotide-binding domain | 36 | 1 | 0 | 0.502513 | 0 | 0.470449 | 0.692903 |
| PF00076 | PFAM: RNA recognition motif. (a.k.a. RRM, RBD, or RNP domain) | 221 | 1 | 0 | 0.502513 | 0 | 0.470449 | 0.692903 |
| PF00085 | PFAM: Thioredoxin | 31 | 1 | 0 | 0.502513 | 0 | 0.470449 | 0.692903 |
| PF00089 | PFAM: Trypsin | 131 | 1 | 0 | 0.502513 | 0 | 0.470449 | 0.692903 |
| PF00104 | PFAM: Ligand-binding domain of nuclear hormone receptor | 48 | 1 | 0 | 0.502513 | 0 | 0.470449 | 0.692903 |
| PF00105 | PFAM: Zinc finger, C4 type (two domains) | 46 | 1 | 0 | 0.502513 | 0 | 0.470449 | 0.692903 |
| PF00130 | PFAM: Phorbol esters/diacylglycerol binding domain (C1 domain) | 63 | 1 | 0 | 0.502513 | 0 | 0.470449 | 0.692903 |
| PF00147 | PFAM: Fibrinogen beta and gamma chains, C-terminal globular domain | 25 | 1 | 0 | 0.502513 | 0 | 0.470449 | 0.692903 |
| PF00160 | PFAM: Cyclophilin type peptidyl-prolyl cis-trans isomerase/CLD | 35 | 1 | 0 | 0.502513 | 0 | 0.470449 | 0.692903 |
| PF00167 | PFAM: Fibroblast growth factor | 26 | 1 | 0 | 0.502513 | 0 | 0.470449 | 0.692903 |
| PF00170 | PFAM: bZIP transcription factor | 34 | 1 | 0 | 0.502513 | 0 | 0.470449 | 0.692903 |
| PF00412 | PFAM: LIM domain | 72 | 1 | 0 | 0.502513 | 0 | 0.470449 | 0.692903 |
| PF00443 | PFAM: Ubiquitin carboxyl-terminal hydrolase | 83 | 1 | 0 | 0.502513 | 0 | 0.470449 | 0.692903 |
| PF00560 | PFAM: Leucine Rich Repeat | 217 | 1 | 0 | 0.502513 | 0 | 0.470449 | 0.692903 |
| PF00615 | PFAM: Regulator of G protein signaling domain | 35 | 1 | 0 | 0.502513 | 0 | 0.470449 | 0.692903 |
| PF00640 | PFAM: Phosphotyrosine interaction domain (PTB/PID) | 34 | 1 | 0 | 0.502513 | 0 | 0.470449 | 0.692903 |
| PF00651 | PFAM: BTB/POZ domain | 131 | 1 | 0 | 0.502513 | 0 | 0.470449 | 0.692903 |
| PF00788 | PFAM: Ras association (RalGDS/AF-6) domain | 39 | 1 | 0 | 0.502513 | 0 | 0.470449 | 0.692903 |
| PF01094 | PFAM: Receptor family ligand binding region | 38 | 1 | 0 | 0.502513 | 0 | 0.470449 | 0.692903 |
| PF01403 | PFAM: Sema domain | 30 | 1 | 0 | 0.502513 | 0 | 0.470449 | 0.692903 |
| PF01462 | PFAM: Leucine rich repeat N-terminal domain | 45 | 1 | 0 | 0.502513 | 0 | 0.470449 | 0.692903 |
| PF01833 | PFAM: IPT/TIG domain | 26 | 1 | 0 | 0.502513 | 0 | 0.470449 | 0.692903 |
| PF02373 | PFAM: JmjC domain | 25 | 1 | 0 | 0.502513 | 0 | 0.470449 | 0.692903 |
| PF02518 | PFAM: Histidine kinase-, DNA gyrase B-, and HSP90-like ATPase | 30 | 1 | 0 | 0.502513 | 0 | 0.470449 | 0.692903 |
| PF07714 | PFAM: Protein tyrosine kinase | 127 | 1 | 0 | 0.502513 | 0 | 0.470449 | 0.692903 |
| PF08266 | PFAM: Cadherin-like | 66 | 1 | 0 | 0.502513 | 0 | 0.470449 | 0.692903 |
| REACT:101 | REACT: Post NMDA receptor activation events | 33 | 1 | 0 | 0.502513 | 0 | 0.470449 | 0.692903 |
| REACT:1029 | REACT: Metabolism of amino acids and derivatives | 195 | 1 | 0 | 0.502513 | 0 | 0.470449 | 0.692903 |
| REACT:1037 | REACT: Signaling by FGFR | 45 | 1 | 0 | 0.502513 | 0 | 0.470449 | 0.692903 |
| REACT:1046 | REACT: L1CAM interactions | 94 | 1 | 0 | 0.502513 | 0 | 0.470449 | 0.692903 |
| REACT:1047 | REACT: Cap-dependent Translation Initiation | 112 | 1 | 0 | 0.502513 | 0 | 0.470449 | 0.692903 |
| REACT:1050 | REACT: FGFR2 ligand binding and activation | 27 | 1 | 0 | 0.502513 | 0 | 0.470449 | 0.692903 |
| REACT:1053 | REACT: Phase II conjugation | 63 | 1 | 0 | 0.502513 | 0 | 0.470449 | 0.692903 |
| REACT:1068 | REACT: FGFR1c ligand binding and activation | 27 | 1 | 0 | 0.502513 | 0 | 0.470449 | 0.692903 |
| REACT:1072 | REACT: Activation of the mRNA upon binding of the cap-binding complex and eIFs, and subsequent binding to 43S | 56 | 1 | 0 | 0.502513 | 0 | 0.470449 | 0.692903 |
| REACT:112 | REACT: Ligand-gated ion channel transport | 25 | 1 | 0 | 0.502513 | 0 | 0.470449 | 0.692903 |
| REACT:128 | REACT: Formation of a pool of free 40S subunits | 94 | 1 | 0 | 0.502513 | 0 | 0.470449 | 0.692903 |
| REACT:136 | REACT: Cell surface interactions at the vascular wall | 94 | 1 | 0 | 0.502513 | 0 | 0.470449 | 0.692903 |
| REACT:166 | REACT: FGFR1c and Klotho ligand binding and activation | 27 | 1 | 0 | 0.502513 | 0 | 0.470449 | 0.692903 |
| REACT:188 | REACT: Signaling by EGFR | 52 | 1 | 0 | 0.502513 | 0 | 0.470449 | 0.692903 |
| REACT:20 | REACT: Ribosomal scanning and start codon recognition | 55 | 1 | 0 | 0.502513 | 0 | 0.470449 | 0.692903 |
| REACT:204 | REACT: GTP hydrolysis and joining of the 60S ribosomal subunit | 105 | 1 | 0 | 0.502513 | 0 | 0.470449 | 0.692903 |
| REACT:206 | REACT: Signalling to ERKs | 35 | 1 | 0 | 0.502513 | 0 | 0.470449 | 0.692903 |
| REACT:212 | REACT: NCAM signaling for neurite out-growth | 69 | 1 | 0 | 0.502513 | 0 | 0.470449 | 0.692903 |
| REACT:234 | REACT: Circadian Clock | 29 | 1 | 0 | 0.502513 | 0 | 0.470449 | 0.692903 |
| REACT:296 | REACT: FRS2-mediated cascade | 27 | 1 | 0 | 0.502513 | 0 | 0.470449 | 0.692903 |
| REACT:305 | REACT: Interleukin receptor SHC signaling | 29 | 1 | 0 | 0.502513 | 0 | 0.470449 | 0.692903 |
| REACT:344 | REACT: Formation of the ternary complex, and subsequently, the 43S complex | 48 | 1 | 0 | 0.502513 | 0 | 0.470449 | 0.692903 |
| REACT:381 | REACT: Signalling to RAS | 27 | 1 | 0 | 0.502513 | 0 | 0.470449 | 0.692903 |
| REACT:382 | REACT: FGFR ligand binding and activation | 27 | 1 | 0 | 0.502513 | 0 | 0.470449 | 0.692903 |
| REACT:398 | REACT: CREB phosphorylation through the activation of Ras | 27 | 1 | 0 | 0.502513 | 0 | 0.470449 | 0.692903 |
| REACT:409 | REACT: Translation initiation complex formation | 55 | 1 | 0 | 0.502513 | 0 | 0.470449 | 0.692903 |
| REACT:434 | REACT: Hormone biosynthesis | 59 | 1 | 0 | 0.502513 | 0 | 0.470449 | 0.692903 |
| REACT:44 | REACT: Insulin Synthesis and Processing | 133 | 1 | 0 | 0.502513 | 0 | 0.470449 | 0.692903 |
| REACT:463 | REACT: Netrin-1 signaling | 42 | 1 | 0 | 0.502513 | 0 | 0.470449 | 0.692903 |
| REACT:466 | REACT: Semaphorin interactions | 66 | 1 | 0 | 0.502513 | 0 | 0.470449 | 0.692903 |
| REACT:47 | REACT: GABA receptor activation | 53 | 1 | 0 | 0.502513 | 0 | 0.470449 | 0.692903 |
| REACT:470 | REACT: Signaling by Interleukins | 89 | 1 | 0 | 0.502513 | 0 | 0.470449 | 0.692903 |
| REACT:526 | REACT: Eukaryotic Translation Initiation | 112 | 1 | 0 | 0.502513 | 0 | 0.470449 | 0.692903 |
| REACT:53 | REACT: FGFR3b ligand binding and activation | 27 | 1 | 0 | 0.502513 | 0 | 0.470449 | 0.692903 |
| REACT:54 | REACT: Interleukin-3, 5 and GM-CSF signaling | 45 | 1 | 0 | 0.502513 | 0 | 0.470449 | 0.692903 |
| REACT:548 | REACT: Regulation of beta-cell development | 114 | 1 | 0 | 0.502513 | 0 | 0.470449 | 0.692903 |
| REACT:549 | REACT: FGFR2c ligand binding and activation | 27 | 1 | 0 | 0.502513 | 0 | 0.470449 | 0.692903 |
| REACT:589 | REACT: FGFR3 ligand binding and activation | 27 | 1 | 0 | 0.502513 | 0 | 0.470449 | 0.692903 |
| REACT:59 | REACT: NGF signalling via TRKA from the plasma membrane | 136 | 1 | 0 | 0.502513 | 0 | 0.470449 | 0.692903 |
| REACT:6 | REACT: Ion channel transport | 61 | 1 | 0 | 0.502513 | 0 | 0.470449 | 0.692903 |
| REACT:607 | REACT: Transcription | 177 | 1 | 0 | 0.502513 | 0 | 0.470449 | 0.692903 |
| REACT:614 | REACT: Iron uptake and transport | 37 | 1 | 0 | 0.502513 | 0 | 0.470449 | 0.692903 |
| REACT:630 | REACT: RNA Polymerase III Abortive And Retractive Initiation | 34 | 1 | 0 | 0.502513 | 0 | 0.470449 | 0.692903 |
| REACT:636 | REACT: PI-3K cascade | 38 | 1 | 0 | 0.502513 | 0 | 0.470449 | 0.692903 |
| REACT:646 | REACT: RNA Polymerase III Transcription Initiation | 29 | 1 | 0 | 0.502513 | 0 | 0.470449 | 0.692903 |
| REACT:66 | REACT: RNA Polymerase I, RNA Polymerase III, and Mitochondrial Transcription | 91 | 1 | 0 | 0.502513 | 0 | 0.470449 | 0.692903 |
| REACT:671 | REACT: Pyruvate metabolism and Citric Acid (TCA) cycle | 40 | 1 | 0 | 0.502513 | 0 | 0.470449 | 0.692903 |
| REACT:679 | REACT: Nuclear Receptor transcription pathway | 51 | 1 | 0 | 0.502513 | 0 | 0.470449 | 0.692903 |
| REACT:69 | REACT: FGFR4 ligand binding and activation | 27 | 1 | 0 | 0.502513 | 0 | 0.470449 | 0.692903 |
| REACT:70 | REACT: PI3K Cascade | 69 | 1 | 0 | 0.502513 | 0 | 0.470449 | 0.692903 |
| REACT:701 | REACT: FGFR1b ligand binding and activation | 27 | 1 | 0 | 0.502513 | 0 | 0.470449 | 0.692903 |
| REACT:703 | REACT: Transferrin endocytosis and recycling | 27 | 1 | 0 | 0.502513 | 0 | 0.470449 | 0.692903 |
| REACT:705 | REACT: Diabetes pathways | 312 | 1 | 0 | 0.502513 | 0 | 0.470449 | 0.692903 |
| REACT:738 | REACT: Activation of NMDA receptor upon glutamate binding and postsynaptic events | 37 | 1 | 0 | 0.502513 | 0 | 0.470449 | 0.692903 |
| REACT:741 | REACT: 3' -UTR-mediated translational regulation | 104 | 1 | 0 | 0.502513 | 0 | 0.470449 | 0.692903 |
| REACT:743 | REACT: FGFR1 ligand binding and activation | 27 | 1 | 0 | 0.502513 | 0 | 0.470449 | 0.692903 |
| REACT:785 | REACT: FGFR3c ligand binding and activation | 27 | 1 | 0 | 0.502513 | 0 | 0.470449 | 0.692903 |
| REACT:801 | REACT: Activation of Kainate Receptors upon glutamate binding | 30 | 1 | 0 | 0.502513 | 0 | 0.470449 | 0.692903 |
| REACT:814 | REACT: Translation | 119 | 1 | 0 | 0.502513 | 0 | 0.470449 | 0.692903 |
| REACT:824 | REACT: L13a-mediated translational silencing of Ceruloplasmin expression | 104 | 1 | 0 | 0.502513 | 0 | 0.470449 | 0.692903 |
| REACT:896 | REACT: FGFR2b ligand binding and activation | 27 | 1 | 0 | 0.502513 | 0 | 0.470449 | 0.692903 |
| REACT:909 | REACT: Downstream signaling of activated FGFR | 43 | 1 | 0 | 0.502513 | 0 | 0.470449 | 0.692903 |
| REACT:993 | REACT: RNA Polymerase III Transcription | 34 | 1 | 0 | 0.502513 | 0 | 0.470449 | 0.692903 |
| GO:0072521 | purine-containing compound metabolic process | 676 | 6 | 6 | 3.015075 | 2.678571 | 0.531512 | 0.693102 |
| GO:0034655 | nucleobase, nucleoside, nucleotide and nucleic acid catabolic process | 435 | 5 | 5 | 2.512563 | 2.232143 | 0.54925 | 0.692248 |
| GO:0034656 | nucleobase, nucleoside and nucleotide catabolic process | 435 | 5 | 5 | 2.512563 | 2.232143 | 0.54925 | 0.692248 |
| GO:0042802 | identical protein binding | 727 | 5 | 5 | 2.512563 | 2.232143 | 0.54925 | 0.692248 |
| GO:0044270 | cellular nitrogen compound catabolic process | 461 | 5 | 5 | 2.512563 | 2.232143 | 0.54925 | 0.692248 |
| GO:0072523 | purine-containing compound catabolic process | 406 | 5 | 5 | 2.512563 | 2.232143 | 0.54925 | 0.692248 |
| GO:0005085 | guanyl-nucleotide exchange factor activity | 160 | 4 | 4 | 2.01005 | 1.785714 | 0.571206 | 0.69548 |
| GO:0006195 | purine nucleotide catabolic process | 401 | 4 | 4 | 2.01005 | 1.785714 | 0.571206 | 0.69548 |
| GO:0009150 | purine ribonucleotide metabolic process | 456 | 4 | 4 | 2.01005 | 1.785714 | 0.571206 | 0.69548 |
| GO:0009166 | nucleotide catabolic process | 418 | 4 | 4 | 2.01005 | 1.785714 | 0.571206 | 0.69548 |
| GO:0009308 | amine metabolic process | 542 | 4 | 4 | 2.01005 | 1.785714 | 0.571206 | 0.69548 |
| GO:0016874 | ligase activity | 455 | 4 | 4 | 2.01005 | 1.785714 | 0.571206 | 0.69548 |
| GO:0044429 | mitochondrial part | 676 | 4 | 4 | 2.01005 | 1.785714 | 0.571206 | 0.69548 |
| GO:0005525 | GTP binding | 369 | 3 | 3 | 1.507538 | 1.339286 | 0.600168 | 0.697878 |
| GO:0005770 | late endosome | 122 | 3 | 3 | 1.507538 | 1.339286 | 0.600168 | 0.697878 |
| GO:0006259 | DNA metabolic process | 632 | 3 | 3 | 1.507538 | 1.339286 | 0.600168 | 0.697878 |
| GO:0006412 | translation | 422 | 3 | 3 | 1.507538 | 1.339286 | 0.600168 | 0.697878 |
| GO:0006520 | cellular amino acid metabolic process | 384 | 3 | 3 | 1.507538 | 1.339286 | 0.600168 | 0.697878 |
| GO:0007010 | cytoskeleton organization | 557 | 3 | 3 | 1.507538 | 1.339286 | 0.600168 | 0.697878 |
| GO:0009894 | regulation of catabolic process | 324 | 3 | 3 | 1.507538 | 1.339286 | 0.600168 | 0.697878 |
| GO:0016044 | cellular membrane organization | 459 | 3 | 3 | 1.507538 | 1.339286 | 0.600168 | 0.697878 |
| GO:0019001 | guanyl nucleotide binding | 380 | 3 | 3 | 1.507538 | 1.339286 | 0.600168 | 0.697878 |
| GO:0032561 | guanyl ribonucleotide binding | 380 | 3 | 3 | 1.507538 | 1.339286 | 0.600168 | 0.697878 |
| GO:0044106 | cellular amine metabolic process | 461 | 3 | 3 | 1.507538 | 1.339286 | 0.600168 | 0.697878 |
| GO:0045211 | postsynaptic membrane | 153 | 3 | 3 | 1.507538 | 1.339286 | 0.600168 | 0.697878 |
| GO:0050776 | regulation of immune response | 449 | 3 | 3 | 1.507538 | 1.339286 | 0.600168 | 0.697878 |
| GO:0051336 | regulation of hydrolase activity | 481 | 3 | 3 | 1.507538 | 1.339286 | 0.600168 | 0.697878 |
| GO:0061024 | membrane organization | 461 | 3 | 3 | 1.507538 | 1.339286 | 0.600168 | 0.697878 |
| GO:2000026 | regulation of multicellular organismal development | 696 | 3 | 3 | 1.507538 | 1.339286 | 0.600168 | 0.697878 |
| KEGG:04740 | KEGG: Olfactory transduction | 388 | 3 | 3 | 1.507538 | 1.339286 | 0.600168 | 0.697878 |
| GO:0007264 | small GTPase mediated signal transduction | 579 | 7 | 8 | 3.517588 | 3.571429 | 0.613001 | 0.697386 |
| GO:0002757 | immune response-activating signal transduction | 192 | 2 | 2 | 1.005025 | 0.892857 | 0.642692 | 0.708638 |
| GO:0002764 | immune response-regulating signaling pathway | 196 | 2 | 2 | 1.005025 | 0.892857 | 0.642692 | 0.708638 |
| GO:0004812 | aminoacyl-tRNA ligase activity | 49 | 2 | 2 | 1.005025 | 0.892857 | 0.642692 | 0.708638 |
| GO:0005198 | structural molecule activity | 606 | 2 | 2 | 1.005025 | 0.892857 | 0.642692 | 0.708638 |
| GO:0005543 | phospholipid binding | 194 | 2 | 2 | 1.005025 | 0.892857 | 0.642692 | 0.708638 |
| GO:0005740 | mitochondrial envelope | 469 | 2 | 2 | 1.005025 | 0.892857 | 0.642692 | 0.708638 |
| GO:0005765 | lysosomal membrane | 117 | 2 | 2 | 1.005025 | 0.892857 | 0.642692 | 0.708638 |
| GO:0005774 | vacuolar membrane | 151 | 2 | 2 | 1.005025 | 0.892857 | 0.642692 | 0.708638 |
| GO:0006418 | tRNA aminoacylation for protein translation | 48 | 2 | 2 | 1.005025 | 0.892857 | 0.642692 | 0.708638 |
| GO:0006954 | inflammatory response | 361 | 2 | 2 | 1.005025 | 0.892857 | 0.642692 | 0.708638 |
| GO:0007283 | spermatogenesis | 329 | 2 | 2 | 1.005025 | 0.892857 | 0.642692 | 0.708638 |
| GO:0007548 | sex differentiation | 200 | 2 | 2 | 1.005025 | 0.892857 | 0.642692 | 0.708638 |
| GO:0007610 | behavior | 354 | 2 | 2 | 1.005025 | 0.892857 | 0.642692 | 0.708638 |
| GO:0008202 | steroid metabolic process | 257 | 2 | 2 | 1.005025 | 0.892857 | 0.642692 | 0.708638 |
| GO:0008289 | lipid binding | 434 | 2 | 2 | 1.005025 | 0.892857 | 0.642692 | 0.708638 |
| GO:0008610 | lipid biosynthetic process | 421 | 2 | 2 | 1.005025 | 0.892857 | 0.642692 | 0.708638 |
| GO:0009057 | macromolecule catabolic process | 633 | 2 | 2 | 1.005025 | 0.892857 | 0.642692 | 0.708638 |
| GO:0009123 | nucleoside monophosphate metabolic process | 193 | 2 | 2 | 1.005025 | 0.892857 | 0.642692 | 0.708638 |
| GO:0009967 | positive regulation of signal transduction | 467 | 2 | 2 | 1.005025 | 0.892857 | 0.642692 | 0.708638 |
| GO:0010008 | endosome membrane | 250 | 2 | 2 | 1.005025 | 0.892857 | 0.642692 | 0.708638 |
| GO:0010647 | positive regulation of cell communication | 490 | 2 | 2 | 1.005025 | 0.892857 | 0.642692 | 0.708638 |
| GO:0016337 | cell-cell adhesion | 316 | 2 | 2 | 1.005025 | 0.892857 | 0.642692 | 0.708638 |
| GO:0016701 | oxidoreductase activity, acting on single donors with incorporation of molecular oxygen | 81 | 2 | 2 | 1.005025 | 0.892857 | 0.642692 | 0.708638 |
| GO:0016702 | oxidoreductase activity, acting on single donors with incorporation of molecular oxygen, incorporation of two atoms of oxygen | 80 | 2 | 2 | 1.005025 | 0.892857 | 0.642692 | 0.708638 |
| GO:0016875 | ligase activity, forming carbon-oxygen bonds | 49 | 2 | 2 | 1.005025 | 0.892857 | 0.642692 | 0.708638 |
| GO:0016876 | ligase activity, forming aminoacyl-tRNA and related compounds | 49 | 2 | 2 | 1.005025 | 0.892857 | 0.642692 | 0.708638 |
| GO:0023056 | positive regulation of signaling | 493 | 2 | 2 | 1.005025 | 0.892857 | 0.642692 | 0.708638 |
| GO:0030529 | ribonucleoprotein complex | 515 | 2 | 2 | 1.005025 | 0.892857 | 0.642692 | 0.708638 |
| GO:0031966 | mitochondrial membrane | 446 | 2 | 2 | 1.005025 | 0.892857 | 0.642692 | 0.708638 |
| GO:0035091 | phosphatidylinositol binding | 116 | 2 | 2 | 1.005025 | 0.892857 | 0.642692 | 0.708638 |
| GO:0040007 | growth | 576 | 2 | 2 | 1.005025 | 0.892857 | 0.642692 | 0.708638 |
| GO:0043038 | amino acid activation | 48 | 2 | 2 | 1.005025 | 0.892857 | 0.642692 | 0.708638 |
| GO:0043039 | tRNA aminoacylation | 48 | 2 | 2 | 1.005025 | 0.892857 | 0.642692 | 0.708638 |
| GO:0044437 | vacuolar part | 160 | 2 | 2 | 1.005025 | 0.892857 | 0.642692 | 0.708638 |
| GO:0044440 | endosomal part | 252 | 2 | 2 | 1.005025 | 0.892857 | 0.642692 | 0.708638 |
| GO:0045137 | development of primary sexual characteristics | 172 | 2 | 2 | 1.005025 | 0.892857 | 0.642692 | 0.708638 |
| GO:0045892 | negative regulation of transcription, DNA-dependent | 494 | 2 | 2 | 1.005025 | 0.892857 | 0.642692 | 0.708638 |
| GO:0046545 | development of primary female sexual characteristics | 86 | 2 | 2 | 1.005025 | 0.892857 | 0.642692 | 0.708638 |
| GO:0046660 | female sex differentiation | 90 | 2 | 2 | 1.005025 | 0.892857 | 0.642692 | 0.708638 |
| GO:0048232 | male gamete generation | 329 | 2 | 2 | 1.005025 | 0.892857 | 0.642692 | 0.708638 |
| GO:0048608 | reproductive structure development | 194 | 2 | 2 | 1.005025 | 0.892857 | 0.642692 | 0.708638 |
| GO:0051128 | regulation of cellular component organization | 737 | 2 | 2 | 1.005025 | 0.892857 | 0.642692 | 0.708638 |
| GO:0051213 | dioxygenase activity | 80 | 2 | 2 | 1.005025 | 0.892857 | 0.642692 | 0.708638 |
| GO:0080134 | regulation of response to stress | 480 | 2 | 2 | 1.005025 | 0.892857 | 0.642692 | 0.708638 |
| KEGG:00970 | KEGG: Aminoacyl-tRNA biosynthesis | 41 | 2 | 2 | 1.005025 | 0.892857 | 0.642692 | 0.708638 |
| REACT:625 | REACT: Cell Cycle, Mitotic | 315 | 2 | 2 | 1.005025 | 0.892857 | 0.642692 | 0.708638 |
| GO:0002682 | regulation of immune system process | 673 | 4 | 5 | 2.01005 | 2.232143 | 0.68663 | 0.711676 |
| GO:0002684 | positive regulation of immune system process | 438 | 4 | 5 | 2.01005 | 2.232143 | 0.68663 | 0.711676 |
| GO:0009259 | ribonucleotide metabolic process | 467 | 4 | 5 | 2.01005 | 2.232143 | 0.68663 | 0.711676 |
| GO:0001523 | retinoid metabolic process | 35 | 1 | 1 | 0.502513 | 0.446429 | 0.720166 | 0.759933 |
| GO:0001890 | placenta development | 84 | 1 | 1 | 0.502513 | 0.446429 | 0.720166 | 0.759933 |
| GO:0001948 | glycoprotein binding | 47 | 1 | 1 | 0.502513 | 0.446429 | 0.720166 | 0.759933 |
| GO:0002252 | immune effector process | 290 | 1 | 1 | 0.502513 | 0.446429 | 0.720166 | 0.759933 |
| GO:0002576 | platelet degranulation | 80 | 1 | 1 | 0.502513 | 0.446429 | 0.720166 | 0.759933 |
| GO:0003779 | actin binding | 324 | 1 | 1 | 0.502513 | 0.446429 | 0.720166 | 0.759933 |
| GO:0004112 | cyclic-nucleotide phosphodiesterase activity | 25 | 1 | 1 | 0.502513 | 0.446429 | 0.720166 | 0.759933 |
| GO:0004252 | serine-type endopeptidase activity | 154 | 1 | 1 | 0.502513 | 0.446429 | 0.720166 | 0.759933 |
| GO:0004713 | protein tyrosine kinase activity | 140 | 1 | 1 | 0.502513 | 0.446429 | 0.720166 | 0.759933 |
| GO:0004842 | ubiquitin-protein ligase activity | 208 | 1 | 1 | 0.502513 | 0.446429 | 0.720166 | 0.759933 |
| GO:0004857 | enzyme inhibitor activity | 285 | 1 | 1 | 0.502513 | 0.446429 | 0.720166 | 0.759933 |
| GO:0004866 | endopeptidase inhibitor activity | 151 | 1 | 1 | 0.502513 | 0.446429 | 0.720166 | 0.759933 |
| GO:0004867 | serine-type endopeptidase inhibitor activity | 92 | 1 | 1 | 0.502513 | 0.446429 | 0.720166 | 0.759933 |
| GO:0005231 | excitatory extracellular ligand-gated ion channel activity | 48 | 1 | 1 | 0.502513 | 0.446429 | 0.720166 | 0.759933 |
| GO:0005253 | anion channel activity | 82 | 1 | 1 | 0.502513 | 0.446429 | 0.720166 | 0.759933 |
| GO:0005254 | chloride channel activity | 73 | 1 | 1 | 0.502513 | 0.446429 | 0.720166 | 0.759933 |
| GO:0005795 | Golgi stack | 107 | 1 | 1 | 0.502513 | 0.446429 | 0.720166 | 0.759933 |
| GO:0005929 | cilium | 170 | 1 | 1 | 0.502513 | 0.446429 | 0.720166 | 0.759933 |
| GO:0006066 | alcohol metabolic process | 504 | 1 | 1 | 0.502513 | 0.446429 | 0.720166 | 0.759933 |
| GO:0006081 | cellular aldehyde metabolic process | 32 | 1 | 1 | 0.502513 | 0.446429 | 0.720166 | 0.759933 |
| GO:0006164 | purine nucleotide biosynthetic process | 245 | 1 | 1 | 0.502513 | 0.446429 | 0.720166 | 0.759933 |
| GO:0006457 | protein folding | 209 | 1 | 1 | 0.502513 | 0.446429 | 0.720166 | 0.759933 |
| GO:0006732 | coenzyme metabolic process | 182 | 1 | 1 | 0.502513 | 0.446429 | 0.720166 | 0.759933 |
| GO:0006775 | fat-soluble vitamin metabolic process | 49 | 1 | 1 | 0.502513 | 0.446429 | 0.720166 | 0.759933 |
| GO:0006776 | vitamin A metabolic process | 30 | 1 | 1 | 0.502513 | 0.446429 | 0.720166 | 0.759933 |
| GO:0007088 | regulation of mitosis | 82 | 1 | 1 | 0.502513 | 0.446429 | 0.720166 | 0.759933 |
| GO:0007091 | mitotic metaphase/anaphase transition | 50 | 1 | 1 | 0.502513 | 0.446429 | 0.720166 | 0.759933 |
| GO:0007093 | mitotic cell cycle checkpoint | 126 | 1 | 1 | 0.502513 | 0.446429 | 0.720166 | 0.759933 |
| GO:0007094 | mitotic cell cycle spindle assembly checkpoint | 31 | 1 | 1 | 0.502513 | 0.446429 | 0.720166 | 0.759933 |
| GO:0007249 | I-kappaB kinase/NF-kappaB cascade | 178 | 1 | 1 | 0.502513 | 0.446429 | 0.720166 | 0.759933 |
| GO:0007368 | determination of left/right symmetry | 61 | 1 | 1 | 0.502513 | 0.446429 | 0.720166 | 0.759933 |
| GO:0008081 | phosphoric diester hydrolase activity | 89 | 1 | 1 | 0.502513 | 0.446429 | 0.720166 | 0.759933 |
| GO:0008361 | regulation of cell size | 312 | 1 | 1 | 0.502513 | 0.446429 | 0.720166 | 0.759933 |
| GO:0009124 | nucleoside monophosphate biosynthetic process | 168 | 1 | 1 | 0.502513 | 0.446429 | 0.720166 | 0.759933 |
| GO:0009156 | ribonucleoside monophosphate biosynthetic process | 26 | 1 | 1 | 0.502513 | 0.446429 | 0.720166 | 0.759933 |
| GO:0009161 | ribonucleoside monophosphate metabolic process | 30 | 1 | 1 | 0.502513 | 0.446429 | 0.720166 | 0.759933 |
| GO:0009164 | nucleoside catabolic process | 25 | 1 | 1 | 0.502513 | 0.446429 | 0.720166 | 0.759933 |
| GO:0009165 | nucleotide biosynthetic process | 285 | 1 | 1 | 0.502513 | 0.446429 | 0.720166 | 0.759933 |
| GO:0009187 | cyclic nucleotide metabolic process | 157 | 1 | 1 | 0.502513 | 0.446429 | 0.720166 | 0.759933 |
| GO:0009260 | ribonucleotide biosynthetic process | 109 | 1 | 1 | 0.502513 | 0.446429 | 0.720166 | 0.759933 |
| GO:0009306 | protein secretion | 99 | 1 | 1 | 0.502513 | 0.446429 | 0.720166 | 0.759933 |
| GO:0009617 | response to bacterium | 254 | 1 | 1 | 0.502513 | 0.446429 | 0.720166 | 0.759933 |
| GO:0009792 | embryo development ending in birth or egg hatching | 357 | 1 | 1 | 0.502513 | 0.446429 | 0.720166 | 0.759933 |
| GO:0009799 | specification of symmetry | 66 | 1 | 1 | 0.502513 | 0.446429 | 0.720166 | 0.759933 |
| GO:0009855 | determination of bilateral symmetry | 65 | 1 | 1 | 0.502513 | 0.446429 | 0.720166 | 0.759933 |
| GO:0009887 | organ morphogenesis | 585 | 1 | 1 | 0.502513 | 0.446429 | 0.720166 | 0.759933 |
| GO:0010639 | negative regulation of organelle organization | 124 | 1 | 1 | 0.502513 | 0.446429 | 0.720166 | 0.759933 |
| GO:0010740 | positive regulation of intracellular protein kinase cascade | 256 | 1 | 1 | 0.502513 | 0.446429 | 0.720166 | 0.759933 |
| GO:0014069 | postsynaptic density | 83 | 1 | 1 | 0.502513 | 0.446429 | 0.720166 | 0.759933 |
| GO:0015035 | protein disulfide oxidoreductase activity | 28 | 1 | 1 | 0.502513 | 0.446429 | 0.720166 | 0.759933 |
| GO:0015036 | disulfide oxidoreductase activity | 31 | 1 | 1 | 0.502513 | 0.446429 | 0.720166 | 0.759933 |
| GO:0016049 | cell growth | 297 | 1 | 1 | 0.502513 | 0.446429 | 0.720166 | 0.759933 |
| GO:0016101 | diterpenoid metabolic process | 35 | 1 | 1 | 0.502513 | 0.446429 | 0.720166 | 0.759933 |
| GO:0016567 | protein ubiquitination | 338 | 1 | 1 | 0.502513 | 0.446429 | 0.720166 | 0.759933 |
| GO:0016705 | oxidoreductase activity, acting on paired donors, with incorporation or reduction of molecular oxygen | 140 | 1 | 1 | 0.502513 | 0.446429 | 0.720166 | 0.759933 |
| GO:0016814 | hydrolase activity, acting on carbon-nitrogen (but not peptide) bonds, in cyclic amidines | 33 | 1 | 1 | 0.502513 | 0.446429 | 0.720166 | 0.759933 |
| GO:0016879 | ligase activity, forming carbon-nitrogen bonds | 294 | 1 | 1 | 0.502513 | 0.446429 | 0.720166 | 0.759933 |
| GO:0016881 | acid-amino acid ligase activity | 263 | 1 | 1 | 0.502513 | 0.446429 | 0.720166 | 0.759933 |
| GO:0017038 | protein import | 196 | 1 | 1 | 0.502513 | 0.446429 | 0.720166 | 0.759933 |
| GO:0018130 | heterocycle biosynthetic process | 351 | 1 | 1 | 0.502513 | 0.446429 | 0.720166 | 0.759933 |
| GO:0019221 | cytokine-mediated signaling pathway | 209 | 1 | 1 | 0.502513 | 0.446429 | 0.720166 | 0.759933 |
| GO:0019239 | deaminase activity | 25 | 1 | 1 | 0.502513 | 0.446429 | 0.720166 | 0.759933 |
| GO:0019722 | calcium-mediated signaling | 60 | 1 | 1 | 0.502513 | 0.446429 | 0.720166 | 0.759933 |
| GO:0019787 | small conjugating protein ligase activity | 221 | 1 | 1 | 0.502513 | 0.446429 | 0.720166 | 0.759933 |
| GO:0019867 | outer membrane | 124 | 1 | 1 | 0.502513 | 0.446429 | 0.720166 | 0.759933 |
| GO:0019932 | second-messenger-mediated signaling | 304 | 1 | 1 | 0.502513 | 0.446429 | 0.720166 | 0.759933 |
| GO:0019955 | cytokine binding | 114 | 1 | 1 | 0.502513 | 0.446429 | 0.720166 | 0.759933 |
| GO:0030071 | regulation of mitotic metaphase/anaphase transition | 41 | 1 | 1 | 0.502513 | 0.446429 | 0.720166 | 0.759933 |
| GO:0030141 | stored secretory granule | 209 | 1 | 1 | 0.502513 | 0.446429 | 0.720166 | 0.759933 |
| GO:0030173 | integral to Golgi membrane | 46 | 1 | 1 | 0.502513 | 0.446429 | 0.720166 | 0.759933 |
| GO:0030414 | peptidase inhibitor activity | 159 | 1 | 1 | 0.502513 | 0.446429 | 0.720166 | 0.759933 |
| GO:0031228 | intrinsic to Golgi membrane | 49 | 1 | 1 | 0.502513 | 0.446429 | 0.720166 | 0.759933 |
| GO:0031300 | intrinsic to organelle membrane | 160 | 1 | 1 | 0.502513 | 0.446429 | 0.720166 | 0.759933 |
| GO:0031301 | integral to organelle membrane | 136 | 1 | 1 | 0.502513 | 0.446429 | 0.720166 | 0.759933 |
| GO:0031577 | spindle checkpoint | 36 | 1 | 1 | 0.502513 | 0.446429 | 0.720166 | 0.759933 |
| GO:0031968 | organelle outer membrane | 120 | 1 | 1 | 0.502513 | 0.446429 | 0.720166 | 0.759933 |
| GO:0031984 | organelle subcompartment | 83 | 1 | 1 | 0.502513 | 0.446429 | 0.720166 | 0.759933 |
| GO:0031985 | Golgi cisterna | 81 | 1 | 1 | 0.502513 | 0.446429 | 0.720166 | 0.759933 |
| GO:0032103 | positive regulation of response to external stimulus | 90 | 1 | 1 | 0.502513 | 0.446429 | 0.720166 | 0.759933 |
| GO:0032403 | protein complex binding | 260 | 1 | 1 | 0.502513 | 0.446429 | 0.720166 | 0.759933 |
| GO:0032446 | protein modification by small protein conjugation | 360 | 1 | 1 | 0.502513 | 0.446429 | 0.720166 | 0.759933 |
| GO:0032535 | regulation of cellular component size | 379 | 1 | 1 | 0.502513 | 0.446429 | 0.720166 | 0.759933 |
| GO:0032944 | regulation of mononuclear cell proliferation | 105 | 1 | 1 | 0.502513 | 0.446429 | 0.720166 | 0.759933 |
| GO:0032946 | positive regulation of mononuclear cell proliferation | 76 | 1 | 1 | 0.502513 | 0.446429 | 0.720166 | 0.759933 |
| GO:0033043 | regulation of organelle organization | 295 | 1 | 1 | 0.502513 | 0.446429 | 0.720166 | 0.759933 |
| GO:0034097 | response to cytokine stimulus | 310 | 1 | 1 | 0.502513 | 0.446429 | 0.720166 | 0.759933 |
| GO:0034404 | nucleobase, nucleoside and nucleotide biosynthetic process | 306 | 1 | 1 | 0.502513 | 0.446429 | 0.720166 | 0.759933 |
| GO:0034612 | response to tumor necrosis factor | 50 | 1 | 1 | 0.502513 | 0.446429 | 0.720166 | 0.759933 |
| GO:0034654 | nucleobase, nucleoside, nucleotide and nucleic acid biosynthetic process | 306 | 1 | 1 | 0.502513 | 0.446429 | 0.720166 | 0.759933 |
| GO:0034707 | chloride channel complex | 55 | 1 | 1 | 0.502513 | 0.446429 | 0.720166 | 0.759933 |
| GO:0034754 | cellular hormone metabolic process | 83 | 1 | 1 | 0.502513 | 0.446429 | 0.720166 | 0.759933 |
| GO:0035270 | endocrine system development | 177 | 1 | 1 | 0.502513 | 0.446429 | 0.720166 | 0.759933 |
| GO:0043009 | chordate embryonic development | 353 | 1 | 1 | 0.502513 | 0.446429 | 0.720166 | 0.759933 |
| GO:0043086 | negative regulation of catalytic activity | 355 | 1 | 1 | 0.502513 | 0.446429 | 0.720166 | 0.759933 |
| GO:0043193 | positive regulation of gene-specific transcription | 193 | 1 | 1 | 0.502513 | 0.446429 | 0.720166 | 0.759933 |
| GO:0044257 | cellular protein catabolic process | 339 | 1 | 1 | 0.502513 | 0.446429 | 0.720166 | 0.759933 |
| GO:0044262 | cellular carbohydrate metabolic process | 472 | 1 | 1 | 0.502513 | 0.446429 | 0.720166 | 0.759933 |
| GO:0044265 | cellular macromolecule catabolic process | 510 | 1 | 1 | 0.502513 | 0.446429 | 0.720166 | 0.759933 |
| GO:0044271 | cellular nitrogen compound biosynthetic process | 504 | 1 | 1 | 0.502513 | 0.446429 | 0.720166 | 0.759933 |
| GO:0045596 | negative regulation of cell differentiation | 253 | 1 | 1 | 0.502513 | 0.446429 | 0.720166 | 0.759933 |
| GO:0045839 | negative regulation of mitosis | 36 | 1 | 1 | 0.502513 | 0.446429 | 0.720166 | 0.759933 |
| GO:0045841 | negative regulation of mitotic metaphase/anaphase transition | 32 | 1 | 1 | 0.502513 | 0.446429 | 0.720166 | 0.759933 |
| GO:0047485 | protein N-terminus binding | 76 | 1 | 1 | 0.502513 | 0.446429 | 0.720166 | 0.759933 |
| GO:0048520 | positive regulation of behavior | 47 | 1 | 1 | 0.502513 | 0.446429 | 0.720166 | 0.759933 |
| GO:0048562 | embryonic organ morphogenesis | 155 | 1 | 1 | 0.502513 | 0.446429 | 0.720166 | 0.759933 |
| GO:0048568 | embryonic organ development | 222 | 1 | 1 | 0.502513 | 0.446429 | 0.720166 | 0.759933 |
| GO:0048585 | negative regulation of response to stimulus | 465 | 1 | 1 | 0.502513 | 0.446429 | 0.720166 | 0.759933 |
| GO:0048589 | developmental growth | 191 | 1 | 1 | 0.502513 | 0.446429 | 0.720166 | 0.759933 |
| GO:0048598 | embryonic morphogenesis | 364 | 1 | 1 | 0.502513 | 0.446429 | 0.720166 | 0.759933 |
| GO:0048732 | gland development | 205 | 1 | 1 | 0.502513 | 0.446429 | 0.720166 | 0.759933 |
| GO:0050670 | regulation of lymphocyte proliferation | 104 | 1 | 1 | 0.502513 | 0.446429 | 0.720166 | 0.759933 |
| GO:0050671 | positive regulation of lymphocyte proliferation | 75 | 1 | 1 | 0.502513 | 0.446429 | 0.720166 | 0.759933 |
| GO:0050900 | leukocyte migration | 183 | 1 | 1 | 0.502513 | 0.446429 | 0.720166 | 0.759933 |
| GO:0050921 | positive regulation of chemotaxis | 40 | 1 | 1 | 0.502513 | 0.446429 | 0.720166 | 0.759933 |
| GO:0051020 | GTPase binding | 118 | 1 | 1 | 0.502513 | 0.446429 | 0.720166 | 0.759933 |
| GO:0051052 | regulation of DNA metabolic process | 137 | 1 | 1 | 0.502513 | 0.446429 | 0.720166 | 0.759933 |
| GO:0051093 | negative regulation of developmental process | 305 | 1 | 1 | 0.502513 | 0.446429 | 0.720166 | 0.759933 |
| GO:0051129 | negative regulation of cellular component organization | 219 | 1 | 1 | 0.502513 | 0.446429 | 0.720166 | 0.759933 |
| GO:0051186 | cofactor metabolic process | 230 | 1 | 1 | 0.502513 | 0.446429 | 0.720166 | 0.759933 |
| GO:0051241 | negative regulation of multicellular organismal process | 234 | 1 | 1 | 0.502513 | 0.446429 | 0.720166 | 0.759933 |
| GO:0051325 | interphase | 338 | 1 | 1 | 0.502513 | 0.446429 | 0.720166 | 0.759933 |
| GO:0051329 | interphase of mitotic cell cycle | 331 | 1 | 1 | 0.502513 | 0.446429 | 0.720166 | 0.759933 |
| GO:0051640 | organelle localization | 118 | 1 | 1 | 0.502513 | 0.446429 | 0.720166 | 0.759933 |
| GO:0051783 | regulation of nuclear division | 82 | 1 | 1 | 0.502513 | 0.446429 | 0.720166 | 0.759933 |
| GO:0051784 | negative regulation of nuclear division | 36 | 1 | 1 | 0.502513 | 0.446429 | 0.720166 | 0.759933 |
| GO:0055037 | recycling endosome | 50 | 1 | 1 | 0.502513 | 0.446429 | 0.720166 | 0.759933 |
| GO:0055080 | cation homeostasis | 338 | 1 | 1 | 0.502513 | 0.446429 | 0.720166 | 0.759933 |
| GO:0060249 | anatomical structure homeostasis | 138 | 1 | 1 | 0.502513 | 0.446429 | 0.720166 | 0.759933 |
| GO:0061134 | peptidase regulator activity | 189 | 1 | 1 | 0.502513 | 0.446429 | 0.720166 | 0.759933 |
| GO:0061135 | endopeptidase regulator activity | 157 | 1 | 1 | 0.502513 | 0.446429 | 0.720166 | 0.759933 |
| GO:0070647 | protein modification by small protein conjugation or removal | 409 | 1 | 1 | 0.502513 | 0.446429 | 0.720166 | 0.759933 |
| GO:0070663 | regulation of leukocyte proliferation | 107 | 1 | 1 | 0.502513 | 0.446429 | 0.720166 | 0.759933 |
| GO:0070665 | positive regulation of leukocyte proliferation | 77 | 1 | 1 | 0.502513 | 0.446429 | 0.720166 | 0.759933 |
| GO:0070851 | growth factor receptor binding | 83 | 1 | 1 | 0.502513 | 0.446429 | 0.720166 | 0.759933 |
| GO:0071173 | spindle assembly checkpoint | 32 | 1 | 1 | 0.502513 | 0.446429 | 0.720166 | 0.759933 |
| GO:0071174 | mitotic cell cycle spindle checkpoint | 32 | 1 | 1 | 0.502513 | 0.446429 | 0.720166 | 0.759933 |
| GO:0071345 | cellular response to cytokine stimulus | 228 | 1 | 1 | 0.502513 | 0.446429 | 0.720166 | 0.759933 |
| GO:0071356 | cellular response to tumor necrosis factor | 33 | 1 | 1 | 0.502513 | 0.446429 | 0.720166 | 0.759933 |
| GO:0071843 | cellular component biogenesis at cellular level | 208 | 1 | 1 | 0.502513 | 0.446429 | 0.720166 | 0.759933 |
| GO:0072376 | protein activation cascade | 79 | 1 | 1 | 0.502513 | 0.446429 | 0.720166 | 0.759933 |
| GO:0072522 | purine-containing compound biosynthetic process | 256 | 1 | 1 | 0.502513 | 0.446429 | 0.720166 | 0.759933 |
| GO:0090066 | regulation of anatomical structure size | 450 | 1 | 1 | 0.502513 | 0.446429 | 0.720166 | 0.759933 |
| KEGG:00140 | KEGG: Steroid hormone biosynthesis | 56 | 1 | 1 | 0.502513 | 0.446429 | 0.720166 | 0.759933 |
| KEGG:04060 | KEGG: Cytokine-cytokine receptor interaction | 266 | 1 | 1 | 0.502513 | 0.446429 | 0.720166 | 0.759933 |
| KEGG:04110 | KEGG: Cell cycle | 128 | 1 | 1 | 0.502513 | 0.446429 | 0.720166 | 0.759933 |
| KEGG:05223 | KEGG: Non-small cell lung cancer | 54 | 1 | 1 | 0.502513 | 0.446429 | 0.720166 | 0.759933 |
| NCI:156 | Regulation of RAC1 activity | 40 | 1 | 1 | 0.502513 | 0.446429 | 0.720166 | 0.759933 |
| NCI:165 | Regulation of RhoA activity | 45 | 1 | 1 | 0.502513 | 0.446429 | 0.720166 | 0.759933 |
| NCI:18 | Arf6 signaling events | 35 | 1 | 1 | 0.502513 | 0.446429 | 0.720166 | 0.759933 |
| NCI:23 | Aurora B signaling | 39 | 1 | 1 | 0.502513 | 0.446429 | 0.720166 | 0.759933 |
| NCI:31 | Regulation of nuclear beta catenin signaling and target gene transcription | 79 | 1 | 1 | 0.502513 | 0.446429 | 0.720166 | 0.759933 |
| NCI:65 | ErbB2/ErbB3 signaling events | 43 | 1 | 1 | 0.502513 | 0.446429 | 0.720166 | 0.759933 |
| PF00008 | PFAM: EGF-like domain | 90 | 1 | 1 | 0.502513 | 0.446429 | 0.720166 | 0.759933 |
| PF00041 | PFAM: Fibronectin type III domain | 153 | 1 | 1 | 0.502513 | 0.446429 | 0.720166 | 0.759933 |
| PF00071 | PFAM: Ras family | 144 | 1 | 1 | 0.502513 | 0.446429 | 0.720166 | 0.759933 |
| PF00084 | PFAM: Sushi domain (SCR repeat) | 54 | 1 | 1 | 0.502513 | 0.446429 | 0.720166 | 0.759933 |
| PF00397 | PFAM: WW domain | 46 | 1 | 1 | 0.502513 | 0.446429 | 0.720166 | 0.759933 |
| PF00566 | PFAM: TBC domain | 53 | 1 | 1 | 0.502513 | 0.446429 | 0.720166 | 0.759933 |
| PF00989 | PFAM: PAS fold | 26 | 1 | 1 | 0.502513 | 0.446429 | 0.720166 | 0.759933 |
| PF01049 | PFAM: Cadherin cytoplasmic region | 26 | 1 | 1 | 0.502513 | 0.446429 | 0.720166 | 0.759933 |
| PF01437 | PFAM: Plexin repeat | 32 | 1 | 1 | 0.502513 | 0.446429 | 0.720166 | 0.759933 |
| PF02931 | PFAM: Neurotransmitter-gated ion-channel ligand binding domain | 47 | 1 | 1 | 0.502513 | 0.446429 | 0.720166 | 0.759933 |
| PF02932 | PFAM: Neurotransmitter-gated ion-channel transmembrane region | 46 | 1 | 1 | 0.502513 | 0.446429 | 0.720166 | 0.759933 |
| REACT:1054 | REACT: Integrin alphaIIb beta3 signaling | 27 | 1 | 1 | 0.502513 | 0.446429 | 0.720166 | 0.759933 |
| REACT:127 | REACT: Metabolism of proteins | 296 | 1 | 1 | 0.502513 | 0.446429 | 0.720166 | 0.759933 |
| REACT:129 | REACT: Signaling in Immune system | 506 | 1 | 1 | 0.502513 | 0.446429 | 0.720166 | 0.759933 |
| REACT:141 | REACT: Platelet Aggregation (Plug Formation) | 35 | 1 | 1 | 0.502513 | 0.446429 | 0.720166 | 0.759933 |
| REACT:172 | REACT: Platelet degranulation | 78 | 1 | 1 | 0.502513 | 0.446429 | 0.720166 | 0.759933 |
| REACT:190 | REACT: Signalling by NGF | 220 | 1 | 1 | 0.502513 | 0.446429 | 0.720166 | 0.759933 |
| REACT:261 | REACT: Factors involved in megakaryocyte development and platelet production | 124 | 1 | 1 | 0.502513 | 0.446429 | 0.720166 | 0.759933 |
| REACT:345 | REACT: Metabolism of lipids and lipoproteins | 289 | 1 | 1 | 0.502513 | 0.446429 | 0.720166 | 0.759933 |
| REACT:63 | REACT: Integrin cell surface interactions | 85 | 1 | 1 | 0.502513 | 0.446429 | 0.720166 | 0.759933 |
| REACT:669 | REACT: Response to elevated platelet cytosolic Ca2+ | 83 | 1 | 1 | 0.502513 | 0.446429 | 0.720166 | 0.759933 |
| REACT:699 | REACT: Generic Transcription Pathway | 85 | 1 | 1 | 0.502513 | 0.446429 | 0.720166 | 0.759933 |
| REACT:966 | REACT: Platelet activation triggers | 83 | 1 | 1 | 0.502513 | 0.446429 | 0.720166 | 0.759933 |
| GO:0006184 | GTP catabolic process | 291 | 3 | 4 | 1.507538 | 1.785714 | 0.723952 | 0.763309 |
| GO:0009143 | nucleoside triphosphate catabolic process | 365 | 3 | 4 | 1.507538 | 1.785714 | 0.723952 | 0.763309 |
| GO:0009146 | purine nucleoside triphosphate catabolic process | 362 | 3 | 4 | 1.507538 | 1.785714 | 0.723952 | 0.763309 |
| GO:0009154 | purine ribonucleotide catabolic process | 365 | 3 | 4 | 1.507538 | 1.785714 | 0.723952 | 0.763309 |
| GO:0009199 | ribonucleoside triphosphate metabolic process | 434 | 3 | 4 | 1.507538 | 1.785714 | 0.723952 | 0.763309 |
| GO:0009203 | ribonucleoside triphosphate catabolic process | 361 | 3 | 4 | 1.507538 | 1.785714 | 0.723952 | 0.763309 |
| GO:0009205 | purine ribonucleoside triphosphate metabolic process | 433 | 3 | 4 | 1.507538 | 1.785714 | 0.723952 | 0.763309 |
| GO:0009207 | purine ribonucleoside triphosphate catabolic process | 361 | 3 | 4 | 1.507538 | 1.785714 | 0.723952 | 0.763309 |
| GO:0009261 | ribonucleotide catabolic process | 367 | 3 | 4 | 1.507538 | 1.785714 | 0.723952 | 0.763309 |
| GO:0045595 | regulation of cell differentiation | 633 | 3 | 4 | 1.507538 | 1.785714 | 0.723952 | 0.763309 |
| GO:0046039 | GTP metabolic process | 301 | 3 | 4 | 1.507538 | 1.785714 | 0.723952 | 0.763309 |
| GO:0051301 | cell division | 374 | 3 | 4 | 1.507538 | 1.785714 | 0.723952 | 0.763309 |
| GO:0000323 | lytic vacuole | 272 | 2 | 3 | 1.005025 | 1.339286 | 0.774848 | 0.76903 |
| GO:0005216 | ion channel activity | 391 | 2 | 3 | 1.005025 | 1.339286 | 0.774848 | 0.76903 |
| GO:0005730 | nucleolus | 505 | 2 | 3 | 1.005025 | 1.339286 | 0.774848 | 0.76903 |
| GO:0005764 | lysosome | 272 | 2 | 3 | 1.005025 | 1.339286 | 0.774848 | 0.76903 |
| GO:0005773 | vacuole | 319 | 2 | 3 | 1.005025 | 1.339286 | 0.774848 | 0.76903 |
| GO:0006140 | regulation of nucleotide metabolic process | 301 | 2 | 3 | 1.005025 | 1.339286 | 0.774848 | 0.76903 |
| GO:0006396 | RNA processing | 612 | 2 | 3 | 1.005025 | 1.339286 | 0.774848 | 0.76903 |
| GO:0006399 | tRNA metabolic process | 122 | 2 | 3 | 1.005025 | 1.339286 | 0.774848 | 0.76903 |
| GO:0007276 | gamete generation | 414 | 2 | 3 | 1.005025 | 1.339286 | 0.774848 | 0.76903 |
| GO:0007601 | visual perception | 221 | 2 | 3 | 1.005025 | 1.339286 | 0.774848 | 0.76903 |
| GO:0008083 | growth factor activity | 164 | 2 | 3 | 1.005025 | 1.339286 | 0.774848 | 0.76903 |
| GO:0015267 | channel activity | 414 | 2 | 3 | 1.005025 | 1.339286 | 0.774848 | 0.76903 |
| GO:0015630 | microtubule cytoskeleton | 652 | 2 | 3 | 1.005025 | 1.339286 | 0.774848 | 0.76903 |
| GO:0022803 | passive transmembrane transporter activity | 415 | 2 | 3 | 1.005025 | 1.339286 | 0.774848 | 0.76903 |
| GO:0022836 | gated channel activity | 308 | 2 | 3 | 1.005025 | 1.339286 | 0.774848 | 0.76903 |
| GO:0022838 | substrate-specific channel activity | 400 | 2 | 3 | 1.005025 | 1.339286 | 0.774848 | 0.76903 |
| GO:0030246 | carbohydrate binding | 380 | 2 | 3 | 1.005025 | 1.339286 | 0.774848 | 0.76903 |
| GO:0030811 | regulation of nucleotide catabolic process | 172 | 2 | 3 | 1.005025 | 1.339286 | 0.774848 | 0.76903 |
| GO:0031329 | regulation of cellular catabolic process | 285 | 2 | 3 | 1.005025 | 1.339286 | 0.774848 | 0.76903 |
| GO:0032504 | multicellular organism reproduction | 519 | 2 | 3 | 1.005025 | 1.339286 | 0.774848 | 0.76903 |
| GO:0033121 | regulation of purine nucleotide catabolic process | 172 | 2 | 3 | 1.005025 | 1.339286 | 0.774848 | 0.76903 |
| GO:0042110 | T cell activation | 242 | 2 | 3 | 1.005025 | 1.339286 | 0.774848 | 0.76903 |
| GO:0048609 | multicellular organismal reproductive process | 519 | 2 | 3 | 1.005025 | 1.339286 | 0.774848 | 0.76903 |
| GO:0050953 | sensory perception of light stimulus | 221 | 2 | 3 | 1.005025 | 1.339286 | 0.774848 | 0.76903 |
| KEGG:04080 | KEGG: Neuroactive ligand-receptor interaction | 272 | 2 | 3 | 1.005025 | 1.339286 | 0.774848 | 0.76903 |
| PF07974 | PFAM: EGF-like domain | 58 | 2 | 3 | 1.005025 | 1.339286 | 0.774848 | 0.76903 |
| GO:0007265 | Ras protein signal transduction | 343 | 4 | 6 | 2.01005 | 2.678571 | 0.778217 | 0.770417 |
| GO:0005096 | GTPase activator activity | 258 | 3 | 5 | 1.507538 | 2.232143 | 0.8156 | 0.773484 |
| GO:0009141 | nucleoside triphosphate metabolic process | 444 | 3 | 5 | 1.507538 | 2.232143 | 0.8156 | 0.773484 |
| GO:0009144 | purine nucleoside triphosphate metabolic process | 436 | 3 | 5 | 1.507538 | 2.232143 | 0.8156 | 0.773484 |
| GO:0019725 | cellular homeostasis | 542 | 3 | 5 | 1.507538 | 2.232143 | 0.8156 | 0.773484 |
| GO:0031012 | extracellular matrix | 370 | 3 | 5 | 1.507538 | 2.232143 | 0.8156 | 0.773484 |
| GO:0001501 | skeletal system development | 303 | 1 | 2 | 0.502513 | 0.892857 | 0.852439 | 0.809441 |
| GO:0001655 | urogenital system development | 159 | 1 | 2 | 0.502513 | 0.892857 | 0.852439 | 0.809441 |
| GO:0001822 | kidney development | 119 | 1 | 2 | 0.502513 | 0.892857 | 0.852439 | 0.809441 |
| GO:0005097 | Rab GTPase activator activity | 51 | 1 | 2 | 0.502513 | 0.892857 | 0.852439 | 0.809441 |
| GO:0005743 | mitochondrial inner membrane | 322 | 1 | 2 | 0.502513 | 0.892857 | 0.852439 | 0.809441 |
| GO:0006260 | DNA replication | 245 | 1 | 2 | 0.502513 | 0.892857 | 0.852439 | 0.809441 |
| GO:0006766 | vitamin metabolic process | 116 | 1 | 2 | 0.502513 | 0.892857 | 0.852439 | 0.809441 |
| GO:0006873 | cellular ion homeostasis | 440 | 1 | 2 | 0.502513 | 0.892857 | 0.852439 | 0.809441 |
| GO:0006997 | nucleus organization | 32 | 1 | 2 | 0.502513 | 0.892857 | 0.852439 | 0.809441 |
| GO:0008092 | cytoskeletal protein binding | 530 | 1 | 2 | 0.502513 | 0.892857 | 0.852439 | 0.809441 |
| GO:0008284 | positive regulation of cell proliferation | 485 | 1 | 2 | 0.502513 | 0.892857 | 0.852439 | 0.809441 |
| GO:0008406 | gonad development | 153 | 1 | 2 | 0.502513 | 0.892857 | 0.852439 | 0.809441 |
| GO:0008509 | anion transmembrane transporter activity | 150 | 1 | 2 | 0.502513 | 0.892857 | 0.852439 | 0.809441 |
| GO:0008585 | female gonad development | 78 | 1 | 2 | 0.502513 | 0.892857 | 0.852439 | 0.809441 |
| GO:0009116 | nucleoside metabolic process | 83 | 1 | 2 | 0.502513 | 0.892857 | 0.852439 | 0.809441 |
| GO:0009119 | ribonucleoside metabolic process | 58 | 1 | 2 | 0.502513 | 0.892857 | 0.852439 | 0.809441 |
| GO:0009607 | response to biotic stimulus | 524 | 1 | 2 | 0.502513 | 0.892857 | 0.852439 | 0.809441 |
| GO:0023057 | negative regulation of signaling | 411 | 1 | 2 | 0.502513 | 0.892857 | 0.852439 | 0.809441 |
| GO:0030217 | T cell differentiation | 104 | 1 | 2 | 0.502513 | 0.892857 | 0.852439 | 0.809441 |
| GO:0030424 | axon | 187 | 1 | 2 | 0.502513 | 0.892857 | 0.852439 | 0.809441 |
| GO:0032313 | regulation of Rab GTPase activity | 50 | 1 | 2 | 0.502513 | 0.892857 | 0.852439 | 0.809441 |
| GO:0032482 | Rab protein signal transduction | 51 | 1 | 2 | 0.502513 | 0.892857 | 0.852439 | 0.809441 |
| GO:0032483 | regulation of Rab protein signal transduction | 50 | 1 | 2 | 0.502513 | 0.892857 | 0.852439 | 0.809441 |
| GO:0032943 | mononuclear cell proliferation | 129 | 1 | 2 | 0.502513 | 0.892857 | 0.852439 | 0.809441 |
| GO:0034504 | protein localization to nucleus | 173 | 1 | 2 | 0.502513 | 0.892857 | 0.852439 | 0.809441 |
| GO:0034702 | ion channel complex | 211 | 1 | 2 | 0.502513 | 0.892857 | 0.852439 | 0.809441 |
| GO:0042278 | purine nucleoside metabolic process | 35 | 1 | 2 | 0.502513 | 0.892857 | 0.852439 | 0.809441 |
| GO:0042578 | phosphoric ester hydrolase activity | 346 | 1 | 2 | 0.502513 | 0.892857 | 0.852439 | 0.809441 |
| GO:0045087 | innate immune response | 416 | 1 | 2 | 0.502513 | 0.892857 | 0.852439 | 0.809441 |
| GO:0046128 | purine ribonucleoside metabolic process | 35 | 1 | 2 | 0.502513 | 0.892857 | 0.852439 | 0.809441 |
| GO:0046651 | lymphocyte proliferation | 127 | 1 | 2 | 0.502513 | 0.892857 | 0.852439 | 0.809441 |
| GO:0048471 | perinuclear region of cytoplasm | 353 | 1 | 2 | 0.502513 | 0.892857 | 0.852439 | 0.809441 |
| GO:0051707 | response to other organism | 432 | 1 | 2 | 0.502513 | 0.892857 | 0.852439 | 0.809441 |
| GO:0055082 | cellular chemical homeostasis | 455 | 1 | 2 | 0.502513 | 0.892857 | 0.852439 | 0.809441 |
| GO:0070661 | leukocyte proliferation | 132 | 1 | 2 | 0.502513 | 0.892857 | 0.852439 | 0.809441 |
| GO:0072001 | renal system development | 123 | 1 | 2 | 0.502513 | 0.892857 | 0.852439 | 0.809441 |
| PF00400 | PFAM: WD domain, G-beta repeat | 265 | 1 | 2 | 0.502513 | 0.892857 | 0.852439 | 0.809441 |
| PF00595 | PFAM: PDZ domain (Also known as DHR or GLGF) | 152 | 1 | 2 | 0.502513 | 0.892857 | 0.852439 | 0.809441 |
| PF01391 | PFAM: Collagen triple helix repeat (20 copies) | 83 | 1 | 2 | 0.502513 | 0.892857 | 0.852439 | 0.809441 |
| PF02210 | PFAM: Laminin G domain | 48 | 1 | 2 | 0.502513 | 0.892857 | 0.852439 | 0.809441 |
| REACT:977 | REACT: G alpha (s) signalling events | 125 | 1 | 2 | 0.502513 | 0.892857 | 0.852439 | 0.809441 |
| GO:0019953 | sexual reproduction | 479 | 2 | 4 | 1.005025 | 1.785714 | 0.862188 | 0.817743 |
| GO:0034660 | ncRNA metabolic process | 267 | 2 | 4 | 1.005025 | 1.785714 | 0.862188 | 0.817743 |
| GO:0005509 | calcium ion binding | 648 | 3 | 6 | 1.507538 | 2.678571 | 0.880084 | 0.817593 |
| GO:0008047 | enzyme activator activity | 389 | 3 | 6 | 1.507538 | 2.678571 | 0.880084 | 0.817593 |
| GO:0046578 | regulation of Ras protein signal transduction | 240 | 3 | 6 | 1.507538 | 2.678571 | 0.880084 | 0.817593 |
| GO:0030695 | GTPase regulator activity | 454 | 4 | 8 | 2.01005 | 3.571429 | 0.897295 | 0.816831 |
| GO:0060589 | nucleoside-triphosphatase regulator activity | 466 | 4 | 8 | 2.01005 | 3.571429 | 0.897295 | 0.816831 |
| GO:0005578 | proteinaceous extracellular matrix | 312 | 2 | 5 | 1.005025 | 2.232143 | 0.917482 | 0.822201 |
| GO:0031967 | organelle envelope | 710 | 2 | 5 | 1.005025 | 2.232143 | 0.917482 | 0.822201 |
| GO:0031975 | envelope | 722 | 2 | 5 | 1.005025 | 2.232143 | 0.917482 | 0.822201 |
| GO:0002694 | regulation of leukocyte activation | 240 | 1 | 3 | 0.502513 | 1.339286 | 0.922355 | 0.832176 |
| GO:0002696 | positive regulation of leukocyte activation | 184 | 1 | 3 | 0.502513 | 1.339286 | 0.922355 | 0.832176 |
| GO:0005099 | Ras GTPase activator activity | 100 | 1 | 3 | 0.502513 | 1.339286 | 0.922355 | 0.832176 |
| GO:0005815 | microtubule organizing center | 321 | 1 | 3 | 0.502513 | 1.339286 | 0.922355 | 0.832176 |
| GO:0006461 | protein complex assembly | 561 | 1 | 3 | 0.502513 | 1.339286 | 0.922355 | 0.832176 |
| GO:0008236 | serine-type peptidase activity | 178 | 1 | 3 | 0.502513 | 1.339286 | 0.922355 | 0.832176 |
| GO:0016667 | oxidoreductase activity, acting on a sulfur group of donors | 51 | 1 | 3 | 0.502513 | 1.339286 | 0.922355 | 0.832176 |
| GO:0017171 | serine hydrolase activity | 181 | 1 | 3 | 0.502513 | 1.339286 | 0.922355 | 0.832176 |
| GO:0019866 | organelle inner membrane | 350 | 1 | 3 | 0.502513 | 1.339286 | 0.922355 | 0.832176 |
| GO:0030001 | metal ion transport | 540 | 1 | 3 | 0.502513 | 1.339286 | 0.922355 | 0.832176 |
| GO:0032318 | regulation of Ras GTPase activity | 131 | 1 | 3 | 0.502513 | 1.339286 | 0.922355 | 0.832176 |
| GO:0033124 | regulation of GTP catabolic process | 154 | 1 | 3 | 0.502513 | 1.339286 | 0.922355 | 0.832176 |
| GO:0033365 | protein localization to organelle | 283 | 1 | 3 | 0.502513 | 1.339286 | 0.922355 | 0.832176 |
| GO:0043087 | regulation of GTPase activity | 153 | 1 | 3 | 0.502513 | 1.339286 | 0.922355 | 0.832176 |
| GO:0045454 | cell redox homeostasis | 67 | 1 | 3 | 0.502513 | 1.339286 | 0.922355 | 0.832176 |
| GO:0050801 | ion homeostasis | 473 | 1 | 3 | 0.502513 | 1.339286 | 0.922355 | 0.832176 |
| GO:0050865 | regulation of cell activation | 251 | 1 | 3 | 0.502513 | 1.339286 | 0.922355 | 0.832176 |
| GO:0050867 | positive regulation of cell activation | 188 | 1 | 3 | 0.502513 | 1.339286 | 0.922355 | 0.832176 |
| GO:0051249 | regulation of lymphocyte activation | 219 | 1 | 3 | 0.502513 | 1.339286 | 0.922355 | 0.832176 |
| GO:0051251 | positive regulation of lymphocyte activation | 172 | 1 | 3 | 0.502513 | 1.339286 | 0.922355 | 0.832176 |
| GO:0051259 | protein oligomerization | 220 | 1 | 3 | 0.502513 | 1.339286 | 0.922355 | 0.832176 |
| GO:0051260 | protein homooligomerization | 122 | 1 | 3 | 0.502513 | 1.339286 | 0.922355 | 0.832176 |
| GO:0070271 | protein complex biogenesis | 563 | 1 | 3 | 0.502513 | 1.339286 | 0.922355 | 0.832176 |
| GO:0071822 | protein complex subunit organization | 673 | 1 | 3 | 0.502513 | 1.339286 | 0.922355 | 0.832176 |
| PF00090 | PFAM: Thrombospondin type 1 domain | 63 | 1 | 3 | 0.502513 | 1.339286 | 0.922355 | 0.832176 |
| GO:0005083 | small GTPase regulator activity | 291 | 3 | 7 | 1.507538 | 3.125 | 0.923741 | 0.832208 |
| GO:0051056 | regulation of small GTPase mediated signal transduction | 354 | 3 | 7 | 1.507538 | 3.125 | 0.923741 | 0.832208 |
| GO:0015075 | ion transmembrane transporter activity | 730 | 2 | 6 | 1.005025 | 2.678571 | 0.951443 | 0.834138 |
| GO:0070011 | peptidase activity, acting on L-amino acid peptides | 557 | 2 | 6 | 1.005025 | 2.678571 | 0.951443 | 0.834138 |
| GO:0004175 | endopeptidase activity | 374 | 1 | 4 | 0.502513 | 1.785714 | 0.959232 | 0.842497 |
| GO:0006812 | cation transport | 618 | 1 | 4 | 0.502513 | 1.785714 | 0.959232 | 0.842497 |
| GO:0007186 | G-protein coupled receptor protein signaling pathway | 569 | 1 | 4 | 0.502513 | 1.785714 | 0.959232 | 0.842497 |
| GO:0009986 | cell surface | 373 | 1 | 4 | 0.502513 | 1.785714 | 0.959232 | 0.842497 |
| GO:0048878 | chemical homeostasis | 611 | 1 | 4 | 0.502513 | 1.785714 | 0.959232 | 0.842497 |
| KEGG:00230 | KEGG: Purine metabolism | 161 | 1 | 4 | 0.502513 | 1.785714 | 0.959232 | 0.842497 |
| GO:0008233 | peptidase activity | 576 | 2 | 7 | 1.005025 | 3.125 | 0.971831 | 0.842899 |
| GO:0010942 | positive regulation of cell death | 507 | 1 | 5 | 0.502513 | 2.232143 | 0.978641 | 0.845193 |
| GO:0043065 | positive regulation of apoptosis | 495 | 1 | 5 | 0.502513 | 2.232143 | 0.978641 | 0.845193 |
| GO:0043068 | positive regulation of programmed cell death | 499 | 1 | 5 | 0.502513 | 2.232143 | 0.978641 | 0.845193 |
| BIOC:72 | BIOC: NO1 Pathway | 28 | 0 | 1 | 0 | 0.446429 | 1 | 1 |
| GO:0000018 | regulation of DNA recombination | 26 | 0 | 1 | 0 | 0.446429 | 1 | 1 |
| GO:0000049 | tRNA binding | 25 | 0 | 1 | 0 | 0.446429 | 1 | 1 |
| GO:0000086 | G2/M transition of mitotic cell cycle | 115 | 0 | 1 | 0 | 0.446429 | 1 | 1 |
| GO:0000272 | polysaccharide catabolic process | 44 | 0 | 1 | 0 | 0.446429 | 1 | 1 |
| GO:0000287 | magnesium ion binding | 168 | 0 | 2 | 0 | 0.892857 | 1 | 1 |
| GO:0000302 | response to reactive oxygen species | 92 | 0 | 1 | 0 | 0.446429 | 1 | 1 |
| GO:0000313 | organellar ribosome | 49 | 0 | 1 | 0 | 0.446429 | 1 | 1 |
| GO:0001101 | response to acid | 58 | 0 | 1 | 0 | 0.446429 | 1 | 1 |
| GO:0001503 | ossification | 196 | 0 | 1 | 0 | 0.446429 | 1 | 1 |
| GO:0001508 | regulation of action potential | 73 | 0 | 2 | 0 | 0.892857 | 1 | 1 |
| GO:0001541 | ovarian follicle development | 45 | 0 | 2 | 0 | 0.892857 | 1 | 1 |
| GO:0001649 | osteoblast differentiation | 93 | 0 | 1 | 0 | 0.446429 | 1 | 1 |
| GO:0001653 | peptide receptor activity | 115 | 0 | 1 | 0 | 0.446429 | 1 | 1 |
| GO:0001727 | lipid kinase activity | 29 | 0 | 1 | 0 | 0.446429 | 1 | 1 |
| GO:0001816 | cytokine production | 284 | 0 | 1 | 0 | 0.446429 | 1 | 1 |
| GO:0001817 | regulation of cytokine production | 254 | 0 | 1 | 0 | 0.446429 | 1 | 1 |
| GO:0001819 | positive regulation of cytokine production | 123 | 0 | 1 | 0 | 0.446429 | 1 | 1 |
| GO:0001889 | liver development | 64 | 0 | 2 | 0 | 0.892857 | 1 | 1 |
| GO:0001906 | cell killing | 64 | 0 | 1 | 0 | 0.446429 | 1 | 1 |
| GO:0001909 | leukocyte mediated cytotoxicity | 40 | 0 | 1 | 0 | 0.446429 | 1 | 1 |
| GO:0001910 | regulation of leukocyte mediated cytotoxicity | 29 | 0 | 1 | 0 | 0.446429 | 1 | 1 |
| GO:0002020 | protease binding | 36 | 0 | 1 | 0 | 0.446429 | 1 | 1 |
| GO:0002062 | chondrocyte differentiation | 41 | 0 | 1 | 0 | 0.446429 | 1 | 1 |
| GO:0002218 | activation of innate immune response | 97 | 0 | 1 | 0 | 0.446429 | 1 | 1 |
| GO:0002221 | pattern recognition receptor signaling pathway | 94 | 0 | 1 | 0 | 0.446429 | 1 | 1 |
| GO:0002250 | adaptive immune response | 146 | 0 | 1 | 0 | 0.446429 | 1 | 1 |
| GO:0002377 | immunoglobulin production | 51 | 0 | 1 | 0 | 0.446429 | 1 | 1 |
| GO:0002440 | production of molecular mediator of immune response | 70 | 0 | 1 | 0 | 0.446429 | 1 | 1 |
| GO:0002443 | leukocyte mediated immunity | 165 | 0 | 1 | 0 | 0.446429 | 1 | 1 |
| GO:0002449 | lymphocyte mediated immunity | 139 | 0 | 1 | 0 | 0.446429 | 1 | 1 |
| GO:0002456 | T cell mediated immunity | 32 | 0 | 1 | 0 | 0.446429 | 1 | 1 |
| GO:0002460 | adaptive immune response based on somatic recombination of immune receptors built from immunoglobulin superfamily domains | 143 | 0 | 1 | 0 | 0.446429 | 1 | 1 |
| GO:0002526 | acute inflammatory response | 79 | 0 | 1 | 0 | 0.446429 | 1 | 1 |
| GO:0002683 | negative regulation of immune system process | 97 | 0 | 1 | 0 | 0.446429 | 1 | 1 |
| GO:0002697 | regulation of immune effector process | 148 | 0 | 1 | 0 | 0.446429 | 1 | 1 |
| GO:0002698 | negative regulation of immune effector process | 25 | 0 | 1 | 0 | 0.446429 | 1 | 1 |
| GO:0002703 | regulation of leukocyte mediated immunity | 67 | 0 | 1 | 0 | 0.446429 | 1 | 1 |
| GO:0002706 | regulation of lymphocyte mediated immunity | 58 | 0 | 1 | 0 | 0.446429 | 1 | 1 |
| GO:0002709 | regulation of T cell mediated immunity | 27 | 0 | 1 | 0 | 0.446429 | 1 | 1 |
| GO:0002758 | innate immune response-activating signal transduction | 97 | 0 | 1 | 0 | 0.446429 | 1 | 1 |
| GO:0002819 | regulation of adaptive immune response | 62 | 0 | 1 | 0 | 0.446429 | 1 | 1 |
| GO:0002822 | regulation of adaptive immune response based on somatic recombination of immune receptors built from immunoglobulin superfamily domains | 61 | 0 | 1 | 0 | 0.446429 | 1 | 1 |
| GO:0003012 | muscle system process | 225 | 0 | 1 | 0 | 0.446429 | 1 | 1 |
| GO:0003702 | RNA polymerase II transcription factor activity | 256 | 0 | 2 | 0 | 0.892857 | 1 | 1 |
| GO:0003713 | transcription coactivator activity | 223 | 0 | 1 | 0 | 0.446429 | 1 | 1 |
| GO:0003735 | structural constituent of ribosome | 158 | 0 | 1 | 0 | 0.446429 | 1 | 1 |
| GO:0003823 | antigen binding | 62 | 0 | 1 | 0 | 0.446429 | 1 | 1 |
| GO:0004180 | carboxypeptidase activity | 43 | 0 | 1 | 0 | 0.446429 | 1 | 1 |
| GO:0004222 | metalloendopeptidase activity | 107 | 0 | 3 | 0 | 1.339286 | 1 | 1 |
| GO:0004428 | inositol or phosphatidylinositol kinase activity | 36 | 0 | 1 | 0 | 0.446429 | 1 | 1 |
| GO:0004553 | hydrolase activity, hydrolyzing O-glycosyl compounds | 89 | 0 | 1 | 0 | 0.446429 | 1 | 1 |
| GO:0004714 | transmembrane receptor protein tyrosine kinase activity | 66 | 0 | 1 | 0 | 0.446429 | 1 | 1 |
| GO:0004896 | cytokine receptor activity | 57 | 0 | 1 | 0 | 0.446429 | 1 | 1 |
| GO:0005044 | scavenger receptor activity | 45 | 0 | 1 | 0 | 0.446429 | 1 | 1 |
| GO:0005100 | Rho GTPase activator activity | 30 | 0 | 1 | 0 | 0.446429 | 1 | 1 |
| GO:0005125 | cytokine activity | 205 | 0 | 2 | 0 | 0.892857 | 1 | 1 |
| GO:0005178 | integrin binding | 67 | 0 | 1 | 0 | 0.446429 | 1 | 1 |
| GO:0005179 | hormone activity | 108 | 0 | 1 | 0 | 0.446429 | 1 | 1 |
| GO:0005244 | voltage-gated ion channel activity | 190 | 0 | 1 | 0 | 0.446429 | 1 | 1 |
| GO:0005261 | cation channel activity | 272 | 0 | 2 | 0 | 0.892857 | 1 | 1 |
| GO:0005267 | potassium channel activity | 130 | 0 | 1 | 0 | 0.446429 | 1 | 1 |
| GO:0005518 | collagen binding | 40 | 0 | 1 | 0 | 0.446429 | 1 | 1 |
| GO:0005529 | sugar binding | 200 | 0 | 3 | 0 | 1.339286 | 1 | 1 |
| GO:0005625 | soluble fraction | 332 | 0 | 1 | 0 | 0.446429 | 1 | 1 |
| GO:0005635 | nuclear envelope | 248 | 0 | 3 | 0 | 1.339286 | 1 | 1 |
| GO:0005637 | nuclear inner membrane | 28 | 0 | 1 | 0 | 0.446429 | 1 | 1 |
| GO:0005643 | nuclear pore | 71 | 0 | 1 | 0 | 0.446429 | 1 | 1 |
| GO:0005761 | mitochondrial ribosome | 49 | 0 | 1 | 0 | 0.446429 | 1 | 1 |
| GO:0005791 | rough endoplasmic reticulum | 36 | 0 | 1 | 0 | 0.446429 | 1 | 1 |
| GO:0005813 | centrosome | 216 | 0 | 2 | 0 | 0.892857 | 1 | 1 |
| GO:0005814 | centriole | 39 | 0 | 1 | 0 | 0.446429 | 1 | 1 |
| GO:0005819 | spindle | 179 | 0 | 2 | 0 | 0.892857 | 1 | 1 |
| GO:0005840 | ribosome | 199 | 0 | 1 | 0 | 0.446429 | 1 | 1 |
| GO:0005911 | cell-cell junction | 209 | 0 | 1 | 0 | 0.446429 | 1 | 1 |
| GO:0005923 | tight junction | 78 | 0 | 1 | 0 | 0.446429 | 1 | 1 |
| GO:0006091 | generation of precursor metabolites and energy | 415 | 0 | 1 | 0 | 0.446429 | 1 | 1 |
| GO:0006144 | purine base metabolic process | 37 | 0 | 1 | 0 | 0.446429 | 1 | 1 |
| GO:0006310 | DNA recombination | 162 | 0 | 1 | 0 | 0.446429 | 1 | 1 |
| GO:0006352 | transcription initiation, DNA-dependent | 106 | 0 | 1 | 0 | 0.446429 | 1 | 1 |
| GO:0006354 | transcription elongation, DNA-dependent | 88 | 0 | 1 | 0 | 0.446429 | 1 | 1 |
| GO:0006364 | rRNA processing | 95 | 0 | 1 | 0 | 0.446429 | 1 | 1 |
| GO:0006367 | transcription initiation from RNA polymerase II promoter | 77 | 0 | 1 | 0 | 0.446429 | 1 | 1 |
| GO:0006368 | transcription elongation from RNA polymerase II promoter | 66 | 0 | 1 | 0 | 0.446429 | 1 | 1 |
| GO:0006486 | protein glycosylation | 167 | 0 | 1 | 0 | 0.446429 | 1 | 1 |
| GO:0006487 | protein N-linked glycosylation | 94 | 0 | 1 | 0 | 0.446429 | 1 | 1 |
| GO:0006493 | protein O-linked glycosylation | 26 | 0 | 1 | 0 | 0.446429 | 1 | 1 |
| GO:0006575 | cellular modified amino acid metabolic process | 115 | 0 | 1 | 0 | 0.446429 | 1 | 1 |
| GO:0006626 | protein targeting to mitochondrion | 33 | 0 | 1 | 0 | 0.446429 | 1 | 1 |
| GO:0006643 | membrane lipid metabolic process | 99 | 0 | 1 | 0 | 0.446429 | 1 | 1 |
| GO:0006664 | glycolipid metabolic process | 32 | 0 | 1 | 0 | 0.446429 | 1 | 1 |
| GO:0006694 | steroid biosynthetic process | 128 | 0 | 1 | 0 | 0.446429 | 1 | 1 |
| GO:0006725 | cellular aromatic compound metabolic process | 193 | 0 | 1 | 0 | 0.446429 | 1 | 1 |
| GO:0006767 | water-soluble vitamin metabolic process | 66 | 0 | 1 | 0 | 0.446429 | 1 | 1 |
| GO:0006813 | potassium ion transport | 167 | 0 | 2 | 0 | 0.892857 | 1 | 1 |
| GO:0006814 | sodium ion transport | 141 | 0 | 2 | 0 | 0.892857 | 1 | 1 |
| GO:0006820 | anion transport | 140 | 0 | 1 | 0 | 0.446429 | 1 | 1 |
| GO:0006821 | chloride transport | 44 | 0 | 1 | 0 | 0.446429 | 1 | 1 |
| GO:0006839 | mitochondrial transport | 81 | 0 | 1 | 0 | 0.446429 | 1 | 1 |
| GO:0006885 | regulation of pH | 38 | 0 | 1 | 0 | 0.446429 | 1 | 1 |
| GO:0006898 | receptor-mediated endocytosis | 84 | 0 | 1 | 0 | 0.446429 | 1 | 1 |
| GO:0006916 | anti-apoptosis | 241 | 0 | 1 | 0 | 0.446429 | 1 | 1 |
| GO:0006917 | induction of apoptosis | 362 | 0 | 5 | 0 | 2.232143 | 1 | 1 |
| GO:0006936 | muscle contraction | 211 | 0 | 1 | 0 | 0.446429 | 1 | 1 |
| GO:0006941 | striated muscle contraction | 57 | 0 | 1 | 0 | 0.446429 | 1 | 1 |
| GO:0006968 | cellular defense response | 59 | 0 | 1 | 0 | 0.446429 | 1 | 1 |
| GO:0006979 | response to oxidative stress | 190 | 0 | 2 | 0 | 0.892857 | 1 | 1 |
| GO:0007005 | mitochondrion organization | 146 | 0 | 1 | 0 | 0.446429 | 1 | 1 |
| GO:0007034 | vacuolar transport | 35 | 0 | 1 | 0 | 0.446429 | 1 | 1 |
| GO:0007041 | lysosomal transport | 31 | 0 | 1 | 0 | 0.446429 | 1 | 1 |
| GO:0007272 | ensheathment of neurons | 49 | 0 | 1 | 0 | 0.446429 | 1 | 1 |
| GO:0007292 | female gamete generation | 73 | 0 | 2 | 0 | 0.892857 | 1 | 1 |
| GO:0007338 | single fertilization | 67 | 0 | 1 | 0 | 0.446429 | 1 | 1 |
| GO:0007584 | response to nutrient | 191 | 0 | 1 | 0 | 0.446429 | 1 | 1 |
| GO:0008033 | tRNA processing | 79 | 0 | 2 | 0 | 0.892857 | 1 | 1 |
| GO:0008037 | cell recognition | 61 | 0 | 1 | 0 | 0.446429 | 1 | 1 |
| GO:0008060 | ARF GTPase activator activity | 32 | 0 | 1 | 0 | 0.446429 | 1 | 1 |
| GO:0008188 | neuropeptide receptor activity | 41 | 0 | 1 | 0 | 0.446429 | 1 | 1 |
| GO:0008203 | cholesterol metabolic process | 105 | 0 | 1 | 0 | 0.446429 | 1 | 1 |
| GO:0008237 | metallopeptidase activity | 182 | 0 | 4 | 0 | 1.785714 | 1 | 1 |
| GO:0008238 | exopeptidase activity | 92 | 0 | 1 | 0 | 0.446429 | 1 | 1 |
| GO:0008324 | cation transmembrane transporter activity | 557 | 0 | 5 | 0 | 2.232143 | 1 | 1 |
| GO:0008366 | axon ensheathment | 49 | 0 | 1 | 0 | 0.446429 | 1 | 1 |
| GO:0008378 | galactosyltransferase activity | 32 | 0 | 1 | 0 | 0.446429 | 1 | 1 |
| GO:0008528 | peptide receptor activity, G-protein coupled | 115 | 0 | 1 | 0 | 0.446429 | 1 | 1 |
| GO:0008565 | protein transporter activity | 87 | 0 | 1 | 0 | 0.446429 | 1 | 1 |
| GO:0008624 | induction of apoptosis by extracellular signals | 115 | 0 | 1 | 0 | 0.446429 | 1 | 1 |
| GO:0008629 | induction of apoptosis by intracellular signals | 77 | 0 | 1 | 0 | 0.446429 | 1 | 1 |
| GO:0008630 | DNA damage response, signal transduction resulting in induction of apoptosis | 34 | 0 | 1 | 0 | 0.446429 | 1 | 1 |
| GO:0009112 | nucleobase metabolic process | 58 | 0 | 1 | 0 | 0.446429 | 1 | 1 |
| GO:0009142 | nucleoside triphosphate biosynthetic process | 83 | 0 | 1 | 0 | 0.446429 | 1 | 1 |
| GO:0009145 | purine nucleoside triphosphate biosynthetic process | 81 | 0 | 1 | 0 | 0.446429 | 1 | 1 |
| GO:0009163 | nucleoside biosynthetic process | 26 | 0 | 1 | 0 | 0.446429 | 1 | 1 |
| GO:0009262 | deoxyribonucleotide metabolic process | 27 | 0 | 1 | 0 | 0.446429 | 1 | 1 |
| GO:0009267 | cellular response to starvation | 48 | 0 | 1 | 0 | 0.446429 | 1 | 1 |
| GO:0009451 | RNA modification | 52 | 0 | 1 | 0 | 0.446429 | 1 | 1 |
| GO:0009566 | fertilization | 84 | 0 | 1 | 0 | 0.446429 | 1 | 1 |
| GO:0009582 | detection of abiotic stimulus | 64 | 0 | 1 | 0 | 0.446429 | 1 | 1 |
| GO:0009620 | response to fungus | 28 | 0 | 1 | 0 | 0.446429 | 1 | 1 |
| GO:0009628 | response to abiotic stimulus | 431 | 0 | 1 | 0 | 0.446429 | 1 | 1 |
| GO:0009743 | response to carbohydrate stimulus | 95 | 0 | 1 | 0 | 0.446429 | 1 | 1 |
| GO:0009897 | external side of plasma membrane | 143 | 0 | 3 | 0 | 1.339286 | 1 | 1 |
| GO:0009898 | internal side of plasma membrane | 47 | 0 | 1 | 0 | 0.446429 | 1 | 1 |
| GO:0009991 | response to extracellular stimulus | 302 | 0 | 2 | 0 | 0.892857 | 1 | 1 |
| GO:0010035 | response to inorganic substance | 269 | 0 | 1 | 0 | 0.446429 | 1 | 1 |
| GO:0010469 | regulation of receptor activity | 29 | 0 | 1 | 0 | 0.446429 | 1 | 1 |
| GO:0012502 | induction of programmed cell death | 364 | 0 | 5 | 0 | 2.232143 | 1 | 1 |
| GO:0015030 | Cajal body | 45 | 0 | 1 | 0 | 0.446429 | 1 | 1 |
| GO:0015077 | monovalent inorganic cation transmembrane transporter activity | 195 | 0 | 2 | 0 | 0.892857 | 1 | 1 |
| GO:0015078 | hydrogen ion transmembrane transporter activity | 107 | 0 | 2 | 0 | 0.892857 | 1 | 1 |
| GO:0015081 | sodium ion transmembrane transporter activity | 96 | 0 | 1 | 0 | 0.446429 | 1 | 1 |
| GO:0015103 | inorganic anion transmembrane transporter activity | 46 | 0 | 1 | 0 | 0.446429 | 1 | 1 |
| GO:0015291 | secondary active transmembrane transporter activity | 201 | 0 | 3 | 0 | 1.339286 | 1 | 1 |
| GO:0015293 | symporter activity | 133 | 0 | 1 | 0 | 0.446429 | 1 | 1 |
| GO:0015294 | solute:cation symporter activity | 89 | 0 | 1 | 0 | 0.446429 | 1 | 1 |
| GO:0015296 | anion:cation symporter activity | 32 | 0 | 1 | 0 | 0.446429 | 1 | 1 |
| GO:0015297 | antiporter activity | 69 | 0 | 2 | 0 | 0.892857 | 1 | 1 |
| GO:0015298 | solute:cation antiporter activity | 34 | 0 | 2 | 0 | 0.892857 | 1 | 1 |
| GO:0015300 | solute:solute antiporter activity | 60 | 0 | 2 | 0 | 0.892857 | 1 | 1 |
| GO:0015629 | actin cytoskeleton | 293 | 0 | 2 | 0 | 0.892857 | 1 | 1 |
| GO:0015672 | monovalent inorganic cation transport | 342 | 0 | 3 | 0 | 1.339286 | 1 | 1 |
| GO:0015698 | inorganic anion transport | 81 | 0 | 1 | 0 | 0.446429 | 1 | 1 |
| GO:0015934 | large ribosomal subunit | 62 | 0 | 1 | 0 | 0.446429 | 1 | 1 |
| GO:0016052 | carbohydrate catabolic process | 145 | 0 | 1 | 0 | 0.446429 | 1 | 1 |
| GO:0016072 | rRNA metabolic process | 98 | 0 | 1 | 0 | 0.446429 | 1 | 1 |
| GO:0016125 | sterol metabolic process | 112 | 0 | 1 | 0 | 0.446429 | 1 | 1 |
| GO:0016209 | antioxidant activity | 53 | 0 | 3 | 0 | 1.339286 | 1 | 1 |
| GO:0016229 | steroid dehydrogenase activity | 29 | 0 | 1 | 0 | 0.446429 | 1 | 1 |
| GO:0016327 | apicolateral plasma membrane | 92 | 0 | 1 | 0 | 0.446429 | 1 | 1 |
| GO:0016485 | protein processing | 79 | 0 | 2 | 0 | 0.892857 | 1 | 1 |
| GO:0016604 | nuclear body | 222 | 0 | 1 | 0 | 0.446429 | 1 | 1 |
| GO:0016614 | oxidoreductase activity, acting on CH-OH group of donors | 118 | 0 | 1 | 0 | 0.446429 | 1 | 1 |
| GO:0016616 | oxidoreductase activity, acting on the CH-OH group of donors, NAD or NADP as acceptor | 106 | 0 | 1 | 0 | 0.446429 | 1 | 1 |
| GO:0016651 | oxidoreductase activity, acting on NADH or NADPH | 89 | 0 | 3 | 0 | 1.339286 | 1 | 1 |
| GO:0016706 | oxidoreductase activity, acting on paired donors, with incorporation or reduction of molecular oxygen, 2-oxoglutarate as one donor, and incorporation of one atom each of oxygen into both donors | 37 | 0 | 1 | 0 | 0.446429 | 1 | 1 |
| GO:0016757 | transferase activity, transferring glycosyl groups | 259 | 0 | 2 | 0 | 0.892857 | 1 | 1 |
| GO:0016758 | transferase activity, transferring hexosyl groups | 183 | 0 | 1 | 0 | 0.446429 | 1 | 1 |
| GO:0016791 | phosphatase activity | 254 | 0 | 1 | 0 | 0.446429 | 1 | 1 |
| GO:0016798 | hydrolase activity, acting on glycosyl bonds | 113 | 0 | 1 | 0 | 0.446429 | 1 | 1 |
| GO:0018196 | peptidyl-asparagine modification | 88 | 0 | 1 | 0 | 0.446429 | 1 | 1 |
| GO:0018279 | protein N-linked glycosylation via asparagine | 88 | 0 | 1 | 0 | 0.446429 | 1 | 1 |
| GO:0019199 | transmembrane receptor protein kinase activity | 84 | 0 | 1 | 0 | 0.446429 | 1 | 1 |
| GO:0019205 | nucleobase, nucleoside, nucleotide kinase activity | 46 | 0 | 1 | 0 | 0.446429 | 1 | 1 |
| GO:0019228 | regulation of action potential in neuron | 57 | 0 | 2 | 0 | 0.892857 | 1 | 1 |
| GO:0019838 | growth factor binding | 110 | 0 | 1 | 0 | 0.446429 | 1 | 1 |
| GO:0019842 | vitamin binding | 133 | 0 | 1 | 0 | 0.446429 | 1 | 1 |
| GO:0019897 | extrinsic to plasma membrane | 58 | 0 | 1 | 0 | 0.446429 | 1 | 1 |
| GO:0019898 | extrinsic to membrane | 96 | 0 | 1 | 0 | 0.446429 | 1 | 1 |
| GO:0022602 | ovulation cycle process | 76 | 0 | 2 | 0 | 0.892857 | 1 | 1 |
| GO:0022613 | ribonucleoprotein complex biogenesis | 198 | 0 | 1 | 0 | 0.446429 | 1 | 1 |
| GO:0022804 | active transmembrane transporter activity | 351 | 0 | 3 | 0 | 1.339286 | 1 | 1 |
| GO:0022832 | voltage-gated channel activity | 190 | 0 | 1 | 0 | 0.446429 | 1 | 1 |
| GO:0022890 | inorganic cation transmembrane transporter activity | 247 | 0 | 2 | 0 | 0.892857 | 1 | 1 |
| GO:0022900 | electron transport chain | 142 | 0 | 1 | 0 | 0.446429 | 1 | 1 |
| GO:0030016 | myofibril | 116 | 0 | 1 | 0 | 0.446429 | 1 | 1 |
| GO:0030017 | sarcomere | 98 | 0 | 1 | 0 | 0.446429 | 1 | 1 |
| GO:0030031 | cell projection assembly | 111 | 0 | 1 | 0 | 0.446429 | 1 | 1 |
| GO:0030035 | microspike assembly | 29 | 0 | 1 | 0 | 0.446429 | 1 | 1 |
| GO:0030100 | regulation of endocytosis | 80 | 0 | 1 | 0 | 0.446429 | 1 | 1 |
| GO:0030135 | coated vesicle | 199 | 0 | 1 | 0 | 0.446429 | 1 | 1 |
| GO:0030136 | clathrin-coated vesicle | 162 | 0 | 1 | 0 | 0.446429 | 1 | 1 |
| GO:0030278 | regulation of ossification | 97 | 0 | 1 | 0 | 0.446429 | 1 | 1 |
| GO:0030330 | DNA damage response, signal transduction by p53 class mediator | 96 | 0 | 1 | 0 | 0.446429 | 1 | 1 |
| GO:0030594 | neurotransmitter receptor activity | 72 | 0 | 2 | 0 | 0.892857 | 1 | 1 |
| GO:0030595 | leukocyte chemotaxis | 66 | 0 | 1 | 0 | 0.446429 | 1 | 1 |
| GO:0030674 | protein binding, bridging | 122 | 0 | 2 | 0 | 0.892857 | 1 | 1 |
| GO:0031072 | heat shock protein binding | 82 | 0 | 1 | 0 | 0.446429 | 1 | 1 |
| GO:0031091 | platelet alpha granule | 59 | 0 | 1 | 0 | 0.446429 | 1 | 1 |
| GO:0031093 | platelet alpha granule lumen | 47 | 0 | 1 | 0 | 0.446429 | 1 | 1 |
| GO:0031341 | regulation of cell killing | 32 | 0 | 1 | 0 | 0.446429 | 1 | 1 |
| GO:0031347 | regulation of defense response | 304 | 0 | 1 | 0 | 0.446429 | 1 | 1 |
| GO:0031349 | positive regulation of defense response | 159 | 0 | 1 | 0 | 0.446429 | 1 | 1 |
| GO:0031406 | carboxylic acid binding | 157 | 0 | 1 | 0 | 0.446429 | 1 | 1 |
| GO:0031589 | cell-substrate adhesion | 150 | 0 | 1 | 0 | 0.446429 | 1 | 1 |
| GO:0031594 | neuromuscular junction | 27 | 0 | 1 | 0 | 0.446429 | 1 | 1 |
| GO:0031623 | receptor internalization | 28 | 0 | 1 | 0 | 0.446429 | 1 | 1 |
| GO:0031667 | response to nutrient levels | 280 | 0 | 2 | 0 | 0.892857 | 1 | 1 |
| GO:0031668 | cellular response to extracellular stimulus | 116 | 0 | 1 | 0 | 0.446429 | 1 | 1 |
| GO:0031669 | cellular response to nutrient levels | 93 | 0 | 1 | 0 | 0.446429 | 1 | 1 |
| GO:0031902 | late endosome membrane | 67 | 0 | 1 | 0 | 0.446429 | 1 | 1 |
| GO:0031965 | nuclear membrane | 149 | 0 | 2 | 0 | 0.892857 | 1 | 1 |
| GO:0031983 | vesicle lumen | 51 | 0 | 1 | 0 | 0.446429 | 1 | 1 |
| GO:0032011 | ARF protein signal transduction | 48 | 0 | 3 | 0 | 1.339286 | 1 | 1 |
| GO:0032012 | regulation of ARF protein signal transduction | 48 | 0 | 3 | 0 | 1.339286 | 1 | 1 |
| GO:0032312 | regulation of ARF GTPase activity | 32 | 0 | 1 | 0 | 0.446429 | 1 | 1 |
| GO:0032507 | maintenance of protein location in cell | 78 | 0 | 2 | 0 | 0.892857 | 1 | 1 |
| GO:0032526 | response to retinoic acid | 64 | 0 | 1 | 0 | 0.446429 | 1 | 1 |
| GO:0032602 | chemokine production | 28 | 0 | 1 | 0 | 0.446429 | 1 | 1 |
| GO:0032642 | regulation of chemokine production | 26 | 0 | 1 | 0 | 0.446429 | 1 | 1 |
| GO:0033077 | T cell differentiation in thymus | 45 | 0 | 2 | 0 | 0.892857 | 1 | 1 |
| GO:0033189 | response to vitamin A | 76 | 0 | 1 | 0 | 0.446429 | 1 | 1 |
| GO:0033273 | response to vitamin | 121 | 0 | 1 | 0 | 0.446429 | 1 | 1 |
| GO:0033764 | steroid dehydrogenase activity, acting on the CH-OH group of donors, NAD or NADP as acceptor | 25 | 0 | 1 | 0 | 0.446429 | 1 | 1 |
| GO:0034329 | cell junction assembly | 142 | 0 | 1 | 0 | 0.446429 | 1 | 1 |
| GO:0034330 | cell junction organization | 156 | 0 | 1 | 0 | 0.446429 | 1 | 1 |
| GO:0034332 | adherens junction organization | 36 | 0 | 1 | 0 | 0.446429 | 1 | 1 |
| GO:0034470 | ncRNA processing | 193 | 0 | 3 | 0 | 1.339286 | 1 | 1 |
| GO:0034774 | secretory granule lumen | 48 | 0 | 1 | 0 | 0.446429 | 1 | 1 |
| GO:0035591 | signaling adaptor activity | 56 | 0 | 1 | 0 | 0.446429 | 1 | 1 |
| GO:0042098 | T cell proliferation | 82 | 0 | 1 | 0 | 0.446429 | 1 | 1 |
| GO:0042100 | B cell proliferation | 48 | 0 | 1 | 0 | 0.446429 | 1 | 1 |
| GO:0042102 | positive regulation of T cell proliferation | 49 | 0 | 1 | 0 | 0.446429 | 1 | 1 |
| GO:0042129 | regulation of T cell proliferation | 69 | 0 | 1 | 0 | 0.446429 | 1 | 1 |
| GO:0042165 | neurotransmitter binding | 81 | 0 | 2 | 0 | 0.892857 | 1 | 1 |
| GO:0042254 | ribosome biogenesis | 128 | 0 | 1 | 0 | 0.446429 | 1 | 1 |
| GO:0042277 | peptide binding | 212 | 0 | 1 | 0 | 0.446429 | 1 | 1 |
| GO:0042391 | regulation of membrane potential | 143 | 0 | 2 | 0 | 0.892857 | 1 | 1 |
| GO:0042552 | myelination | 46 | 0 | 1 | 0 | 0.446429 | 1 | 1 |
| GO:0042594 | response to starvation | 70 | 0 | 1 | 0 | 0.446429 | 1 | 1 |
| GO:0042632 | cholesterol homeostasis | 50 | 0 | 1 | 0 | 0.446429 | 1 | 1 |
| GO:0042698 | ovulation cycle | 82 | 0 | 2 | 0 | 0.892857 | 1 | 1 |
| GO:0042742 | defense response to bacterium | 125 | 0 | 1 | 0 | 0.446429 | 1 | 1 |
| GO:0042770 | signal transduction in response to DNA damage | 117 | 0 | 1 | 0 | 0.446429 | 1 | 1 |
| GO:0042923 | neuropeptide binding | 45 | 0 | 1 | 0 | 0.446429 | 1 | 1 |
| GO:0043112 | receptor metabolic process | 71 | 0 | 2 | 0 | 0.892857 | 1 | 1 |
| GO:0043122 | regulation of I-kappaB kinase/NF-kappaB cascade | 137 | 0 | 1 | 0 | 0.446429 | 1 | 1 |
| GO:0043123 | positive regulation of I-kappaB kinase/NF-kappaB cascade | 126 | 0 | 1 | 0 | 0.446429 | 1 | 1 |
| GO:0043176 | amine binding | 129 | 0 | 1 | 0 | 0.446429 | 1 | 1 |
| GO:0043178 | alcohol binding | 29 | 0 | 1 | 0 | 0.446429 | 1 | 1 |
| GO:0043235 | receptor complex | 133 | 0 | 2 | 0 | 0.892857 | 1 | 1 |
| GO:0043292 | contractile fiber | 123 | 0 | 1 | 0 | 0.446429 | 1 | 1 |
| GO:0043296 | apical junction complex | 89 | 0 | 1 | 0 | 0.446429 | 1 | 1 |
| GO:0043413 | macromolecule glycosylation | 167 | 0 | 1 | 0 | 0.446429 | 1 | 1 |
| GO:0043523 | regulation of neuron apoptosis | 99 | 0 | 1 | 0 | 0.446429 | 1 | 1 |
| GO:0043687 | post-translational protein modification | 170 | 0 | 1 | 0 | 0.446429 | 1 | 1 |
| GO:0044433 | cytoplasmic vesicle part | 305 | 0 | 1 | 0 | 0.446429 | 1 | 1 |
| GO:0044445 | cytosolic part | 154 | 0 | 1 | 0 | 0.446429 | 1 | 1 |
| GO:0044449 | contractile fiber part | 113 | 0 | 1 | 0 | 0.446429 | 1 | 1 |
| GO:0044450 | microtubule organizing center part | 65 | 0 | 1 | 0 | 0.446429 | 1 | 1 |
| GO:0044455 | mitochondrial membrane part | 135 | 0 | 1 | 0 | 0.446429 | 1 | 1 |
| GO:0045088 | regulation of innate immune response | 170 | 0 | 1 | 0 | 0.446429 | 1 | 1 |
| GO:0045089 | positive regulation of innate immune response | 122 | 0 | 1 | 0 | 0.446429 | 1 | 1 |
| GO:0045185 | maintenance of protein location | 85 | 0 | 2 | 0 | 0.892857 | 1 | 1 |
| GO:0045216 | cell-cell junction organization | 89 | 0 | 1 | 0 | 0.446429 | 1 | 1 |
| GO:0045580 | regulation of T cell differentiation | 56 | 0 | 2 | 0 | 0.892857 | 1 | 1 |
| GO:0045582 | positive regulation of T cell differentiation | 41 | 0 | 2 | 0 | 0.892857 | 1 | 1 |
| GO:0045597 | positive regulation of cell differentiation | 313 | 0 | 2 | 0 | 0.892857 | 1 | 1 |
| GO:0045619 | regulation of lymphocyte differentiation | 65 | 0 | 2 | 0 | 0.892857 | 1 | 1 |
| GO:0045621 | positive regulation of lymphocyte differentiation | 45 | 0 | 2 | 0 | 0.892857 | 1 | 1 |
| GO:0045667 | regulation of osteoblast differentiation | 56 | 0 | 1 | 0 | 0.446429 | 1 | 1 |
| GO:0045778 | positive regulation of ossification | 30 | 0 | 1 | 0 | 0.446429 | 1 | 1 |
| GO:0045807 | positive regulation of endocytosis | 48 | 0 | 1 | 0 | 0.446429 | 1 | 1 |
| GO:0046467 | membrane lipid biosynthetic process | 37 | 0 | 1 | 0 | 0.446429 | 1 | 1 |
| GO:0046847 | filopodium assembly | 28 | 0 | 1 | 0 | 0.446429 | 1 | 1 |
| GO:0046873 | metal ion transmembrane transporter activity | 151 | 0 | 1 | 0 | 0.446429 | 1 | 1 |
| GO:0046930 | pore complex | 85 | 0 | 1 | 0 | 0.446429 | 1 | 1 |
| GO:0048029 | monosaccharide binding | 45 | 0 | 1 | 0 | 0.446429 | 1 | 1 |
| GO:0048037 | cofactor binding | 256 | 0 | 2 | 0 | 0.892857 | 1 | 1 |
| GO:0048259 | regulation of receptor-mediated endocytosis | 27 | 0 | 1 | 0 | 0.446429 | 1 | 1 |
| GO:0048511 | rhythmic process | 161 | 0 | 2 | 0 | 0.892857 | 1 | 1 |
| GO:0048704 | embryonic skeletal system morphogenesis | 60 | 0 | 1 | 0 | 0.446429 | 1 | 1 |
| GO:0048705 | skeletal system morphogenesis | 128 | 0 | 1 | 0 | 0.446429 | 1 | 1 |
| GO:0048706 | embryonic skeletal system development | 81 | 0 | 1 | 0 | 0.446429 | 1 | 1 |
| GO:0048872 | homeostasis of number of cells | 107 | 0 | 1 | 0 | 0.446429 | 1 | 1 |
| GO:0050660 | flavin adenine dinucleotide binding | 71 | 0 | 2 | 0 | 0.892857 | 1 | 1 |
| GO:0050661 | NADP binding | 41 | 0 | 2 | 0 | 0.892857 | 1 | 1 |
| GO:0050662 | coenzyme binding | 181 | 0 | 2 | 0 | 0.892857 | 1 | 1 |
| GO:0050663 | cytokine secretion | 49 | 0 | 1 | 0 | 0.446429 | 1 | 1 |
| GO:0050707 | regulation of cytokine secretion | 40 | 0 | 1 | 0 | 0.446429 | 1 | 1 |
| GO:0050708 | regulation of protein secretion | 71 | 0 | 1 | 0 | 0.446429 | 1 | 1 |
| GO:0050714 | positive regulation of protein secretion | 50 | 0 | 1 | 0 | 0.446429 | 1 | 1 |
| GO:0050715 | positive regulation of cytokine secretion | 30 | 0 | 1 | 0 | 0.446429 | 1 | 1 |
| GO:0050777 | negative regulation of immune response | 30 | 0 | 1 | 0 | 0.446429 | 1 | 1 |
| GO:0050808 | synapse organization | 74 | 0 | 1 | 0 | 0.446429 | 1 | 1 |
| GO:0050863 | regulation of T cell activation | 173 | 0 | 3 | 0 | 1.339286 | 1 | 1 |
| GO:0050870 | positive regulation of T cell activation | 140 | 0 | 3 | 0 | 1.339286 | 1 | 1 |
| GO:0050905 | neuromuscular process | 57 | 0 | 1 | 0 | 0.446429 | 1 | 1 |
| GO:0051047 | positive regulation of secretion | 138 | 0 | 1 | 0 | 0.446429 | 1 | 1 |
| GO:0051050 | positive regulation of transport | 298 | 0 | 2 | 0 | 0.892857 | 1 | 1 |
| GO:0051087 | chaperone binding | 39 | 0 | 1 | 0 | 0.446429 | 1 | 1 |
| GO:0051130 | positive regulation of cellular component organization | 266 | 0 | 1 | 0 | 0.446429 | 1 | 1 |
| GO:0051216 | cartilage development | 112 | 0 | 1 | 0 | 0.446429 | 1 | 1 |
| GO:0051222 | positive regulation of protein transport | 94 | 0 | 1 | 0 | 0.446429 | 1 | 1 |
| GO:0051235 | maintenance of location | 145 | 0 | 2 | 0 | 0.892857 | 1 | 1 |
| GO:0051240 | positive regulation of multicellular organismal process | 313 | 0 | 2 | 0 | 0.892857 | 1 | 1 |
| GO:0051302 | regulation of cell division | 56 | 0 | 1 | 0 | 0.446429 | 1 | 1 |
| GO:0051402 | neuron apoptosis | 110 | 0 | 1 | 0 | 0.446429 | 1 | 1 |
| GO:0051604 | protein maturation | 88 | 0 | 2 | 0 | 0.892857 | 1 | 1 |
| GO:0051605 | protein maturation by peptide bond cleavage | 58 | 0 | 1 | 0 | 0.446429 | 1 | 1 |
| GO:0051606 | detection of stimulus | 120 | 0 | 1 | 0 | 0.446429 | 1 | 1 |
| GO:0051651 | maintenance of location in cell | 87 | 0 | 2 | 0 | 0.892857 | 1 | 1 |
| GO:0051656 | establishment of organelle localization | 85 | 0 | 1 | 0 | 0.446429 | 1 | 1 |
| GO:0051705 | behavioral interaction between organisms | 36 | 0 | 1 | 0 | 0.446429 | 1 | 1 |
| GO:0051781 | positive regulation of cell division | 44 | 0 | 1 | 0 | 0.446429 | 1 | 1 |
| GO:0055067 | monovalent inorganic cation homeostasis | 46 | 0 | 1 | 0 | 0.446429 | 1 | 1 |
| GO:0055088 | lipid homeostasis | 67 | 0 | 1 | 0 | 0.446429 | 1 | 1 |
| GO:0055092 | sterol homeostasis | 50 | 0 | 1 | 0 | 0.446429 | 1 | 1 |
| GO:0060021 | palate development | 45 | 0 | 1 | 0 | 0.446429 | 1 | 1 |
| GO:0060090 | binding, bridging | 125 | 0 | 2 | 0 | 0.892857 | 1 | 1 |
| GO:0060205 | cytoplasmic membrane-bounded vesicle lumen | 49 | 0 | 1 | 0 | 0.446429 | 1 | 1 |
| GO:0060326 | cell chemotaxis | 78 | 0 | 1 | 0 | 0.446429 | 1 | 1 |
| GO:0060627 | regulation of vesicle-mediated transport | 131 | 0 | 1 | 0 | 0.446429 | 1 | 1 |
| GO:0061008 | hepaticobiliary system development | 65 | 0 | 2 | 0 | 0.892857 | 1 | 1 |
| GO:0061035 | regulation of cartilage development | 29 | 0 | 1 | 0 | 0.446429 | 1 | 1 |
| GO:0061039 | ovum-producing ovary development | 65 | 0 | 2 | 0 | 0.892857 | 1 | 1 |
| GO:0070085 | glycosylation | 168 | 0 | 1 | 0 | 0.446429 | 1 | 1 |
| GO:0070160 | occluding junction | 78 | 0 | 1 | 0 | 0.446429 | 1 | 1 |
| GO:0070585 | protein localization in mitochondrion | 35 | 0 | 1 | 0 | 0.446429 | 1 | 1 |
| GO:0070997 | neuron death | 112 | 0 | 1 | 0 | 0.446429 | 1 | 1 |
| GO:0071496 | cellular response to external stimulus | 121 | 0 | 1 | 0 | 0.446429 | 1 | 1 |
| GO:0071813 | lipoprotein particle binding | 31 | 0 | 1 | 0 | 0.446429 | 1 | 1 |
| GO:0071814 | protein-lipid complex binding | 31 | 0 | 1 | 0 | 0.446429 | 1 | 1 |
| GO:0072594 | establishment of protein localization to organelle | 69 | 0 | 1 | 0 | 0.446429 | 1 | 1 |
| GO:0072595 | maintenance of protein localization to organelle | 26 | 0 | 2 | 0 | 0.892857 | 1 | 1 |
| GO:0072655 | establishment of protein localization in mitochondrion | 34 | 0 | 1 | 0 | 0.446429 | 1 | 1 |
| KEGG:00240 | KEGG: Pyrimidine metabolism | 99 | 0 | 3 | 0 | 1.339286 | 1 | 1 |
| KEGG:00512 | KEGG: O-Glycan biosynthesis | 30 | 0 | 1 | 0 | 0.446429 | 1 | 1 |
| KEGG:00520 | KEGG: Amino sugar and nucleotide sugar metabolism | 45 | 0 | 1 | 0 | 0.446429 | 1 | 1 |
| KEGG:03010 | KEGG: Ribosome | 88 | 0 | 1 | 0 | 0.446429 | 1 | 1 |
| KEGG:04144 | KEGG: Endocytosis | 205 | 0 | 3 | 0 | 1.339286 | 1 | 1 |
| KEGG:04145 | KEGG: Phagosome | 158 | 0 | 1 | 0 | 0.446429 | 1 | 1 |
| KEGG:04610 | KEGG: Complement and coagulation cascades | 69 | 0 | 1 | 0 | 0.446429 | 1 | 1 |
| KEGG:04630 | KEGG: Jak-STAT signaling pathway | 155 | 0 | 1 | 0 | 0.446429 | 1 | 1 |
| KEGG:04640 | KEGG: Hematopoietic cell lineage | 88 | 0 | 1 | 0 | 0.446429 | 1 | 1 |
| KEGG:04744 | KEGG: Phototransduction | 29 | 0 | 1 | 0 | 0.446429 | 1 | 1 |
| KEGG:05340 | KEGG: Primary immunodeficiency | 35 | 0 | 1 | 0 | 0.446429 | 1 | 1 |
| NCI:104 | IL4-mediated signaling events | 60 | 0 | 1 | 0 | 0.446429 | 1 | 1 |
| NCI:146 | Class I PI3K signaling events | 54 | 0 | 1 | 0 | 0.446429 | 1 | 1 |
| NCI:42 | C-MYB transcription factor network | 82 | 0 | 1 | 0 | 0.446429 | 1 | 1 |
| PF00019 | PFAM: Transforming growth factor beta like domain | 39 | 0 | 1 | 0 | 0.446429 | 1 | 1 |
| PF00036 | PFAM: EF hand | 87 | 0 | 2 | 0 | 0.892857 | 1 | 1 |
| PF00047 | PFAM: Immunoglobulin domain | 151 | 0 | 1 | 0 | 0.446429 | 1 | 1 |
| PF00059 | PFAM: Lectin C-type domain | 83 | 0 | 1 | 0 | 0.446429 | 1 | 1 |
| PF00092 | PFAM: von Willebrand factor type A domain | 57 | 0 | 2 | 0 | 0.892857 | 1 | 1 |
| PF00093 | PFAM: von Willebrand factor type C domain | 31 | 0 | 2 | 0 | 0.892857 | 1 | 1 |
| PF00106 | PFAM: short chain dehydrogenase | 57 | 0 | 1 | 0 | 0.446429 | 1 | 1 |
| PF00168 | PFAM: C2 domain | 133 | 0 | 1 | 0 | 0.446429 | 1 | 1 |
| PF00169 | PFAM: PH domain | 216 | 0 | 4 | 0 | 1.785714 | 1 | 1 |
| PF00307 | PFAM: Calponin homology (CH) domain | 74 | 0 | 1 | 0 | 0.446429 | 1 | 1 |
| PF00431 | PFAM: CUB domain | 51 | 0 | 2 | 0 | 0.892857 | 1 | 1 |
| PF00435 | PFAM: Spectrin repeat | 25 | 0 | 1 | 0 | 0.446429 | 1 | 1 |
| PF00505 | PFAM: HMG (high mobility group) box | 63 | 0 | 1 | 0 | 0.446429 | 1 | 1 |
| PF00620 | PFAM: RhoGAP domain | 73 | 0 | 2 | 0 | 0.892857 | 1 | 1 |
| PF01344 | PFAM: Kelch motif | 68 | 0 | 2 | 0 | 0.892857 | 1 | 1 |
| PF01412 | PFAM: Putative GTPase activating protein for Arf | 34 | 0 | 1 | 0 | 0.446429 | 1 | 1 |
| PF01421 | PFAM: Reprolysin (M12B) family zinc metalloprotease | 45 | 0 | 3 | 0 | 1.339286 | 1 | 1 |
| PF01562 | PFAM: Reprolysin family propeptide | 43 | 0 | 3 | 0 | 1.339286 | 1 | 1 |
| PF05986 | PFAM: ADAM-TS Spacer 1 | 27 | 0 | 2 | 0 | 0.892857 | 1 | 1 |
| PF07645 | PFAM: Calcium-binding EGF domain | 87 | 0 | 2 | 0 | 0.892857 | 1 | 1 |
| PF07653 | PFAM: Variant SH3 domain | 86 | 0 | 1 | 0 | 0.446429 | 1 | 1 |
| PF07686 | PFAM: Immunoglobulin V-set domain | 382 | 0 | 1 | 0 | 0.446429 | 1 | 1 |
| REACT:106 | REACT: GPCR ligand binding | 409 | 0 | 2 | 0 | 0.892857 | 1 | 1 |
| REACT:1066 | REACT: Recruitment of mitotic centrosome proteins and complexes | 71 | 0 | 1 | 0 | 0.446429 | 1 | 1 |
| REACT:155 | REACT: Centrosome maturation | 71 | 0 | 1 | 0 | 0.446429 | 1 | 1 |
| REACT:160 | REACT: Post-translational protein modification | 123 | 0 | 1 | 0 | 0.446429 | 1 | 1 |
| REACT:17 | REACT: Cell Cycle Checkpoints | 117 | 0 | 1 | 0 | 0.446429 | 1 | 1 |
| REACT:182 | REACT: Fatty acid, triacylglycerol, and ketone body metabolism | 112 | 0 | 1 | 0 | 0.446429 | 1 | 1 |
| REACT:189 | REACT: Cell-cell junction organization | 59 | 0 | 1 | 0 | 0.446429 | 1 | 1 |
| REACT:218 | REACT: Metabolism of nucleotides | 77 | 0 | 2 | 0 | 0.892857 | 1 | 1 |
| REACT:224 | REACT: Transport to the Golgi and subsequent modification | 36 | 0 | 1 | 0 | 0.446429 | 1 | 1 |
| REACT:257 | REACT: SLC-mediated transmembrane transport | 250 | 0 | 2 | 0 | 0.892857 | 1 | 1 |
| REACT:268 | REACT: G alpha (q) signalling events | 185 | 0 | 1 | 0 | 0.446429 | 1 | 1 |
| REACT:331 | REACT: Asparagine N-linked glycosylation | 85 | 0 | 1 | 0 | 0.446429 | 1 | 1 |
| REACT:353 | REACT: Regulation of Apoptosis | 60 | 0 | 1 | 0 | 0.446429 | 1 | 1 |
| REACT:372 | REACT: Rho GTPase cycle | 121 | 0 | 2 | 0 | 0.892857 | 1 | 1 |
| REACT:387 | REACT: Downstream TCR signaling | 49 | 0 | 1 | 0 | 0.446429 | 1 | 1 |
| REACT:391 | REACT: Amine ligand-binding receptors | 42 | 0 | 1 | 0 | 0.446429 | 1 | 1 |
| REACT:447 | REACT: Mitotic G2-G2/M phases | 86 | 0 | 1 | 0 | 0.446429 | 1 | 1 |
| REACT:505 | REACT: Regulation of Lipid Metabolism by Peroxisome proliferator-activated receptor alpha (PPARalpha) | 55 | 0 | 1 | 0 | 0.446429 | 1 | 1 |
| REACT:536 | REACT: Class A/1 (Rhodopsin-like receptors) | 304 | 0 | 2 | 0 | 0.892857 | 1 | 1 |
| REACT:618 | REACT: p75 NTR receptor-mediated signalling | 85 | 0 | 1 | 0 | 0.446429 | 1 | 1 |
| REACT:626 | REACT: Transport of inorganic cations/anions and amino acids/oligopeptides | 94 | 0 | 2 | 0 | 0.892857 | 1 | 1 |
| REACT:654 | REACT: Formation of Fibrin Clot (Clotting Cascade) | 32 | 0 | 1 | 0 | 0.446429 | 1 | 1 |
| REACT:697 | REACT: Loss of Nlp from mitotic centrosomes | 61 | 0 | 1 | 0 | 0.446429 | 1 | 1 |
| REACT:80 | REACT: Loss of proteins required for interphase microtubule organizationÂ from the centrosome | 61 | 0 | 1 | 0 | 0.446429 | 1 | 1 |
| REACT:806 | REACT: Apoptosis | 147 | 0 | 1 | 0 | 0.446429 | 1 | 1 |
| REACT:808 | REACT: Cell death signalling via NRAGE, NRIF and NADE | 63 | 0 | 1 | 0 | 0.446429 | 1 | 1 |
| REACT:853 | REACT: NRAGE signals death through JNK | 46 | 0 | 1 | 0 | 0.446429 | 1 | 1 |
| REACT:86 | REACT: Peptide ligand-binding receptors | 185 | 0 | 1 | 0 | 0.446429 | 1 | 1 |
| REACT:871 | REACT: TCR signaling | 66 | 0 | 1 | 0 | 0.446429 | 1 | 1 |
| REACT:874 | REACT: Cell junction organization | 97 | 0 | 1 | 0 | 0.446429 | 1 | 1 |
| REACT:887 | REACT: Purine metabolism | 32 | 0 | 1 | 0 | 0.446429 | 1 | 1 |
| REACT:90 | REACT: G2/M Transition | 83 | 0 | 1 | 0 | 0.446429 | 1 | 1 |
| REACT:900 | REACT: G alpha (12/13) signalling events | 76 | 0 | 1 | 0 | 0.446429 | 1 | 1 |
| REACT:903 | REACT: Adherens junctions interactions | 29 | 0 | 1 | 0 | 0.446429 | 1 | 1 |
| REACT:925 | REACT: Signaling by Rho GTPases | 121 | 0 | 2 | 0 | 0.892857 | 1 | 1 |

**Supplementary Table 8: Additional gene-set information for the 19 gene-sets selected for final results**

This supplementary table contains the rare CNVs restricted to exonic losses and is an extension of Supplementary table 5. Additional table fields: Support_size: number of support genes; a 'support gene' harbours rare CNV(s) more frequently in cases than OPGP controls. Support_symbol: official symbol of support genes (for 'support gene' definition see above). FETpv_remTop: gene-set association p-value (Fisher's Exact Test) after removing the 'Topgene' from the gene-set. Topgene: gene with the most significant association p-value

| GsID | GsName | GsSize | TOF_N | Ctrl_N | TOF_% | CTRL_% | FET_pv | FET_fdr | Support_size | Support_symbol | FETpv_remTop | Topgene |
| --- | --- | --- | --- | --- | --- | --- | --- | --- | --- | --- | --- | --- |
| GO:0030334 | regulation of cell migration | 230 | 5 | 0 | 2.512562814 | 0 | 0.022432462 | 0.64075 | 4 | FGF10,HDAC9,BCAR1,ANGPT2 | 0.103287931 | HDAC9 |
| GO:0051270 | regulation of cellular component movement | 258 | 5 | 0 | 2.512562814 | 0 | 0.022432462 | 0.64075 | 4 | HDAC9,BCAR1,ANGPT2,FGF10 | 0.103287931 | HDAC9 |
| GO:0051276 | chromosome organization | 609 | 5 | 0 | 2.512562814 | 0 | 0.022432462 | 0.64075 | 4 | HDAC9,H1FNT,BUB1,KDM4DL | 0.103287931 | HDAC9 |
| GO:2000145 | regulation of cell motility | 233 | 5 | 0 | 2.512562814 | 0 | 0.022432462 | 0.64075 | 4 | FGF10,HDAC9,BCAR1,ANGPT2 | 0.103287931 | HDAC9 |
| GO:0016477 | cell migration | 554 | 7 | 1 | 3.51758794 | 0.446428571 | 0.022981162 | 0.443357143 | 6 | DOCK1,BCAR1,PLXNA2,HDAC9,ANGPT2,FGF10 | 0.082538582 | HDAC9 |
| GO:0048870 | cell motility | 590 | 7 | 1 | 3.51758794 | 0.446428571 | 0.022981162 | 0.443357143 | 6 | DOCK1,BCAR1,PLXNA2,HDAC9,ANGPT2,FGF10 | 0.082538582 | HDAC9 |
| GO:0051674 | localization of cell | 590 | 7 | 1 | 3.51758794 | 0.446428571 | 0.022981162 | 0.443357143 | 6 | DOCK1,BCAR1,PLXNA2,HDAC9,ANGPT2,FGF10 | 0.082538582 | HDAC9 |
| GO:0001568 | blood vessel development | 330 | 8 | 2 | 4.020100503 | 0.892857143 | 0.03510492 | 0.383611111 | 6 | RECK,WARS2,HDAC9,ANGPT2,FGF10,DLX3 | 0.106924053 | WARS2 |
| GO:0001944 | vasculature development | 349 | 8 | 2 | 4.020100503 | 0.892857143 | 0.03510492 | 0.383611111 | 6 | RECK,WARS2,HDAC9,ANGPT2,FGF10,DLX3 | 0.106924053 | WARS2 |
| GO:0000904 | cell morphogenesis involved in differentiation | 548 | 6 | 1 | 3.015075377 | 0.446428571 | 0.043966885 | 0.268105263 | 6 | ATL1,DOCK1,RAF1,PLXNA2,NTNG1,CNTN6 | 0.082538582 | DOCK1 |
| GO:0006935 | chemotaxis | 520 | 6 | 1 | 3.015075377 | 0.446428571 | 0.043966885 | 0.268105263 | 6 | DOCK1,RAF1,PLXNA2,CNTN6,ANGPT2,FGF10 | 0.082538582 | DOCK1 |
| GO:0007409 | axonogenesis | 444 | 6 | 1 | 3.015075377 | 0.446428571 | 0.043966885 | 0.268105263 | 6 | ATL1,DOCK1,NTNG1,CNTN6,RAF1,PLXNA2 | 0.082538582 | DOCK1 |
| GO:0031175 | neuron projection development | 545 | 6 | 1 | 3.015075377 | 0.446428571 | 0.043966885 | 0.268105263 | 6 | ATL1,DOCK1,RAF1,PLXNA2,NTNG1,CNTN6 | 0.082538582 | DOCK1 |
| GO:0032990 | cell part morphogenesis | 529 | 6 | 1 | 3.015075377 | 0.446428571 | 0.043966885 | 0.268105263 | 6 | ATL1,DOCK1,RAF1,PLXNA2,NTNG1,CNTN6 | 0.082538582 | DOCK1 |
| GO:0042330 | taxis | 520 | 6 | 1 | 3.015075377 | 0.446428571 | 0.043966885 | 0.268105263 | 6 | DOCK1,RAF1,PLXNA2,CNTN6,ANGPT2,FGF10 | 0.082538582 | DOCK1 |
| GO:0048666 | neuron development | 615 | 6 | 1 | 3.015075377 | 0.446428571 | 0.043966885 | 0.268105263 | 6 | ATL1,DOCK1,RAF1,PLXNA2,NTNG1,CNTN6 | 0.082538582 | DOCK1 |
| GO:0048667 | cell morphogenesis involved in neuron differentiation | 478 | 6 | 1 | 3.015075377 | 0.446428571 | 0.043966885 | 0.268105263 | 6 | ATL1,DOCK1,NTNG1,CNTN6,RAF1,PLXNA2 | 0.082538582 | DOCK1 |
| GO:0048812 | neuron projection morphogenesis | 480 | 6 | 1 | 3.015075377 | 0.446428571 | 0.043966885 | 0.268105263 | 6 | ATL1,DOCK1,NTNG1,CNTN6,RAF1,PLXNA2 | 0.082538582 | DOCK1 |
| GO:0048858 | cell projection morphogenesis | 525 | 6 | 1 | 3.015075377 | 0.446428571 | 0.043966885 | 0.268105263 | 6 | ATL1,DOCK1,RAF1,PLXNA2,NTNG1,CNTN6 | 0.082538582 | DOCK1 |

**Supplementary Table 9: Known TOF disease genes used for the disease gene neighborhood analysis**

List was compiled using information from the entry in the Online Mendelian Inheritance in Man (OMIM) database for TOF (MIM *602054) and a review of literature for genes reported to be mutated in TOF patients.

| Gene symbol | Gene name |
| --- | --- |
| TBX1 | T-BOX 1 |
| TBX5 | T-BOX 5 |
| NKX2-5 | NK2 HOMEOBOX 5 |
| GATA4 | GATA-BINDING PROTEIN 4 |
| GATA6 | GATA-BINDING PROTEIN 6 |
| NOTCH1 | NOTCH, DROSOPHILA, HOMOLOG OF, 1 |
| JAG1 | JAGGED 1 |
| GDF1 | GROWTH/DIFFERENTIATION FACTOR 1 |
| ZFPM2 | ZINC FINGER PROTEIN, MULTITYPE 2 |

**Supplementary Table 10: Test results on disease gene neighborhoods for all disease genes, using the STRING network**

Significant disease genes have functional neighbors that harbour rare CNVs in cases more frequently than controls. Table fields: GeneID: EntrezGene ID of the disease gene. Symbol: official symbol of the disease gene. Name: gene name of the disease gene. Lg_xCoeff: x coefficient of the logistic regression; if positive, there are more case than OPGP control individuals harbouring CNVs on the disease gene neighbors; if negative, the opposite. Lg_Pvalue: logistic regression p-value (nominal). Lg_BH_FDR: Benjamini-Hochberg FDR q-value based on the logistic regression p-value (multiple test correction). Case%_w>0: fraction of case individuals with a score > 0; these have at least one CNV hitting at least one disease gene neighbor. Ctrl%_w>0: fraction of OPGP control individuals with a score > 0; these have at least one CNV hitting at least one disease gene neighbor. CaseN_w=max: fraction of case individuals with a maximal score; these have at least one CNV hitting the disease gene. CtrlN_w=max: fraction of OPGP control individuals with a maximal score; these have at least one CNV hitting the disease gene. LM_Pvalue, LM_BH_FDR: same as their Lg_.. counterparts, but using a linear instead of logistic regression model; p-values and FDR q-values are slightly improved. Note that coefficients for the linear model are the same as the values in the Lg_xCoeff column.

| GeneID | Symbol | Name | Lg_xCoeff | Lg_Pvalue | Lg_BH_FDR | Case%_w>0 | Ctrl%_w>0 | Case_max>w>0_mu | Ctrl_max>w>0_mu | CaseN _ w = max | CtrlN _ w = max | LM_Pvalue | LM_BH_FDR |
| --- | --- | --- | --- | --- | --- | --- | --- | --- | --- | --- | --- | --- | --- |
| 2626 | GATA4 | GATA binding protein 4 | 1 | 0.0616525 | 0.320325431 | 0.100502513 | 0.058035714 | 481.6 | 372 | 0 | 0 | 0.042318668 | 0.215387782 |
| 1482 | NKX2-5 | NK2 transcription factor related, locus 5 (Drosophila) | 1 | 0.071183429 | 0.320325431 | 0.105527638 | 0.040178571 | 478.7142857 | 534.5555556 | 0 | 0 | 0.047863952 | 0.215387782 |
| 6910 | TBX5 | T-box 5 | 1 | 0.144489728 | 0.433469183 | 0.065326633 | 0.040178571 | 467.1538462 | 313.6666667 | 0 | 0 | 0.093758558 | 0.281275675 |
| 4851 | NOTCH1 | notch 1 | 1 | 0.273122557 | 0.614525753 | 0.190954774 | 0.183035714 | 419.4736842 | 311.8 | 0 | 1 | 0.257998461 | 0.580496537 |
| 2627 | GATA6 | GATA binding protein 6 | 1 | 0.602255581 | 0.729901799 | 0.035175879 | 0.049107143 | 533.1428571 | 284 | 0 | 0 | 0.571827554 | 0.643305999 |
| 6899 | TBX1 | T-box 1 | 1 | 0.648801599 | 0.729901799 | 0.045226131 | 0.022321429 | 250.4444444 | 179.75 | 0 | 1 | 0.560382298 | 0.643305999 |
| 2657 | GDF1 | growth differentiation factor 1 | -1 | 0.97528585 | 0.97528585 | 0.015075377 | 0.013392857 | 287.3333333 | 341.3333333 | 0 | 0 | 0.949271546 | 0.949271546 |
| 23414 | ZFPM2 | zinc finger protein, multitype 2 | -1 | 0.580216239 | 0.729901799 | 0.025125628 | 0.035714286 | 303.6 | 354.625 | 0 | 0 | 0.466832671 | 0.643305999 |
| 182 | JAG1 | jagged 1 | -1 | 0.51333536 | 0.729901799 | 0.050251256 | 0.089285714 | 397.4 | 315.55 | 0 | 0 | 0.451732118 | 0.643305999 |

**Supplementary Table 11: Neighbor gene details for the three top disease genes Only neighbors with weight > 700, 2 case individuals with rare CNVs and no controls were selected for the final results. GeneID, Symbol and Name indicate the EntrezGene ID, official symbol and gene name respectively of the neighbor. CaseN and CtrlN indicate number of case and OPGP control individuals harboring a rare CNV on the neighbor gene. Weight: STRING edge weight, which is related to the probability that the disease gene and the neighbor belong to the same pathway**

| **GeneID** | **Symbol** | **Name** | **CaseN** | **CtrlN** | **Weight** |
| --- | --- | --- | --- | --- | --- |
| **Neighbors of GATA4** | | | | | |
| 2702 | GJA5 | gap junction protein, alpha 5, 40kDa | 3 | 0 | 780 |
| 9734 | HDAC9 | histone deacetylase 9 | 2 | 0 | 439 |
| 132625 | ZFP42 | zinc finger protein 42 homolog (mouse) | 1 | 0 | 720 |
| 2033 | EP300 | E1A binding protein p300 | 1 | 0 | 916 |
| 2100 | ESR2 | estrogen receptor 2 (ER beta) | 1 | 0 | 174 |
| 26586 | CKAP2 | cytoskeleton associated protein 2 | 1 | 0 | 215 |
| 4772 | NFATC1 | nuclear factor of activated T-cells, cytoplasmic, calcineurin-dependent 1 | 1 | 0 | 557 |
| 5595 | MAPK3 | mitogen-activated protein kinase 3 | 1 | 0 | 728 |
| 610 | HCN2 | hyperpolarization activated cyclic nucleotide-gated potassium channel 2 | 1 | 0 | 372 |
| 6659 | SOX4 | SRY (sex determining region Y)-box 4 | 1 | 0 | 260 |
| 7249 | TSC2 | tuberous sclerosis 2 | 1 | 0 | 235 |
| 79648 | MCPH1 | microcephalin 1 | 1 | 0 | 299 |
| 84676 | TRIM63 | tripartite motif containing 63 | 1 | 0 | 210 |
| 94137 | RP1L1 | retinitis pigmentosa 1-like 1 | 1 | 0 | 513 |
| 9564 | BCAR1 | breast cancer anti-estrogen resistance 1 | 1 | 0 | 274 |
| 5894 | RAF1 | v-raf-1 murine leukemia viral oncogene homolog 1 | 1 | 1 | 221 |
| 6546 | SLC8A1 | solute carrier family 8 (sodium/calcium exchanger), member 1 | 1 | 1 | 720 |
| **Neighbors of NKX2-5** | | | | | |
| 2702 | GJA5 | gap junction protein, alpha 5, 40kDa | 3 | 0 | 892 |
| 2255 | FGF10 | fibroblast growth factor 10 | 2 | 0 | 199 |
| 9734 | HDAC9 | histone deacetylase 9 | 2 | 0 | 958 |
| 10923 | SUB1 | SUB1 homolog (S. cerevisiae) | 1 | 0 | 899 |
| 11155 | LDB3 | LIM domain binding 3 | 1 | 0 | 243 |
| 196528 | ARID2 | AT rich interactive domain 2 (ARID, RFX-like) | 1 | 0 | 555 |
| 2033 | EP300 | E1A binding protein p300 | 1 | 0 | 655 |
| 26586 | CKAP2 | cytoskeleton associated protein 2 | 1 | 0 | 215 |
| 4772 | NFATC1 | nuclear factor of activated T-cells, cytoplasmic, calcineurin-dependent 1 | 1 | 0 | 188 |
| 5310 | PKD1 | polycystic kidney disease 1 (autosomal dominant) | 1 | 0 | 227 |
| 5617 | PRL | prolactin | 1 | 0 | 216 |
| 608 | TNFRSF17 | tumor necrosis factor receptor superfamily, member 17 | 1 | 0 | 232 |
| 610 | HCN2 | hyperpolarization activated cyclic nucleotide-gated potassium channel 2 | 1 | 0 | 254 |
| 6820 | SULT2B1 | sulfotransferase family, cytosolic, 2B, member 1 | 1 | 0 | 217 |
| 6911 | TBX6 | T-box 6 | 1 | 0 | 733 |
| 781 | CACNA2D1 | calcium channel, voltage-dependent, alpha 2/delta subunit 1 | 1 | 0 | 183 |
| 84676 | TRIM63 | tripartite motif containing 63 | 1 | 0 | 246 |
| **Neighbors of TBX5** | | | | | |
| 2702 | GJA5 | gap junction protein, alpha 5, 40kDa | 3 | 0 | 822 |
| 2255 | FGF10 | fibroblast growth factor 10 | 2 | 0 | 952 |
| 9734 | HDAC9 | histone deacetylase 9 | 2 | 0 | 221 |
| 1462 | VCAN | versican | 1 | 0 | 180 |
| 2033 | EP300 | E1A binding protein p300 | 1 | 0 | 194 |
| 2220 | FCN2 | ficolin (collagen/fibrinogen domain containing lectin) 2 (hucolin) | 1 | 0 | 224 |
| 6911 | TBX6 | T-box 6 | 1 | 0 | 171 |
| 9351 | SLC9A3R2 | solute carrier family 9 (sodium/hydrogen exchanger), member 3 regulator 2 | 1 | 0 | 271 |
| 5894 | RAF1 | v-raf-1 murine leukemia viral oncogene homolog 1 | 1 | 1 | 221 |

**Supplementary Figure 1: Overview of study design and CNV analysis workflow**

**
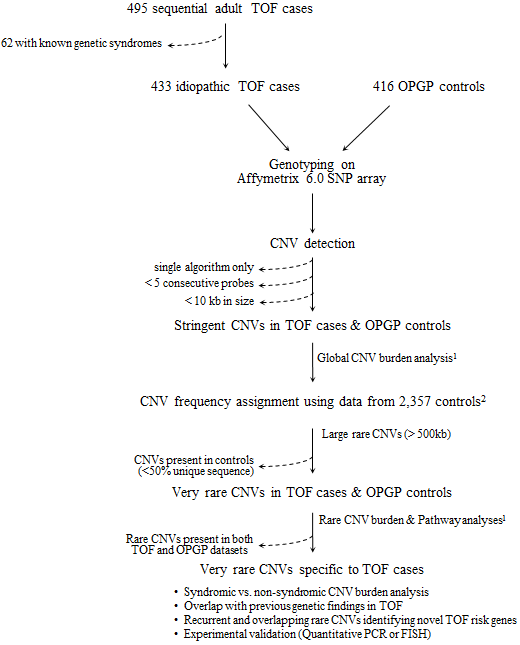
**

^1^CNV burden and pathway analysis focused on comparing subset of TOF cases of European ancestry (n = 340) with ancestry-matched OPGP controls (n=416).^2^ Population based controls of European ancestry (n=2,357) were genotyped on the Affymetrix 6.0 platform and analyzed for CNVs in an identical manner to the TOF cases and OPGP controls.

**Supplementary Figure 2: Rare CNVs at chromosome region 1q21.1 in TOF cases**


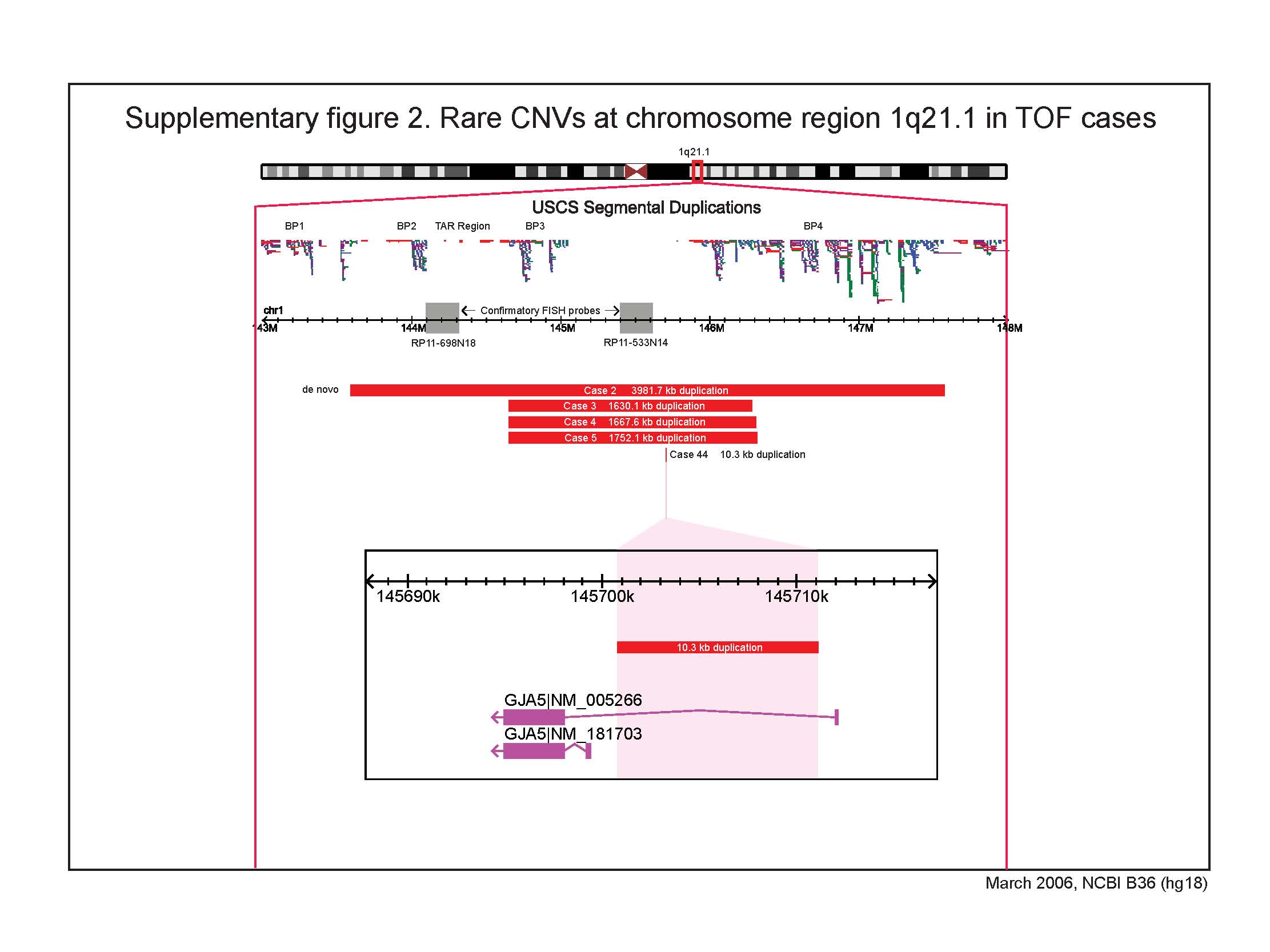
10.3 kb duplication

**Supplementary Figure 3: Rare CNVs at chromosome region 18q22.1-q23 in TOF cases**

**
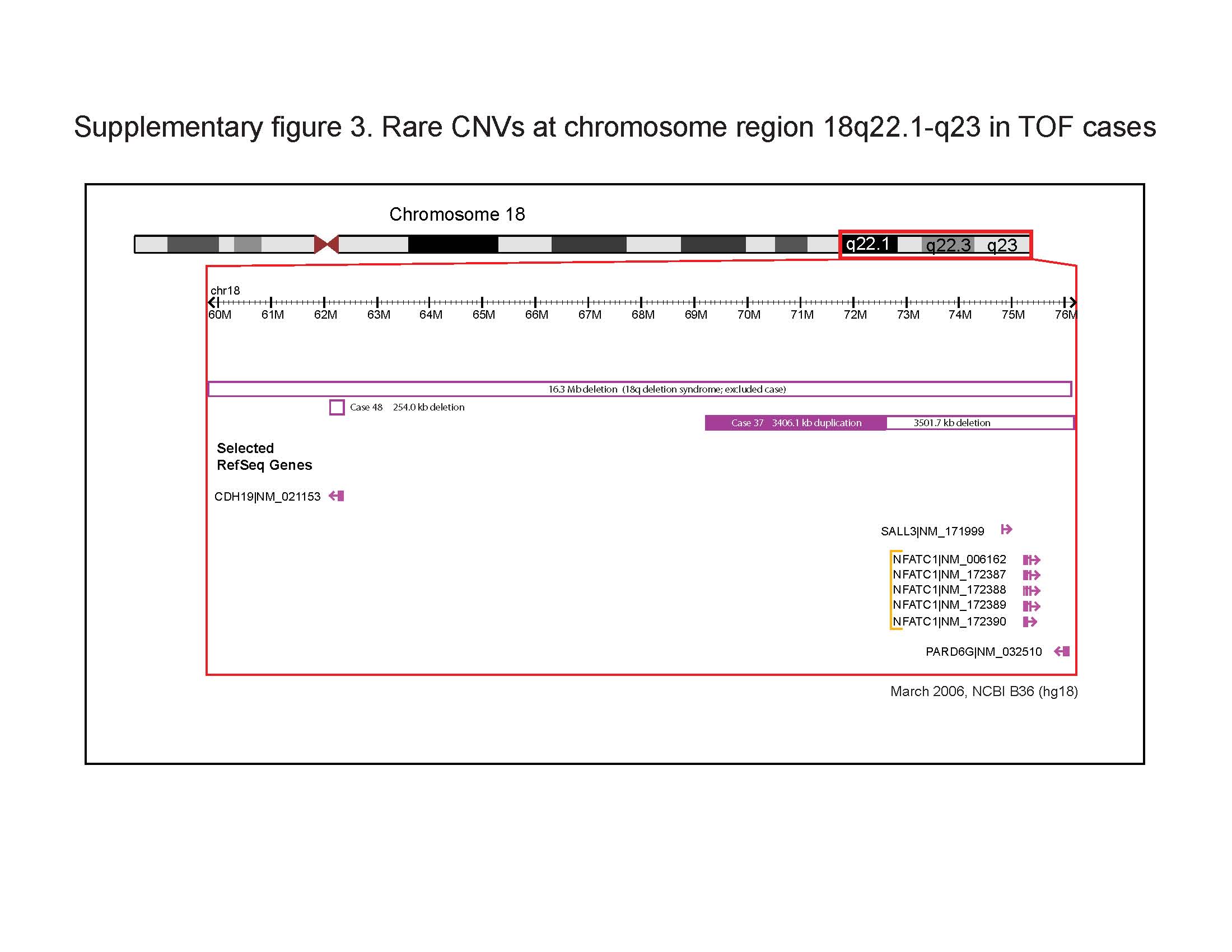
**

**Supplementary Figure 4: Integrated TOF pathway and candidate gene connectivity**

**
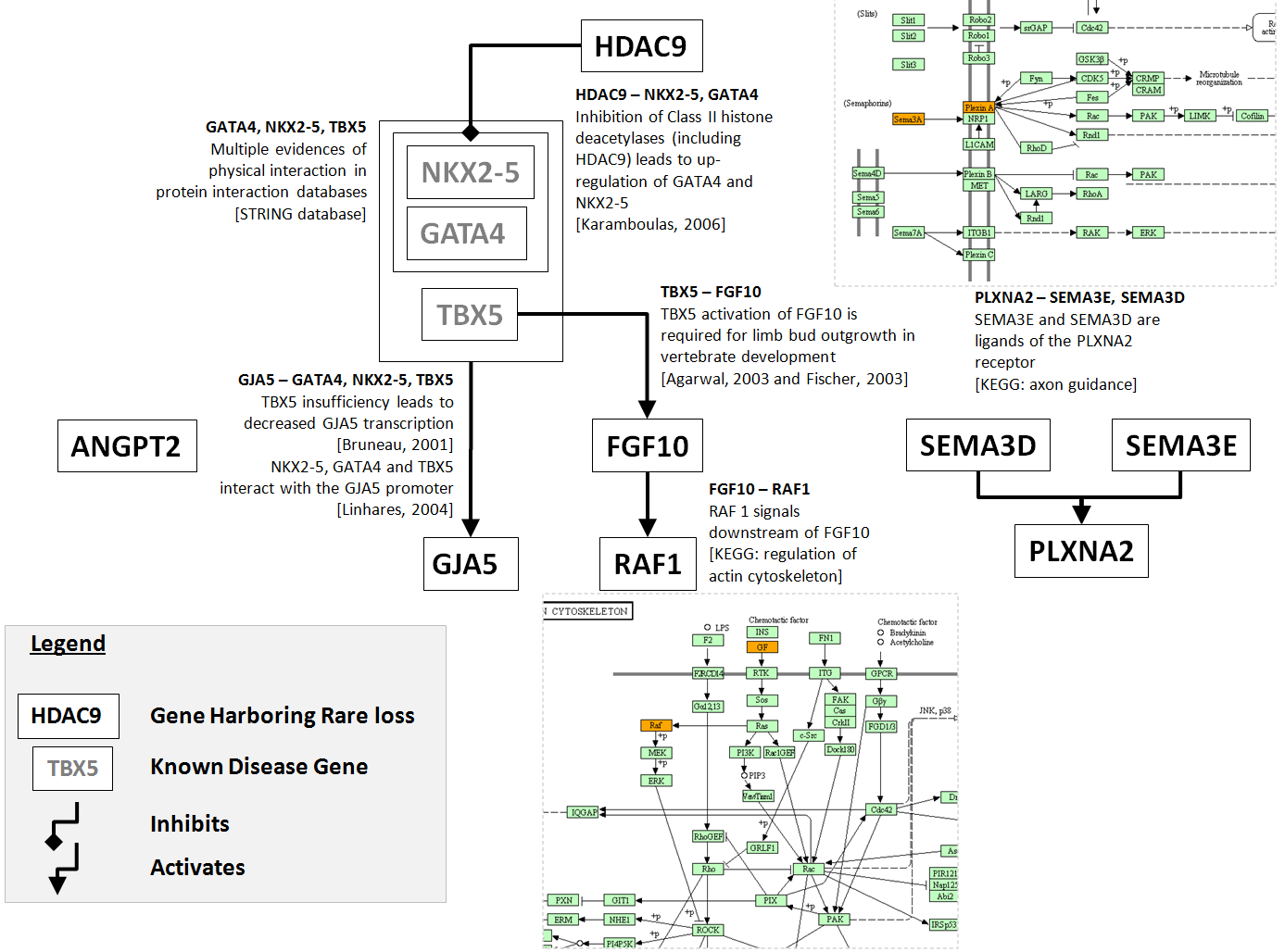
**

Candidate genes identified through pathway analyses and CNV manual curation were connected through bona fide regulatory relations. First, we queried the KEGG database for pathways that included the candidate genes identified. Only a few KEGG pathways were found, with a maximum 2 or 3 of the 8 candidate genes (shown in bold font). We selected two pathways that were particularly representative: regulation of actin cytoskeleton (related to cell motility, genes found: FGF10 and RAF1) and axon guidance (related to axonogenesis, genes found: PLXNA2 and SEMA3D, SEMA3E). We intersected the regulatory interactions reported by KEGG with predicted interactions according to the STRING database. We found that disease genes whose functional neighborhood displayed greater prevalence of cases with rare loss CNVs compared to controls (GATA4, NKX2-5, TBX5) were important for connecting candidate genes. We manually reviewed publications supporting STRING interactions based on co-citation and pruned spurious interactions. The studies cited in the figure are Karamboulas, 2006 [130], Agarwal, 2003 [131], Fischer, 2003 [132], Bruneau, 2001 [133] and Linhares, 2004 [134].

**Supplementary References**
